# Latent disconnectome prediction of long-term cognitive-behavioural symptoms in stroke

**DOI:** 10.1093/brain/awad013

**Published:** 2023-03-16

**Authors:** Lia Talozzi, Stephanie J Forkel, Valentina Pacella, Victor Nozais, Etienne Allart, Céline Piscicelli, Dominic Pérennou, Daniel Tranel, Aaron Boes, Maurizio Corbetta, Parashkev Nachev, Michel Thiebaut de Schotten

**Affiliations:** Groupe d’Imagerie Neurofonctionnelle, Institut des Maladies Neurodégénératives-UMR 5293, CNRS, CEA, University of Bordeaux, Bordeaux, 33076, France; Brain Connectivity and Behaviour Laboratory, Sorbonne Universities, Paris, 75006, France; Department of Neurology and Neurological Sciences, Stanford University School of Medicine, Stanford, CA, 94305, USA; Brain Connectivity and Behaviour Laboratory, Sorbonne Universities, Paris, 75006, France; Donders Centre for Cognition, Radboud University, 6525 GD Nijmegen, The Netherlands; Centre for Neuroimaging Sciences, Department of Neuroimaging, Institute of Psychiatry, Psychology and Neuroscience, King’s College London, London, SE5 8AF, UK; Departments of Neurosurgery, Technical University of Munich School of Medicine, Munich, 81675, Germany; Groupe d’Imagerie Neurofonctionnelle, Institut des Maladies Neurodégénératives-UMR 5293, CNRS, CEA, University of Bordeaux, Bordeaux, 33076, France; Brain Connectivity and Behaviour Laboratory, Sorbonne Universities, Paris, 75006, France; Scuola Universitaria Superiore IUSS, Pavia, 27100, Italy; Groupe d’Imagerie Neurofonctionnelle, Institut des Maladies Neurodégénératives-UMR 5293, CNRS, CEA, University of Bordeaux, Bordeaux, 33076, France; Brain Connectivity and Behaviour Laboratory, Sorbonne Universities, Paris, 75006, France; CHU Lille, Neurorehabilitation Unit, Lille, 59000, France; Universitaire Lille, INSERM UMR1172-Lille Neuroscience and Cognition, Lille, 59000, France; Laboratoire de Psychologie et Neurocognition, CNRS UMR5105, Université Grenoble-Alpes, Grenoble cedex 9, 38040, France; Service de Rééducation Neurologique, Institut de Rééducation, Hôpital Sud, CHU de Grenoble-Alpes, Échirolles, 38834, France; Laboratoire Psychology and Neurocognition, University Grenoble-Alpes, Service de Rééducation Neurologique, Institut de Rééducation, Hôpital sud-CHU Grenoble-Alpes, 38043 Grenoble, France; Department of Psychological and Brain Sciences, University of Iowa, Iowa City, IA 52242, USA; Department of Neurology, Carver College of Medicine, Iowa City, IA 52242, USA; Departments of Neurology, Psychiatry, and Pediatrics, Carver College of Medicine, Iowa City, IA 52242, USA; Clinica Neurologica, Department of Neuroscience, University of Padova, Padova, 32122, Italy; Padova Neuroscience Center (PNC), University of Padova, Padova, 32122, Italy; Venetian Institute of Molecular Medicine, VIMM, Padova, 32122, Italy; Department of Brain Repair and Rehabilitation, Institute of Neurology, UCL, London, WC1N 3AZ, UK; Groupe d’Imagerie Neurofonctionnelle, Institut des Maladies Neurodégénératives-UMR 5293, CNRS, CEA, University of Bordeaux, Bordeaux, 33076, France; Brain Connectivity and Behaviour Laboratory, Sorbonne Universities, Paris, 75006, France

**Keywords:** stroke, prognostic predictors, disconnectome, neuropsychology, web application

## Abstract

Stroke significantly impacts the quality of life. However, the long-term cognitive evolution in stroke is poorly predictable at the individual level. There is an urgent need to better predict long-term symptoms based on acute clinical neuroimaging data. Previous works have demonstrated a strong relationship between the location of white matter disconnections and clinical symptoms. However, rendering the entire space of possible disconnection-deficit associations optimally surveyable will allow for a systematic association between brain disconnections and cognitive-behavioural measures at the individual level. Here we present the most comprehensive framework, a composite morphospace of white matter disconnections (disconnectome) to predict neuropsychological scores 1 year after stroke. Linking the latent disconnectome morphospace to neuropsychological outcomes yields biological insights that are available as the first comprehensive atlas of disconnectome-deficit relations across 86 scores—a Neuropsychological White Matter Atlas. Our novel predictive framework, the Disconnectome Symptoms Discoverer, achieved better predictivity performances than six other models, including functional disconnection, lesion topology and volume modelling. Out-of-sample prediction derived from this atlas presented a mean absolute error below 20% and allowed personalize neuropsychological predictions. Prediction on an external cohort achieved an *R*^2^ = 0.201 for semantic fluency. In addition, training and testing were replicated on two external cohorts achieving an *R*^2^ = 0.18 for visuospatial performance.

This framework is available as an interactive web application (http://disconnectomestudio.bcblab.com) to provide the foundations for a new and practical approach to modelling cognition in stroke. We hope our atlas and web application will help to reduce the burden of cognitive deficits on patients, their families and wider society while also helping to tailor future personalized treatment programmes and discover new targets for treatments. We expect our framework’s range of assessments and predictive power to increase even further through future crowdsourcing.

## Teaser

Talozzi *et al.* report that long-term stroke symptoms can be predicted individually based on acute clinical neuroimaging with a mean absolute error below 20%, and provide the first atlas of disconnectome-deficit relationships. The model is available as an interactive web application (http://disconnectomestudio.bcblab.com).

## Introduction

The fidelity of lesion-deficit models depends not only on the quality of the data but also on the underlying theoretical framework. Together they establish the evidence of a relationship between the location of brain lesions and clinical symptoms such as visuospatial neglect,^1–3^ aphasias,^4–12^ apraxias^13,14^ or motor anosognosia,^15–18^ among others. The associations between anatomical white matter networks and clinical presentations have revealed no one-to-one relationship between structures and clinical presentation, as different lesions can cause the same functional impairments.^19–23^ One example would be that a stroke in the middle or posterior cerebral artery may lead to visuospatial neglect,^24^ just like different perisylvian white matter disconnections can lead to aphasia.^20^ Despite its importance,^25^ current methodologies do not systematically capture the potential overlap between brain signatures and clinical manifestations nor the distributed nature of their neural substrate. Therefore, a comprehensive framework that systematically associates brain disconnections with cognitive-behavioural assessments is needed for accurate and reliable precision medicine.^26–31^

We hypothesize that quantifying brain connections will provide more accurate predictors of long-term brain functioning. This hypothesis is based on previous stroke recovery investigations^32^ and on the rationale that our brains work as an interconnected network, and not as segregated entities.^33^

Beneath the surface complexity, there may lie a simpler order that can be described within a compact representational space. Namely, an anatomical lesion described by the presence or absence of damage across thousands of anatomical circuits in imaging space can be reduced to a two-dimensional Cartesian space. The patient’s coordinate, in this case, summarizes the lesion load on the surrounding white matter tracts. As such, dimensionality reduction algorithms allow defining low-dimensional spaces that can embed multivariate data. In embedding spaces, also known as morphospace,^34,35^ patients with similar disconnectivity patterns will cluster together, while dissimilar disconnectivity patterns will be located far apart.^21,36,37^ Morphospaces render lesion-deficit relations more easily surveyable, allowing correlation analyses based on patients’ embedding coordinates. Hence, specific brain features can define territories within a morphospace and help predict symptoms and brain pathologies, similar to typical machine learning approaches.^38,39^ Artificial intelligence (AI) has recently progressed in modelling the association of symptom severity with medical imaging modalities, e.g. reaching high accuracy and sensitivity in the characterization of tumour tissues.^40^ However, AI models need to be refined with a broader spectrum of clinically practical end points, including neuropsychological measures. The next challenge will be making AI patient-centric for a more effective deployment into the clinical routine and potentially benefiting patients’ quality of life.^41^

To drive this challenge forward, we propose a modelling approach that employs a morphospace to predict neuropsychological assessments of one of the most common neurological disorders: stroke.^42^ We first mapped the distribution of 1333 brain disconnection patterns in stroke—the disconnectome morphospace. A second dataset (training set) with rich neuropsychological measures 1 year after stroke was imported into this disconnectome morphospace. This second dataset enriched the morphospace with clinical symptoms obtained from 86 neuropsychological assessments. An out-of-sample validation set with the same neuropsychological data assessed prediction accuracy. This procedure, referred to as Disconnectome Symptoms Discoverer (DSD), reliably predicted the performance of patients with a mean absolute error below 20%. To make the DSD tool readily available to the clinical–academic community and facilitate its translation and incorporation into the clinic, we provide an open-access web application (http://disconnectomestudio.bcblab.com), in which individual disconnection patterns can be uploaded to predict the expected 1-year neuropsychological scores. We also demonstrated the DSD model generalizability by including three external cohorts. The web application will be interactively updated, thanks to future crowdsourcing, informing the DSD model with any newly available datasets.

## Materials and methods

**Figure 1:**
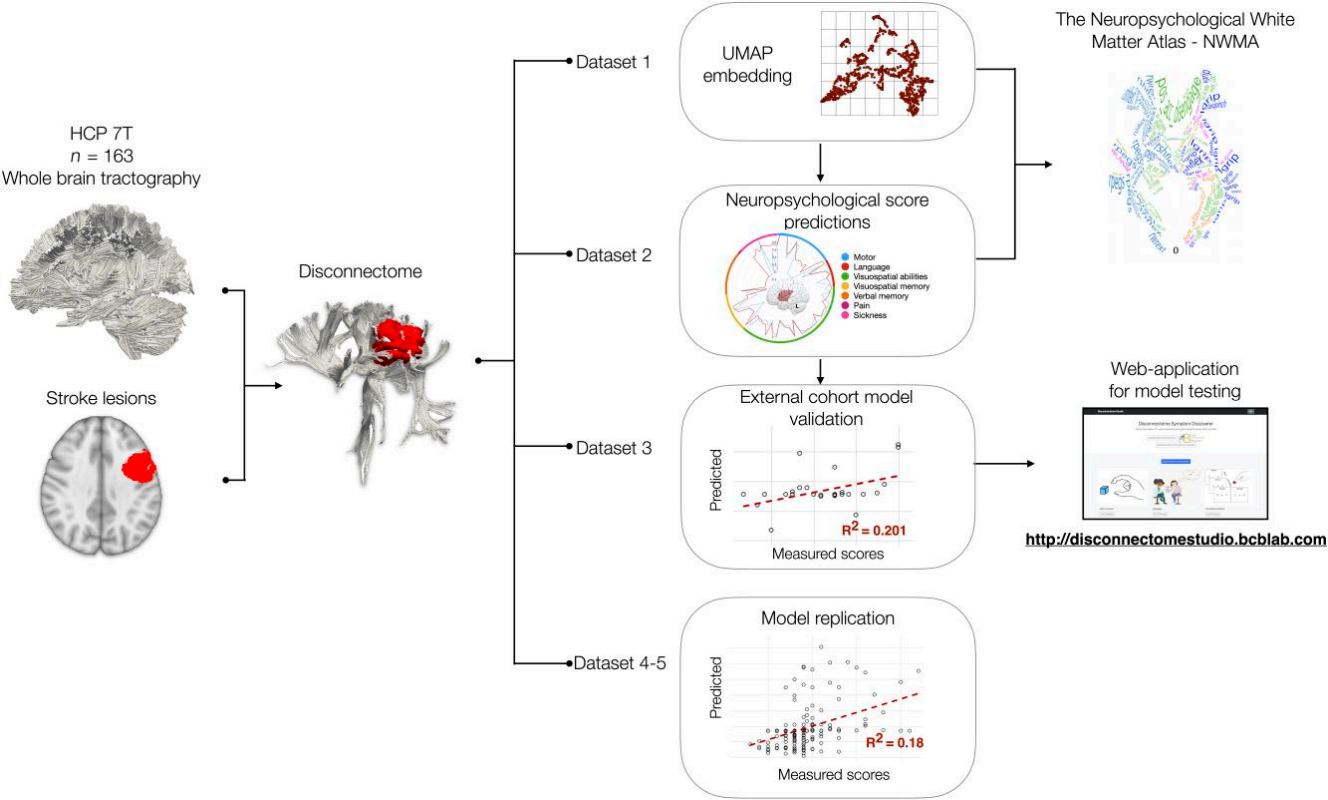
**Graphical summary of the analysis workflow**. Normative disconnectomes were derived from Human Connectome Project (HCP) participants and the patient’s lesion location. Dataset 1 defined the disconnectome morphospace (a latent space of two dimensions). Dataset 2 was imported into dataset 1’s morphospace to capture the neuropsychological scores’ variability and their voxel-wise correlations —the Disconnectome Symptoms Discoverer (DSD). This analysis extended to the 86 neuropsychological scores and allowed us to create a Neuropsychological White Matter Atlas (NWMA). Dataset 3 served as an out-of-sample validation of the DSD prediction. As an external cohorts’ model replication, dataset 4 served as a training set of the DSD method and dataset 5 as external validation. For broader model testing, the DSD calculation was integrated into an open-access interactive web application (http://disconnectomestudio.bcblab.com). Full details are provided in Supplementary Fig. 1 and the Supplementary material.

Bash and Python programming languages were used for automatizing all the processing steps summarized in Fig. 1 and with more details in Supplementary Fig. 1.

### Stroke lesions

Information on datasets is summarized in Table 1.

**Table 1:** Demographic information

	Dataset 1: UMAP	Dataset 2: training	Dataset 2: validation	Dataset 3: validation	Dataset 4: training	Dataset 5: validation
Recruitment site	University College London Hospitals, London (UK)	School of Medicine of the Washington University, St. Louis (USA)	School of Medicine of the Washington University, St. Louis (USA)	Iowa Neurological Patient Registry (USA)	Centre Hospitalier Universitaire Lille (FR)	Centre Hospitalier Universitaire Grenoble Alpes (FR)
Conducted analyses	UMAP embedding, NWMA derivation	DSD model training, NWMA derivation, 86 scores	DSD out-of-sample testing, 86 scores	DSD external cohort testing, 1 scores	DSD training-replication, 1 score	DSD external cohort testing-replication,1 score
**Demographics**						
Patients, *n*	1333	119	20	26	190	193
Males/females, *n*	748/585	65/54	12/8	12/14	86/104	126/67
Age, years	64 ± 16 (18–97)	54 ± 11 (19–83)	58 ± 12 (34–95)	55 ± 15.7 (14–92)	58.4 ± 15.4 (18–84)	63.7 ± 12 (18–79)
Education, years	NA	13.2 ± 2.5 (5–20)	13.7 ± 2.6 (9–19)	12.4 ± 2.7 (8–20)	NA	NA
Right-handed/left-handed, *n*	NA	109/10	17/3	25/1	177/13	178/15
**MRI**						
Chronology	1–2 weeks after stroke	14 ± 8 days after stroke	13 ± 4 days after stroke	1.5 ± 4.5 days after stroke	15–30 days after stroke	30 days after stroke
Dominant lesion site	44% right hemisphere 56% left hemisphere	46% right hemisphere 54% left hemisphere	40% right hemisphere 60% left hemisphere	42% right hemisphere 58% left hemisphere	63% right hemisphere 37% left hemisphere	52% right hemisphere 48% left hemisphere
**Neuropsychological assessment**						
Delay after stroke onset, days	NA	393 ± 56	385 ± 22	339 ± 219	52.9 ± 20.8	41 ± 16.9
Delay after MRI scan, days	NA	379 ± 57	373 ± 22	338 ± 219	NA	NA

Values are presented as mean ± SD (range). NA = not available; UMAP = Uniform Manifold Approximation and Projection method.

Lesion data were derived from five different international centres (datasets 1–5). Across all five datasets acute MRI data was acquired within 2 weeks of stroke onset. Dataset 1: (*n* = 1333 participants) MRI scans acquired at University College London Hospitals.^37,43^ MRI scans (1.5 T and 3 T) were acquired across several scanners during the routine clinical care of patients presenting with acute ischaemic stroke. Patients were on average 64 ± 16 years old (age range: 18–97 years) and 56% were male. This cohort was recruited as part of a study approved by the West London and GTAC Research Ethics Committee. Dataset 2 was recruited at the School of Medicine at Washington University in St. Louis and included both MRI and neuropsychological assessments.^25^ The MRI clinical scans were acquired with a standardized 3 T protocol. The MRI clinical scans were acquired with a standardized 3 T protocol. The neuropsychological assessments evaluated for dataset 2 were conducted on average 1 year post-stroke onset (see Table 1 for more information). All dataset 2 participants provided informed consent following the Declaration of Helsinki (2013) and procedures established by the Washington University in Saint Louis Institutional Review Board. Dataset 2 was divided into two independent subgroups: dataset 2-training and dataset 2-validation. From dataset 2 a subgroup of patients (dataset 2-validation) was randomly selected with the constraint that the subgroup’s lesion variability map was balanced across brain hemispheres and vascular territories (Supplementary Fig. 2) and the dataset 2-validation gender and age distributions were comparable to dataset 2-training. As such, dataset 2-validation was used as a patient population representative external dataset. For dataset 2-training (*n* = 119 participants), the patient average age was 54 ± 11 years (age range: 19–83 years) and 54.6% were male. The average education level was 13 ± 2.5 years and 91.6% were right-handed. For dataset 2-validation (*n* = 20 participants), the average age was 58 ± 12 years (age range: 34–95 years), with 60% male; average education level was 14 ± 2.6 years and 85% were right-handed.

Dataset 3 contains *n* = 26 stroke patients [average age 55 ± 15.7 years (age range: 14–92 years), with 46% male, average education level was 12.4 ± 2.7 years and 96% were right-handed] selected from the Iowa Neurological Patient Registry (USA).^44^

Dataset 4 contains *n* = 190 stroke patients (average age 58.4 ± 15.4 years [age range: 8–84 years], with 45% male and 93% right-handed) recruited from the Centre Hospitalier Universitaire Lille (France).

Dataset 5 contains *n* = 193 stroke patients (average age 63.7 ± 12 years [age range: 18–79 years], with 65% male and 92% right-handed) recruited from the Centre Hospitalier Universitaire Grenoble Alpes (France). MRI clinical scans were acquired with a standardized 1.5 T protocol within 15–30 days after stroke onset. In these datasets the Bells Test was performed between 30 and 90 days after stroke onset.^45^ Each cohort was approved by the local institutional review board (CHU de Lille for DISCONEGLECT, CHU Grenoble Alpes for DOBRAS) and registered at the National Committee for Informatics and Freedom (Commission Nationale Informatique et Liberté). According to French Law, observational studies do not require approval by a national ethics committee.

### Neuropsychological scores

A total of *n* = 86 neuropsychological scores were available for dataset 2. The details of each neuropsychological evaluation (grading, test battery, administration) are reported in the Supplementary material, Section C. In brief, motor abilities (Section C.1) were assessed for upper limb hand grasping, gripping, pinching, grip strength, peg replacement, motion shoulder flexion, wrist extension, and lower limb walking. Language abilities (Section C.2) were assessed using picture naming, non-word repetition, commands, sentence reading, sentence comprehension and semantic fluency. Visuospatial abilities (Section C.3) were tested for using discrimination accuracy, reaction time, subbing, behavioural inattention, and unstructured symbol cancellation. Visuospatial memory (Section C.4) was evaluated using abstract figures retrieval scores and verbal memory (Section C.5) for listed word recognition scores. A pain scale during the MRI scanning was recorded (Section C.6) and a stroke sickness questionnaire administered, investigating physical and psychosocial daily sickness (Section C.7). Despite some scores’ collinearity, most evident for visuospatial abilities, we chose to use single score measures instead of combined score indices,^46,47^ to remain data-driven and clinically compatible with individual patients’ measures.

Semantic fluency (animals) was the only comparable test between dataset 2 and dataset 3 and, accordingly, was chosen to perform the external validation of the prediction model. On average, the time of assessment after stroke was 11.1 ± 7.2 months (3.7–29.4 months).

For datasets 4 and 5, the Bells Test, a cancellation test, was chosen to validate the external training and out-of-sample prediction. Participants are asked to circle 35 bells among 280 distractors in this test.^45,48^

All participants gave written informed consent to participate in the conducted study, which was approved by the respective Institutional Review Boards.

### Disconnectome

Patients’ diffusion-weighted data were not required for the disconnection analyses. Instead, white matter disconnection maps were derived from a normative diffusion-weighted dataset composed of *n* = 176 healthy participants, 45% males with 7 T MRI diffusion-weighted scans (HCP). Whole-brain tractography was reconstructed using the same procedure reported in Thiebaut de Schotten *et al.*^21^ Briefly, the default HCP preprocessing pipeline (v3.19.0),^49^ including TOPUP and EDDY corrections (https://fsl.fmrib.ox.ac.uk), was applied to the participants’ diffusion-weighted images, selecting the 65 volumes of uniformly distributed gradient directions with *b-*value = 2000 s/mm^2^ (1.05 mm isotropic voxel, 131 near-axial slices, acceleration factor of 3, echo time = 71.2 ms, relaxation time = 7000 ms and phase encoding direction paired anterior–posterior and reverse). Deterministic tractography was performed in the native diffusion MRI space using the software StartTrack (Version ST_20170905, https://www.mr-startrack.com). For the damped Richardson–Lucy algorithm,^50^ a fixed fibre response factor of *α* = 1.5 × 10–3 mm^2^/s was set with a geometric damping parameter of 8 and 200 interactions. For the spherical fibre orientation distribution, an absolute threshold of three times a grey matter isotropic voxel was set, and a relative threshold of 8% of its maximum amplitude.^51^ Whole-brain tractography streamline propagation was performed with a modified Euler algorithm (angle threshold of 35°, step size of 0.5 mm and minimum streamline length of 15 mm). Subsequently, first the subject tractography was converted into streamline density volumes where the intensities corresponded to the number of streamlines crossing each voxel. Second, a study-specific template of streamline density volumes was generated using the Greedy symmetric diffeomorphic normalization (GreedySyN) pipeline distributed with the Advanced Normalization Tools (ANTs) library.^52^ The template was then co-registered to the MNI152 space (2 mm resolution). Third, individual streamline density volumes were registered to the streamline density template in the MNI152 space and the same transformation was applied to the individual whole-brain streamline tractography using the trackmath tool distributed with the software package Tract Querier^53^ using ANTs GreedySyN. This step produced whole-brain streamline tractographies in the standard MNI152 space.

Stroke lesions were manually delineated in MRI scans and subsequently normalized to the MNI152 space (2 mm resolution) using the enantiomorphic normalization tool in the BCBtoolkit (http://toolkit.bcblab.com). Accordingly, before registering the patient’s T_1_-weighted image to the MNI152, the lesioned areas were replaced with the contralateral healthy tissues to calculate the normalization transformation.^54^ Then, disconnectome profiles were processed with the BCBtoolkit.^55^ HCP tractography was filtered considering only streamlines passing through each stroke lesion. To obtain a normative population group statistic, every filtered tractography was binarized. Thus, if at least one streamline passed in a voxel, the voxel value would be one. This step allowed the creation of a summarising percentage (%) map. Namely, for each stroke patient, a map ranging from 0 to 1 was obtained according to the number of HCP participants who would have reported a streamline disconnection in that voxel.

### The disconnectome morphospace

Dimensionality reduction of patients’ disconnectome was obtained using the Uniform Manifold Approximation and Projection (UMAP) method,^56^ a non-linear embedding method that distributes data variability along major axes. Specifically, UMAP projects data into a newly constructed manifold while preserving the original pairwise distance between the input data structure over the global distance. The UMAP manifold obtained will follow the theoretical framework of Riemannian geometry and algebraic topology. Accordingly, patients with a similar disconnection profile cluster together in the UMAP morphospace and patients with different disconnection profiles are located further apart. For dataset 1 the three-dimensional disconnectome maps were vectorized and imported as features of the embedding methods. UMAP parameters were set to default parameters (i.e. an approximation of 15 neighbours and a minimum 0.1 Euclidean distance to obtain a two-dimensional embedding of dataset 1). A two-latent variable configuration was preferred to provide a more intuitive space facilitating clinically meaningful interpretation, a space locally connected as Riemannian manifold that we addressed in the paper as the disconnectome morphospace.^56^ The UMAP embedding transformation was stored as a Python object, using the Pickle library, to apply the same low-dimensional transformation when new patients are imported into the model. Subsequently, coordinate scales were shifted to only have positive coordinates with zero as origin.

### The Disconnectome Symptom Discoverer

Statistical correlations between patient localization in the disconnectome morphospace and neuropsychological scores were conducted. Before the multiple regression formula, UMAP coordinates were converted into a 2D nifti image (260 × 260 matrix, 0.05 mm pixel size), and a Gaussian kernel spatial smoothing of 1 mm was applied (using FSL libraries https://fsl.fmrib.ox.ac.uk/fsl/fslwiki/). We used a high resolution (0.05 mm) to avoid an overlap between embedded coordinates; subsequently, a spatial smoothing of 20 pixels (i.e. Gaussian kernel sigma 1 mm) was chosen to ensure coverage of the whole morphospace. This step was conducted to model for the uncertainty of UMAP coordinates and to obtain a spatial distribution of patient localization in the disconnectome morphospace. Pixel-wise Pearson correlations between the patient probability of localization and neuropsychological scores were conducted with iterative loops in Python (python numpy.corrcoef). Medium effect size correlation results only were considered informative (*R* > |0.2|). Subsequently, because multiple clusters of voxels survived the threshold, a principal component analysis (PCA) was run to compress the patient coordinate distribution variability. Three main principal components have been considered (Python sklearn.decomposition.PCA). The number of principal components was chosen considering the amount of variance explained (always higher than 80%) and the number of morphospace clusters surviving the |*R*| > 0.2 threshold that included patients’ UMAP projection data-points (no more than three). Subsequently, patients’ principal components have been entered, as dependent variables, in the multiple regression model (Python sklearn.linear_model.LinearRegression) to predict neuropsychological scores:


(1) $$score_{1-year\;prediction}=c+\;\sum_{i=1}^{3}\;w_{i\;}PCA_{\;patient\;score\;}$$

where *c* is the intercept of the linear regression model, *w*_*i*_ are the model weights and *PCA*_*patient score*_ the model variables obtained as the inner product between the patient distribution of localization and PCA components.

The multiple regression formula was trained with dataset 2-training disconnectomes and validated using the out-of-sample dataset 2-validation and dataset 3 patients, and independently trained with dataset 4 and tested with dataset 5.

Accuracy of prediction was assessed as the mean absolute error (MAE)^57^ normalized by the maximum score obtained in the neuropsychological evaluation (MAE %):


(2) $$MAE\;\text{\%}=\;\frac{\sum_{i=1}^{N}\;|measured-predicted|/N}{max(score)}\;$$

Such normalization allows the comparison of prediction accuracy across different clinical scales, and it offers an intuitive interpretation for error measures of individual scores—the per cent of error (MAE %).

To assess how well our model, the DSD, fits group observations, we report the goodness of fit of predictions *R*^2^. Such a measure was calculated only when the number of subjects included was more than 20. Thus, *R*^2^ is provided for all the datasets except dataset 2-validation, where individual MAE % measures are reported.

### Disconnectome Symptom Discoverer comparison with lesion and functional connectivity models

We statistically compared the DSD’s *R*^2^ with six other commonly used prediction models. These models included (i) the disconnectome voxel-based approach (D-VB)^46^; (ii) the symptom discoverer (SD) embedding of the lesion data^21^ (L-SD); (iii) the lesion voxel-based symptoms mapping (L-VB)^4,58^; (iv) the functional disconnectome voxel-based symptoms mapping (f-VB); (v) the lesion volume and age SD (VolAge-SD); and (vi) the mean of the group. The D-VB approach allows the comparison of the DSD prediction power to the classic disconnection voxel-based approaches. The UMAP embedding of the lesion data (L-SD) is the same sophisticated framework as the DSD applied directly to the patients’ lesion data, to test the DSD added predictive value of disconnection to lesion topology in a comparable framework. L-VB allows the comparison of the DSD prediction power to the classic lesion approaches. The fourth model exploits functional dysconnectivity maps in a voxel-based association with clinical symptoms. The fifth model compares the DSD to predictions that consider two factors commonly argued as a primary determinant for recovery after stroke (i.e. lesion size and age)^32,59^ and the sixth model simply considers the mean of the neuropsychological scores.

Functional disconnectivity maps were analysed following the procedure of Boes and colleagues.^60^ We processed the same HCP cohort (*n* = 176, 7 T) to estimate the synchronous activity of the lesioned area and the rest of the brain. Subsequently, similarly to the disconnectome maps procedure (see the ‘Disconnectome’ section), an HCP group average was calculated in the MNI space to produce functional disconnection maps, in which each voxel ranged between −1 and 1. Negative and positive values correspond to the Pearson correlation between the lesion and other areas of the brain.^46^

For the SD methods (DSD, L-SD and VolAge-SD), for which multiple regressions used a training set, a *k*-fold validation was assessed by randomly assigning 70% of the Washington St. Louis cohort (dataset 2) as the training set in each iteration (*n* = 100). For the voxel-based (VB) approaches (D-VB, L-VB, f-VB) we ran 5000 permutations and estimated the goodness of fit of the model (*R*^2^) for the most significant voxels (98th percentile). This code is openly available in a previous publication of our group^21^: https://github.com/chrisfoulon/BCBlib/blob/devel/bcblib/scripts/effectsize_T2R.py. VB correlations included all the Washington St. Louis patients (dataset 2). Finally, we compared the DSD *R*^2^ results to the other methods using a two-tailed paired *t*-test and applying the Bonferroni correction for multiple comparisons.

### Disconnectome studio web application

The DSD web application (http://disconnectomestudio.bcblab.com) was built using the Django framework (https://www.djangoproject.com). This web framework allows database manipulation and is Python-based. The DSD front end was created with standard Javascript and css templates, whereas the backend is hosted in a DigitalOcean web server (https://www.digitalocean.com). Gunicorn and Ngnix are used for the web application live production.

### The Neuropsychological White Matter Atlas

To create a white matter atlas of the evaluated neuropsychological assessments, white matter disconnectomes (dataset 1) were correlated with patients’ PCA scores, evaluated by running the prediction model on dataset 1. The disconnectome data were used in defining the UMAP space, whereas the DSD model weights as variables of the multiple regression model to predict long-term neuropsychological symptoms. Using *randomise* (FSL libraries) a generalized voxel-based linear regression model was run, with disconnectome maps as independent variables and PCA scores as dependent variables. To address the result of replicability this procedure was repeated twice, splitting the dataset 1 into two halves of *n* = 666 subjects each.

The *randomise* T-maps obtained were used to calculate the correspondent effect size maps (*f*^2^, python code available in the open data section at http://www.bcblab.com). For each neuropsychological score, the three PCA scores, obtained from the DSD model, were evaluated and the maximum effect size across the components was considered. Subsequently, the highest effect size across neuropsychological assessments was reported in the NWMA summary map (FSL libraries *find_the_biggest* function). The replicability of the NWMA was quantified by means of Pearson correlations between the two dataset 1 summary maps.

### Data availability

All neuropsychological score maps used for defining the white matter atlas of neuropsychological components are freely available at https://neurovault.org/collections/11260/. The raw dataset imported in the BCBtoolkit software to calculate individual patient disconnectomes is available at https://www.humanconnectome.org (7 T diffusion data). In addition, processed data are available on request to the corresponding author or directly at https://osf.io/5zqwg/. The code used in the analyses is available as part of the BCBtoolkit package http://toolkit.bcblab.com and the DSD web application http://disconnectomestudio.bcblab.com. Any additional information is available upon request to L.T. and M.T.S.

## Results

### The disconnectome morphospace

The latent disconnectome configuration was defined based on *n* = 1333 stroke patients^43^ (see dataset 1 in Table 1) because its numerosity of >1000 stroke patients allowed for an ecological description of lesion variability for clinical translation.

Dataset 1 stroke lesions were processed to obtain disconnectome maps. Disconnectome maps quantify the pattern of white matter connections interrupted by each lesion based on the high-resolution tractography of a healthy population.^21,61,62^ Subsequently, the UMAP^56^ method was used to embed the disconnection complexity. A latent two-dimensional configuration of the disconnectome maps was obtained. Subsequently, external patient cohorts were imported into the dataset 1 latent configuration to train (datasets 2-training and 4) and test (datasets 2-validation, 3 and 5) the morphospace ability in predicting neuropsychological performance 1 year after the stroke.

**Figure 2:**
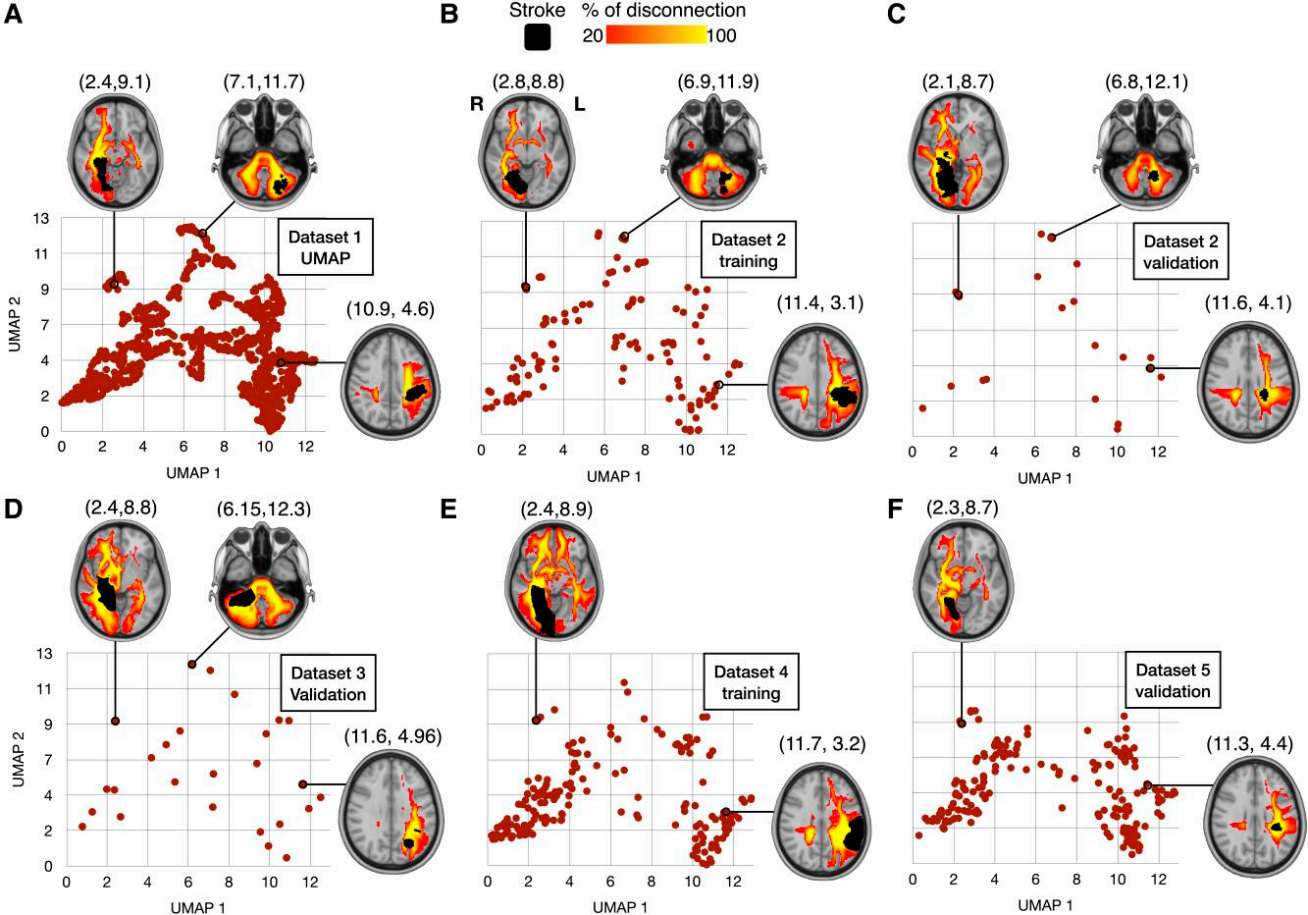
**Disconnectome morphospace**. Embedding of *n* = 1333 stroke disconnectomes (dataset 1) using the UMAP method (**A**). External cohort projections in the disconnectome morphospace for dataset 2-training (**B**) and dataset 2-validation (**C**); dataset 3-validation (**D**), dataset 4-training (**E**) and dataset 5-validation (**F**).

Patient disconnectome profiles were distributed based on cortical lesion location and commonly disconnected white matter tracts. Patients with major left or right hemisphere disconnections were embedded in the right and left half of the morphospace, respectively. Similarly, patients with posterior or anterior disconnections were localized at the top or the bottom of the embedded space. For instance, patients with a prominent disconnection of the right inferior fronto-occipital fasciculus (IFOF) were located at the top left extremity of the morphospace. In contrast, left corticospinal tract (CST) and arcuate fasciculus (AF) disconnections were located relatively more central and toward the bottom right side of the morphospace (Fig. 2). Importation of the different datasets showed consistency in the distribution of the disconnectivity pattern. These results demonstrate that the morphospace appropriately segregated the different profiles of disconnections (see Supplementary Fig. 3 for more details).^33,63^

### The composite morphospace

**Figure 3:**
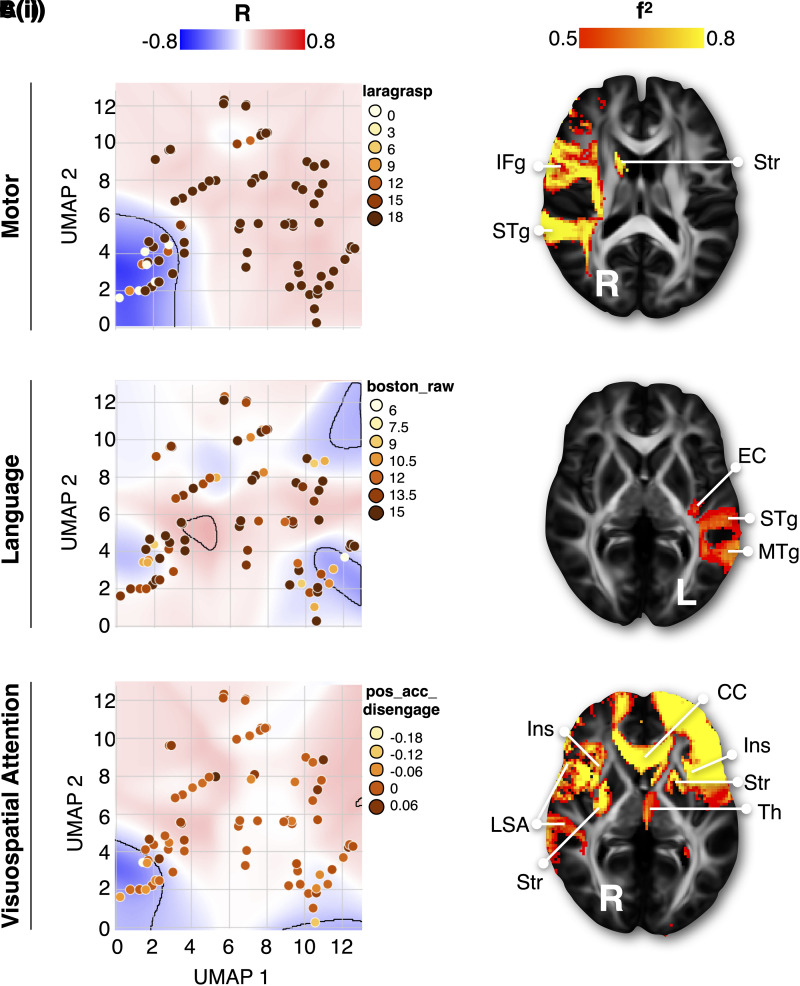
**Composite morphospace.** The composite morphospace corresponds to the disconnectome morphospace statistically combined with individual neuropsychological scores, **A**(**i**)–**C**(**i**) are three examples of different neuropsychological score associations with morphospace territories presented together with [**A**(**ii**)–**C**(**ii**)] their prototypical disconnection profile. In the morphospace background, Pearson correlations (*R*) with neuropsychological scores are shown location-wise; medium effect size territories (|*R* > 0.2|) have been delineated. All neuropsychological assessments and maps are reported in the Supplementary material (Section C). *f*^2^ = effect size; laragrasp = left grasping Action Research Arm test test; boston_raw = Boston naming test; pos_acc_disengage = accuracy in the Posner orienting task; CC = corpus callosum; EC = external/extreme capsule; IFg = inferior frontal gyrus; Ins = insula; LSA = long segment of the arcuate fasciculus; MTg = middle temporal gyrus; STg = superior temporal gyrus; Str = striatum; Th = thalamus.

The extent to which the disconnectome morphospace can predict different neuropsychological performances is currently unknown. To answer this question, we took advantage of the second independent dataset of stroke patients^25^ (*n* = 119; see dataset 2 in Table 1) who were extensively tested with standard neuropsychological assessments (*n* = 86, Supplementary Table 1). For each patient of the second dataset, disconnectome maps were calculated and imported into the disconnectome morphospace using the UMAP-defined transformation. To tackle embedding uncertainty, patient coordinates were spatially smoothed in the morphospace (see the ‘Materials and methods’ section). In so doing, each patient’s coordinates in the disconnectome morphospace were converted into probabilities of localization. A Pearson correlation approach was then used to estimate the association between each morphospace coordinate and neuropsychological performance (see Supplementary Fig. 1 for more details). Figure 3 indicates that a medium to large effect size association (all *|R|* > 0.2) existed between territories in the disconnectome morphospace and neuropsychological scores [Fig. 3A(i)–C(i)]. Importantly, for some scores, multiple clusters in the disconnectome morphospace, corresponding to different disconnection profiles, apparently led to the same neuropsychological impairment. This confirmed that no one-to-one relationship exists between lesioned structures and clinical disorders, and likewise, different brain damage locations can lead to the same functional impairment. We did not perform a simple linear association between the morphospace coordinate scale and neuropsychological scores. However, to extensively capture data variance in the morphospace, patients’ probability of localization within the significant clusters was modelled by a PCA (later referred to as spatial PCA). For each patient, the first three components of the spatial PCA were entered into a multiple regression analysis to predict single-patient neuropsychological scores 1 year after symptom onset. The multiple regressions created equations, modelling the relationship between each patient’s potential localization in the disconnectome morphospace (i.e. as defined by the first three components of the spatial PCA) and their neuropsychological scores. In so doing, we obtained a composite morphospace that takes advantage of the joint strengths of the two datasets. The composite morphospace accurately (with a medium to large effect size) and reliably predicted 70 of 86 neuropsychological scores ( Supplementary Table 2).

### Disconnection Symptoms Discovery web application

To make this resource and method available to the clinical-research community, we deployed an interactive web application platform, DSD (http://disconnectomestudio.bcblab.com). The DSD platform requires the input of disconnection maps. The DSD tool returns the expected 1-year neuropsychological scores for individual disconnectome maps (see the user guide in the Supplementary material, Section E). The prediction model relies on the databases presented in this study and can be updated on-demand with new neuropsychological assessments and patients’ disconnectomes.

### Disconnectome morphospace component mapping

**Figure 4:**
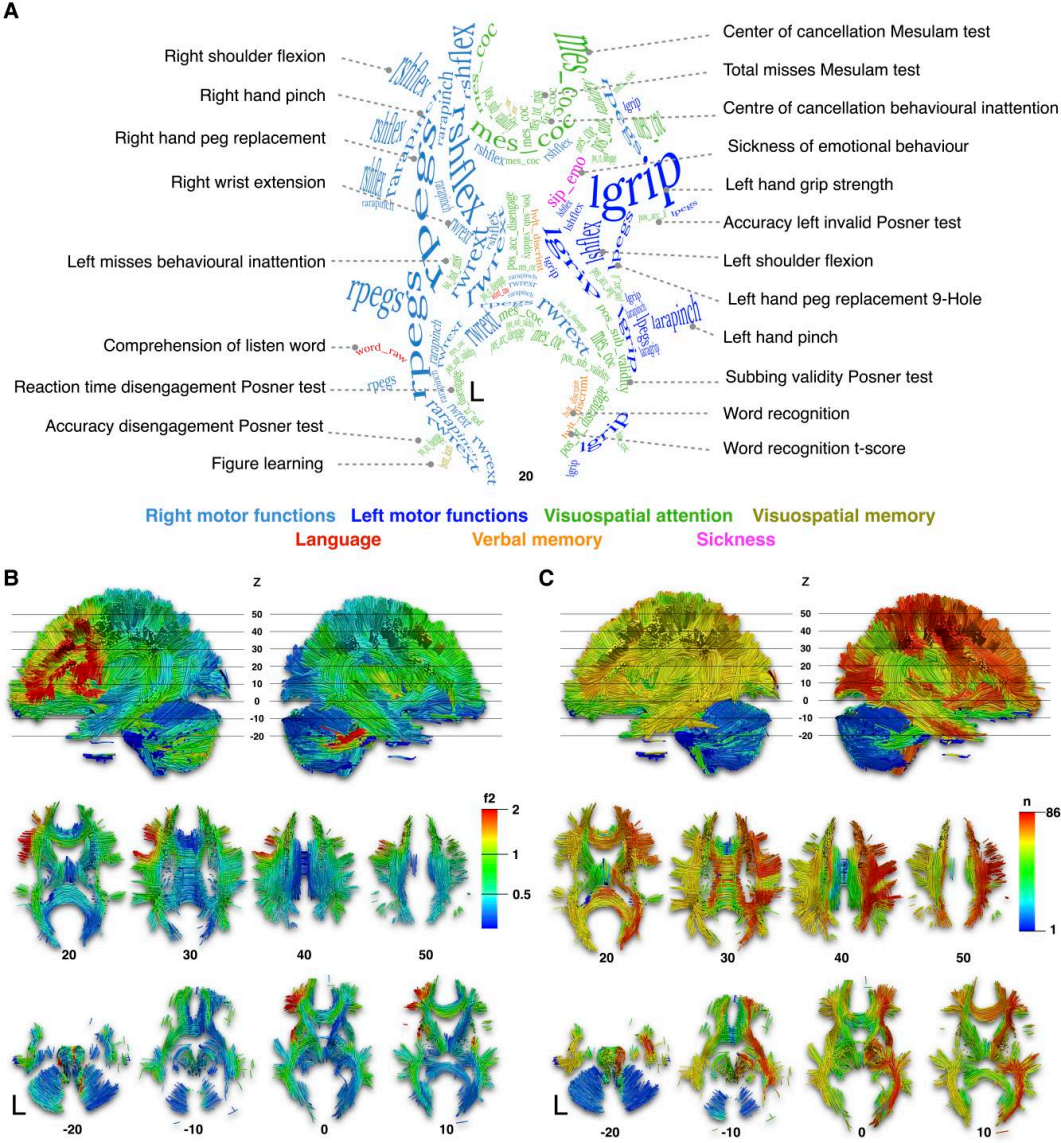
**NWMA**. (**A**) The axial projection of the labelling of neuropsychological scores corresponds to the strongest white matter associations. Visit https://neurovault.org/collections/11260/ or Supplementary material, Section C to view individual neuropsychological white matter maps. The labelling text font size and curvature reported were manually set to suit the size of the significant clusters and to follow the orientation of the white matter (see Supplementary material, Section D for high-resolution images). (**B**) The colour map corresponds to the highest effect size score (*f*^2^) across neuropsychological scores. (**C**) The colour map corresponds to the number (*n*) of neuropsychological scores overlapping their effect size map distributions. Such overlap will be addressed as versatility maps. MNI152 reference Z coordinates are reported below each axial slice.

In the next level, we brought the score prediction results back to the neuroimaging space to explore the neuroanatomical patterns leading to symptoms. The first dataset was split in half (2 × 666 disconnectome maps) to assess reproducibility. Latent patterns of predicted neuropsychological performances were statistically associated with brain disconnection maps of the two halves of the first dataset using voxel-wise linear regressions. In doing so we obtained two sets of maps of brain disconnection for each neuropsychological score [see example in Fig. 3A(ii)–C(ii) and all maps together with their full discussion in Supplementary material, Section C]. We were able to produce a comprehensive atlas of the brain disconnections associated with neuropsychological scores and the statistical comparison of the two sets of maps indicated an excellent level of reproducibility (Pearson *R* = 0.82). Figure 4A summarizes the highest statistical associations spanning from medium (0.15 > *f*^2^> 0.35) to high effect size (0.35 > *f*^2^), intending to provide a white matter atlas framing the novel NWMA (https://neurovault.org/collections/11260/). The highest effect sizes were in the left hemisphere, particularly in the frontal lobe connections, indicating the strongest associations between these disconnections and neuropsychological scores (Fig. 4B). Some areas can also be associated with multiple neuropsychological scores. To summarize this information, we calculated a versatility map that indicates how many neuropsychological scores can be predicted with a large effect size per volume unit of white matter (Fig. 4C). The versatility maps revealed a clear asymmetry between the left and the right hemispheres. This lower effect size and higher versatility in the right hemisphere suggest that more work is required to finely measure and dissociate right hemisphere functions in neuropsychology.

### External cohorts model validation

**Figure 5:**
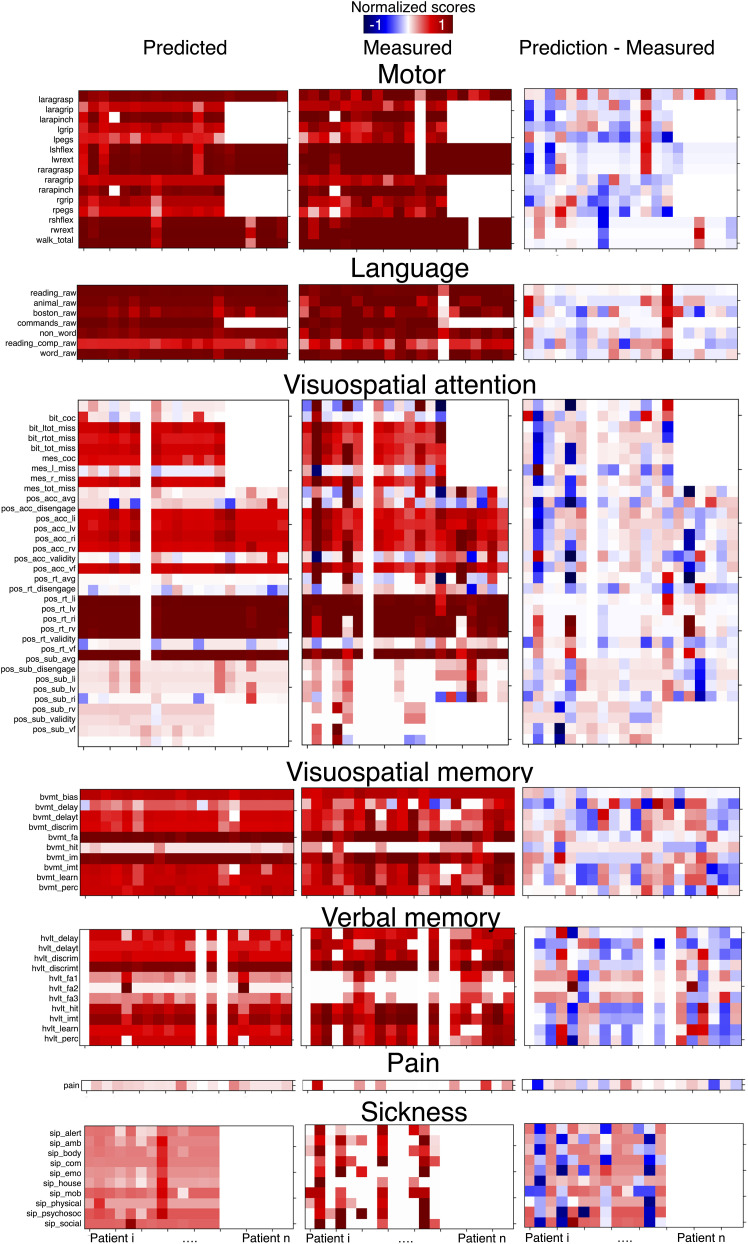
**Accuracy of the neuropsychological scores.** *Left*: Predicted neuropsychological scores, according to the composite disconnectome morphospace modelling. *Middle*: Measured neuropsychological scores 1 year after the stroke onset. *Right*: Normalized error as the difference between predicted and measured scores. Columns correspond to single patients’ neuropsychological profiles. Rows correspond to different neuropsychological scores. Scores were normalized with respect to the maximum scale score for visual purposes. See Supplementary Fig. 4 for the same figure derived from the training set. Full names of the abbreviations used for the scores are available in Supplementary Table 1 and at http://disconnectomestudio.bcblab.com.

To assess the accuracy of the predictions, data derived from a third independent dataset^25^ (20 stroke patients withheld from the original dataset 2; see dataset 2-validation in Table 1) were projected into the morphospace. From there, equations derived from the composite morphospace were applied to predict individual neuropsychological scores. Prediction accuracy of individual neuropsychological scores was assessed using the MAE, which reflects the difference between the observed and predicted scores normalized by the maximum score (MAE %; Fig. 5). The MAE is a standard metric for assessing machine learning accuracy (https://scikit-learn.org/stable/modules/model_evaluation.html) and it provides a clinically meaningful measure.^57,64^ Individual scores were predicted with an average MAE of 16.1 ± 7 (range 4.4, 39.2) %. More than three-quarters (*n* = 65) of all scores available (*n* = 83) were predicted in this third independent dataset with an MAE < 20% (see Supplementary Table 3 for comprehensive statistics).

From a personalized clinical perspective, the neuropsychological profiles of these 20 patients were assessed by comparing the measured and predicted scores for each patient. Figure 5 displays a colour map of the predicted and measured scores for dataset 2-validation.

Additionally, Fig. 6 illustrates three representative patients derived from dataset 2-validation. The radar plots demonstrate the correspondence of the DSD model prediction with the measured scores. The DSD prediction agreement with the patient’s recorded performances has been reported and discussed in the Supplementary material, Section F (Supplementary Figs 58–77).

**Figure 6:**
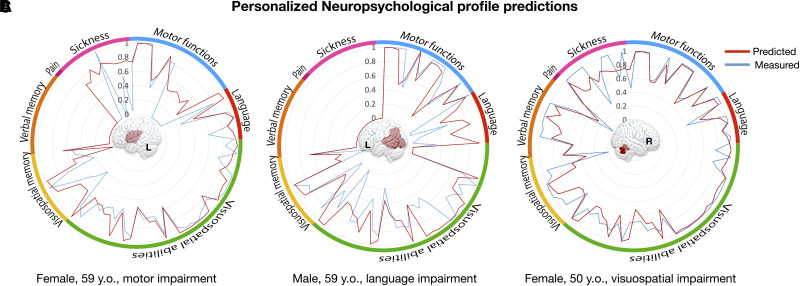
**Personalized neuropsychological profile predictions.** Predictions of neuropsychological profiles are reported for three representative patients from dataset 2-validation. For each profile, the outside ring indicates the correspondence with neuropsychological domains. Note that the polarity of some scores was inverted for readability so that higher scores always indicate better performance. **A**–**C** illustrate patients with a left inferior frontal lesion associated with chronic motor impairment (**A**); a left temporoparietal lesion suffering from chronic language impairment (**B**); and a right cerebellar stroke with chronic deficits in visuospatial and verbal memory processes (**C**). All radar plots from dataset 2-validation are reported in Supplementary Figs 58–77.

**Figure 7:**
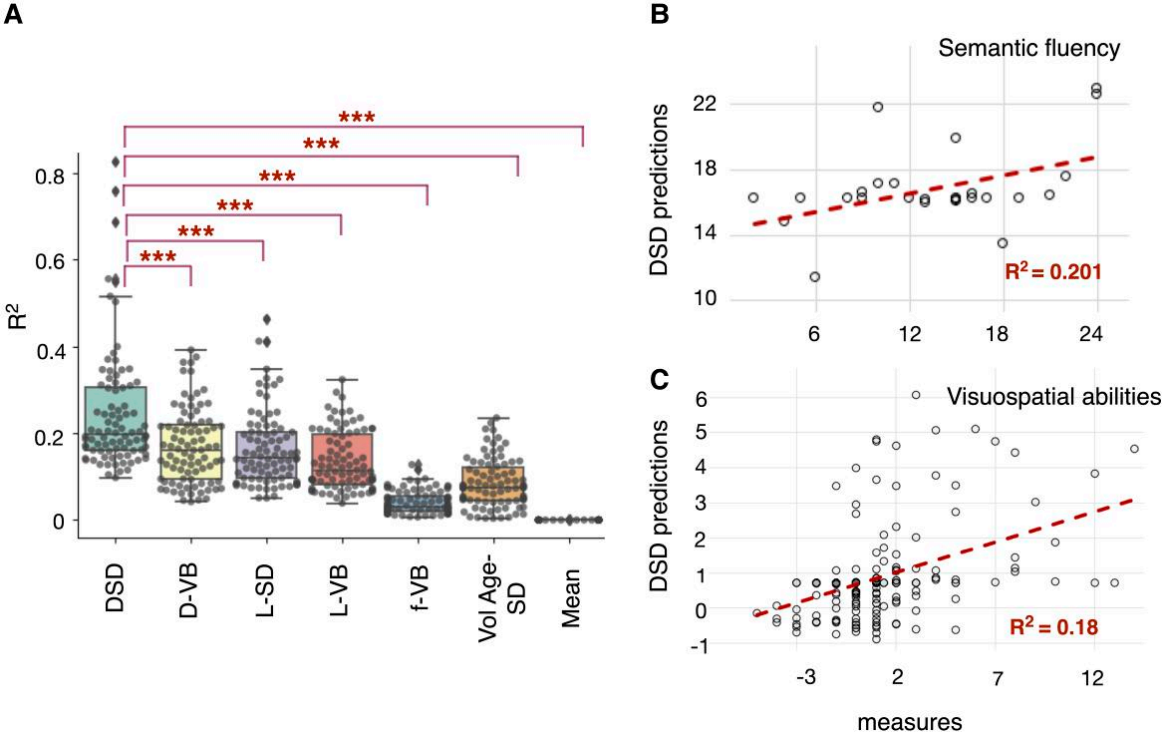
**DSD *R*^2^ and comparison with other predictive models.** (**A**) The box plot reports all the *R*^2^ across the neuropsychological scores (dataset 2) by regressing measured and predicted scores as obtained from the DSD compared with six other models: the disconnectome voxel-based (VB) correlations (D-VB), the symptom discoverer (SD) UMAP embedding of the lesion data (L-SD), the lesion VB mapping (L-VB), the functional dysconnectivity VB (f-VB), the SD with variables lesion volume and age (VolAge-SD) and the mean of the group. The box shows the quartiles, and the whiskers extend to the rest of the distribution, excluding the interquartile range outliers. Inside the boxes, the line indicates the median *R*^2^: *R*^2^ = 0.20 for the DSD, *R*^2^ = 0.16 for the D-VB, *R*^2^ = 0.14 for the L-SD, *R*^2^ = 0.11 for L-VB, *R*^2^ = 0.03 for f-VB, *R*^2^ = 0.07 for lesion size and age as predictors and *R*^2^ = 0 for the group mean. The asterisks correspond to the *P*-value obtained from a paired *t*-test (2-tails, Bonferroni corrected for multiple comparisons): ***P < 0.001. (**B**) DSD out-of-sample predictions for the semantic fluency test, animal category, *n* = 26 stroke patients (dataset 3). (**C**) DSD replication and out-of-sample predictions for visuospatial abilities, Bell’s score, *n* = 193 stroke patients (dataset 5).

The DSD model was further tested in a third external cohort—dataset 3 (see Table 1 for demographics) for semantic fluency (animals) scores, the only test comparable across their different neuropsychological routine assessments. The DSD model predictions for dataset 3 achieved *R*^2^ = 0.201 and MAE % of 13.71% (Fig. 7B).

Further, we demonstrated the DSD model generalizability by training and validating it in two different cohorts (datasets 4 and 5). We trained the DSD model for dataset 4, a French cohort of stroke patients comprising *n* = 190 participants, and we tested the trained DSD model on an external cohort, dataset 5 of *n* = 193 participants. For the Bells Test score predictions, in the training phase *R*^2^ = 0.2985 and MAE of 16% were achieved, in the external validation *R*^2^ = 0.1797 and MAE % of 13.69% (Fig. 7C).

### *R*^2^ comparison with other predictive models

The goodness of fit (*R*^2^) has been calculated by regressing measured and predicted scores to assess the neuropsychological variance explained by the proposed prediction model. In the model training phase (dataset 2-training), the average *R*^2^ achieved was 0.19 ± 0.09 (range 0.05, 0.67). See Supplementary Table 2 for the prediction *R*^2^ of each neuropsychological score. Moreover, to assess model stability, we randomly assigned patients in the DSD model training and validation, with a 100 permutation *k*-fold validation (all dataset 2-training and -validation). The training set *R*^2^ distribution across permutations is reported in Supplementary Fig. 4C. On average the median *R*^2^ achieved across iterations was 0.20 ± 0.14 (range 0.10, 0.83).

In Fig. 7C, the box plot of the individual *R*^2^ obtained for each model shows that the variance explained by the DSD model was significantly higher to all the six compared methods.

## Discussion

Applying state-of-the-art data-embedding methods, we succeeded in combining complementary databases of stroke patients. We produced a novel atlas of neuropsychological scores associated with brain disconnections—the NWMA. The proposed atlas is associated with an openly available web application, the DSD, which capitalizes on our methods and provides new anatomical insights into cognitive symptoms for researchers and clinicians. Out-of-sample validation of the DSD model (dataset 2-validation) accurately predicted 65 neuropsychological scores with a small prediction error below 20%.

Similar patterns of disconnections in our stroke cohorts were distributed closely in the embedding space, comparable to other research fields using the UMAP method for data clusterization purposes,^56^ e.g. single-cell genetic transcriptomes.^65,66^ Such embedded information allowed us to associate single-patient neuropsychological profiles at 1 year after a stroke with territories in the morphospace and patterns of disconnections. Therefore, the disconnectome morphospace acted as a reference to import and summarize new stroke disconnections.

By exploring white matter correlates systematically, we created a comprehensive atlas of the neuropsychological scores associated with brain disconnections. Our study evaluates single score measures. This rationale allowed us to discuss the similarities and differences in the white matter correlates of the individual scores and create a white matter neuropsychological atlas (NWMA; see Supplementary material, Section C). Additionally, in the context of external individual patients’ evaluation, single scores have the advantage of being comparable with other cohorts. Classical functional associations were confirmed, such as the lateralization of motor functions, the left perisylvian language network, the frontoparietal attentional networks or the right insula for sickness sensations. In addition, new insights on brain functioning and disconnection were reported, such as the callosal connectivity related to visual neglect, a cerebellar hub for visuospatial memory and the lingual gyrus for verbal memory (for individual results and discussion see Supplementary material, Section C). Of note, anatomical predictors of left and right motor dysfunctions were different. Widespread and bilateral white matter contribution including the left corticospinal tract was related to right motor dysfunctions 1 year after a stroke. However, long-term left motor dysfunctions were associated with the disconnection of right fronto-temporal and insular connections (Fig. 3 and Supplementary material, Section C.1). These findings agree with preliminary reports for overall motricity indices^47^ and suggest an asymmetrical neural bases for motor functions.^15,47,67–69^

Overall, NWMA’s greatest effect size in the left hemisphere highlights the left frontal lobe as a crucial hub for motor and language functions (Fig. 4B). On the contrary, the highest numerosity of function overlap on the right (Fig. 4C) is primarily due to pain and sickness NWMA maps extensively overlapping in the right frontoparietal and insular connections (Supplementary Figs 39–49). Only half of the visuospatial scores presented an exclusive right hemisphere involvement, e.g. the Mesulam cancellation test presented left lateralized or bilateral distributions (Supplementary Fig. 23).

The NWMA we are providing allows exploiting acute MRI scans to predict long-term stroke symptoms severity. These results indicate the suitability of the disconnectome model to predict a wide range of cognitive behavioural performances and identify a complete personalized, individual patient profile. This information will be a valuable resource in clinical settings, for example for the planning of personalized therapeutics and rehabilitation strategies. This is a step forward in comparison to many previous stroke AI methods that have a merely diagnostic purpose.^70^ The DSD model has a prognostic vocation based on cross-modal data (neuroimaging input–neuropsychological outcome prediction).

However, predictions were not equally accurate across functions (Supplementary Table 3). Three factors might explain these differences. First, some neuropsychological scores are more reliable than others in assessing performances.^71^ Second, plasticity and interindividual variability might interact with recovery.^20,32,72,73^ Third, the disconnectome model may not capture all the variance of brain injuries. Indeed, hypoperfusion^74^ and hypometabolism^75^ factors as well as acute imaging changes such as pseudonormalization^4^ are not currently accounted for. Moreover, we arbitrarily chose to create the disconnectome morphospace using two embedding dimensions for practical and intuitive purposes. While this proved to be a reliable way to describe the latent data structure and predict symptoms, future research might explore higher dimensionality of the morphospace (data and code available on demand from the authors). Finally, we used one of the largest and the most comprehensively explored stroke dataset available in terms of neuropsychological score. However, as the validation group represented about 20% of the training group, its modest numerosity limited the power of the validation step. In order to circumvent this potential limitation, the DSD web application allows for a wider validation with crowdsourcing and the addition of new datasets.

In Fig. 7A, we compared the DSD with six other models using different predictivity frameworks. The DSD UMAP model leveraging structural dysconnectivity outperformed lesion and functional dysconnectivity predictors. The statistical comparison was made across *n* = 86 neuropsychological scores recorded for dataset 2. The DSD achieved the highest *R*^2^ for motor scores (Supplementary Fig. 4C), in agreement with the structural dysconnectivity results recently reported by Bowren *et al*.^44^ In contrast, Bowren *et al*.^44^ indicated that functional connectivity was the best predictor for language scores. Our structural connectivity model, enriched with the UMAP method, outperformed f-VB results for language scores. This indicates that dimensionality reduction of white matter disconnections improves the variability estimation of the language network and its statistical association with language impairments. We further prove this point by predicting the semantic fluency (animals) score for the same Bowren *et al*.^44^ cohort; out-sample predictions achieved an *R*^2^ = 0.201 (Fig. 7B). Altogether, this evidence demonstrates that disconnectivity predictors may be an optimal strategy for neuropsychological scores 1 year after a stroke.

As a proof of concept, we tested the DSD model generalizability by training and testing in two entirely different cohorts (datasets 4 and 5). Figure 7C indicates that the DSD model external cohort predictions agreed with the actual measurements with a medium effect size (goodness of fit *R*^2^ = 0.18 for the Bell's score). Moreover, disconnectome and lesion data modelled with UMAP outperform voxel-based approaches (Fig. 7A). This result validates our initial observation, for which no one-to-one relationship between structures and clinical presentation is possible, but rather the integrated brain functioning necessitates high-order modelling for an accurate description.^26^

In the current work, we aimed to propose a novel framework that leverages MRI data to predict patients’ long-term clinical/cognitive scores. We obtained access to five independent stroke cohorts thanks to a highly collaborative research network. Dataset 1 included MRI and was acquired in >1000 patients and informed the UMAP method on the variability of white matter damage subsequent to a stroke. With dataset 2, we trained a regression model for predicting 86 neuropsychological scores. One of those, the semantic fluency test, was tested in an independent cohort, dataset 3. Datasets 4 and 5 served to train and test the same datasets 2 and 3 models for visuospatial abilities and demonstrated the proposed framework’s generalizability to external cohorts. The commonality of clinical assessment across centres dictated the specific score choices. However, to promote future model testing and collaborative research at the global level we developed the DSD web application, a free and user-friendly web browser tool that only requires an Internet connection and a lesion disconnection data. Instant software access and automatic updates make the world-wide web the ideal medium for clinical translations. The application of the DSD method can potentially help the assessment of personalized prognosis. The developed web application has been released to facilitate a broader model validation critical for future global validation testing the DSD reliability. Only after this indispensable validation step should the DSD web application be considered for clinical trial testing. Hence, the DSD aims to benefit the researchers’ understanding of brain functioning and patients’ treatments alike.

## Supplementary Material

awad013_Supplementary_Data
