# Supplementary material for: Latent disconnectome prediction of long-term cognitive-behavioural symptoms in stroke
Source: Brain. 2023 Mar 16;146(5):1963–78. doi: 10.1093/brain/awad013 (PMC10151183; doi:10.1093/brain/awad013)
Supplement: awad013_Supplementary_Data [file awad013_Supplementary_Data.zip › brain-2022-00965-File010.pdf]

Supplementary Materials

TABLE OF CONTENTS

A. Dataset information

Imaging acquisition parameters. p.2

Inclusion criteria. p.3

B. The Disconnectome Symptom Discoverer – DSD

Supplementary Figure 1: Graphical summary model workflow. p.4

Supplementary Figure 2: Stroke lesion variability maps. p.5

Supplementary Figure 3: Disconnectome morphospace. p.6

Supplementary Table 1: Neuropsychological score acronyms. p.7-9

Supplementary Table 2: PCA variance and multiple regression model. p.10-12

Supplementary Table 3: Accuracy of prediction single scores. p.12-14

Supplementary Figure 4: Prediction matrixes training set. p.15

A. Dataset information

Imaging acquisition parameters

Dataset 1. MRI was acquired at the University College London Hospitals in London, on GE Signa, Philips Achieva and Ingenia, Siemens Avanto, Skyra and Verio with either 1.5 T or 3 T. The MRI protocol included DWI with null b-value and b-value=1000 s/mm^2 and T2-weighted images, which were used to segment stroke lesions as explained in the original paper Xu et al.[1].

Dataset 2. MRI was acquired at the School of Medicine of the Washington University in St. Louis, on a Siemens 3T Tim-Trio scanner. The MRI acquisition protocol included: 1) structural imaging MP-RAGE T1-weighted, sagittal acquisition with repetition time = 1950 ms, echo time = 2.26 ms, flip angle = 9 deg, voxel size = 1.0 x 1.0 x 1.0 mm, slice thickness = 1.00 mm. 2) a transverse turbo spin-echo T2-weighted image with repetition time = 2500 ms, echo time = 435 ms, voxel-size = 1.0 x 1.0 x 1.0 mm, slice thickness = 1.00 mm. 3) a sagittal FLAIR (fluid attenuated inversion recovery) with repetition time = 7500 ms, echo time = 326 ms, voxel-size = 1.5 x 1.5 x 1.5 mm, slice thickness = 1.50 mm. Lesion segmentation was conducted considering these three sequence metacontrast data in acute stroke. For further details see Corbetta et al. [2].

Dataset 3. Neuroimaging scans from MRI and CT were co-registered using FSL. Lesions were manually traced on native T1-weighted MRI scans, for further details, see Browren et al. [3].

Dataset 4. DISCONEGLECT cohort (Disconnection and Unilateral Spatial Neglect, NCT05268159). the scanner was a 1.5 T MR Achieva dstream (Philips Healthcare, the Netherlands). Whole-brain anatomical scans were acquired in an axial plane parallel to the anterior commissure-posterior commissure consisting of a 2D SE T1-weighted images (30 contiguous slices; voxel size = 0.9x0.9x6 mm^3; TR/TE/TI/α: 5228/10/1100/70°); axial T2-weighted FLAIR images (24 slices; voxel size = 0.47x0.47x6 mm^3; TR/TE/TI/α: 9502/149/2800/90°); axial T2 FFE images (23 slices; voxel size =1 x1x6 mm3; TR/TE/TI/α: 946/32/2800/90°) ; and DWI (24 slices; voxel size = 1x1x6 mm^3; TR/TE/α: 2700/70/90°). Lesions were delineated manually in the native space on axial slices, based on the different available MRI sequences, by a trained examiner (EA) blind to the patient's performance. The T1-weighted scans and lesions were then normalised to the MNI-152 space (1mm resolution) using the "normalisation tool" of the BCBtoolkit software (http://toolkit.bcblab.com) [4] with an enantiomorphic approach [5]. T1-weighted images were registered to the MNI-152 template using affine and diffeomorphic deformations [6]. The quality of normalised T1-weighted and lesions was checked, and a custom mask based on the MNI-152 template removed voxels of lesion outside the white and grey matter brain tissue.

Dataset 5. The DOBRAS cohort (Determinants of Balance Recovery After Stroke; ClinicalTrial.gov/ NCT03203109). Neuroimaging was acquired on a clinical 1.5 T MR Magnetom Aera scanner (Siemens, Erhlangen, Germany). Whole-brain anatomical scans were acquired in an axial plane parallel to the anterior commissure-posterior commissure consisting of 3-D GRE T1-weighted images (160 contiguous slices; voxel size=0.9x0.9x0.9 mm^3; TR/TE/TI/α: 1900/3.67/1100 ms/15°; acquisition time=4.5 min); axial T2-weighted FLAIR images (30 slices; voxel size=0.7x0.7x4 mm^3; TR/TE/TI: 9000/74/2500 ms). Lesion volume, determined as the percentage of hemisphere encroached on by the lesion, was measured by using MRIcron after a manual lesion delineation from axial slices acquired with T2-FLAIR sequences. All drawings involved 2 trained operators, the first from a panel of three and the second always the same and with blinding to behavioural data. Any disagreements were resolved by consensus [7]. Lesion drawings were performed on axial slices of a T1-weighted MRI

template from the Montreal Neurological Institute (https://www.mcgill.ca/bic/neuroinformatics/brain-atlases-human) normalised to Talairach's space [7].

Inclusion criteria

Dataset 1. Patients were enrolled at the University College London Hospitals (UCLH) acute stroke service over the past decade. It was identified where the clinical diagnosis was acute ischaemic stroke, computed tomography (CT) and DWI had both been performed, and at least one definite acute ischaemic lesion could be identified on the DWI. CT is routinely performed on all patients suspected of acute stroke at UCLH; MRI on the majority, constrained mostly by contraindications and tolerability. To reduce bias from the global effects of very large lesions, only patients with DWI-quantified lesion volume of less than 50 mls were included: ~90th centile for our clinical population. The sample is therefore reasonably representative of acute stroke [1].

Dataset 2. For participants' enrolment, inclusion criteria were as follows: (i) age 18 or older, (ii) first symptomatic stroke, ischemic or hemorrhagic, (iii) clinical evidence of any neurological deficit based on an examination, and (iv) time of enrollment < 2 weeks post-stroke onset. Exclusion criteria were: (i) the inability to maintain wakefulness during testing, (ii) the presence of other neurological, psychiatric, or medical conditions that precluded active participation in research and/or may alter the interpretation of the behavioural/imaging studies (e.g., dementia, schizophrenia), or limited life expectancy to less than 1 y (e.g., cancer or congestive heart failure class IV), (iii) evidence of clinically significant periventricular white matter disease (equal or > grade 5 of Longstreth et al. [8]), and (iv) contraindications for MRI including claustrophobia or scanner-incompatible implants [9].

Dataset 3. For the Iowa cohort, patients were tested in the chronic phase, three months or more after lesion onset.

Dataset 4 and 5. In the DOBRAS and DISCONEGLECT cohorts, participants were assessed during routine care in the subacute phase after stroke (between 1 and 3 months). A study-independent clinical multidisciplinary team assessed each participant. Eligible individuals were informed about their inclusion in the study (orally or in writing) and were able to opt out. Participant recruitment lasted from January 2012 to May 2019 for the DOBRAS cohort and from June 2011 to December 2021 for the DISCONECGLECT cohort. Inclusion criteria were the same in both cohorts: adults (18-80 yo), a first-ever unilateral right or left hemispheric stroke (imaging confirmed), and an assessment by the Bells Test between 30 and 90 days after stroke onset. Exclusion criteria were recurrent stroke, history of significant complications at the acute stage, inability to perform the Bells Test due to severe language, cognitive or psychiatric disorder, and severe uncorrected loss of visual acuity.

B. The Disconnectome Symptoms Discoverer

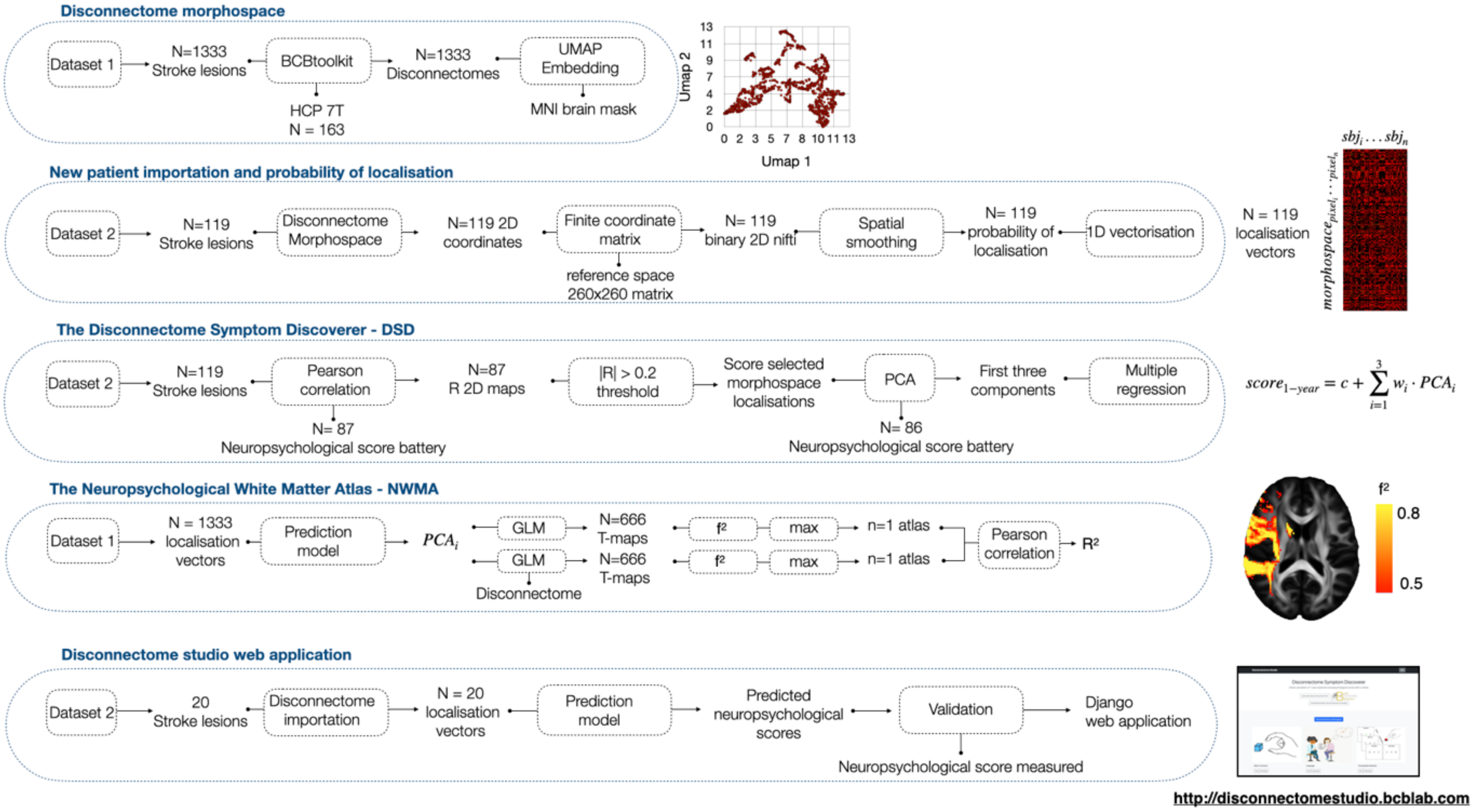

Supplementary Figure 1: Graphical summary of neuroimaging and statistical model analysis workflow.

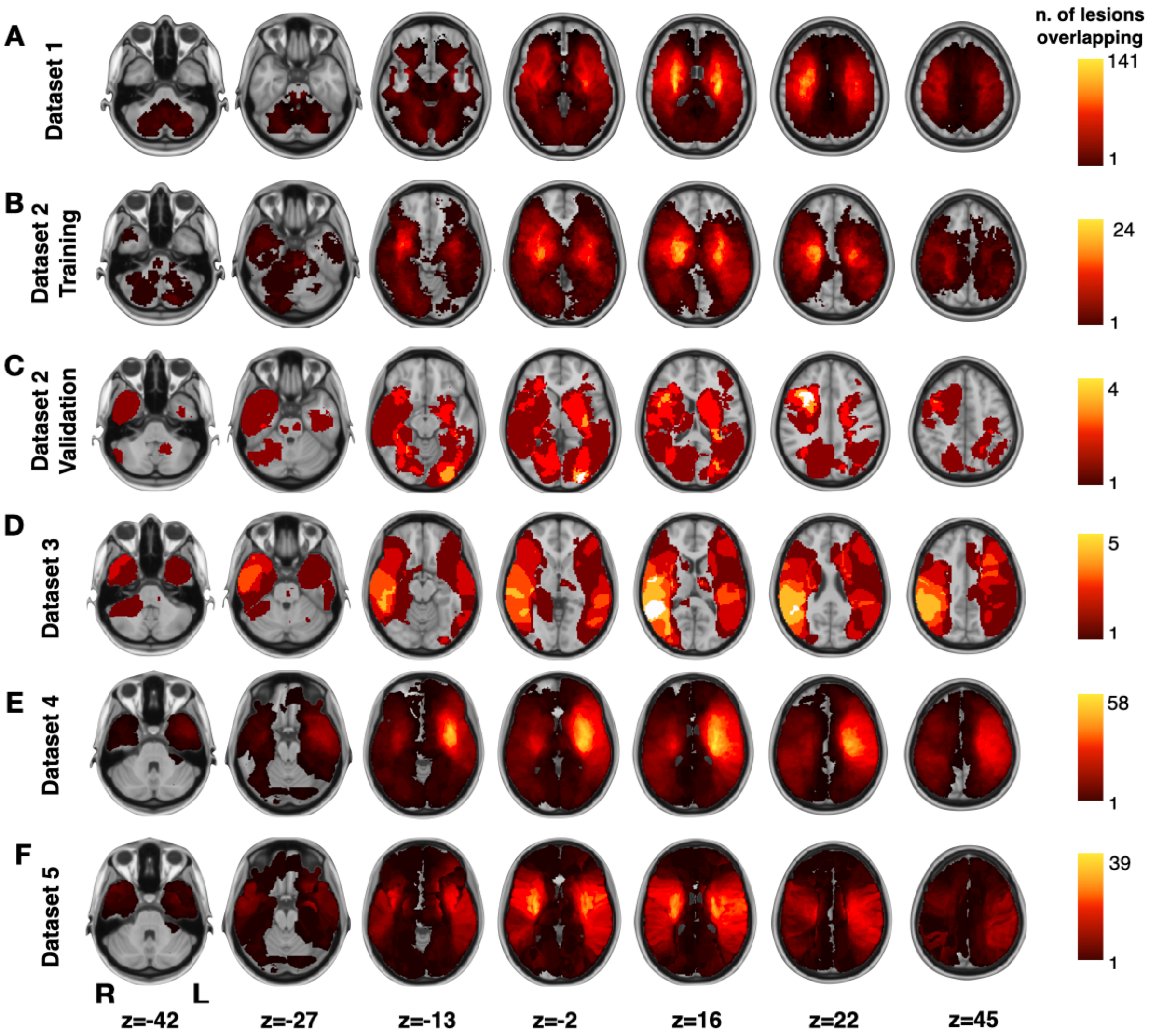

Supplementary Figure 2: Group-level lesion variability maps for dataset 1-UMAP (A), dataset 2-training (B), and dataset 2-validation (C), dataset 4-training (D), and dataset 5-validation (E). Colormaps show the number of overlapping lesions. Inferior-superior z-coordinates are reported according to the MNI152.

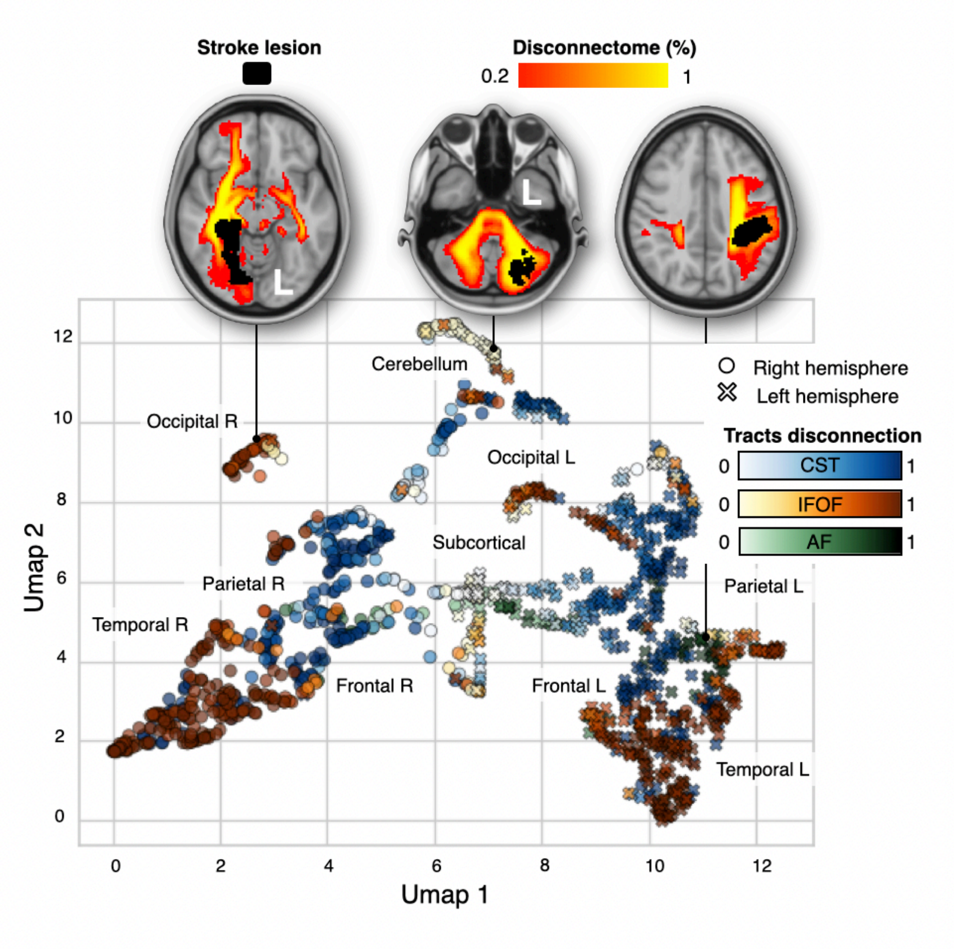

Supplementary Figure 3. Disconnectome morphospace. (*Top*) three patients' disconnectomes, where a red-yellow colour map indicates the probability of disconnection. (*Bottom*) the disconnectome morphospace, where patients are marked with a circle ("o") or with a cross ("x") when their disconnection involves dominantly the right (R) or the left (L) hemisphere, respectively. Colours in the morphospace indicate the probability of disconnection of three exemplary white matter tracts: the cortico-spinal tract (CST), the arcuate fasciculus (AF), and the inferior-fronto occipital fasciculus (IFOF). Brain lobe annotations indicate the dominant location of each stroke lesion.

Domain	Acronym	Neuropsychological score (test battery)
Motor	laragrasp	Left hand grasp (ARAT)
	laragrip	Left hand grip (ARAT)
	larapinch	Left hand pinch (ARAT)
	lgrip	Left hand grip strength
	lpegs	Left hand peg replacement
	lshflex	Left shoulder flexion (AROM)
	lwrext	Left wrist extension (AROM)
	raragrasp	Right hand grasp (ARAT)
	raragrip	Right hand grip (ARAT)
	rarapinch	Right hand pinch (ARAT)
	rgrip	Right hand grip strength
	rpegs	Right hand peg replacement
	rshflex	Right shoulder flexion (AROM)
	rwrext	Right wrist extension (AROM)
	walk_total	Walking combined score
Language	reading_raw	Sentence reading (BDAE)
	animal_raw	Animal name fluency (SVFT)
	boston_raw	Picture naming (BDAE)
	commands_raw	Performing listen commands (BDAE)
	nonword	Nonword repetition (BDAE)
	reading_comp_raw	Comprehension of read sentence (BDAE)
	word_raw	Comprehension of listen word (BDAE)
Visuospatial attention	bit_coc	Center of cancellation (BIT)
	bit_ltot_miss	Left misses (BIT)
	bit_rtot_miss	Right misses (BIT)
	bit_tot_miss	Total misses (BIT)
	mes_coc	Center of cancellation (Mes-USCT)
	mes_l_miss	Left misses (Mes-USCT)
	mes_r_miss	Right misses (Mes-USCT)
	mes_tot_miss	Total misses (Mes-USCT)
	pos_acc_avg	Accuracy average (Posner)
	pos_acc_disengage	Accuracy disengagement (Posner)

	pos_acc_li	Accuracy left invalid (Posner)
	pos_acc_lv	Accuracy left valid (Posner)
	pos_acc_ri	Accuracy right invalid (Posner)
	pos_acc_rv	Accuracy right valid (Posner)
	pos_acc_validity	Accuracy validity (Posner)
	pos_acc_vf	Accuracy visual effect (Posner)
	pos_rt_avg	Reaction time average (Posner)
	pos_rt_disengage	Reaction time disengagement (Posner)
	pos_rt_li	Reaction time left invalid (Posner)
	pos_rt_lv	Reaction time left valid (Posner)
	pos_rt_ri	Reaction time right invalid (Posner)
	pos_rt_rv	Reaction time right valid (Posner)
	pos_rt_validity	Reaction time validity (Posner)
	pos_rt_vf	Reaction time visual effect (Posner)
	pos_sub_avg	Subbing average (Posner)
	pos_sub_disengage	Subbing disengagement (Posner)
	pos_sub_li	Subbing left invalid (Posner)
	pos_sub_lv	Subbing left valid (Posner)
	pos_sub_ri	Subbing right invalid (Posner)
	pos_sub_rv	Subbing right valid (Posner)
	pos_sub_validity	Subbing validity (Posner)
	pos_sub_vf	Subbing visual effect (Posner)
Visuospatial memory	bvmt_bias	Figure bias (BVMT)
	bvmt_delay	Figure delayed recall (BVMT)
	bvmt_delayt	Figure delayed recall t-score (BVMT)
	bvmt_discrim	Figure recognition discrimination (BVMT)
	bvmt_fa	Figure false alarm (BVMT)
	bvmt_hit	Figure recognition hit (BVMT)
	bvmt_im	Figure immediate recall (BVMT)
	bvmt_imt	Figure immediate recall t-score (BVMT)
	bvmt_learn	Figure learning (BVMT)
	bvmt_perc	Figure percent retained (BVMT)
Verbal memory	hvlt_delay	Word recall (HVLT)

	hvlt_delayt	Word recall t-score (HVLT)
	hvlt_discrim	Word recognition (HVLT)
	hvlt_discrimt	Word recognition t-score (HVLT)
	hvlt_fa1	Word recognition related false alarms (HVLT)
	hvlt_fa2	Word recognition unrelated false alarms (HVLT)
	hvlt_fa3	Word recognition false positive (HVLT)
	hvlt_hit	Word recognition hits (HVLT)
	hvlt_imt	Word immediate recall t-score (HVLT)
	hvlt_learn	Word learning (HVLT)
	hvlt_perc	Word recall retained (HVLT)
Pain	pain	Pain
Sickness	sip_alert	Alertness behaviour (SA-SIP)
	sip_amb	Ambulation (SA-SIP)
	sip_body	Body care and movement (SA-SIP)
	sip_com	Communication (SA-SIP)
	sip_emo	Emotional behavior (SA-SIP)
	sip_house	Household (SA-SIP)
	sip_mob	Mobility (SA-SIP)
	sip_physical	Physical function (SA-SIP)
	sip_psychosoc	Psychosocial (SA-SIP)
	sip_social	Social (SA-SIP)

Supplementary Table 1: Neuropsychological score abbreviations. Among the adopted examination tests: ARAT=Action Reaction Arm total test, AROM= Active Range Of Motion, SVFT= Standard Verbal Fluency Test, BIT= Behavioural Inattention Test, BDAE=Boston Diagnostic Aphasia Examination, Mes-USCT=Mesulam Unstructured Symbol Cancellation Test, BVMT=Brief Visuospatial Memory Test-revised, HVLT= the Hopkins Verbal Learning Test-revised, SA-SIP=Stroke-Adapted Sickness Impact Profile.

Neuropsychological scores		PCA variance explain (%)			Multiple regression model					
Domain	Acronym	PCA 1	PCA 2	PCA 3	df	1-β	R^2	f^2	p-value	FDR
Motor	laragrasp	47	25	14	78	1.000	0.44	0.78	2.01E-09	5.75E-08
	laragrip	51	22	12	78	0.999	0.28	0.39	1.50E-05	1.43E-04
	larapinch	46	25	13	77	1.000	0.47	0.89	3.03E-10	1.30E-08
	lgrip	32	19	16	72	0.998	0.25	0.33	1.84E-04	1.05E-03
	lpegs	39	26	9	72	0.999	0.41	0.70	5.10E-08	1.10E-06
	lshflex	35	27	10	70	0.999	0.35	0.54	2.00E-06	3.44E-05
	lwrext	47	29	8	72	0.983	0.19	0.24	2.05E-03	5.04E-03
	raragrasp	47	29	12	78	0.999	0.27	0.37	2.70E-05	2.32E-04
	raragrip	46	30	12	78	0.999	0.31	0.44	5.00E-06	7.17E-05
	rarapinch	42	29	13	78	0.999	0.28	0.40	1.40E-05	1.43E-04
	rgrip	36	28	13	72	0.917	0.14	0.16	1.68E-02	2.12E-02
	rpegs	37	17	13	72	0.989	0.20	0.26	1.25E-03	3.83E-03
	rshflex	49	27	11	72	0.999	0.28	0.38	5.00E-05	3.73E-04
	rwrext	52	27	11	72	0.939	0.15	0.17	1.09E-02	1.56E-02
	walk_total	69	21	6	78	0.808	0.09	0.13	2.18E-03	5.04E-03
Language	reading_raw	65	21	9	76	0.977	0.17	0.21	3.04E-03	6.23E-03
	animal_raw	50	21	12	78	0.975	0.17	0.20	3.20E-03	6.41E-03
	boston_raw	69	12	10	78	0.886	0.12	0.13	2.64E-02	3.11E-02
	commands_raw	56	32	7	72	0.999	0.27	0.37	7.20E-05	4.73E-04
	nonword	67	13	7	76	0.996	0.22	0.28	4.01E-04	1.82E-03
	reading_comp_raw	61	27	8	76	0.841	0.10	0.12	4.31E-02	4.79E-02
	word_raw	48	28	9	78	0.967	0.16	0.19	4.58E-03	8.10E-03
Visuospatial attention	bit_coc	85	9	3	71	0.953	0.16	0.19	7.81E-03	1.27E-02
	bit_ltot_miss	73	17	6	71	0.995	0.23	0.30	4.46E-04	1.92E-03
	bit_rtot_miss	75	16	3	71	0.996	0.24	0.31	3.45E-04	1.71E-03
	bit_tot_miss*	85	8	4	71	0.998	0.26	0.35	2.24E-01*	2.27E-01*
	mes_coc	64	15	9	77	0.989	0.19	0.24	1.05E-03	3.48E-03
	mes_l_miss	69	14	10	77	0.981	0.18	0.22	2.27E-03	5.04E-03
	mes_r_miss	56	29	9	77	0.926	0.13	0.15	1.39E-02	1.86E-02
	mes_tot_miss	45	32	16	77	0.981	0.18	0.22	2.28E-03	5.04E-03
	pos_acc_avg	45	34	12	74	0.986	0.19	0.24	1.67E-03	4.62E-03
	pos_acc_disengage	48	24	12	74	0.995	0.22	0.28	4.92E-04	2.01E-03
	pos_acc_li*	57	21	14	74	0.797	0.10	0.11	6.30E-02*	6.77E-02*
	pos_acc_lv	53	20	15	74	0.999	0.30	0.44	1.00E-05	1.23E-04
	pos_acc_ri	57	25	9	74	0.987	0.19	0.24	1.56E-03	4.47E-03

	pos_acc_rv	58	21	8	74	0.998	0.24	0.32	3.57E-04	1.71E-03
	pos_acc_validity	46	18	9	74	0.911	0.13	0.15	1.85E-02	2.28E-02
	pos_acc_vf	54	19	14	74	0.930	0.14	0.16	1.32E-02	1.81E-02
	pos_rt_avg	59	16	13	74	0.913	0.13	0.15	1.81E-02	2.26E-02
	pos_rt_disengage	48	25	8	74	0.985	0.19	0.23	1.75E-03	4.70E-03
	pos_rt_li	60	17	9	74	0.919	0.13	0.15	1.64E-02	2.10E-02
	pos_rt_lv	56	22	12	74	0.949	0.15	0.18	8.48E-03	1.31E-02
	pos_rt_ri	39	26	14	74	0.939	0.14	0.17	1.07E-02	1.56E-02
	pos_rt_rv	59	17	9	74	0.996	0.23	0.29	3.57E-04	1.71E-03
	pos_rt_validity	48	18	16	74	0.981	0.18	0.22	2.41E-03	5.05E-03
	pos_rt_vf	43	24	10	74	0.990	0.20	0.25	1.04E-03	3.48E-03
	pos_sub_avg	70	14	6	68	0.962	0.17	0.21	6.03E-03	9.98E-03
	pos_sub_disengage	53	25	7	68	0.973	0.18	0.23	3.98E-03	7.45E-03
	pos_sub_li*	50	32	10	68	0.778	0.10	0.11	7.62E-02*	7.89E-02*
	pos_sub_lv	61	15	13	68	0.965	0.18	0.21	5.50E-03	9.46E-03
	pos_sub_ri	74	13	8	68	0.932	0.15	0.18	1.30E-02	1.81E-02
	pos_sub_rv	62	12	10	68	0.927	0.15	0.17	1.49E-02	1.94E-02
	pos_sub_validity	54	27	6	68	0.970	0.18	0.22	4.62E-03	8.10E-03
	pos_sub_vf	39	33	7	68	0.984	0.20	0.25	2.05E-03	5.04E-03
Visuospatial memory	bvmt_bias	32	18	14	78	0.998	0.25	0.33	7.70E-05	4.73E-04
	bvmt_delay	54	20	14	78	0.857	0.11	0.12	3.68E-02	4.22E-02
	bvmt_delayt	46	21	12	78	0.993	0.20	0.25	6.87E-04	2.57E-03
	bvmt_discrim	53	26	11	78	0.883	0.11	0.13	2.69E-02	3.13E-02
	bvmt_fa	49	24	18	78	0.892	0.12	0.13	2.39E-02	2.90E-02
	bvmt_hit	53	32	5	78	0.991	0.19	0.24	9.60E-04	3.44E-03
	bvmt_im	58	16	12	78	0.941	0.14	0.16	1.00E-02	1.48E-02
	bvmt_imt	53	23	10	78	0.960	0.15	0.18	5.94E-03	9.98E-03
	bvmt_learn*	91	5	4	78	0.740	0.08	0.09	9.59E-02*	9.81E-02*
	bvmt_perc	49	26	8	78	0.994	0.21	0.26	5.65E-04	2.21E-03
Verbal memory	hvlt_delay	86	10	2	77	0.886	0.12	0.13	2.60E-02	3.10E-02
	hvlt_delayt	67	20	7	78	0.839	0.10	0.11	4.34E-02	4.79E-02
	hvlt_discrim	54	18	13	77	0.969	0.16	0.19	4.32E-03	7.90E-03
	hvlt_discrimt	41	24	14	78	0.973	0.16	0.20	3.51E-03	6.71E-03
	hvlt_fa1	44	30	10	77	0.925	0.13	0.15	1.42E-02	1.87E-02
	hvlt_fa2	73	16	10	77	1.000	0.67	2.07	5.61E-18	4.82E-16
	hvlt_fa3	43	27	13	77	0.870	0.17	0.20	3.49E-03	6.71E-03

	hvlt_hit	73	10	7	77	0.779	0.09	0.10	7.24E-02*	7.60E-02*
	hvlt_imt	82	17	1	78	0.488	0.05	0.05	3.06E-01*	3.06E-01*
	hvlt_learn	80	10	4	77	0.837	0.10	0.11	4.46E-02	4.86E-02
	hvlt_perc	43	34	17	77	0.980	0.18	0.21	2.35E-03	5.05E-03
Pain	pain	43	24	17	67	0.999	0.29	0.42	5.20E-05	3.73E-04
Sickness	sip_alert	50	26	9	72	0.787	0.10	0.11	6.92E-02*	7.35E-02*
	sip_amb	51	25	8	72	0.951	0.16	0.18	8.24E-03	1.31E-02
	sip_body	53	24	10	72	0.984	0.19	0.24	1.93E-03	5.03E-03
	sip_com	57	13	12	72	0.949	0.15	0.18	8.54E-03	1.31E-02
	sip_emo	44	23	12	72	0.990	0.21	0.26	1.13E-03	3.61E-03
	sip_house	47	35	9	72	0.845	0.11	0.13	4.18E-02	4.73E-02
	sip_mob	60	17	7	72	0.982	0.19	0.23	2.29E-03	5.04E-03
	sip_physical	53	17	12	72	0.934	0.15	0.17	1.19E-02	1.68E-02
	sip_psychosoc	44	27	9	72	0.987	0.20	0.25	1.49E-03	4.42E-03
	sip_social	40	27	13	72	0.946	0.15	0.18	9.48E-03	1.43E-02

Supplementary Table 2: Neuropsychological scores are grouped according to functional domains, and the relative acronyms are reported. The percentage of variance explained in each principal component is reported, evaluating the association between neuropsychological score measured and probability of localisation in the disconnectome morphospace. The reported degree of freedom (df), power calculation (1-β, G*power software), regression coefficient (R^2) and effect size (f^2) describe the correlation obtained in the multiple regression model with PCAs as independent variables and neuropsychological scores as dependent variables (large f^2 above 0.35, medium above 0.15 and small above 0.02) [10]. The corresponding multiple regression model p-values uncorrected and corrected for false discovery rate (FDR) are reported. Asterixis indicate scores with p-value>0.05.

**Neuropsychological scores**		**Training set – Dataset 2**		**Validation set – Dataset 3**	
**Domain**	**Acronym**	**N patients**	**MAE (%)**	**N patients**	**MAE (%)**
Motor	laragrasp	79	10.0	20	4.4
	laragrip	79	9.9	20	6.4
	larapinch	78	11.8	20	8.7
	lgrip	73	18.6	14	21.8
	lpegs	73	14.2	14	17.2
	lshflex	71	13.8	13	10.8
	lwrext	73	17.0	14	10.9
	raragrasp	79	9.5	20	9.6
	raragrip	79	9.2	20	9.7
	rarapinch	79	12.2	20	10.4
	rgrip	73	17.4	14	24.1
	rpegs	73	16.0	14	16.4

	rshflex	73	10.5	13	16.4
	rwrext	73	14.0	14	13.8
	walk_total	79	15.5	20	17.8
Language	reading_raw	77	7.5	20	10.5
	animal_raw	79	11.6	20	17.0
	boston_raw	79	10.2	20	8.8
	commands_raw	73	3.3	14	7.8
	nonword	77	15.1	20	19.0
	reading_comp_raw	77	10.2	20	14.3
	word_raw	79	2.4	20	5.2
Visuospatial attention	bit_coc	72	10.4	13	13.8
	bit_ltot_miss	72	11.9	13	13.7
	bit_rtot_miss	72	12.3	13	8.9
	bit_tot_miss	72	14.0	13	11.9
	mes_coc	78	9.4	20	14.0
	mes_l_miss	78	8.4	20	11.5
	mes_r_miss	78	8.5	20	10.5
	mes_tot_miss	78	7.4	20	9.6
	pos_acc_avg	75	2.7	19	9.0
	pos_acc_disengage	75	11.2	19	13.3
	pos_acc_li	75	4.1	19	15.1
	pos_acc_lv	75	2.6	19	13.9
	pos_acc_ri	75	4.2	19	4.6
	pos_acc_rv	75	2.6	19	5.1
	pos_acc_validity	75	16.6	19	15.1
	pos_acc_vf	75	2.6	19	16.5
	pos_rt_avg	75	7.4	19	14.5
	pos_rt_disengage	75	12.4	19	19.8
	pos_rt_li	75	5.2	19	13.1
	pos_rt_lv	75	4.9	19	13.0
	pos_rt_ri	75	7.3	19	11.2
	pos_rt_rv	75	9.4	19	14.3
	pos_rt_validity	75	13.8	19	22.3
	pos_rt_vf	75	3.8	19	12.5
	pos_sub_avg	69	7.6	13	16.4
	pos_sub_disengage	69	18.2	13	21.3

	pos_sub_li	69	7.0	13	14.3
	pos_sub_lv	69	5.7	13	16.1
	pos_sub_ri	69	8.9	13	14.4
	pos_sub_rv	69	7.4	13	14.0
	pos_sub_validity	69	19.4	13	22.0
	pos_sub_vf	69	3.8	13	16.9
Visuospatial memory	bvmt_bias	79	9.9	20	12.3
	bvmt_delay	79	23.2	20	20.8
	bvmt_delayt	79	21.9	19	21.8
	bvmt_discrim	79	13.3	20	16.5
	bvmt_fa	79	11.0	20	10.4
	bvmt_hit	79	5.9	20	9.7
	bvmt_im	79	21.4	20	16.1
	bvmt_imt	79	20.7	19	18.0
	bvmt_learn	79	21.5	20	29.6
	bvmt_perc	79	7.0	20	5.2
Verbal memory	hvlt_delay	78	21.8	17	25.0
	hvlt_delayt	79	24.5	17	23.9
	hvlt_discrim	78	12.6	17	19.1
	hvlt_discrimt	79	19.9	17	27.0
	hvlt_fa1	78	17.9	17	18.8
	hvlt_fa2	78	4.5	17	15.8
	hvlt_fa3	78	17.6	17	18.9
	hvlt_hit	78	7.6	17	11.4
	hvlt_imt	79	16.0	17	18.1
	hvlt_learn	78	22.9	17	24.4
	hvlt_perc	78	14.2	17	14.4
Pain	pain	68	13.9	17	12.7
Sickness	sip_alert	73	30.6	13	39.2
	sip_amb	73	28.0	13	38.9
	sip_body	73	24.2	13	19.7
	sip_com	73	28.0	13	26.7
	sip_emo	73	21.7	13	20.3
	sip_house	73	27.9	13	33.3
	sip_mob	73	24.4	13	26.7
	sip_physical	73	19.8	13	21.0
	sip_psychosoc	73	20.1	13	25.8
	sip_social	73	22.1	13	23.5

Supplementary Table 3: The DSD prediction error of each neuropsychological score is reported as the relative Mean Absolute Error (MAE). Neuropsychological scores are grouped according to functional domains. N patients indicate the number of patients included to inform the regression model (training phase, dataset 2), and to test it (validation phase, dataset 3).

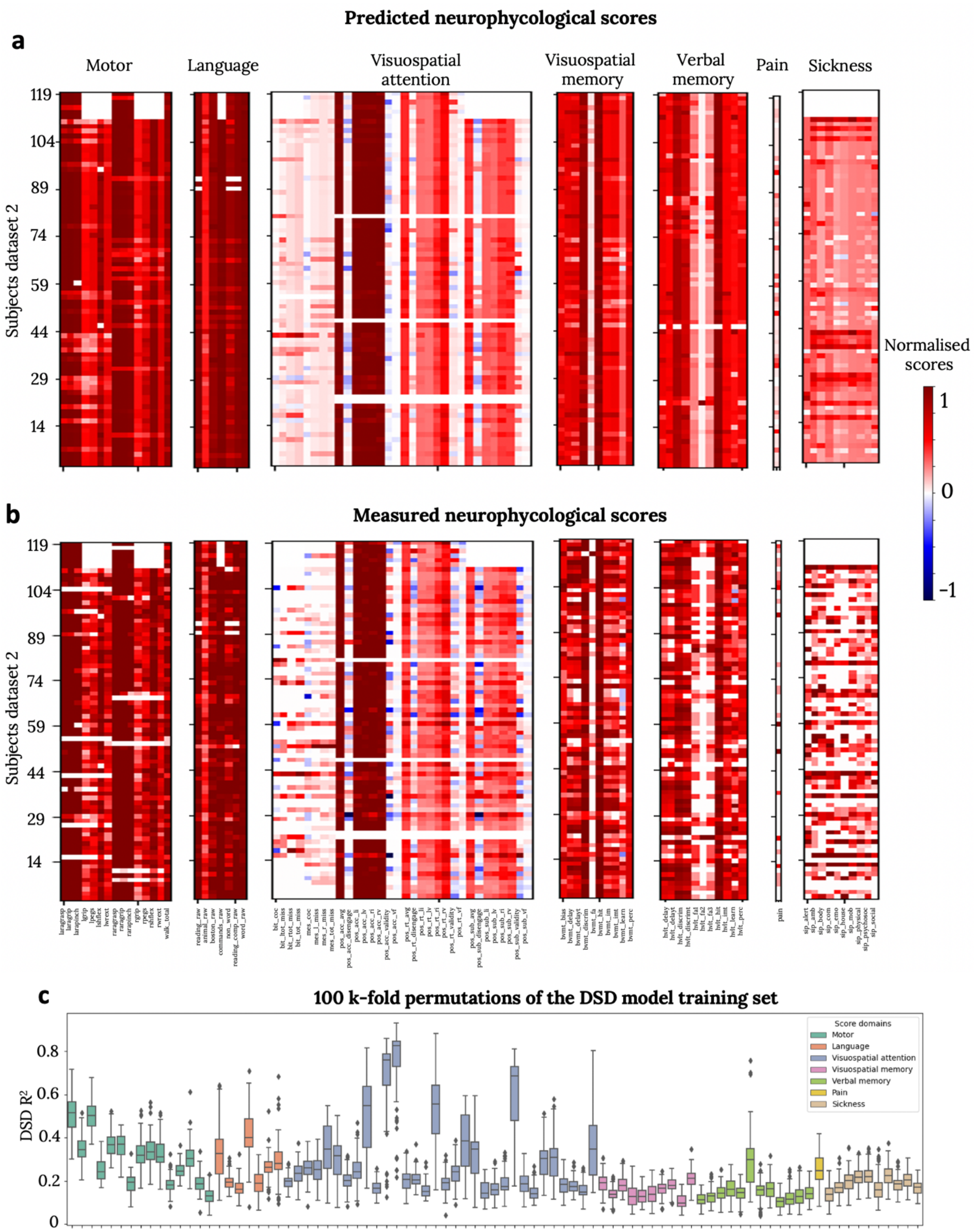

Supplementary Figure 4: Validation of the neuropsychological score prediction. Rows indicate different subjects (derived from training phase, dataset 2) and columns correspond to neuropsychological scores, grouped according to domains. Upper panel (a) corresponds to the predicted

neuropsychological scores by the disconnectome morphospace prediction model. Lower panel (b) indicates measured scores during patients' neuropsychological evaluation at 1-year after the stroke onset. (c) DSD R^2 results plotted across the N=86 neuropsychological scores ordered by the main cognitive/functional domains. Median, interquartile, and the whiskers extend to the rest of the distribution across N=100 k-fold iterations is shown.

Supplementary references

1. Xu T, Jha A, Nachev P. The dimensionalities of lesion-deficit mapping. *Neuropsychologia*. Jul 1 2018;115:134-141. doi:10.1016/j.neuropsychologia.2017.09.007
2. Corbetta M, Ramsey L, Callejas A*, et al*. Common behavioral clusters and subcortical anatomy in stroke. *Neuron*. Mar 4 2015;85(5):927-41. doi:10.1016/j.neuron.2015.02.027
3. Bowren M, Bruss J, Manzel K*, et al*. Post-stroke outcomes predicted from multivariate lesion-behaviour and lesion network mapping. *Brain*. May 24 2022;145(4):1338-1353. doi:10.1093/brain/awac010
4. Foulon C, Cerliani L, Kinkingnehun S*, et al*. Advanced lesion symptom mapping analyses and implementation as BCBtoolkit. *Gigascience*. Feb 8 2018;7(3)doi:10.1093/gigascience/giy004
5. Nachev P, Coulthard E, Jager HR, Kennard C, Husain M. Enantiomorphic normalization of focally lesioned brains. *Neuroimage*. Feb 1 2008;39(3):1215-26. doi:10.1016/j.neuroimage.2007.10.002
6. Avants BB, Tustison NJ, Song G, Cook PA, Klein A, Gee JC. A reproducible evaluation of ANTs similarity metric performance in brain image registration. *Neuroimage*. Feb 1 2011;54(3):2033-2044. doi:10.1016/j.neuroimage.2010.09.025
7. Dai S, Piscicelli C, Lemaire C*, et al*. Recovery of balance and gait after stroke is deteriorated by confluent white matter hyperintensities: Cohort study. *Annals of Physical and Rehabilitation Medicine*. Jan 2022;65(1)doi:ARTN 101488
10.1016/j.rehab.2021.101488
8. Longstreth WT, Jr., Manolio TA, Arnold A*, et al*. Clinical correlates of white matter findings on cranial magnetic resonance imaging of 3301 elderly people. The Cardiovascular Health Study. *Stroke*. Aug 1996;27(8):1274-82. doi:10.1161/01.str.27.8.1274
9. Salvalaggio A, De Filippo De Grazia M, Zorzi M, Thiebaut de Schotten M, Corbetta M. Post-stroke deficit prediction from lesion and indirect structural and functional disconnection. *Brain*. Jul 1 2020;143(7):2173-2188. doi:10.1093/brain/awaa156
10. Cohen J. *Statistical Power Analysis for the Behavioral Sciences*. 2nd Edition ed. Routledge; 1988.
