# Supplementary material for: Latent disconnectome prediction of long-term cognitive-behavioural symptoms in stroke
Source: Brain. 2023 Mar 16;146(5):1963–78. doi: 10.1093/brain/awad013 (PMC10151183; doi:10.1093/brain/awad013)
Supplement: awad013_Supplementary_Data [file awad013_Supplementary_Data.zip › brain-2022-00965-File011.pdf]

Supplementary Materials

TABLE OF CONTENTS

C. Neuropsychological evaluations and composite morphospace maps

C.1 Motor functions. Supplementary Figures 5-14 p.2

C.2 Language functions. Supplementary Figures 16-19. p.11

C.3 Visuospatial attention. Supplementary Figures 20-31. p.18

C. Neuropsychological evaluations and composite morphospace maps

C.1 Motor functions

The tests assessing the patients' upper limbs motor functions investigate the general movement abilities of the hand via the Action Research Arm test [1,2], the grip strength [3], the dexterity via the 9-Hole Peg test [4], the shoulder flexion and wrist extension [5]. The motor abilities of the lower limbs have been examined via the combined walking index.

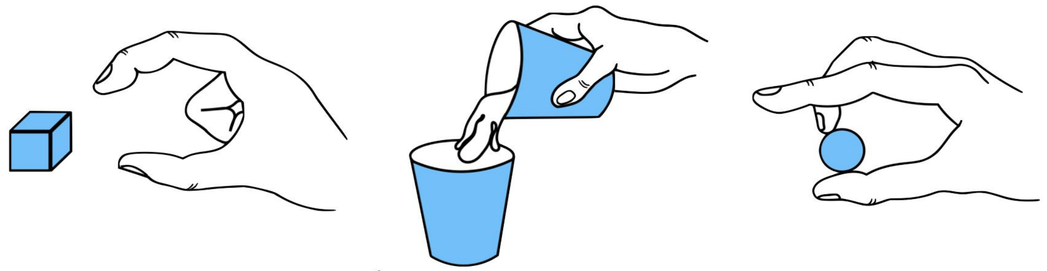

Supplementary Figure 5: Examples of the Action Research Arm subtests. a) First item of the grasp subtest. b) First item of the grip subtest. c) First item of the pitch subtest.

Action Research Arm - ARA test

The ARA test [2] assesses the ability to perform purposeful movements with the upper limb extremities. Specifically, it tests the ability to grasp, grip and pinch objects of different weights and shapes, and perform gross movements with the limb.

The ARA has four subtests of 19 items in total. Each item is rated on a four-point scale (0-3); higher scores indicate better performance. As the test aims to speed up the examination time, the score of 'three' on the first item of each subtest credits the patients with a score of 'three' in all the remaining items without completing the subtest. If the patients score less than 'three' in the first item, item two (the easiest item) is assessed. The score of 'zero' in item two credits the patients with the 'zero' score in the other items of the subtest, as it is unlikely for the patients to accomplish the remaining tasks. If the patients score less than 'three' on item one and more than 'zero' on item two, all the remaining items are administered [2]. In clinical practice, the ARA is assessed on the impaired and unimpaired limbs, separately [2]. For this study, only the scores of the impaired limb have been considered. Furthermore, only the grasp, grip, and pitch subtests have been considered in this study.

<u>Grasp subtest.</u> The grasp subtest is divided into six items. The patients are required to grasp different objects placed on a tray such as four wooden blocks of various sizes, one ball, and one sharpening stone. The first item is grasping the 10 cm block (Supplementary Figure 5a), whilst the second and easiest item is the grasping of the 2.5 cm block. The total subscore of the grasp subtest ranges from 0 to 18.

<u>Grip subtest.</u> Six tools are presented in the grip subtest, such as two plastic tumblers, two metal tubes of different diameters, a washer, and a bolt. In four items, patients are asked to i) pour water glass to glass (first item; Supplementary Figure 5b), ii) grip the 2.5 cm diameter tube (second and easiest item), iii) grip the 1 cm diameter tube, iv) grip the washer over the bolt. The total score of the four items ranges between 0 to 12.

<u>Pitch subtest</u>. During the six items of the pitch subtest, patients are required to bear balls of different sizes and one marble using different fingers. For instance, the first item requires bearing a 6 mm ball between the third finger and the thumb (Supplementary Figure 5c). In the second and easiest item, the

patients pitch a marble with the first finger and the thumb. The total score of the six items ranges between 0 to 18.

Grip strength

The Jamar Dynamometer grip strength assessment investigated patients' grip strength (Supplementary Figure 6). The American Society of Hand Therapists' procedure [6] requires patients to sit on a chair with their back straight and their feet flat on the floor. The examined arm is placed with the elbow flexed at 90°, the fingers flexed as needed for a maximal contraction over the Dynamometer handle, while the forearm and wrist were kept in a neutral position. The patients are asked to take a breath while exerting the maximum grip effort for three consecutive trials. The strength score is recorded in kilograms and the total score is calculated as mean kg over the three trials [6].

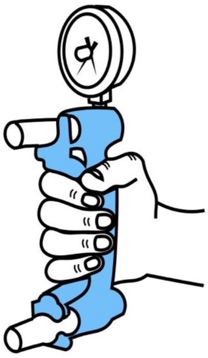

Supplementary Figure 6: Example of the grip strength task.

Dexterity

The hand's ability to coordinate the fingers' movement during objects manipulation in a timely way is defined as manual dexterity [7]. The patients' dexterity has been measured via the 9-Hole Peg test i.e. 9HPT, [8]. The test setting includes a one-piece board with a concave folded dish containing nine pegs next to a 9-holes matrix for the pegs (Supplementary Figure 7). Patients sit on a height-custom chair with the tabletop at midchest level. The task instructions require patients to place and remove the nine pegs one at a time and in random order as quickly as possible [4,9]. The final score is the time in seconds elapsed from when the patients touch the first peg until the last peg is placed back into the dish.

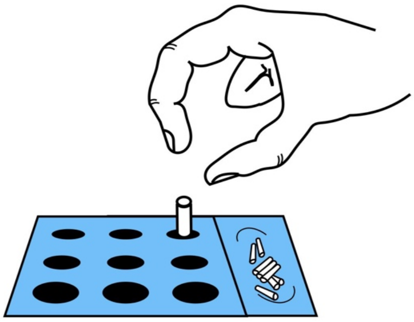

Supplementary Figure 7: Example of the 9-Hole Peg test.

Shoulder flexion and Wrist extension

The shoulder flexion and wrist extension assessments [5] investigate patients' movement range of the upper limb and extremity using a goniometer. The main muscles involved in the shoulder flexion are

the anterior deltoid and coracobrachialis. During the examination for shoulder flexion, patients are asked to raise their arm against gravity as high as they can, while sitting on the chair. The wrist extension requires mainly the activation of the extensor carpi radialis longus, extensor carpi radialis brevis, and extensor carpi ulnaris [5]. The patients sit with their arms on the table in a resting position and with palms down. They are asked to bend back their wrist against gravity. The movement amplitude in the two tasks is recorded as angle grades of the goniometer aligned to the shoulder and wrist respectively (Supplementary Figure 8).

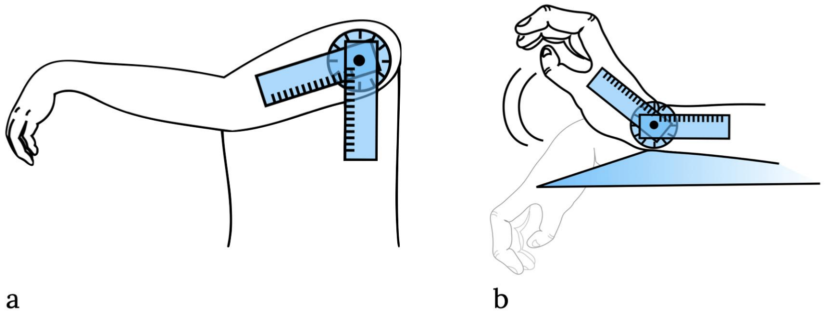

a b

Supplementary Figure 8: Example of the a) shoulder flexion and b) wrist extension tasks.

Combined walking index

To assess patients' lower limbs ability a combined walking index has been computed [10]. The walking index combines the scores of the 10-meters walking test i.e. 10MWT, [11; Supplementary Figure 9a] and the Functional Independence Measure i.e. FIM, [12; Supplementary Figure 9b] to capture the variability of maximally and minimally impaired patients. The 10MWT requires patients to walk unassisted if safely able to do so for 10 meters. The time required to complete the task is recorded in seconds to compute the gait speed (meters/second). Patients unable to safely execute the task have been assessed via the walking item of the FIM. Each patient is assigned with a final, combined 1-9 score which describes on the same scale the performances of maximally and minimally impaired patients. describes the abilities of maximally impaired patients, assessed via the FIM, scores 8 and 9 assigned to each patient Specifically, the scores 1 to 6 correspond to the ranking of patients' walking ability assigned by the therapist according to the FIM (Supplementary Figure 9b): i) the score of one indicates total assistance required, ii) the score of two is assigned when maximal assistance is required, iii) the score of three denotes moderate assistance required, iv) the score of 4 is assigned when minimal contact assistance is needed, v) the score of 5 indicates standby assistance, vi) the score of 6 is assigned when use of assistive device. Scores from 7 to 9 are assigned to independent walking patients who are assessed via the 10MWT: i) the score of seven is assigned at a speed of <0.4 meters/second, ii) the score of 8 indicates a speed of 0.4 to 0.8 meters/second, iii) the score of 9 indicates a speed greater than 0.8 meters/second.

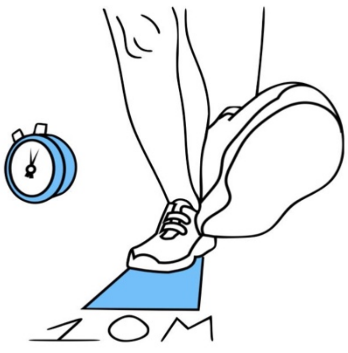

Item: Locomotion - Walk	
Score	Level of Functioning
1	Total assist
2	Maximal assist
3	Moderate assist
4	Minimal assist
5	Supervision
6	Modified independency

a b

Supplementary Figure 9: Example of the a) the 10-meters walking test and b) the walking ability ranking of the Functional Independence Measure.

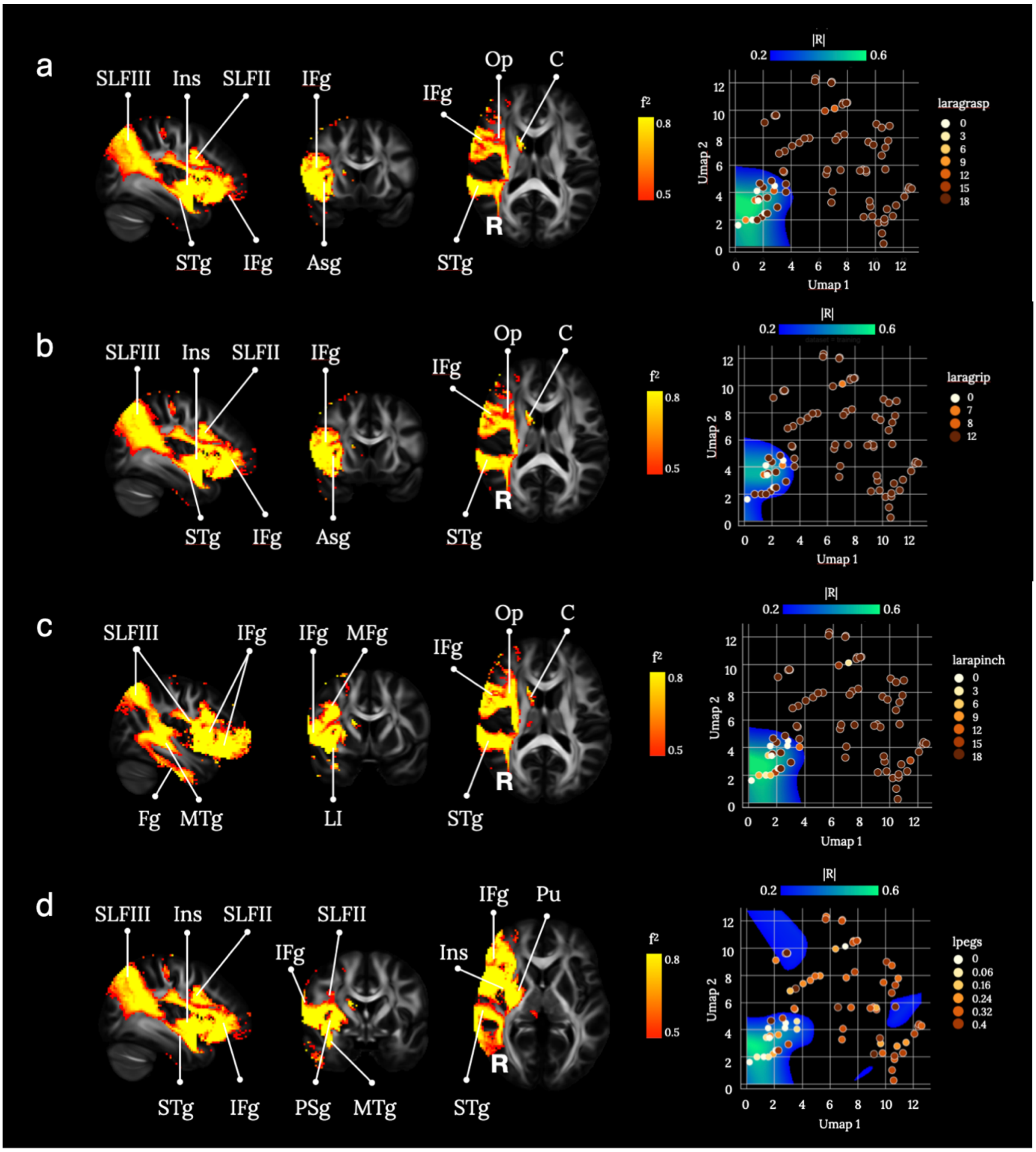

Supplementary Figure 10: Brain disconnections and UMAP related territories contributing significantly to the three subtests (grasp, grip, pitch) of the left hand Action Research Arm (ARA), and the 9-Hole Pegs (9HP) tests. (a) Grasp subtest of the ARA (laragrasp), (b) Grip subtest of the ARA (laragrip), (c) Pitch subtest of the ARA (larapitch), (d) 9HP test (lpegs). ASg: Anterior Short insular gyrus; C: Caudate; Fg: Fusiform gyrus; IFg: Inferior Frontal gyrus; IFg: Inferior Frontal gyrus; Ins: Insula; LI: Limen Insulae; MFg: Middle Frontal gyrus; MTg: Middle Temporal gyrus; Op: Operculum; PSg: Posterior Short insular gyrus ; Pu: Putamen; SLFII: second branch of the Superior Longitudinal Fasciculus; SLFIII: third branch of the Superior Longitudinal Fasciculus. Th: Thalamus. Maps are freely available at https://neurovault.org/collections/11260/.

The disconnection in the right hemisphere of frontoparietal structures, the caudate, the post central gyrus, and the insular and temporal cortices cluster in correspondence to low scores in the three subtests

(grasp, grip, pitch) of the left hand ARA, and the 9HP test. Studies on healthy and clinical populations showed that the movement execution of the left upper limb is subserved mainly by structures of the right hemisphere [13,14] and damage to the connections between motor areas and the spinal cord play a key role in motor impairment after stroke. Nevertheless, our findings reveal that the prediction of the patients' left upper limb motor abilities is given by the disconnection of structures deputed to higher cognitive functions.

Our results confirm that the integrity of the right fronto-parietal network seems to be crucial to execute fine contralesional-hand movements and its disconnection predicts the patients' motor abilities after stroke. Previous studies reported the contribution of right frontoparietal structures in the execution of fine left-hand movements such as grasping and gripping [14-17] and clinical evidence showed that the lesion to frontal and parietal structures impairs the execution of fine hand actions [13]. The right fronto-parietal network subserves monitoring and attentional processes during the movement execution [14,16,18]. In particular, it has been proposed that parietal and inferior frontal structures contribute to generating and maintaining the prediction of the motor outcome, and the updating of sensory expectations regarding the movement is prevented after their damage [19]. In addition, the lesion to the parietal cortex was found to prevent the voluntary initiation of the movement i.e. anarchic hand, [20].

The damage to frontal-parietal structures and the insular cortex and their association to poor motor performance suggest that bodily-related processes might play a role in motor outcome after stroke [21-25]. For instance, studies on awareness disorders specific for motor upper limb paralysis suggest that fronto-parietal-insular attentional networks may prevent the updating of the information about patients' current motor abilities [24,26]. Moreover, the lack of sense of ownership of one's own upper limb can occur after the disconnection of fronto-parietal-insular network [27-29]. These pathologies represent a burden on the success of patients' motor rehabilitation [30,31]. Hence the results presented in Figure 10 are consistent with the literature in stroke, healthy controls variability and recordings in non-human primates.

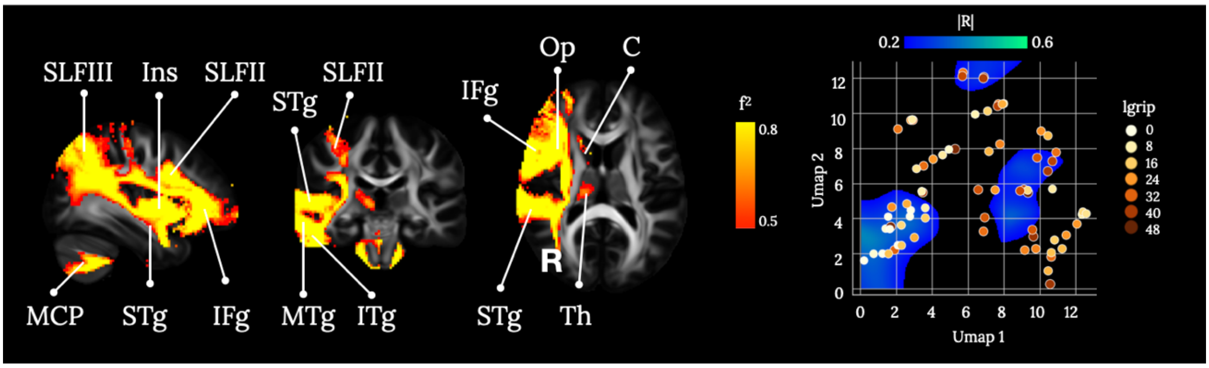

Supplementary Figure 11: Brain disconnections and UMAP related territories contributing significantly to the left-hand grip strength (lgrip) assessment. C: Caudate; IFg: Inferior Frontal gyrus; IFg: Inferior Frontal gyrus; Ins: Insula; ITg: Inferior Temporal gyrus; MCP: Middle Cerebellar Peduncles; MTg: Middle Temporal gyrus; Op: Operculum; SLFII: second branch of the Superior Longitudinal Fasciculus; SLFIII: third branch of the Superior Longitudinal Fasciculus; STg: Superior Temporal gyrus; Th: Thalamus. Maps are freely available at https://neurovault.org/collections/11260/ .

The disconnection of the cortico-pontine-cerebellar loop together with fronto-parietal disconnection observed in prediction of the grip strength assessment scores confirm the participation of the cerebellum in specific components of the movement [32] and the prediction and updating stages of the action [33,34]. For instance, the joint activity of the fronto-parietal network and the cerebellum is correlated to the calibration of the movement and force exerted according to different object weights [32,35]. This is in line with studies on patients with cerebellar damage, who show inaccurate grip force adjustments during object manipulation [13] and impaired on-line correction of the movement [36].

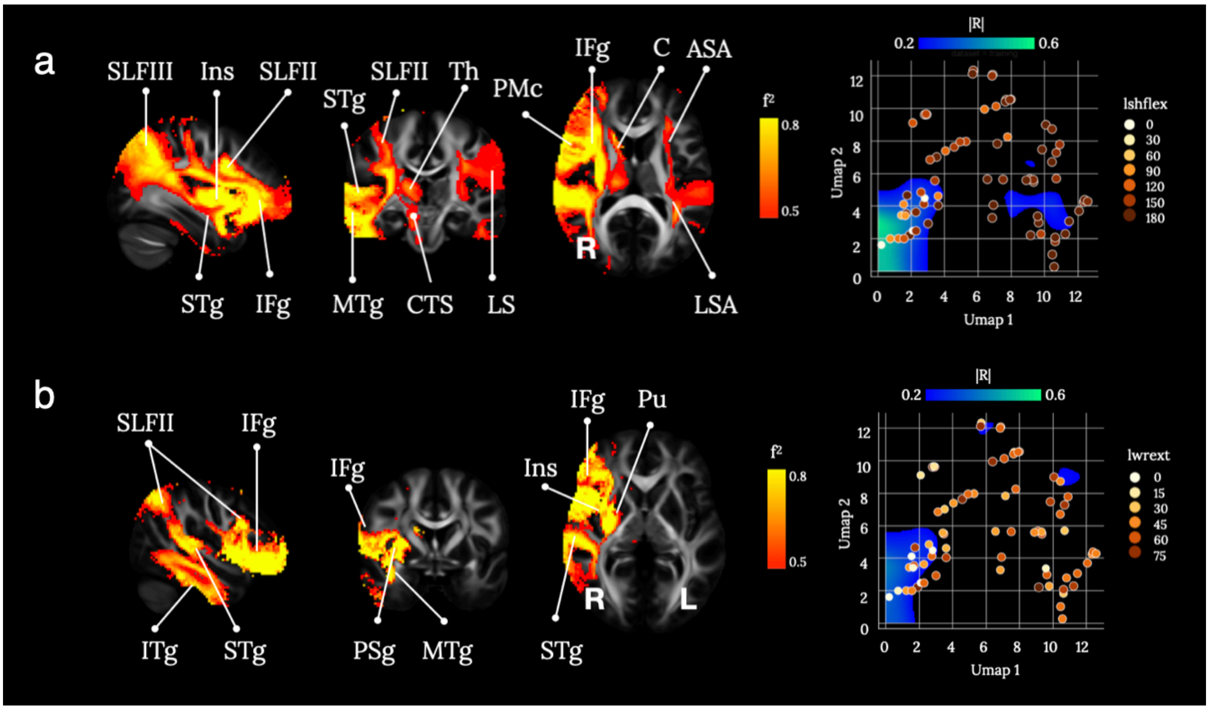

Supplementary Figure 12: Brain disconnections and UMAP related territories contributing significantly to the (a) left shoulder flexion (lshflex), and (b) left wrist extension (lwrex). ASA: Anterior Segment of the arcuate fasciculus; C: Caudate; CST: Cortico-Spinal-Tract; IFg: Inferior Frontal gyrus; IFg: Inferior Frontal gyrus; Ins: Insula; ITg: Inferior Temporal gyrus; LSA: Long Segment of the Arcuate fasciculus; MTg: Middle Temporal gyrus; PSg: Posterior Short insular gyrus ; Pu: Putamen; SLFII: second branch of the Superior Longitudinal Fasciculus; SLFIII: third branch of the Superior Longitudinal Fasciculus; STg: Superior Temporal gyrus. Maps are freely available at https://neurovault.org/collections/11260/ .

The disconnection of fronto-parietal and temporal-parietal structures predicting low scores in wrist flexion and shoulder extension assessments is in line with the results of this study and previous findings on the right hemisphere role in sensorimotor stabilisation during motor execution [37,38]. However, the activation of left hemisphere structures damaged during shoulder flexion confirms the hypothesis of a degree of hemispheric specialisation in motor dynamics [38,39]. Although contralesional motor deficits are more prominent after stroke, the impairment of the ipsilesional limb has been observed [40]. Differences in the extent of the ipsilesional deficits suggest that the left hemisphere supports multi-joint coordination and its damage prevents the accurate limb trajectory during a reaching movement [41]. Thus, the pivotal role of the shoulder in the multi-joint coordination for the upper limb motor performance [42,43] is confirmed by the presence of ipsilesional disconnection only in the prediction of left shoulder extension deficits, compared to the other left limb assessments.

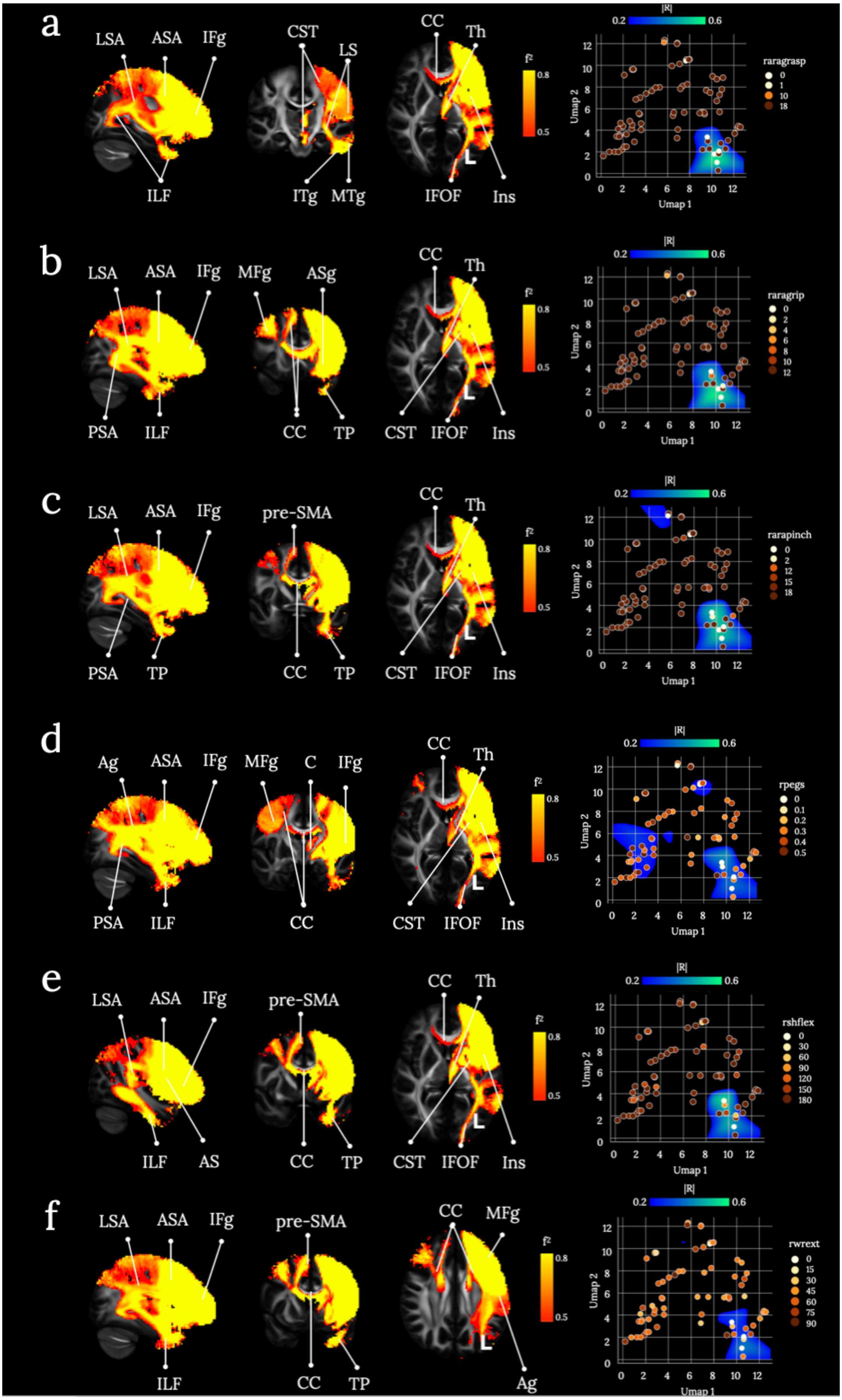

Supplementary Figure 13: Brain disconnections and UMAP related territories contributing significantly to the three subtests (grasp, grip, pitch) of the right hand Action Research Arm (ARA), the

9-Hole Pegs (9HP) test, and the right shoulder flexion and wrist extension. (a) Grasp subtest of the ARA (raragrasp), (b) Grip subtest of the ARA (raragrip), (c) Pitch subtest of the ARA (rarapitch), (d) 9HP test (rpegs), (e) left shoulder flexion (lshflex), and (f) left wrist extension (lwrex). Ag: Angular gyrus; ASA: Anterior Segment of the arcuate fasciculus; ASg: Anterior Short insular gyrus; C: Caudate; CC: Corpus Callosum; CST: Cortico-Spinal-Tract; IFg: Inferior Frontal gyrus; IFOF: Inferior Fronto-Occipital Fasciculus; ILF: Inferior Longitudinal Fasciculus; Ins: Insula; ITg: Inferior Temporal gyrus; LSA: Long Segment of the Arcuate fasciculus; MFg: Middle Frontal gyrus; pre-SMA: pre-Supplementary Motor Area; PSA: Posterior Segment of the Arcuate fasciculus; Th: Thalamus; TP: Temporal Pole. Maps are freely available at https://neurovault.org/collections/11260/ .

An extensive disconnection of the left hemisphere clusters in correspondence to low scores for the right hand in the three subtests (grasp, grip, pitch) of the ARA, 9HP test, the arm flexion and wrist extension tasks. The disconnection involved in the left hemisphere the cortico-spinal tract, postcentral gyrus, fronto-parietal, temporo-occipital and occipito-frontal connections, and the interruption of frontal interhemispheric connections in correspondence to the motor section of the corpus callosum. Our results confirm that the disconnection of the corticospinal tract is associated with the motor outcome after brain damage. Previous studies demonstrated that the integrity of the cortico-spinal tract is a reliable predictor of the patients' motor abilities up to one year after the stroke [44] In particular, integrity of the cortico-spinal tract has been associated with the hand dexterity performance [45] and hand strength [46,47] stroke patients. Furthermore, previous studies showed that the severity of motor impairment is predicted by the integrity of the corpus callosum connection of the primary motor areas [48,49], and reduced interhemispheric resting-state connectivity between motor structures has been associated to motor impairment [50]. In addition, studies on callosal patients showed that the lack of callosal connection affects the grip formation in reach-to-grasp movements [51]. These previous findings are in line with our results suggesting that a callosal disconnection of motor and sensory cortices predicts the movements abilities of stroke patients.
The prediction of low scores in ASA, 9-HP, and shoulder flexion and wrist extension via the disconnection of fronto-parietal and temporo-occipital structures, and the insula indicate that motor outcome after left hemisphere stroke is not related only to sensorimotor functions. In fact, movement impairment after left hemisphere stroke can manifest as the inability to perform voluntary, skilled movements such as tool use and gesture imitation i.e. limb apraxia, [52]. Limb apraxia has been associated usually with an extensive network of structures within the left hemisphere but see [53,54,55`]. The damage of left fronto-parietal structures is associated with kinematic deficits during object-related gesture movements [56-58] and non-object related imitation gestures [59,60]. Some studies have explored the neural structures related to specific errors performed by apraxic patients during movement (e.g. spatio-temporal and action-content errors) and revealed the involvement of the temporal lobe, the temporo-occipital cortex, and the insula [54,58,61].

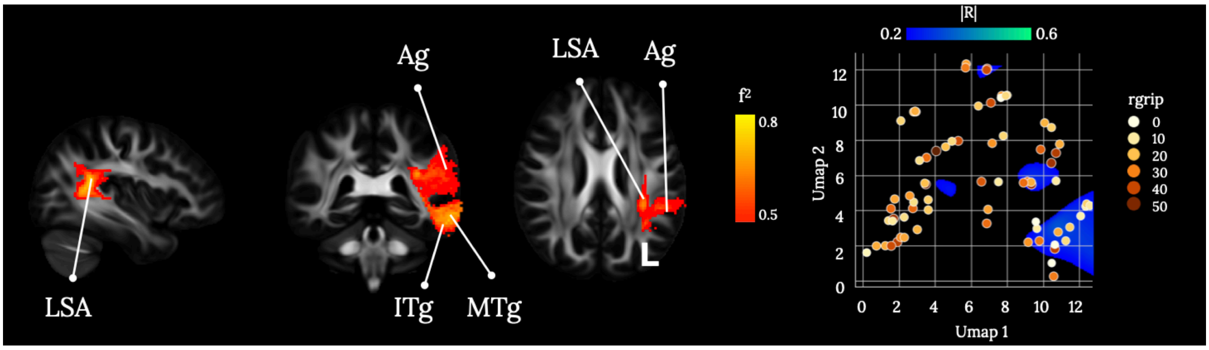

Supplementary Figure 14: Brain disconnections and UMAP related territories contributing significantly to the right-hand grip strength (rgrip) assessment. Ag: Angular gyrus; ITg: Inferior

Temporal gyrus; LSA: Long Segment of the Arcuate fasciculus; MTg: Middle Temporal gyrus. Maps are freely available at https://neurovault.org/collections/11260/.

The low scores of grip strength assessment are predicted by the solely disconnection of the angular gyrus from the temporal cortex via the damage of the long segment of the arcuate Fasciculus. These results are in contrast with other findings that correlate grip strength assessed via the dynamometer with portico-spinal tract integrity [46,47,62]. However, our results partially overlap the recent findings of Garcea and colleagues [63], who report the association of reduced right-hand grip strength with lesion to the inferior parietal, middle and superior temporal cortices. Interestingly, our findings suggest that grip strength ability of the right hand may rely on the left hemisphere ventro-dorsal stream conveying information on object-related actions [64].

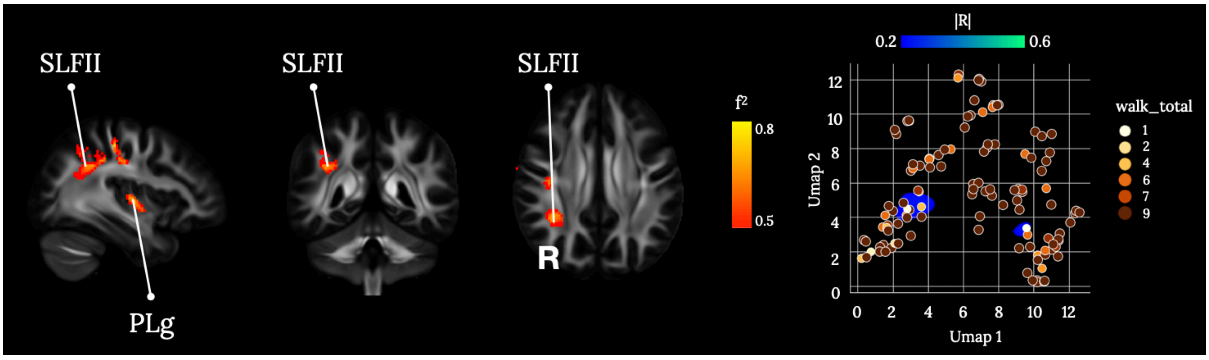

Supplementary Figure 15: Brain disconnections and UMAP related territories contributing significantly to the combined walking index. PLg: posterior long insular gyrus; SLFII: second branch of the Superior Longitudinal Fasciculus. Maps are freely available at https://neurovault.org/collections/11260/ .

The low combined scores for walking abilities cluster in correspondence of the disconnection within the right hemisphere of the second branch of the superior longitudinal fasciculus. Our results indicate that cognitive control and monitoring processes subserved by the frontoparietal network are necessary for spared motor abilities. Previous studies on the neural substrate of walking and gait impairment typically pinpointed to the lesion of the CST [65], suggesting that the disconnection of the primary motor cortices from the spinal cord is the main predictor of lower limbs motor outcome after stroke. However, the participation of other cortical and subcortical structures in walking abilities has been revealed [66,67], such as the corpus callosum, the putamen, and the insula. The contribution of frontoparietal structures and connections has been also reported, unveiling the involvement of cognitive control mechanism in abnormal gate and speed components of walking [66]. In particular, the connection between inferior-parietal and middle-frontal structures via Superior Longitudinal Fasciculus II is involved in spatial and top-down attentional processes [68,69]. The present results indicate that an efficient top-down control of the movement may predict more accurately the lower limb motor abilities than the integrity of motor and sensorimotor mechanisms.

C.2 Language functions

Language was assessed using the 1-min animal verbal fluency test and the Boston Diagnostic Aphasia Examination (BDAE). Familiarity with the Boston school classification of aphasias is necessary to interpret the BDAE e.g. [70], and other non-western assessment batteries have since been put forward e.g. [71].

Verbal fluency tests, like the semantic fluency test, rely on the retrieval of specific information from memory and to verbally produce a list of words. Two types of restrictions are commonly applied: phonological (e.g., letter fluency) and semantic (e.g., animals) clustering (see Supplementary Figure 16). The outcome measure is the speed and ease of production and the final number of words generated in a given time (e.g., 1 min). This group of tests is sometimes considered as speech/language assessments and sometimes as executive functioning tests [72]. This is a sensitive clinical test as brain lesions can alter the speed and ease of verbal production in patients. Acquired aphasia after brain lesions or subsequent to neurodegeneration is commonly associated with greatly reduced verbal productivity [73-77]. Particularly left frontal lobe lesions have been linked to reduced verbal fluency, especially if the lesion encompasses 'Broca's area' in the inferior frontal gyrus [74]. Recently, the frontal aslant tract (FAT) came into focus as a relevant pathway for verbal fluency [77]. The FAT connects the inferior frontal gyrus to the supplementary motor region and lateral superior frontal gyrus. The role of these cortical and white matter structures for fluency was further evaluated in awake surgical settings where direct cortical stimulation induced temporary interruptions to these networks [78]. However, the FAT is also involved in non-speech and language functions [79]. As education, sex, and age have been shown to impact the quantity and diversity of animal recollection and the raw scores are therefore usually corrected e.g. [80].

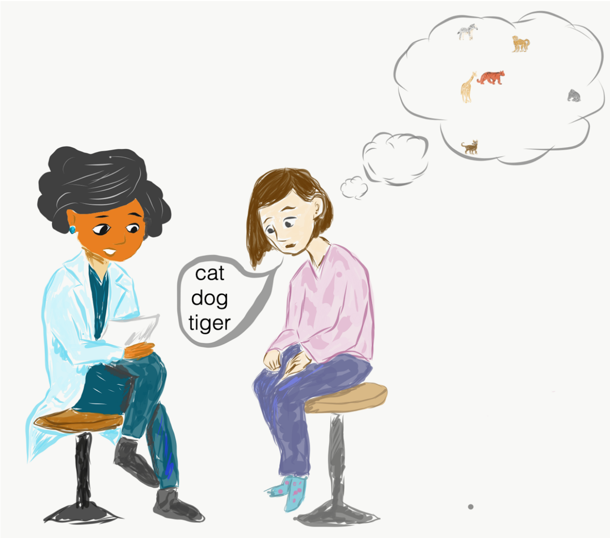

Supplementary Figure 16: Example of a time-limited (1min) animal verbal fluency task.

Boston Naming test (BNT, short version). The BNT is a popular test of visual confrontation naming, where the patient is shown line drawings of objects with increasing difficulty, ranging from high-frequency (e.g. toothbrush) to low-frequency words (e.g. protractor) [81]. Patients are asked to name the objects within 20 seconds, afterwards a phonemic or semantic cue can be given by the examiner (see Supplementary Figure 17). A shortened version was developed that includes only 15 items which was adopted in the most recent version of the BDAE-3 [82,83] and other test batteries (e.g. CERAD, https://www.memoryclinic.ch/fileadmin/user_upload/Memory_Clinic/CERAD-Plus/CERAD-Plus_Testheft.pdf) and is available in multiple languages. The scoring considers the total number of correct responses, cued responses, and an error code or paraphasia type analysis can also be conducted. Due to the limited original normative data, many other normative data had been provided over the years and also showed an interaction of sex, education, socioeconomic background, and age e.g. for the short

version see [84,85,86]. The maximum score for the short version is 15, the scale can be interpreted as the higher the better. The items (and their semantic cues) of the short form include:

1. House (home, a kind of building)
2. Comb (used for fixing hair)
3. Toothbrush (used in the mouth)
4. Octopus (an ocean animal)
5. Bench (used for sitting)
6. Volcano (a kind of mountain)
7. Canoe (used in water)
8. Beaver (an animal)
9. Cactus (saguara, something that grows)
10. Hammock (you lie on it)
11. Stethoscope (used by doctors and nurses)
12. Unicorn (mythical animal)
13. Tripod (photographers or surveyors use it)
14. Sphinx (it's found in Egypt)
15. Palette (artists use it)

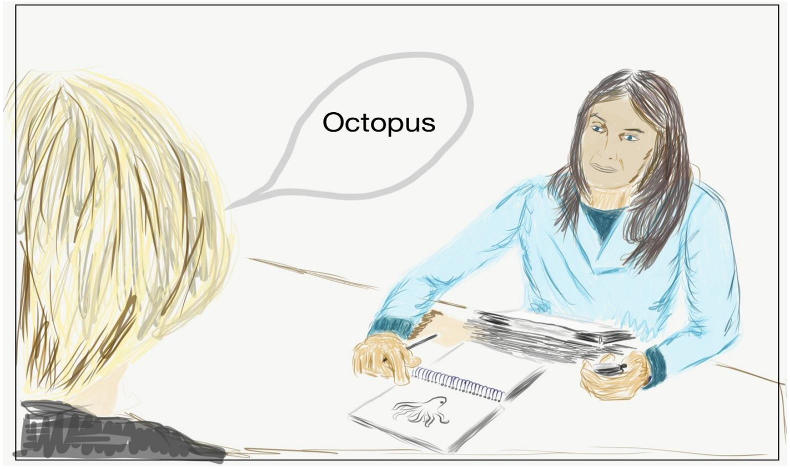

Supplementary Figure 17. Example of the administration of the Boston Naming Test (BNT).

Boston Diagnostic Aphasia Examination (BDAE) is a neuropsychological assessment battery that samples from a range of language components for diagnostic and treatment purposes. The BDAE was originally developed and validated by Goodglass & Kaplan [87] and has since been through several iterations with the latest version also offering a shortened version e.g. [83]. The BDAE is a systematic and comprehensive assessment comprising 34 subtests, which can take up to four hours. Patients are asked to answer a semi structured interview and engage in free conversation. Each subtest is scored for correct responses and converted into percentiles. Normative data is available for all subtests. In this study the following subtests were used: Oral reading of sentences (reading_raw), comprehension (reading_comp_raw), word comprehension (word_raw), Boston picture naming (boston_raw), commands (commands_raw). An additional non-word reading (nonword) test was conducted. Each subtest will be discussed below.

<u>Oral reading of sentences.</u> The read-out-loud sentence reading subtest requires the patient to read ten sentences. The whole sentences must be read without errors to be scored as pass (score of 1) otherwise they are scored as fail (score of 0). The maximum score of the standard form is 10 (short form: 5), and the scale can be interpreted as the higher the better.

1. Summertime.
2. A good beach day.
3. Jim and Mary pack a picnic lunch.
4. They load the car with beach chairs and towels.
5. Off they go with all their equipment.
6. After driving for forty-five minutes, they arrive at the seashore.
7. They decide to go swimming because the water is warm and calm.
8. When they emerge from the water they are famished.
9. That is when they realize they forgot to load their picnic lunch.
10. Luckily, they discover a refreshment stand with a variety of snacks to choose from.

Comprehension of oral reading of sentences. The patients are asked to read each of the comprehension statements aloud and answer by choosing the correct multiple-choice option out of four options. These questions relate to the scenario described in the oral reading of sentences test of the BDAE (see above). The examiner can point to the options on the first run and can ask the patient to select the best completing phrase. The examiner ought not to read the items aloud. The maximum score of the standard form is 5 (short form: 3), and the scale can be interpreted as the higher the better.

1. The weather was …. cool/sunny/crisp/rainy
2. Mary and Jim rode in a …..train/boat/car/plane
3. The trip took about...half a day/five minutes/45 minutes/two hours
4. The water was… rough/warm/ chilly/crowded
5. They forgot to bring a …. towel/umbrella/lunch/swimsuit

Word comprehension. The single word comprehension test belongs to the auditory comprehension assessment of the BDAE and is a basic word discrimination task where stimulus items on the patient's body and in the examination, booklet need to be pointed at after a verbal prompt from the examiner. The stimuli are grouped by body parts, colours, letters, and numbers. If the correct answer is offered within 5 sec the score is 1 point, if the answer takes longer, it is 0.5 point, and 0 for erroneous answers. The maximum score of the standard form is 37 (short form: 16), and the scale can be interpreted as the higher the better.

"Show me your….."

1. Shoulder
2. Cheek
3. Ear
4. Nose
5. Knee

Using the booklet with the picture material the patient is again prompted to point to the following items:

1	Bear	17	J
2	Peanut	18	4
3	Shirt	19	13
4	Bus	20	5
5	Saw	21	20
6	Ant	22	257

7	Tulip	23	Telephone
8	Blue	24	Deer
9	Brown	25	Hamburger
10	Pink	26	Cap
11	Green	27	Wagon
12	Purple	28	Screw
13	T	29	Swan
14	N	30	Spider
15	G	31	Iris
16	K		

Commands. The patient is asked to carry out 1-5 step commands that vary in length and complexity. A point is given for every target element (underlined) that is performed. The command can be repeated in full if requested. The maximum score is 15 (short form: 10), and the scale can be interpreted as the higher the better. Additional items needed for this test are a pencil, a watch, and a card.

1. Make a fist.
2. Point to the ceiling, then to the floor.
3. Put the pencil on top of the card, then put it back.
4. Put the watch on the other side of the pencil and turn over the card.
5. Tap each shoulder twice with two fingers, keeping your eyes shut.

Nonword reading. In this study, we used an experimental measure where the patient was asked to pronounce non-meaningful syllables. Four-letter non words (e.g., NORD) were presented to the patients who were asked to read them aloud. The maximum score for this test is 20, and the scale can be interpreted as the higher the better.

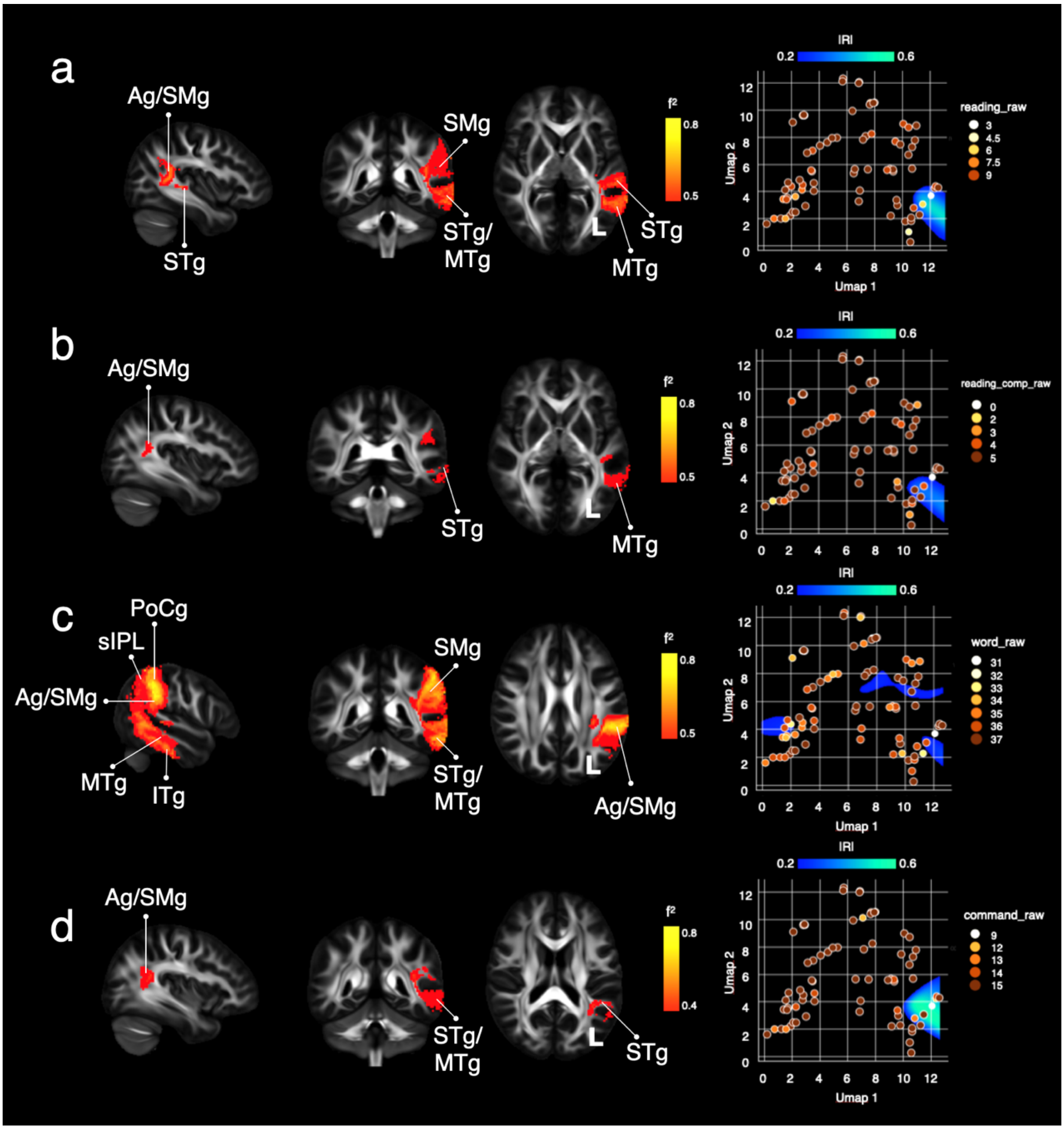

Supplementary Figure 18: Brain disconnections and UMAP related territories contributing significantly to the Boston Diagnostic Aphasia Examination (BDAE). (a) Oral reading of sentences (reading_raw), (b) comprehension (reading_comp_raw), (c) word comprehension (word_raw), (d) commands from the BDAE (command_raw). Ag: Angular gyrus; MSg: Midddle Short insular gyrus; MTg: Middle Temporal gyrus; PoCg: Post-Central gyrus; SPl: superior Parietal lobule; SMg: Supra Marginal gyrus; STg: Superior Temporal gyrus. Maps are freely available at https://neurovault.org/collections/11260.

Profiles of disconnections predict individual deficits on the Boston Diagnostic Aphasia Examination (BDAE) subscales for reading aloud (reading_raw), reading comprehension of sentences (reading_comp_raw) and single word comprehension (word_raw), and impairments when following commands (command_raw).

Reading sentences aloud and sentence comprehension clustered in comparable areas in the UMAP morphospace with large effect size. These clusters corresponded to a significant disconnection of the left temporo-parietal network between the superior and middle temporal gyri and the inferior parietal lobe (e.g., angular and supramarginal gyrus). This temporo-parietal network has been shown to predict

reading performance and the functional specialisation of the visual word form area [88]. This network was already postulated as relevant for reading as early as 1891, when one of Dejerine's case studies presented with pure alexia (reading impairment) and subsequent agraphia (writing impairment) [89]. Lesions associated with alexia are often ascribed to the posterior inferior temporal cortex in the left hemisphere [90,91]. In recent lesion studies using voxel-based lesion symptom mapping in chronic stroke patients the same network emerged as relevant for sentence-level reading that was uniquely associated with the superior and middle temporal gyri as well as the supramarginal gyrus [92]. As such, critical aspects of sentence reading may rely on the ventral visual stream for the identification of familiar words and the integration into a wider language network through parieto-temporal connections, such as the posterior segment of the arcuate fasciculus.

Auditory word comprehension requires the transformation of auditory signals into abstract concepts. As such the adjacent cortex to the primary auditory cortex embedded in the left and right superior temporal gyrus has been consistently shown to be relevant for comprehension in the aphasia stroke literature [93-99]. Recently, other temporal regions have also been implicated such as the temporal pole [100]. Converging evidence from different lines of clinical research suggest that the inferior and middle temporal gyri are associated with word comprehension deficits as the integration of auditory and conceptual processing is interrupted [101]. Additional semantic processing is likely to recruit more anterior temporal regions [100]. Akin to sentence-level comprehension, single words comprehension is also associated with the left supramarginal gyrus [92]. Using fMRI in healthy adult readers, the sensitivity of this network was disentangled whereby the parieto-temporal cortex responded to phonology and the VWFA in the occipital-temporal cortex to orthography [102]. Current models suggest a delicate temporo-parietal and frontal network to be involved in single word comprehension that links lexical concepts (anterior temporal) lemma and lexical-syntactic information (posterior temporal), phoneme-to-motor transfer (parietal-frontal), top-down and executive modulation (frontal) e.g. [103].

Following commands are significantly impaired with disconnections between the inferior parietal lobe and superior and middle temporal gyri. The cluster in the UMAP morphospace is comparable to sentence reading and comprehension. Identifying a similar network is to be expected as speech comprehension can be assessed using 1-2-3-stage commands [104-106]

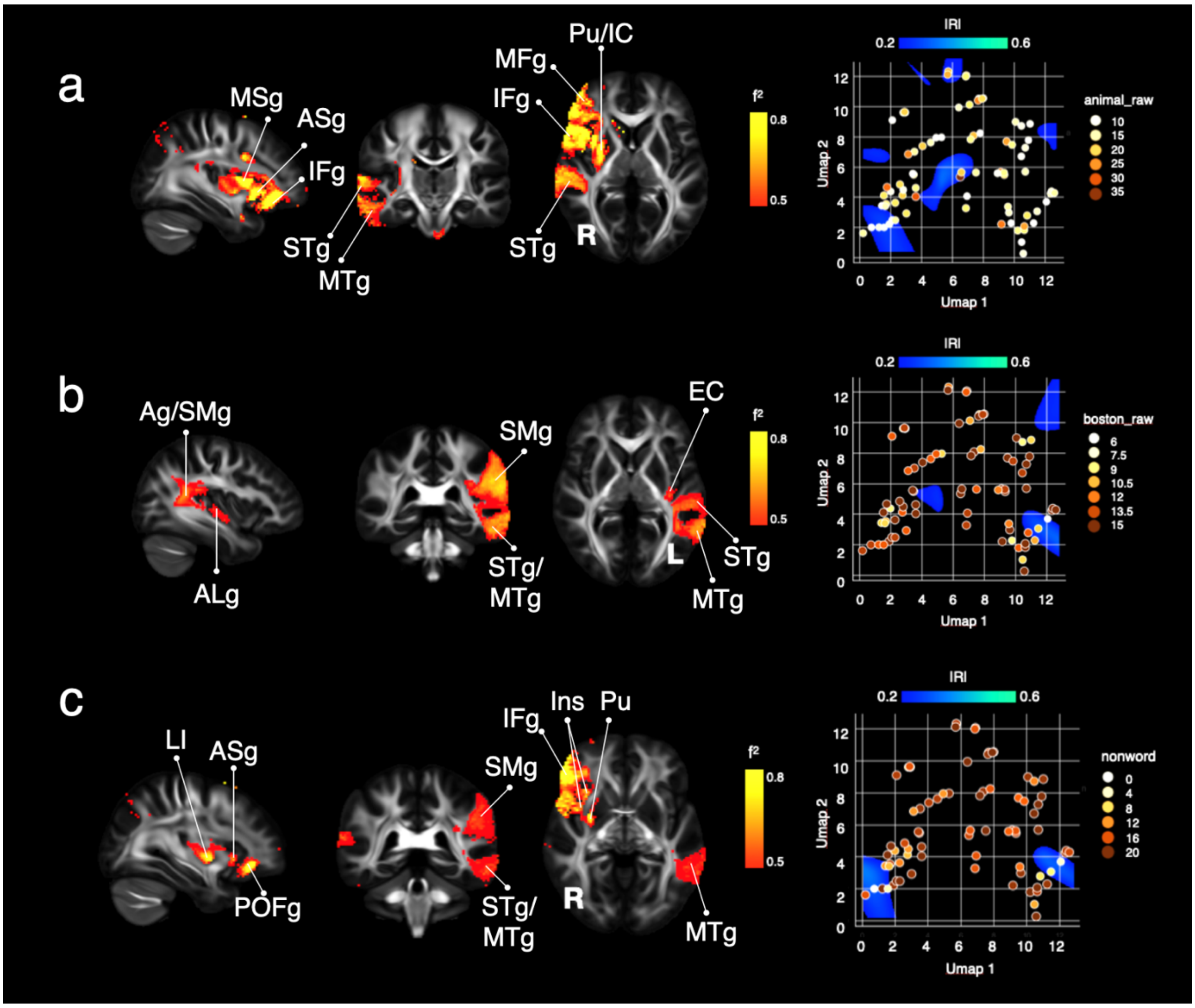

Supplementary Figure 19: Brain disconnections and UMAP related territories contributing significantly to semantic fluency (animals), the Boston Naming Test (BNT), and non-word reading. (a) 1-min animal fluency (animal_raw), (b) Boston Naming Test (boston_raw), and (c) non-word reading (nonword). Ag: Angular gyrus; ALg: Anterior Long insular gyrus; ASg: Anterior Short insular gyrus; EC: External/Extreme Capsule; IC: Internal Capsule; IFg: Inferior Frontal gyrus; Ins: Insula; LI: Limen Insulae; MSg: Midddle Short insular gyrus; MTg: Middle Temporal gyrus; POFg: Posterior Orbital Frontal gyrus; Pu: Putamen; SMg: Supra Marginal gyrus; STg: Superior Temporal gyrus. Maps are freely available at https://neurovault.org/collections/11260/ .

Reduced semantic fluency is associated with lesions to the left inferior frontal gyrus and insula, left medial temporal regions and the right inferior frontal gyrus and periventricular frontal white matter [107]. Disconnection patterns of the right middle and superior temporal gyri, right inferior frontal gyrus insular cortex (anterior and middle short gyri), and the right putamen/internal capsule predicted semantic fluency patterns.

BNT naming score impairments were predicted by disconnections of a temporal-parietal-insular network including the left hemisphere middle and superior temporal gyri, the inferior parietal lobe (angular and supramarginal gyrus), and the anterior long insular gyrus and external/extreme capsule. Patients with persistent naming deficits were shown to have disruptions to this network whereby lesions in the posterior superior temporal and inferior parietal cortex caused semantic paraphasias while lesions to the insula and putamen caused phonological paraphasias [108]. More recent studies also highlighted that lesions to the angular gyrus cause paraphasia that are unrelated to the BNT target item (e.g. not semantic or phonological paraphasia) [109]. In an attempt to isolate lexical-semantic retrieval from visual

recognition and motor speech elements involved in this picture naming task, Baldo et al. [110] identified the crucial role of the left mid-posterior middle temporal gyrus for naming errors on the BNT.

Nonword reading performance hinges on disconnections of the right hemisphere temporal-parietal-frontal network as well as the insula putamen. Previous research has highlighted this network as well where lesions to the middle and inferior frontal gyrus (pars opercularis and triangularis), insular cortex, central and parietal opercular cortex, and anterior middle temporal gyrus in addition to the precentral gyrus predicted reading deficits [111,112]. While these areas are most commonly reported for the left hemisphere, studies of right hemisphere patients have identified the same network albeit the flavour of linguistic errors when reading nonwords may be different [113].

C.3 Visuospatial attention

Visuospatial attention has been assessed along several dimensions, including visual search (i.e., cancellations tasks) and reaction time.

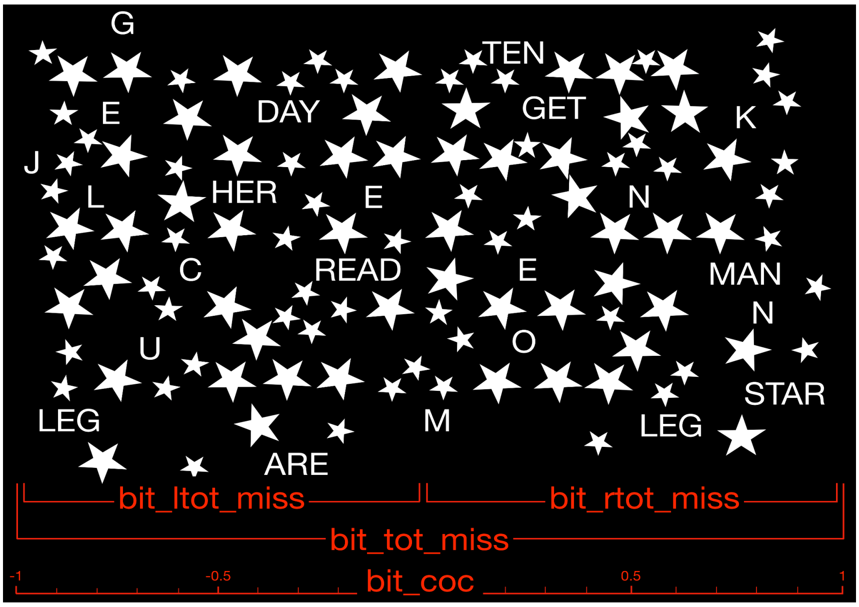

Supplementary Figure 20: Example of the Star cancellation test modified from (Wilson et al. 1987). bit_tot_miss: total missed stars; bit_ltot_miss: stars missed on the left; bit_rtot_miss: stars missed on the right; bit_coc: 'centre of mass' of the total cancellation.

Star cancellation is a paper and pencil visual search test originally proposed in and part of the Behavioral Inattention Test [114]. The patient has to circle all stars printed on a large A4 piece of paper. Stars are mixed with letters and words that the patient should not circle, as shown in an example of the star cancellation test in Supplementary Figure 20 derived from the original.

Total misses (bit_tot_miss), total left misses (bit_ltot_miss), and total right misses (bit_rtot_miss) scores can be derived from this test, with excellent reliability i.e. test-retest, [115]. Star cancellation also has a high sensibility and specificity for unilateral spatial neglect [116]. Additionally, the centre of cancellation (bit_coc) corresponding to the 'centre of mass' of the cancellation can also be calculated [117].

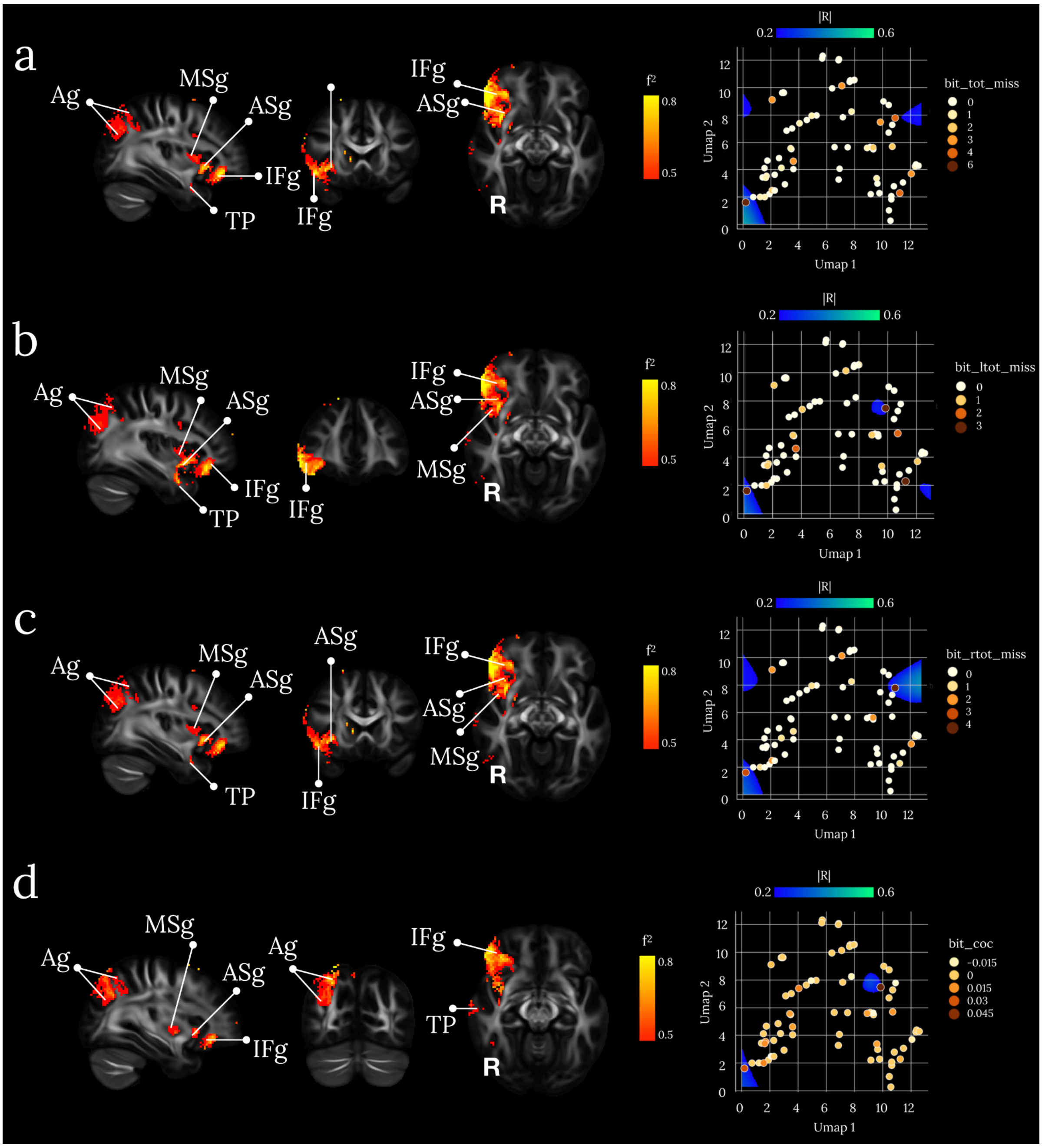

Supplementary Figure 21: Brain disconnections and UMAP related territories contributing significantly to the Star cancellation test. (a) Total misses (bit_tot_miss), (b) total left misses (bit_ltot_miss), (c) total right misses (bit_rtot_miss) scores and (d) the centre of cancellation (bit_coc). Ag: Angular gyrus; ASg: Anterior Short insular gyrus; IFg: Inferior Frontal gyrus; MSg: Midddle Short insular gyrus; TP: Temporal Pole. Maps are freely available at https://neurovault.org/collections/11260/
.

Profiles of disconnections predicting individual total misses (bit_tot_miss), total left misses (bit_ltot_miss), total right misses (bit_rtot_miss) and the centre of cancellation (bit_coc) clustered together in comparable areas in the UMAP morphospace with large effect size. These clusters correspond either to a significant disconnection the right inferior frontal gyrus from the anterior and middle short gyri of the insula via fronto-insular tracts [118,119] and the temporal pole via the uncinate fasciculus [120]. Local disconnection of the angular gyrus [121] was also significant. The role of the inferior frontal gyrus in cancellations tasks such as the star cancellation has been widely documented in the context of unilateral visual neglect [122-124] and discussed with regards to its contribution to visual search in the presence of distractors [125]. The insula has also been reported as a critical area leading to unilateral

visual neglect [126] due to its contribution to the integration between extrapersonnal stimuli and internal milieu and is typically inactivated when attention lapses [127,128]. Additionally, lesions to the temporal pole have been significantly associated with a significant drop in cancellation task performance [129,130] because of its contribution to the visual ventral stream and the conscious identification of the target amongst distractors. Finally, the angular gyrus, when damaged, have also been reported as critical to the performance in cancellation tasks [131] due to its role in spatial attention [132].
The disruption of the circuitry constituted by the frontoinsular tracts, the uncinate and local parietal connections elegantly reconcile these four theories explaining the pathophysiological mechanisms related to cancellation tasks performance and unilateral visual neglect.

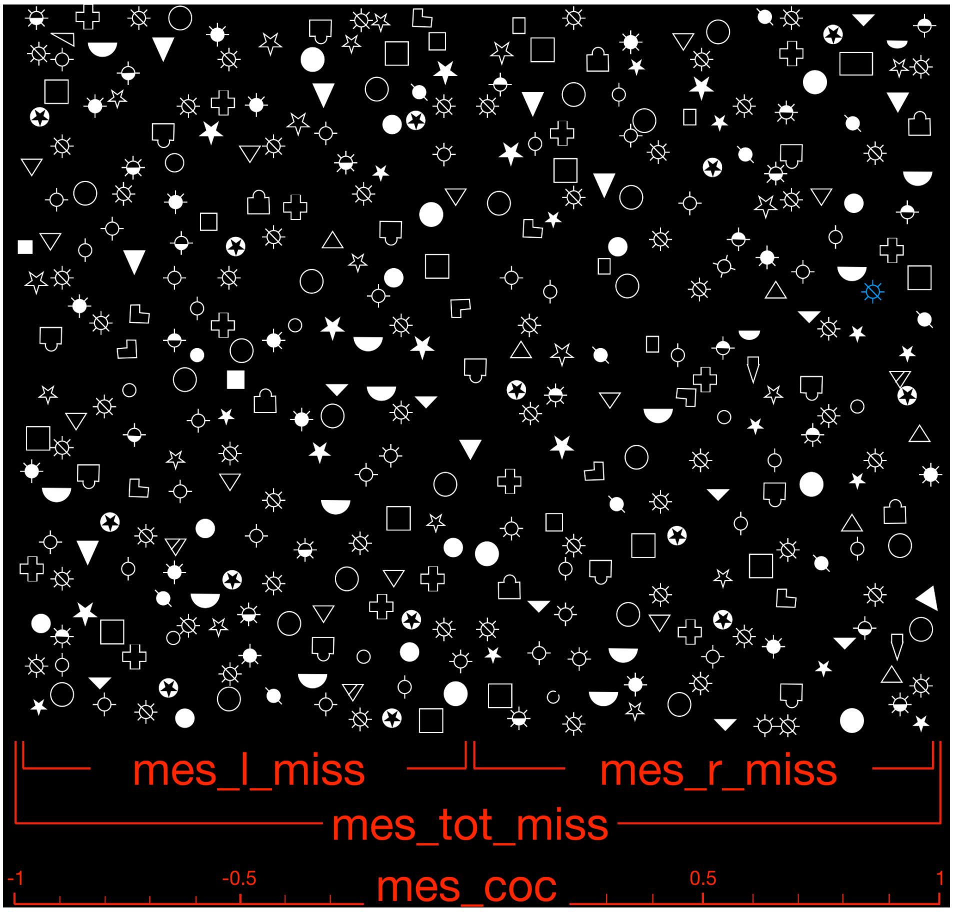

Supplementary Figure 22: Example of the Mesulam Unstructured Symbol Cancellation Test modified from [133]. The item in blue is an example of the target to be cancelled by the patients. mes_tot_miss: total missed stars; mes_ltot_miss: stars missed on the left; mes_rtot_miss: stars missed on the right; mes_coc: 'centre of mass' of the total cancellation.

The Mesulam Unstructured Symbol Cancellation Test is a paper and pencil visual search test. Patients are asked to bar the full cancel suns as the one coloured in blue in supplementary figure 21 derived from [133] amongst distractors. Compared to the star cancellation task, the higher number of distractors and the use of feature conjunctions [134] in the Mesulam Unstructured Symbol Cancellation Test makes it a more challenging task to achieve in the clinic.
Four metrics can be derived from the Mesulam Unstructured Symbol Cancellation Test: Total misses (mes_tot_miss), total left misses (mes_l_miss), total right misses (mes_r_miss), and centre of cancellation (mes_coc) corresponding to the 'centre of mass' of the cancellation [117].

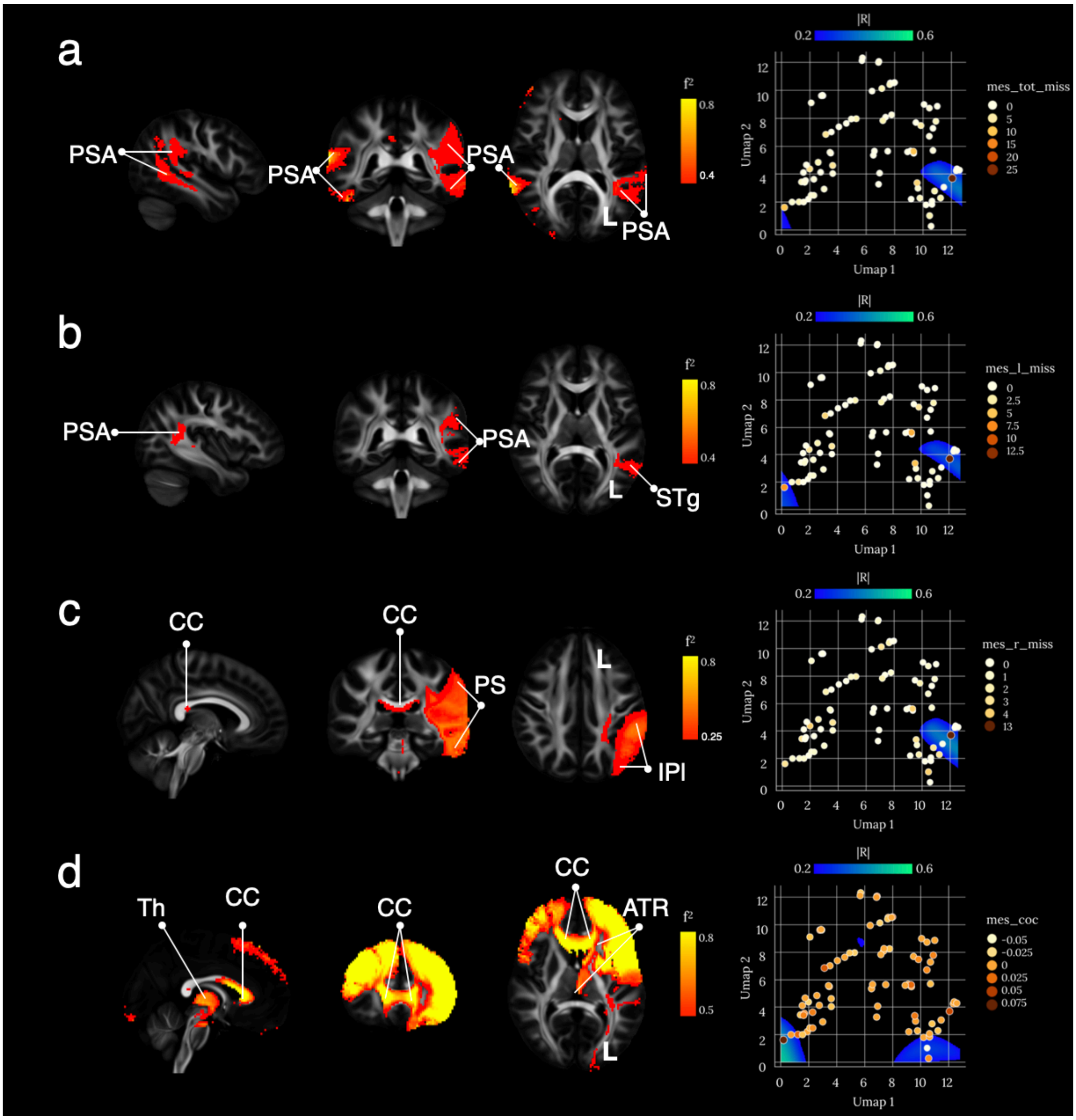

Supplementary Figure 23: Brain disconnections and UMAP related territories contributing significantly to the Mesulam Unstructured Symbol Cancellation Test. (a) Total misses (mes_tot_miss), (b) total left misses (mes_l_miss), (c) total right misses (mes_r_miss) scores and (d) centre of cancellation (mes_coc). ATR: Anterior Thalamic Radiation; CC: Corpus Callosum; IPl: Inferior Parietal lobe; PSA: Posterior Segment of the Arcuate fasciculus; Th: Thalamus. Maps are freely available at https://neurovault.org/collections/11260/ .

The profiles of disconnections associated with the number of targets missed, whether on the left, right or in total, clustered together in comparable areas in the UMAP morphospace with large effect size ($0.35>f^2$). These profiles mostly involved the temporoparietal circuits bilaterally (posterior segment of the arcuate fasciculus) and their interhemispheric callosal connections. Of interest, the disconnection of the left hemisphere for left and right misses was more prominent statistically than the right hemisphere. In contrast, the centre of mass of the task was also well predicted but by different related territories in the UMAP morphospace, also characterised by interhemispheric connections, but this time in the frontal lobe. A diencephalic disconnection via the anterior thalamic radiations was also significant.
The temporo-parietal disconnection fits with previous theories suggesting that visual awareness deficits would be related to a disconnection between the visual ventral stream and the global workspace [135].

Such disconnection would prevent preprocessed visual information i.e. the visual ventral stream, [136,137] to access conscious manipulation (i.e. the global workspace; [68,138,139] indispensable to compare each target mentally to the model during the complex visual search as the Mesulam Unstructured Symbol Cancellation Test.
On the other hand, the callosal disconnection fits with Norman Geschwind original theory [140] suggesting neglected items result from the disconnection of right hemisphere-based knowledge from the left hemisphere. In so doing, the absence of mental verbalisation of right hemisphere-based visual knowledge would prevent their conscious representation [141] and lead to targets' omissions. Of note, the integrity of the corpus callosum also contributes significantly to the recovery of visuospatial neglect [142] and might therefore have contributed to the visual search scores of the chronic patients reported in our study.
Finally, lesions to the thalamus have also been documented to contribute to visual search deficits and hemispatial visual neglect through an interruption of the cortico-subcortical loops critical to the proper function of the cortex [143-157]. Hence, through the capture of high dimensional interactions, our method revealed the poly-origin mechanisms leading to a decreased performance at the Mesulam Unstructured Symbol Cancellation Test in patients and reconciled the inter- with interhemispheric theories of hemispatial neglect.

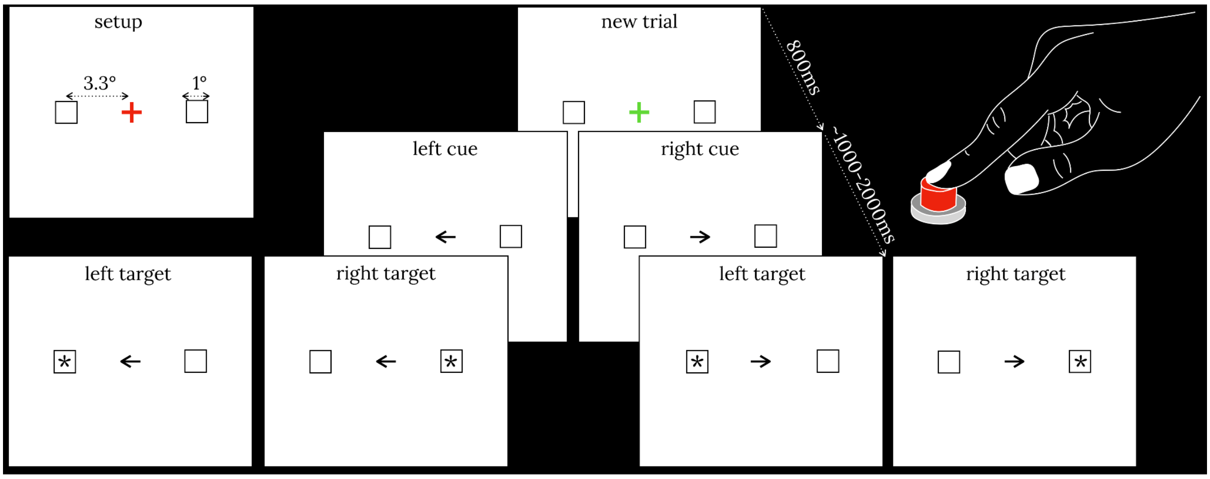

Supplementary Figure 24: Example of the Posner orienting task modified from [151]. The arrow (i.e., the cue) is presented for 2360ms. The asterisk (i.e., the target) is presented for 300ms.

The Posner orienting task is a detection paradigm initially designed by Posner [151]. The paradigm is computerised and consists of two square frames (1° size) placed on the left and the right of a central fixation (at 3.3° of eccentricity). Trials start when the central fixation turns from red to green. After 800ms, an arrow points left or right. 1000 to 2000ms later, an asterisk appears in one of the two square frames, 75% of the time at the location indicated by the cue (valid trials). In the other 25% the asterisk appears in the opposite location (invalid trials). Patients are required to press a button when an asterisk appears on the screen with their ipsilesional hand. A pause of 2360 ms separates each trial. Each patient performed 120 trials divided into one practice block and two test blocks (see supplementary Figure 24 for a Posner orienting task illustration).

Twenty-four metrics can be derived from the Posner orienting task and can be divided into four categories.

Accuracy metrics correspond the percentage of target missed in average (pos_acc_avg), left (pos_acc_lv) and right (pos_acc_rv) valid trials and in left (pos_acc_li) and right (pos_acc_ri) valid trials.

The effect of validity in accuracy (pos_acc_validity) can subsequently be assessed by subtracting the percentage of target missed in valid conditions (i.e., pos_acc_lv and pos_acc_rv) from invalid conditions (i.e., pos_acc_li and pos_acc_ri) divided by 2.
The effect of disengagement in accuracy (pos_acc_disengage) can also be assessed by subtracting the percentage of target missed in valid trials from invalid trials for left (pos_acc_lv - pos_acc_li) and the right (pos_acc_rv - pos_acc_ri) asterisks separately. The result of the right subtraction is further subtracted from the left and divided by 2 to obtain the accuracy's disengagement effect.

Reaction time metrics correspond to the time in milliseconds between the asterisk and pressing of the button in average (pos_rt_avg), left (pos_rt_lv), and right (pos_rt_rv) valid trials and in left (pos_rt_li) and right (pos_rt_ri) invalid trials.
As for accuracy metrics, the effect of validity in reaction time (pos_rt_validity) can subsequently be assessed by subtracting the average reaction time for valid conditions (i.e., pos_rt_lv and pos_rt_rv) from the average reaction time for invalid conditions (i.e. pos_rt_li and pos_rt_ri) divided by 2.
Similarly, the effect of disengagement in reaction time (pos_rt_disengage) can also be assessed by subtracting the average reaction time for valid trials from the average reaction time for invalid trials for left (pos_acc_lv - pos_acc_li) and the right (pos_acc_rv - pos_acc_ri) asterisks separately. The result of the right subtraction is further subtracted from the left and divided by 2 to obtain the reaction time's disengagement effect.

Subbing metrics are a statistical refinement of the reaction times metrics presented above. For these metrics, two standard deviations above or under the average reaction time are discarded from the analysis for precision leading to corresponding new metrics for average (pos_suv_avg), left (pos_sub_lv), and right (pos_sub_rv) valid trials, left (pos_sub_li) and right (pos_sub_ri) invalid trials and equivalent assessments of the effect of validity (pos_sub_validity) and disengagement (pos_sub_disengage) in 'subbed' reaction times.

Finally, the visual field metric (pos_acc_vf, pos_rt_vf, pos_sub_vf) corresponds to the asymmetry in accuracy or reaction time between the left and the right visual fields. It can be calculated by subtracting the accuracy or reaction times for right asterisks from the accuracy or reaction times for left asterisks divided by 2.

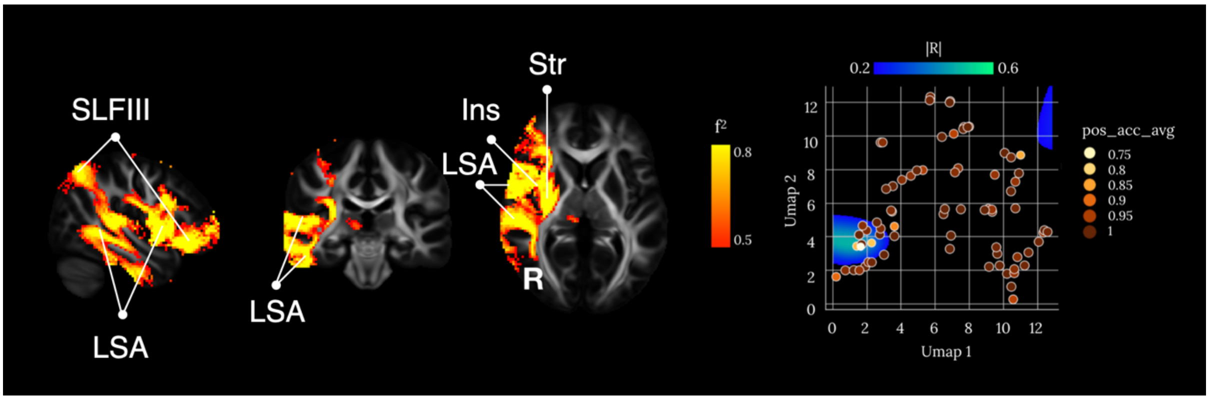

Supplementary Figure 25: Brain disconnections and UMAP related territories contributing significantly to the average accuracy (pos_acc_avg) in the Posner orienting task. Ins: Insula; LSA: Long Segment of the Arcuate fasciculus; SLFIII: third branch of the Superior Longitudinal Fasciculus; Str: Striatum. Maps are freely available at https://neurovault.org/collections/11260/ .

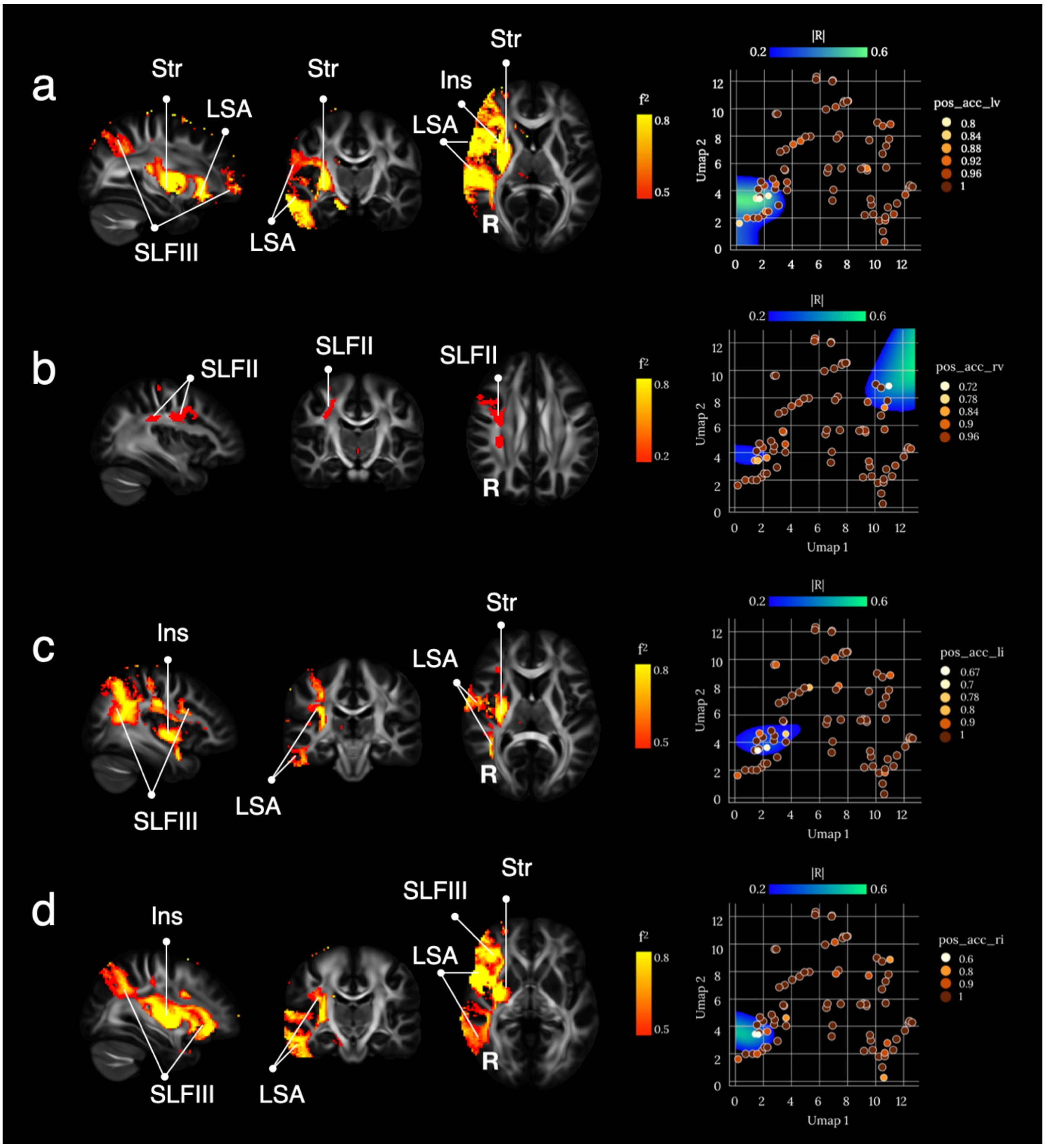

Supplementary Figure 26: Brain disconnections and UMAP related territories contributing significantly to the accuracy for the (a) left valid, (b) right valid, (a) left invalid and (b) right invalid asterisks in the Posner orienting task. Ins: Insula; LSA: Long Segment of the Arcuate fasciculus; SLFII: second branch of the Superior Longitudinal Fasciculus; SLFIII: third branch of the Superior Longitudinal Fasciculus; Str: Striatum. Maps are freely available at https://neurovault.org/collections/11260/ .

The profiles of disconnections associated with the accuracy at the Posner orienting task clustered together in comparable areas in the UMAP morphospace with large effect size ($0.35<f^2$) for all conditions except for the percentage of right valid asterisks (see supplementary Figure 26b).
Disconnections leading to reduced accuracy systematically involved the ventral portion of the superior longitudinal fasciculus mostly the SLF III and the SLF II in some conditions see [158], the long segment of the arcuate fasciculus, the insula and the striatum in the right hemisphere.
The poor results for the right valid target condition might be related to the reduced number of omissions occurring on the cued right hemifield for patients one year after their stroke that limited the variance of the data.

The disconnection of the ventral superior longitudinal fasciculus together with the arcuate fasciculus in the right hemisphere, which connects the core areas of the ventral attention system [68,159], is in line with previous work reporting that hemispatial neglect and, therefore, the percentage of missed targets would be related to a disconnection of the ventral attention network [122,160,161]. Of importance, the insula is 'at the heart' [162,163] of the ventral attention system and was significantly disconnected in all conditions, except for the right valid asterisks for the reasons aforementioned.

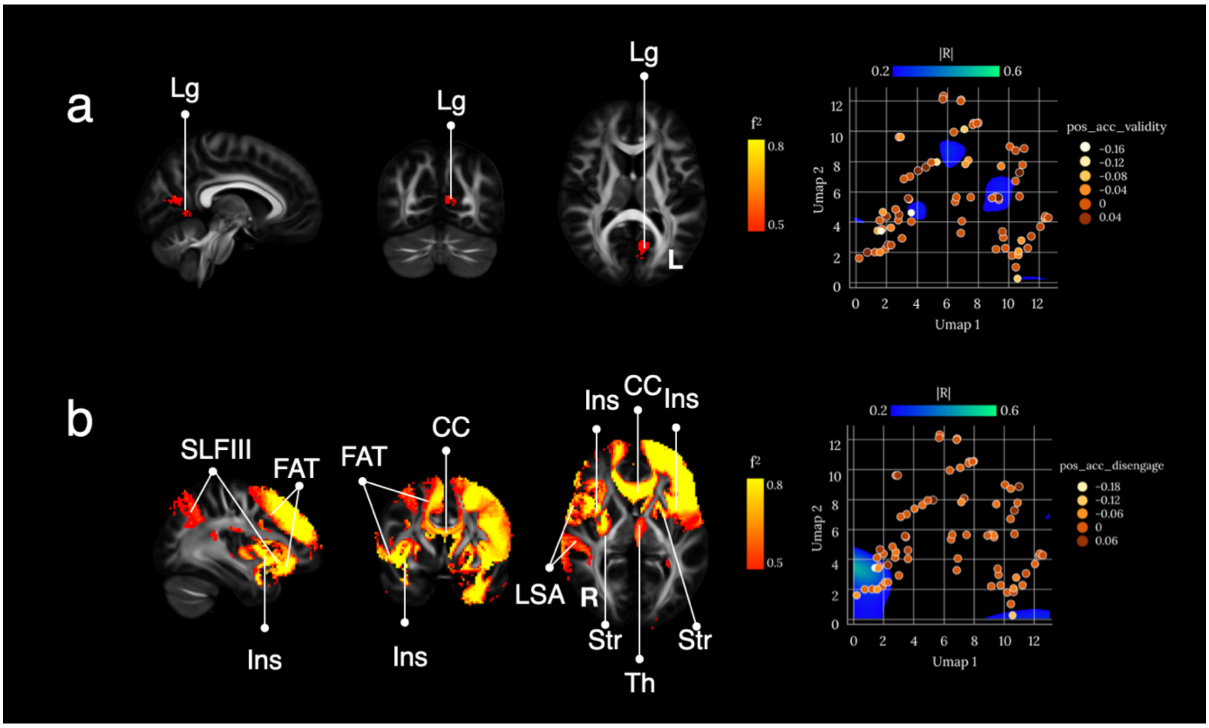

Supplementary Figure 27: Brain disconnections and UMAP related territories contributing significantly to accuracy for the (a) validity and (b) disengage conditions in the Posner orienting task FAT: Frontal Aslant Tract; Ins: Insula; Lg: Lingual gyrus; LSA: Long Segment of the Arcuate fasciculus; SLFIII: third branch of the Superior Longitudinal Fasciculus; Str: Striatum; Th: Thalamus. Maps are freely available at https://neurovault.org/collections/11260/ .

The profiles of disconnections associated with the percentage of asterisks accurately detected when cued (pos_acc_validity) clustered together in comparable areas in the UMAP morphospace with a large effect size (see supplementary Figure 25 and 26) and were associated with disconnection of the lingual gyrus in the left hemisphere (Supplementary Figure 27a). Accuracy for cued asterisks relies partially on dorsal, voluntary orienting of attention [159]. While the lingual gyrus is surprising, previous work reported a significant involvement of the visual areas in the dorsal attention system with decreased activity related to hemispatial neglect and recovery from visual neglect with the re-establishment of this activity [122]. Additionally, the same regions have been reported as damaged after a stroke in the posterior cerebral artery leading again to signs of hemispatial neglect [164]. Hence, since rehabilitation strategies are mainly focused on the use of the voluntary orienting of attention [165] our results suggest that patients may recover with more difficulties from their visual neglect through an early disruption of the dorsal attention network with the disconnection of the lingual gyrus. Future longitudinal analyses exploring the neural bases of visual neglect recovery after a posterior cerebral artery stroke may demonstrate this point further.

The profile of disconnections related to percentage of asterisks accurately detected despite the wrong cue (pos_acc_disengage) clustered together in comparable areas in the UMAP morphospace with a

large effect size (see supplementary Figure 27b) and were involved the same network as for the average accuracy including ventral portion of the superior longitudinal fasciculus, the long segment of the arcuate fasciculus, the insula and the striatum.
In addition, the anterior portion of the corpus callosum together with the frontal aslant tract and the anterior thalamus were involved.
Pos_acc_disengage requires a reorienting of attention that relies on the same structure as the average accuracy condition but also requires an inhibition to perform the reorientation accurately instead of perseverating on the cued target. Simply said, the disengage condition would require additional regions related to inhibition mechanisms compared to the other conditions (i.e., pos_acc_avg, pos_acc_lv, pos_acc_rv, pos_acc_li and pos_acc_ri).
Typically the corpus callosum connectivity between the two hemispheres is critical to the coordination of two hemibodies [166-170] through hypothesised inhibitory mechanisms of one hemisphere onto the other [171]. Accordingly, animal studies exploring the effect of callosal disconnection with frontal lobe damage reveal significant mistakes in the selection paradigm during which monkeys must choose the correct target [172]. Similarly split brain patients are less successful than controls are bilateral visual tasks [173] comparable to the Posner orienting task. Direct electrical stimulation [78] abnormal connectivity [174] of the frontal aslant tract, or damage to its projections [175] have also been associated with deficits in the inhibitory control of behaviour. Finally anterior thalamic lesions in rats [176] and anterior thalamic radiation damage in humans [177] abolish inhibitory control.
Hence the circuitry reported for the pos_acc_disengage condition is concordant with the current literature in the orientation of attention in the inhibitory control.

Since subbed reaction times are just a refinement of raw reaction time and results are mostly similar between the two conditions, we only report below the subbed condition. However, raw reaction time results are available online (https://neurovault.org/collections/11260/).

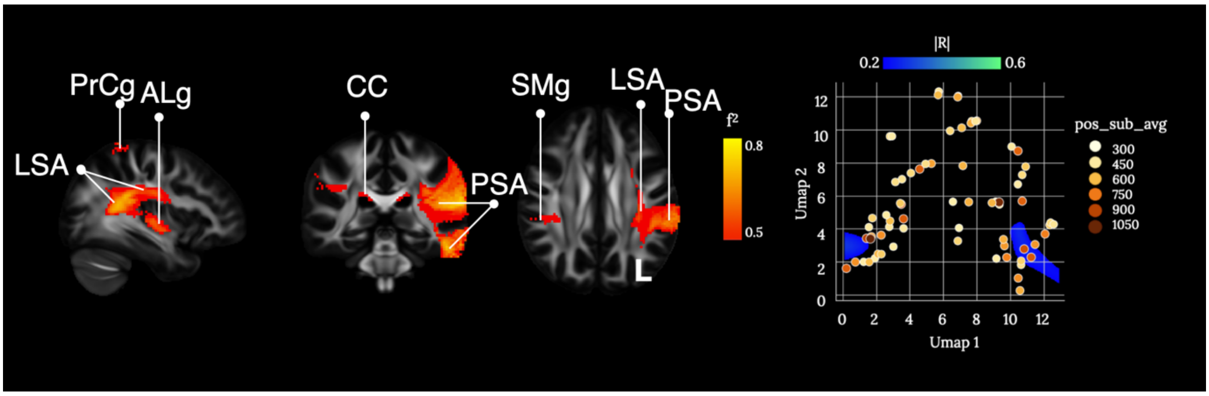

Supplementary Figure 28: Brain disconnections and UMAP related territories contributing significantly to 'subbed' average reaction times in the Posner orienting task. ALg: Anterior Long insular gyrus; CC: Corpus Callosum; LSA: Long Segment of the Arcuate fasciculus; PrCg: Pre-Central gyrus; PSA: Posterior Segment of the Arcuate fasciculus; SMg: Supra Marginal gyrus. Maps are freely available at https://neurovault.org/collections/11260/.

The profiles of disconnections related to the 'subbed' average reaction time at the Posner orienting task clustered together in comparable areas in the UMAP morphospace with a large effect size (see supplementary Figure 28).
These disconnections involved a circuit composed of the left motor cortex, the long segment, and the posterior segment of the arcuate fasciculus in the left hemisphere together with the corpus callosum connections to the right supra marginal gyrus.

While motor disconnections might have an obvious link with the speed of reaction time, the language network represented by the two branches of the arcuate fasciculus reported here might play an important role in the symbol recognition necessary to read [88,178] and interpret the arrow in the left hemisphere [179]. Communication to the right hemisphere via the corpus callosum would be necessary when attention is required to be oriented toward the left.

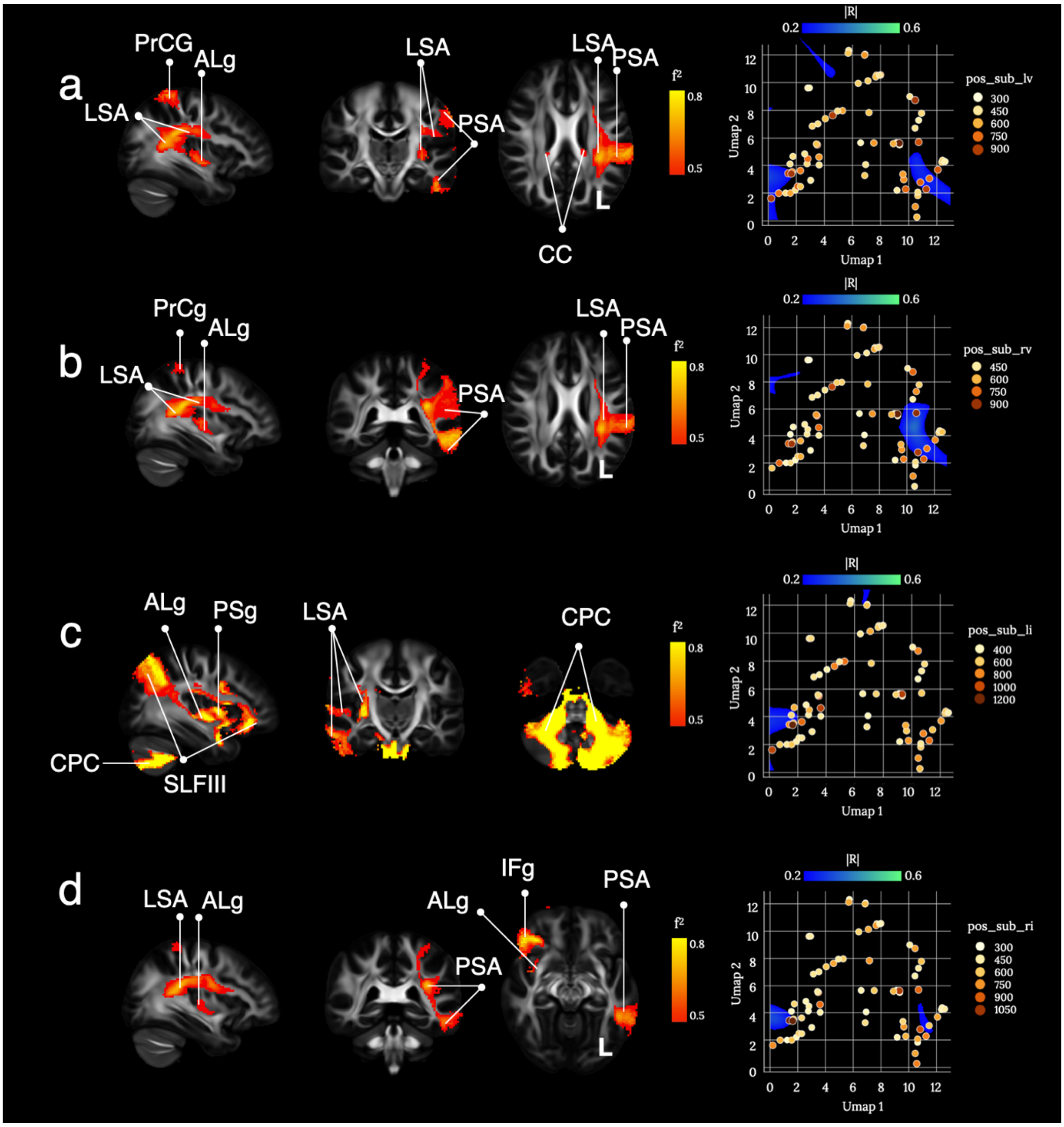

Supplementary Figure 29: Brain disconnections and UMAP related territories contributing significantly to ‘subbed’ reaction times for the (a) left valid, (b) right valid, (a) left invalid and (b) right invalid asterisks in the The Posner orienting task. ALg: Anterior Long insular gyrus; CC: Corpus Callosum; CPC: Cortico-Ponto-Cerebellar tract; LSA: Long Segment of the Arcuate fasciculus; PrCg: Pre-Central gyrus; PSA: Posterior Segment of the Arcuate fasciculus; SMg: Supra Marginal gyrus. Maps are freely available at https://neurovault.org/collections/11260/ .

The profiles of disconnections related to the ‘subbed’ reaction time at the Posner orienting task clustered together in comparable areas in the UMAP morphospace with a large effect size (see supplementary Figure 29a and 29b) for all valid conditions. The same network was involved as for the ‘subbed’ average

reaction time with the exception of the missing involvement of the corpus callosum when the valid target appears on the right as predicted above. In that case, when the target appears on the right most of the processing (interpretation of the target and spatial orientation of the attention) can happen in the left hemisphere compatible with the absence of involvement of the corpus callosum.
For the invalid condition, the reorienting of attention toward the left involved a large network of areas including the cerebellum via the cortico-ponto-cerebellar tract, the insula, the arcuate and the third branch of the superior longitudinal fasciculus in the right hemisphere (see supplementary Figure 29c). For the reorientation of attention toward the left, the network was comparable to all valid conditions with the exception of the involvement of the right inferior frontal gyrus (see supplementary Figure 29d). The involvement of the right inferior frontal gyrus in the left and the right invalid conditions is compatible with the inhibitory requirement of the task, activation and deficit of inhibition have been reported respectively in controls functional MRI [180,181] and in patients behaviour after a lesion in the right inferior frontal gyrus [182]. However, the involvement of the cerebellum in the reorienting of attention toward the left was atypical of any of the other conditions. While left hemineglect has been reported after a lesion of the cerebellum [183,184], whether cerebellar neglect is more specifically affecting the reorientation of attention toward the left and no other conditions of the Posner orienting task awaits for future demonstration. As mentioned previously, the insula is central [162,163] to the ventral attention system that is activated mainly during the reorienting of attention [159].

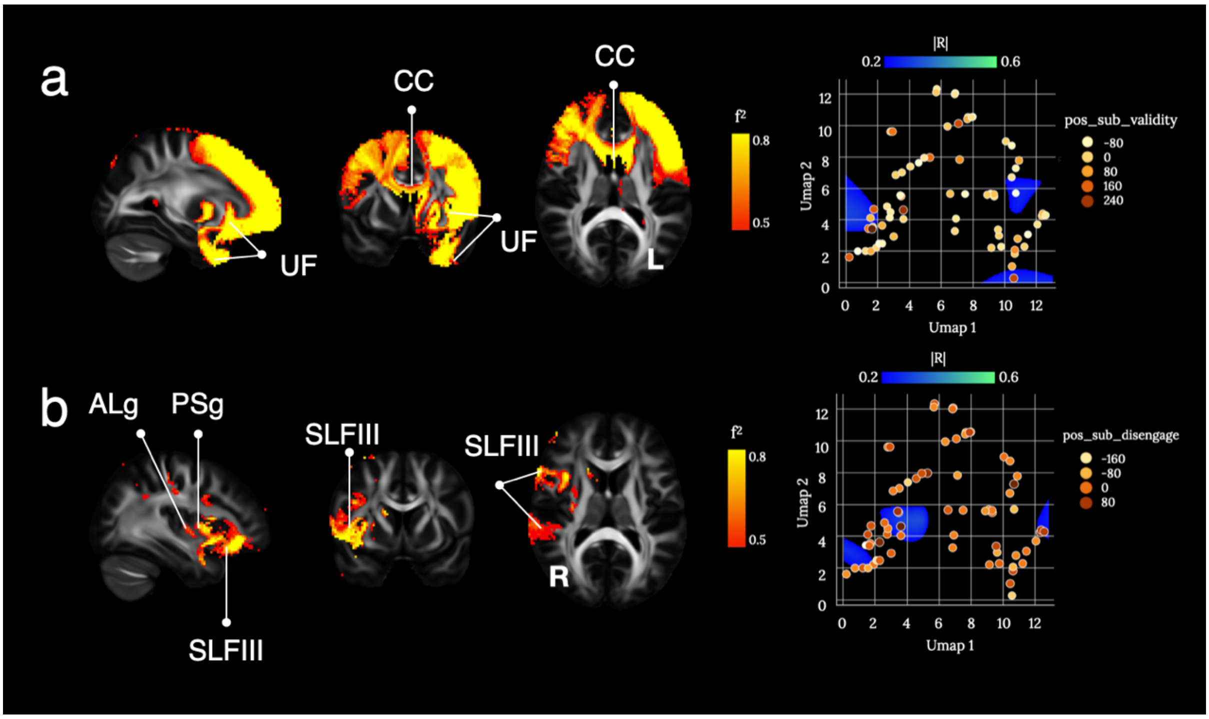

Supplementary Figure 30: Brain disconnections and UMAP related territories contributing significantly to 'subbed' average reaction times for the (a) validity and (b) disengage conditions in the The Posner orienting task. ALg: Anterior Long insular gyrus; CC: Corpus Callosum; PSg: Posterior Short insular gyrus ; SLFIII: third branch of the Superior Longitudinal Fasciculus; UF: Uncinate Fasciculus. Maps are freely available at https://neurovault.org/collections/11260/ .

The profiles of disconnections related to the 'subbed' reaction time at the Posner orienting task for the validity condition clustered together in comparable areas in the UMAP morphospace with a large effect size (see supplementary Figure 30a) revealing a large frontal network supported by the corpus callosum and the uncinate fasciculus.

The validity contusion specifically assesses the voluntary orienting of attention with no preference for the side of appearance of the asterisks. The important role of the frontal lobe in the voluntary orientation of attention have previously been demonstrated in patients with brain lesions [185] and fits quite elegantly with the pattern of results observed here. The role of the uncinate fasciculus is somewhat less clear with eventual contribution to rule learning required for the optimal achievement of the task [186].
In contrast the disconnections related to the disengage condition involved the typical ventral fronto-parietal network supported by the third branch of the superior longitudinal fasciculus in the right hemisphere [68,159] and including the insula [162,163] (see supplementary Figure 30b).

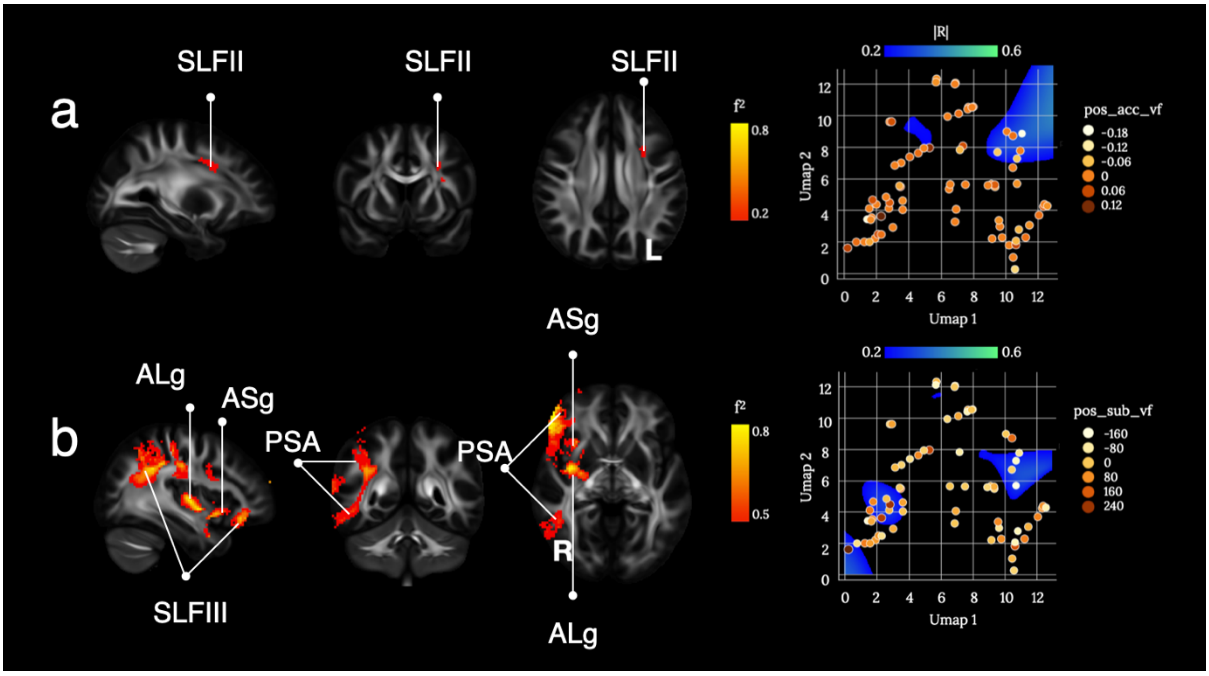

Supplementary Figure 31: Brain disconnections and UMAP related territories contributing significantly to asymmetries in the visual fields in terms of (a) accuracy, (b) reaction time in the Posner orienting task. ALg: Anterior Long insular gyrus; ASg: Anterior Short insular gyrus; PSA: Posterior Segment of the Arcuate fasciculus; SLFII: second branch of the Superior Longitudinal Fasciculus; SLFIII: third branch of the Superior Longitudinal Fasciculus. Maps are freely available at https://neurovault.org/collections/11260/ .

The profiles of disconnections related to the visual field asymmetries at the Posner orienting task clustered together in different areas of the UMAP morphospace according to whether accuracy (see supplementary Figure 31a) or 'subbed' reaction time (see supplementary Figure 31b) were measured.
With regard to accuracy the single involvement of a blob located onto the second branch of left superior longitudinal fasciculus appeared to contribute significantly to the asymmetry in the number of asterisks reported in the left and the right visual field. The patient did not report the asterisk when they did not see it.
While the second branch of the superior longitudinal fasciculus has been recently suggested to support conscious processing [68,139,187] the level to which this process would be preferentially lateralized in the left hemisphere waits for further validation.
In contrast the speed to which targets were detected was particularly asymmetrical after a disconnection of the typical ventral fronto-parietal network [159] supported by the SLF III [68] and involving the insula [162,163]. This result might have been driven by the extra time patients with a lesion in the SLF III might take to process targets requiring their reorientation toward the left.

Supplementary references

1. Addis DR, Moloney EEJ, Tippett LJ, P. Roberts R, Hach S. Characterizing cerebellar activity during autobiographical memory retrieval: ALE and functional connectivity investigations. *Neuropsychologia*. 2016/09/01/ 2016;90:80-93. doi:https://doi.org/10.1016/j.neuropsychologia.2016.05.025
2. Lyle RC. A performance test for assessment of upper limb function in physical rehabilitation treatment and research. *Int J Rehabil Res*. 1981;4(4):483-92. doi:10.1097/00004356-198112000-00001
3. Demeurisse G, Demol O, Robaye E. Motor evaluation in vascular hemiplegia. *Eur Neurol*. 1980;19(6):382-9. doi:10.1159/000115178
4. Oxford Grice K, Vogel KA, Le V, Mitchell A, Muniz S, Vollmer MA. Adult norms for a commercially available Nine Hole Peg Test for finger dexterity. *Am J Occup Ther*. Sep-Oct 2003;57(5):570-3. doi:10.5014/ajot.57.5.570
5. Dreeben-Irimia O. *Physical Therapy Clinical Handbook for PTAs*. Jones & Bartlett Publishers; 2008.
6. Fess E, Moran C. *American Society of Hand Therapists Clinical Assessment Recommendations*. 1981.
7. Backman C, Gibson SCD, Parsons J. Assessment of Hand Function: The Relationship between Pegboard Dexterity and Applied Dexterity. *Canadian Journal of Occupational Therapy*. 1992/10/01 1992;59(4):208-213. doi:10.1177/000841749205900406
8. Kellor M, Frost J, Silberberg N, Iversen I, Cummings R. Hand strength and dexterity. *Am J Occup Ther*. Mar 1971;25(2):77-83.
9. Mathiowetz V, Volland G, Kashman N, Weber K. Adult norms for the Box and Block Test of manual dexterity. *Am J Occup Ther*. Jun 1985;39(6):386-91. doi:10.5014/ajot.39.6.386
10. Corbetta M, Ramsey L, Callejas A*, et al*. Common behavioral clusters and subcortical anatomy in stroke. *Neuron*. Mar 4 2015;85(5):927-41. doi:10.1016/j.neuron.2015.02.027
11. Wade DT, Wood VA, Heller A, Maggs J, Langton Hewer R. Walking after stroke. Measurement and recovery over the first 3 months. *Scand J Rehabil Med*. 1987;19(1):25-30.
12. Keith RA, Granger CV, Hamilton BB, Sherwin FS. The functional independence measure: a new tool for rehabilitation. *Adv Clin Rehabil*. 1987;1:6-18.
13. Nowak DA, Hermsdorfer J, Topka H. Deficits of predictive grip force control during object manipulation in acute stroke. *J Neurol*. Jul 2003;250(7):850-60. doi:10.1007/s00415-003-1095-z
14. Begliomini C, Sartori L, Miotto D, Stramare R, Motta R, Castiello U. Exploring manual asymmetries during grasping: a dynamic causal modeling approach. *Front Psychol*. 2015;6:167. doi:10.3389/fpsyg.2015.00167
15. Davare M, Andres M, Clerget E, Thonnard JL, Olivier E. Temporal dissociation between hand shaping and grip force scaling in the anterior intraparietal area. *J Neurosci*. Apr 11 2007;27(15):3974-80. doi:10.1523/JNEUROSCI.0426-07.2007
16. Howells H, Thiebaut de Schotten M, Dell'Acqua F*, et al*. Frontoparietal Tracts Linked to Lateralized Hand Preference and Manual Specialization. *Cereb Cortex*. Jul 1 2018;28(7):2482-2494. doi:10.1093/cercor/bhy040
17. Howells H, Simone L, Borra E, Fornia L, Cerri G, Luppino G. Reproducing macaque lateral grasping and oculomotor networks using resting state functional connectivity and diffusion tractography. *Brain Structure and Function*. 2020/11/01 2020;225(8):2533-2551. doi:10.1007/s00429-020-02142-2
18. Budisavljevic S, Dell'Acqua F, Zanatto D*, et al*. Asymmetry and Structure of the Fronto-Parietal Networks Underlie Visuomotor Processing in Humans. *Cereb Cortex*. Feb 1 2017;27(2):1532-1544. doi:10.1093/cercor/bhv348

19. Desmurget M, Sirigu A. A parietal-premotor network for movement intention and motor awareness. *Trends Cogn Sci*. Oct 2009;13(10):411-9. doi:10.1016/j.tics.2009.08.001
20. Sirigu A, Daprati E, Ciancia S*, et al*. Altered awareness of voluntary action after damage to the parietal cortex. *Nat Neurosci*. Jan 2004;7(1):80-4. doi:10.1038/nn1160
21. Berti A, Bottini G, Gandola M*, et al*. Shared cortical anatomy for motor awareness and motor control. *Science*. Jul 15 2005;309(5733):488-91. doi:10.1126/science.1110625
22. Karnath HO, Baier B, Nagele T. Awareness of the functioning of one's own limbs mediated by the insular cortex? *J Neurosci*. Aug 3 2005;25(31):7134-8. doi:10.1523/JNEUROSCI.1590-05.2005
23. Baier B, Karnath HO. Tight link between our sense of limb ownership and self-awareness of actions. *Stroke*. Feb 2008;39(2):486-8. doi:10.1161/STROKEAHA.107.495606
24. Pacella V, Foulon C, Jenkinson PM*, et al*. Anosognosia for hemiplegia as a tripartite disconnection syndrome. *Elife*. Aug 6 2019;8doi:10.7554/eLife.46075
25. Jenkinson PM, Papadaki C, Besharati S*, et al*. Welcoming back my arm: affective touch increases body ownership following right-hemisphere stroke. *Brain Commun*. 2020;2(1):fcaa034. doi:10.1093/braincomms/fcaa034
26. Besharati S, Forkel SJ, Kopelman M, Solms M, Jenkinson PM, Fotopoulou A. The affective modulation of motor awareness in anosognosia for hemiplegia: behavioural and lesion evidence. *Cortex*. Dec 2014;61:127-40. doi:10.1016/j.cortex.2014.08.016
27. Gandola M, Invernizzi P, Sedda A*, et al*. An anatomical account of somatoparaphrenia. *Cortex*. Oct 2012;48(9):1165-78. doi:10.1016/j.cortex.2011.06.012
28. Invernizzi P, Gandola M, Romano D, Zapparoli L, Bottini G, Paulesu E. What is mine? Behavioral and anatomical dissociations between somatoparaphrenia and anosognosia for hemiplegia. *Behav Neurol*. 2013;26(1-2):139-50. doi:10.3233/BEN-2012-110226
29. Moro V, Pacella V, Scandola M*, et al*. A fronto-insular-parietal network for the sense of body ownership. *Cereb Cortex*. Mar 2 2022;doi:10.1093/cercor/bhac081
30. Jenkinson PM, Preston C, Ellis SJ. Unawareness after stroke: a review and practical guide to understanding, assessing, and managing anosognosia for hemiplegia. *J Clin Exp Neuropsychol*. Dec 2011;33(10):1079-93. doi:10.1080/13803395.2011.596822
31. Romano D, Maravita A. The dynamic nature of the sense of ownership after brain injury. Clues from asomatognosia and somatoparaphrenia. *Neuropsychologia*. Sep 2019;132:107119. doi:10.1016/j.neuropsychologia.2019.107119
32. Jenmalm P, Schmitz C, Forssberg H, Ehrsson HH. Lighter or heavier than predicted: neural correlates of corrective mechanisms during erroneously programmed lifts. *J Neurosci*. Aug 30 2006;26(35):9015-21. doi:10.1523/JNEUROSCI.5045-05.2006
33. Miall RC, Christensen LO, Cain O, Stanley J. Disruption of state estimation in the human lateral cerebellum. *PLoS Biol*. Nov 2007;5(11):e316. doi:10.1371/journal.pbio.0050316
34. Tomatsu S, Ishikawa T, Tsunoda Y, Lee J, Hoffman DS, Kakei S. Information processing in the hemisphere of the cerebellar cortex for control of wrist movement. *J Neurophysiol*. Jan 1 2016;115(1):255-70. doi:10.1152/jn.00530.2015
35. Schmitz C, Jenmalm P, Ehrsson HH, Forssberg H. Brain activity during predictable and unpredictable weight changes when lifting objects. *J Neurophysiol*. Mar 2005;93(3):1498-509. doi:10.1152/jn.00230.2004
36. Desmurget M, Grafton S. Forward modeling allows feedback control for fast reaching movements. *Trends Cogn Sci*. Nov 1 2000;4(11):423-431. doi:10.1016/s1364-6613(00)01537-0
37. Mani S, Mutha PK, Przybyla A, Haaland KY, Good DC, Sainburg RL. Contralesional motor deficits after unilateral stroke reflect hemisphere-specific control mechanisms. *Brain*. Apr 2013;136(Pt 4):1288-303. doi:10.1093/brain/aws283
38. Mutha PK, Haaland KY, Sainburg RL. The effects of brain lateralization on motor control and adaptation. *J Mot Behav*. 2012;44(6):455-69. doi:10.1080/00222895.2012.747482

39. Serrien DJ, Ivry RB, Swinnen SP. Dynamics of hemispheric specialization and integration in the context of motor control. *Nat Rev Neurosci*. Feb 2006;7(2):160-6. doi:10.1038/nrn1849
40. Schaefer SY, Haaland KY, Sainburg RL. Dissociation of initial trajectory and final position errors during visuomotor adaptation following unilateral stroke. *Brain Res*. Nov 17 2009;1298:78-91. doi:10.1016/j.brainres.2009.08.063
41. Schaefer SY, Haaland KY, Sainburg RL. Hemispheric specialization and functional impact of ipsilesional deficits in movement coordination and accuracy. *Neuropsychologia*. Nov 2009;47(13):2953-66. doi:10.1016/j.neuropsychologia.2009.06.025
42. Galloway JC, Koshland GF. General coordination of shoulder, elbow and wrist dynamics during multijoint arm movements. *Exp Brain Res*. Jan 2002;142(2):163-80. doi:10.1007/s002210100882
43. Liu W, Whitall J, Kepple TM. Multi-joint coordination of functional arm reaching: induced position analysis. *J Appl Biomech*. Apr 2013;29(2):235-40. doi:10.1123/jab.29.2.235
44. Stinear CM, Barber PA, Smale PR, Coxon JP, Fleming MK, Byblow WD. Functional potential in chronic stroke patients depends on corticospinal tract integrity. *Brain*. Jan 2007;130(Pt 1):170-80. doi:10.1093/brain/awl333
45. Schaechter JD, Fricker ZP, Perdue KL*, et al*. Microstructural status of ipsilesional and contralesional corticospinal tract correlates with motor skill in chronic stroke patients. *Hum Brain Mapp*. Nov 2009;30(11):3461-74. doi:10.1002/hbm.20770
46. Borich MR, Mang C, Boyd LA. Both projection and commissural pathways are disrupted in individuals with chronic stroke: investigating microstructural white matter correlates of motor recovery. *BMC Neurosci*. Aug 29 2012;13:107. doi:10.1186/1471-2202-13-107
47. Qiu M, Darling WG, Morecraft RJ, Ni CC, Rajendra J, Butler AJ. White matter integrity is a stronger predictor of motor function than BOLD response in patients with stroke. *Neurorehabil Neural Repair*. Mar-Apr 2011;25(3):275-84. doi:10.1177/1545968310389183
48. Li Y, Wu P, Liang F, Huang W. The microstructural status of the corpus callosum is associated with the degree of motor function and neurological deficit in stroke patients. *PLoS One*. 2015;10(4):e0122615. doi:10.1371/journal.pone.0122615
49. Stewart JC, Dewanjee P, Tran G*, et al*. Role of corpus callosum integrity in arm function differs based on motor severity after stroke. *Neuroimage Clin*. 2017;14:641-647. doi:10.1016/j.nicl.2017.02.023
50. Carter AR, Astafiev SV, Lang CE*, et al*. Resting interhemispheric functional magnetic resonance imaging connectivity predicts performance after stroke. *Ann Neurol*. Mar 2010;67(3):365-75. doi:10.1002/ana.21905
51. Jakobson LS, Servos P, Goodale MA, Lassonde M. Control of proximal and distal components of prehension in callosal agenesis. *Brain*. Oct 1994;117 (Pt 5):1107-13. doi:10.1093/brain/117.5.1107
52. De Renzi E, Faglioni P, Sorgato P. Modality-specific and supramodal mechanisms of apraxia. *Brain*. Jun 1982;105(Pt 2):301-12. doi:10.1093/brain/105.2.301
53. Barbieri C, De Renzi E. The executive and ideational components of apraxia. *Cortex*. Dec 1988;24(4):535-43. doi:10.1016/s0010-9452(88)80047-9
54. Scandola M, Gobbetto V, Bertagnoli S*, et al*. Gesture errors in left and right hemisphere damaged patients: A behavioural and anatomical study. *Neuropsychologia*. Sep 22 2021;162:108027. doi:10.1016/j.neuropsychologia.2021.108027
55. Vanbellingen T, Kersten B, Van Hemelrijk B*, et al*. Comprehensive assessment of gesture production: a new test of upper limb apraxia (TULIA). *Eur J Neurol*. Jan 2010;17(1):59-66. doi:10.1111/j.1468-1331.2009.02741.x
56. Buxbaum LJ, Shapiro AD, Coslett HB. Critical brain regions for tool-related and imitative actions: a componential analysis. *Brain*. Jul 2014;137(Pt 7):1971-85. doi:10.1093/brain/awu111

57. Goldenberg G, Spatt J. The neural basis of tool use. *Brain*. Jun 2009;132(Pt 6):1645-55. doi:10.1093/brain/awp080
58. Tarhan LY, Watson CE, Buxbaum LJ. Shared and Distinct Neuroanatomic Regions Critical for Tool-related Action Production and Recognition: Evidence from 131 Left-hemisphere Stroke Patients. *J Cogn Neurosci*. Dec 2015;27(12):2491-511. doi:10.1162/jocn_a_00876
59. Pazzaglia M, Smania N, Corato E, Aglioti SM. Neural underpinnings of gesture discrimination in patients with limb apraxia. *J Neurosci*. Mar 19 2008;28(12):3030-41. doi:10.1523/JNEUROSCI.5748-07.2008
60. Weiss PH, Ubben SD, Kaesberg S*, et al*. Where language meets meaningful action: a combined behavior and lesion analysis of aphasia and apraxia. *Brain Struct Funct*. Jan 2016;221(1):563-76. doi:10.1007/s00429-014-0925-3
61. Hoeren M, Kummerer D, Bormann T*, et al*. Neural bases of imitation and pantomime in acute stroke patients: distinct streams for praxis. *Brain*. Oct 2014;137(Pt 10):2796-810. doi:10.1093/brain/awu203
62. Greene C, Cieslak M, Volz LJ*, et al*. Finding maximally disconnected subnetworks with shortest path tractography. *Neuroimage Clin*. 2019;23:101903. doi:10.1016/j.nicl.2019.101903
63. Garcea FE, Greene C, Grafton ST, Buxbaum LJ. Structural Disconnection of the Tool Use Network after Left Hemisphere Stroke Predicts Limb Apraxia Severity. *Cereb Cortex Commun*. 2020;1(1):tgaa035. doi:10.1093/texcom/tgaa035
64. Binkofski F, Buxbaum LJ. Two action systems in the human brain. *Brain Lang*. Nov 2013;127(2):222-9. doi:10.1016/j.bandl.2012.07.007
65. Jayaram G, Stagg CJ, Esser P, Kischka U, Stinear J, Johansen-Berg H. Relationships between functional and structural corticospinal tract integrity and walking post stroke. *Clin Neurophysiol*. Dec 2012;123(12):2422-8. doi:10.1016/j.clinph.2012.04.026
66. Jones PS, Pomeroy VM, Wang J*, et al*. Does stroke location predict walk speed response to gait rehabilitation? *Hum Brain Mapp*. Feb 2016;37(2):689-703. doi:10.1002/hbm.23059
67. de Laat KF, Tuladhar AM, van Norden AG, Norris DG, Zwiers MP, de Leeuw FE. Loss of white matter integrity is associated with gait disorders in cerebral small vessel disease. *Brain*. Jan 2011;134(Pt 1):73-83. doi:10.1093/brain/awq343
68. Parlatini V, Radua J, Dell'Acqua F*, et al*. Functional segregation and integration within fronto-parietal networks. *NeuroImage*. 2017/02/01/ 2017;146:367-375. doi:https://doi.org/10.1016/j.neuroimage.2016.08.031
69. Barbeau EB, Descoteaux M, Petrides M. Dissociating the white matter tracts connecting the temporo-parietal cortical region with frontal cortex using diffusion tractography. *Sci Rep*. May 18 2020;10(1):8186. doi:10.1038/s41598-020-64124-y
70. Benson DF. *Aphasia, alexia, agraphia*. Churchill Livingstone; 1979.
71. Ivanova MV, Akinina YS, Soloukhina OA*, et al*. The Russian Aphasia Test: The First Comprehensive, Quantitative, Standardized, and Computerized Aphasia Language Battery in Russian. *PsyArXiv*. 2021;doi:doi:10.31234/osf.io/wajdz
72. Whiteside DM, Kealey T, Semla M*, et al*. Verbal Fluency: Language or Executive Function Measure? *Appl Neuropsychol Adult*. 2016;23(1):29-34. doi:10.1080/23279095.2015.1004574
73. Baldo JV, Schwartz S, Wilkins D, Dronkers NF. Role of frontal versus temporal cortex in verbal fluency as revealed by voxel-based lesion symptom mapping. *Journal of the International Neuropsychological Society*. 2006;12(6):896-900. doi:10.1017/S1355617706061078
74. Baldo JV, Shimamura AP, Delis DC, Kramer J, Kaplan E. Verbal and design fluency in patients with frontal lobe lesions. *Journal of the International Neuropsychological Society*. 2001;7(5):586-596. doi:10.1017/S1355617701755063

75. Henry JD, Crawford JR. A Meta-Analytic Review of Verbal Fluency Performance Following Focal Cortical Lesions. *Neuropsychology*. 2004;18(2):284-295. doi:10.1037/0894-4105.18.2.284
76. Marczinski CA, Kertesz A. Category and letter fluency in semantic dementia, primary progressive aphasia, and Alzheimer's disease. *Brain and Language*. 2006/06/01/ 2006;97(3):258-265. doi:https://doi.org/10.1016/j.bandl.2005.11.001
77. Catani M, Mesulam MM, Jakobsen E*, et al*. A novel frontal pathway underlies verbal fluency in primary progressive aphasia. *Brain*. Aug 2013;136(Pt 8):2619-28. doi:10.1093/brain/awt163
78. Kemerdere R, de Champfleur NM, Deverdun J*, et al*. Role of the left frontal aslant tract in stuttering: a brain stimulation and tractographic study. *Journal of Neurology*. 2016/01/01 2016;263(1):157-167. doi:10.1007/s00415-015-7949-3
79. Forkel SJ, Friedrich P, Thiebaut de Schotten M, Howells H. White matter variability, cognition, and disorders: a systematic review. *Brain Structure & Function*. 2021;(in press)
80. Tombaugh TN, Kozak J, Rees L. Normative Data Stratified by Age and Education for Two Measures of Verbal Fluency: FAS and Animal Naming. *Archives of Clinical Neuropsychology*. 1999/02/01/ 1999;14(2):167-177. doi:https://doi.org/10.1016/S0887-6177(97)00095-4
81. Kaplan E, Goodglass H, Weintraub S. *Boston naming test*. Lea & Febiger; 1983.
82. Mack WJ, Freed DM, Williams BW, Henderson VW. Boston Naming Test: shortened versions for use in Alzheimer's disease. *J Gerontol*. May 1992;47(3):P154-8. doi:10.1093/geronj/47.3.p154
83. Goodglass H, Kaplan E, Barresi B. *The Boston Diagnostic Aphasia Examination*. Lippincott; 2000.
84. Lansing AE, Ivnik RJ, Cullum CM, Randolph C. An empirically derived short form of the Boston naming test. *Arch Clin Neuropsychol*. Aug 1999;14(6):481-7.
85. Villardita C, Cultrera S, Cupone V, Mejìa R. Neuropsychological test performances and normal aging. *Archives of Gerontology and Geriatrics*. 1985/01/01/ 1985;4(4):311-319. doi:https://doi.org/10.1016/0167-4943(85)90038-X
86. Nicholas LE, Brookshire RH, Maclennan DL, Schumacher JG, Porrazzo SA. Revised administration and scoring procedures for the Boston Naming test and norms for non-brain-damaged adults. *Aphasiology*. 1989/09/01 1989;3(6):569-580. doi:10.1080/02687038908249023
87. Goodglass H, Kaplan E. *The assessment of aphasia and related disorders (2nd ed.)*. Liea & Febiger; 1983.
88. Thiebaut de Schotten M, Cohen L, Amemiya E, Braga LW, Dehaene S. Learning to read improves the structure of the arcuate fasciculus. *Cereb Cortex*. Apr 2014;24(4):989-95. doi:10.1093/cercor/bhs383
89. Dejerine J. Sur un cas de cécité verbale avec agraphie suivie d'autopsie. *Mem Soc Biol*. 1891;3:197-201.
90. Epelbaum S, Pinel P, Gaillard R*, et al*. Pure alexia as a disconnection syndrome: New diffusion imaging evidence for an old concept. *Cortex*. Sep 1 2008;44(8):962-974. doi:10.1016/j.cortex.2008.05.003
91. Gaillard R, Naccache L, Pinel P*, et al*. Direct intracranial, FMRI, and lesion evidence for the causal role of left inferotemporal cortex in reading. *Neuron*. Apr 20 2006;50(2):191-204. doi:10.1016/j.neuron.2006.03.031
92. Baldo JV, Kacinik N, Ludy C*, et al*. Voxel-based lesion analysis of brain regions underlying reading and writing. *Neuropsychologia*. 2018/07/01/ 2018;115:51-59. doi:https://doi.org/10.1016/j.neuropsychologia.2018.03.021
93. Naeser MA, Helm-Estabrooks N, Haas G, Auerbach S, Srinivasan M. Relationship between lesion extent in 'Wernicke's area' on computed tomographic scan and predicting

recovery of comprehension in Wernicke's aphasia. *Arch Neurol*. Jan 1987;44(1):73-82. doi:10.1001/archneur.1987.00520130057018

94. Crinion J, Price CJ. Right anterior superior temporal activation predicts auditory sentence comprehension following aphasic stroke. *Brain*. 2005;128(12):2858-2871. doi:10.1093/brain/awh659

95. Gajardo-Vidal A, Lorca-Puls DL, Hope TMH*, et al*. How right hemisphere damage after stroke can impair speech comprehension. *Brain*. 2018;141(12):3389-3404. doi:10.1093/brain/awy270

96. Robson H, Zahn R, Keidel JL, Binney RJ, Sage K, Lambon Ralph MA. The anterior temporal lobes support residual comprehension in Wernicke's aphasia. *Brain*. 2014;137(3):931-943. doi:10.1093/brain/awt373

97. Kreisler A, Godefroy O, Delmaire C*, et al*. The anatomy of aphasia revisited. *Neurology*. Mar 14 2000;54(5):1117-23. doi:10.1212/wnl.54.5.1117

98. Bates E, Wilson SM, Saygin AP*, et al*. Voxel-based lesion-symptom mapping. *Nat Neurosci*. May 2003;6(5):448-50. doi:10.1038/nn1050

99. Damasio AR. Aphasia. *N Engl J Med*. Feb 20 1992;326(8):531-9. doi:10.1056/NEJM199202203260806

100. Mesulam MM, Thompson CK, Weintraub S, Rogalski EJ. The Wernicke conundrum and the anatomy of language comprehension in primary progressive aphasia. *Brain*. Aug 2015;138(Pt 8):2423-37. doi:10.1093/brain/awv154

101. Bonilha L, Hillis AE, Hickok G, den Ouden DB, Rorden C, Fridriksson J. Temporal lobe networks supporting the comprehension of spoken words. *Brain*. 2017;140(9):2370-2380. doi:10.1093/brain/awx169

102. Glezer LS, Eden G, Jiang X*, et al*. Uncovering phonological and orthographic selectivity across the reading network using fMRI-RA. *Neuroimage*. Sep 2016;138:248-256. doi:10.1016/j.neuroimage.2016.05.072

103. Hagoort P. The neurobiology of language beyond single-word processing. *Science*. Oct 4 2019;366(6461):55-58. doi:10.1126/science.aax0289

104. Price CJ, Seghier ML, Leff AP. Predicting language outcome and recovery after stroke: the PLORAS system. *Nat Rev Neurol*. Apr 2010;6(4):202-10. doi:10.1038/nrneurol.2010.15

105. Wilson SM, Eriksson DK, Schneck SM, Lucanie JM. Correction: A quick aphasia battery for efficient, reliable, and multidimensional assessment of language function. *PLoS One*. 2018;13(6):e0199469. doi:10.1371/journal.pone.0199469

106. Kertesz A. Western Aphasia Battery-Revised. Pearson. https://www.pearsonassessments.com/store/usassessments/en/Store/Professional-Assessments/Speech-%26-Language/Western-Aphasia-Battery-Revised/p/100000194.html?tab=faqs#

107. Biesbroek JM, van Zandvoort MJ, Kappelle LJ, Velthuis BK, Biessels GJ, Postma A. Shared and distinct anatomical correlates of semantic and phonemic fluency revealed by lesion-symptom mapping in patients with ischemic stroke. *Brain Struct Funct*. May 2016;221(4):2123-34. doi:10.1007/s00429-015-1033-8

108. Knopman DS, Selnes OA, Niccum N, Rubens AB. Recovery of naming in aphasia: relationship to fluency, comprehension and CT findings. *Neurology*. Nov 1984;34(11):1461-70. doi:10.1212/wnl.34.11.1461

109. Meier EL, Sheppard SM, Goldberg EB*, et al*. Naming errors and dysfunctional tissue metrics predict language recovery after acute left hemisphere stroke. *Neuropsychologia*. 2020;148:107651-107651. doi:10.1016/j.neuropsychologia.2020.107651

110. Baldo JV, Arévalo A, Patterson JP, Dronkers NF. Grey and white matter correlates of picture naming: Evidence from a voxel-based lesion analysis of the Boston Naming Test. *Cortex*. 2013/03/01/ 2013;49(3):658-667. doi:https://doi.org/10.1016/j.cortex.2012.03.001

111. Woollams AM, Halai A, Lambon Ralph MA. Mapping the intersection of language and reading: the neural bases of the primary systems hypothesis. *Brain Structure and Function*. 2018/11/01 2018;223(8):3769-3786. doi:10.1007/s00429-018-1716-z
112. Cloutman LL, Newhart M, Davis CL, Heidler-Gary J, Hillis AE. Neuroanatomical correlates of oral reading in acute left hemispheric stroke. *Brain Lang*. Jan 2011;116(1):14-21. doi:10.1016/j.bandl.2010.09.002
113. Buiatti T, Skrap M, Shallice T. Left- and right-hemisphere forms of phonological alexia. *Cognitive Neuropsychology*. 2012/12/01 2012;29(7-8):531-549. doi:10.1080/02643294.2013.771773
114. Wilson B, Cockburn JF, Halligan P. Development of a behavioral test of visuospatial neglect. *Arch Phys Med Rehabil*. 1987;68(2):98-102.
115. Bailey MJ, Riddoch MJ, Crome P. Test–retest stability of three tests for unilateral visual neglect in patients with stroke: Star Cancellation, Line Bisection, and the Baking Tray Task. *Neuropsychological Rehabilitation*. 2004/09/01 2004;14(4):403-419. doi:10.1080/09602010343000282
116. Jehkonen M, Ahonen J-P, Dastidar P, Koivisto A-M, Laippala P, Vilkki J. How to detect visual neglect in acute stroke. *The Lancet*. 1998/03/07/ 1998;351(9104):727-728. doi:https://doi.org/10.1016/S0140-6736(05)78497-X
117. Rorden C, Karnath H-O. A simple measure of neglect severity. *Neuropsychologia*. 2010/07/01/ 2010;48(9):2758-2763. doi:https://doi.org/10.1016/j.neuropsychologia.2010.04.018
118. Catani M, Dell'Acqua F, Vergani F*, et al*. Short frontal lobe connections of the human brain. *Cortex*. 2012/02/01/ 2012;48(2):273-291. doi:https://doi.org/10.1016/j.cortex.2011.12.001
119. Rojkova K, Volle E, Urbanski M, Humbert F, Dell'Acqua F, Thiebaut de Schotten M. Atlasing the frontal lobe connections and their variability due to age and education: a spherical deconvolution tractography study. *Brain Structure and Function*. 2016/04/01 2016;221(3):1751-1766. doi:10.1007/s00429-015-1001-3
120. Catani M, Thiebaut de Schotten M. *Atlas of human brain connections*. Oxford University Press; 2012:xii, 519 p.
121. Catani M, Robertsson N, Beyh A*, et al*. Short parietal lobe connections of the human and monkey brain. *Cortex*. 2017/12/01/ 2017;97:339-357. doi:https://doi.org/10.1016/j.cortex.2017.10.022
122. Corbetta M, Kincade MJ, Lewis C, Snyder AZ, Sapir A. Neural basis and recovery of spatial attention deficits in spatial neglect. *Nature Neuroscience*. 2005/11/01 2005;8(11):1603-1610. doi:10.1038/nn1574
123. He BJ, Snyder AZ, Vincent JL, Epstein A, Shulman GL, Corbetta M. Breakdown of Functional Connectivity in Frontoparietal Networks Underlies Behavioral Deficits in Spatial Neglect. *Neuron*. 2007/03/15/ 2007;53(6):905-918. doi:https://doi.org/10.1016/j.neuron.2007.02.013
124. Heilman KM, Valenstein E. Frontal lobe neglect in man. *Neurology*. 1998;50(5):1202. doi:10.1212/WNL.50.5.1202-a
125. Husain M, Kennard C. Distractor-dependent frontal neglect. *Neuropsychologia*. 1997/05/19/ 1997;35(6):829-841. doi:https://doi.org/10.1016/S0028-3932(97)00034-1
126. Manes F, Paradiso S, Springer JA, Lamberty G, Robinson RG. Neglect after right insular cortex infarction. *Stroke*. May 1999;30(5):946-8. doi:10.1161/01.str.30.5.946
127. Kranczioch C, Debener S, Schwarzbach J, Goebel R, Engel AK. Neural correlates of conscious perception in the attentional blink. *Neuroimage*. Feb 1 2005;24(3):704-14. doi:10.1016/j.neuroimage.2004.09.024
128. Weissman DH, Roberts KC, Visscher KM, Woldorff MG. The neural bases of momentary lapses in attention. *Nature Neuroscience*. 2006/07/01 2006;9(7):971-978. doi:10.1038/nn1727

129. Karnath H-O, Ferber S, Himmelbach M. Spatial awareness is a function of the temporal not the posterior parietal lobe. *Nature*. 2001/06/01 2001;411(6840):950-953. doi:10.1038/35082075
130. Karnath H-O, Rennig J, Johannsen L, Rorden C. The anatomy underlying acute versus chronic spatial neglect: a longitudinal study. *Brain*. 2011;134(3):903-912. doi:10.1093/brain/awq355
131. Mort DJ, Malhotra P, Mannan SK*, et al*. The anatomy of visual neglect. *Brain*. 2003;126(9):1986-1997. doi:10.1093/brain/awg200
132. Husain M, Rorden C. Non-spatially lateralized mechanisms in hemispatial neglect. *Nature Reviews Neuroscience*. 2003/01/01 2003;4(1):26-36. doi:10.1038/nrn1005
133. Mesulam MM. *Principle of Behavioural Neurology: Tests of Directed Attention and Memory (Contemporary Neurology Series)*. F.A. Davis Company; 1985.
134. Treisman AM, Gelade G. A feature-integration theory of attention. *Cognitive Psychology*. 1980/01/01/ 1980;12(1):97-136. doi:https://doi.org/10.1016/0010-0285(80)90005-5
135. Dalla Barba G, Brazzarola M, Barbera C*, et al*. Different patterns of confabulation in left visuo-spatial neglect. *Experimental Brain Research*. 2018/07/01 2018;236(7):2037-2046. doi:10.1007/s00221-018-5281-8
136. Ungerleider L, Mishkin M. *Two cortical visual systems. In Analysis of visual behavior*. MIT Press; 1982.
137. Kravitz DJ, Saleem KS, Baker CI, Mishkin M. A new neural framework for visuospatial processing. *Nature Reviews Neuroscience*. 2011/04/01 2011;12(4):217-230. doi:10.1038/nrn3008
138. Baars BJ. The conscious access hypothesis: origins and recent evidence. *Trends in Cognitive Sciences*. 2002/01/01/ 2002;6(1):47-52. doi:https://doi.org/10.1016/S1364-6613(00)01819-2
139. Dehaene S, Changeux J-P. Experimental and Theoretical Approaches to Conscious Processing. *Neuron*. 2011/04/28/ 2011;70(2):200-227. doi:https://doi.org/10.1016/j.neuron.2011.03.018
140. Geschwind N. Disconnexion syndromes in animals and man. *Brain*. 1965;88(3):585-585. doi:10.1093/brain/88.3.585
141. Bartolomeo P, Thiebaut de Schotten M, Doricchi F. Left Unilateral Neglect as a Disconnection Syndrome. *Cerebral Cortex*. 2007;17(11):2479-2490. doi:10.1093/cercor/bhl181
142. Lunven M, Thiebaut De Schotten M, Bourlon C*, et al*. White matter lesional predictors of chronic visual neglect: a longitudinal study. *Brain*. 2015;138(3):746-760. doi:10.1093/brain/awu389
143. Watson RT, Heilman KM. Thalamic neglect. *Neurology*. May 1979;29(5):690-4. doi:10.1212/wnl.29.5.690
144. Watson RT, Valenstein E, Heilman KM. Thalamic Neglect: Possible Role of the Medial Thalamus and Nucleus Reticularis in Behavior. *Archives of Neurology*. 1981;38(8):501-506. doi:10.1001/archneur.1981.00510080063009
145. Cambier J, Elghozi D, Strube E. Lésion du thalamus droit avec syndrome de l'hémisphère mineur. Discussion du concept de négligence thalamique. *Rev Neurol* 1980;136:105–116.
146. Graff-Radford NR, Damasio H, Yamada T, Eslinger PJ, Damasio AR. Nonhaemorrhagic thalamic infarction: clinical, neuropsychological and electrophysiological findings in four anatomical groups defined by computerized tomography. *Brain*. 1985;108(2):485-516. doi:10.1093/brain/108.2.485
147. Hirose G, Kosoegawa H, Saeki M*, et al*. The syndrome of posterior thalamic hemorrhage. *Neurology*. Jul 1985;35(7):998-1002. doi:10.1212/wnl.35.7.998

148. Bogousslavsky J, Regli F, Assal G. The syndrome of unilateral tuberothalamic artery territory infarction. *Stroke*. May-Jun 1986;17(3):434-41. doi:10.1161/01.str.17.3.434
149. Vallar G, Perani D. The anatomy of unilateral neglect after right-hemisphere stroke lesions. A clinical/CT-scan correlation study in man. *Neuropsychologia*. 1986/01/01/ 1986;24(5):609-622. doi:https://doi.org/10.1016/0028-3932(86)90001-1
150. Waxman SG, Ricaurte GA, Tucker SB. Thalamic hemorrhage with neglect and memory disorder. *Journal of the Neurological Sciences*. 1986/08/01/ 1986;75(1):105-112. doi:https://doi.org/10.1016/0022-510X(86)90053-5
151. Rafal RD, Posner MI. Deficits in human visual spatial attention following thalamic lesions. *Proceedings of the National Academy of Sciences*. 1987;84(20):7349. doi:10.1073/pnas.84.20.7349
152. Kumral E, Kocaer T, Ertubey NO, Kumral K. Thalamic hemorrhage. A prospective study of 100 patients. *Stroke*. Jun 1995;26(6):964-70. doi:10.1161/01.str.26.6.964
153. Chung C-S, Caplan LR, Han W, Pessin MS, Lee K-H, Kim J-M. Thalamic haemorrhage. *Brain*. 1996;119(6):1873-1886. doi:10.1093/brain/119.6.1873
154. Leibovitch FS, Black SE, Caldwell CB, Ebert PL, Ehrlich LE, Szalai JP. Brain-behavior correlations in hemispatial neglect using CT and SPECT: the Sunnybrook Stroke Study. *Neurology*. Apr 1998;50(4):901-8. doi:10.1212/wnl.50.4.901
155. Karussis D, Leker RR, Abramsky O. Cognitive dysfunction following thalamic stroke: a study of 16 cases and review of the literature. *Journal of the Neurological Sciences*. 2000/01/01/ 2000;172(1):25-29. doi:https://doi.org/10.1016/S0022-510X(99)00267-1
156. Karnath HO, Himmelbach M, Rorden C. The subcortical anatomy of human spatial neglect: putamen, caudate nucleus and pulvinar. *Brain*. 2002;125(2):350-360. doi:10.1093/brain/awf032
157. De Witte L, Verhoeven J, Engelborghs S, De Deyn PP, Marien P. Crossed aphasia and visuo-spatial neglect following a right thalamic stroke: a case study and review of the literature. *Behav Neurol*. 2008;19(4):177-94. doi:10.1155/2008/905187
158. Thiebaut de Schotten M, Dell'Acqua F, Forkel SJ*, et al*. A lateralized brain network for visuospatial attention. *Nature Neuroscience*. 2011/10/01 2011;14(10):1245-1246. doi:10.1038/nn.2905
159. Corbetta M, Shulman GL. Control of goal-directed and stimulus-driven attention in the brain. *Nature Reviews Neuroscience*. 2002/03/01 2002;3(3):201-215. doi:10.1038/nrn755
160. Thiebaut de Schotten M, Urbanski M, Duffau H*, et al*. Direct Evidence for a Parietal-Frontal Pathway Subserving Spatial Awareness in Humans. *Science*. 2005/09/30 2005;309(5744):2226-2228. doi:10.1126/science.1116251
161. Thiebaut de Schotten M, Tomaiuolo F, Aiello M*, et al*. Damage to White Matter Pathways in Subacute and Chronic Spatial Neglect: A Group Study and 2 Single-Case Studies with Complete Virtual "In Vivo" Tractography Dissection. *Cerebral Cortex*. 2014;24(3):691-706. doi:10.1093/cercor/bhs351
162. Eckert MA, Menon V, Walczak A*, et al*. At the heart of the ventral attention system: The right anterior insula. https://doi.org/10.1002/hbm.20688. *Human Brain Mapping*. 2009/08/01 2009;30(8):2530-2541. doi:https://doi.org/10.1002/hbm.20688
163. Menon V, Uddin LQ. Saliency, switching, attention and control: a network model of insula function. *Brain Structure and Function*. 2010/06/01 2010;214(5):655-667. doi:10.1007/s00429-010-0262-0
164. Bird CM, Malhotra P, Parton A, Coulthard E, Rushworth MFS, Husain M. Visual neglect after right posterior cerebral artery infarction. *Journal of Neurology, Neurosurgery & Psychiatry*. 2006;77(9):1008. doi:10.1136/jnnp.2006.094417
165. Bartolomeo P, Chokron S. Orienting of attention in left unilateral neglect. *Neuroscience & Biobehavioral Reviews*. 2002/03/01/ 2002;26(2):217-234. doi:https://doi.org/10.1016/S0149-7634(01)00065-3

166. Akelaitis AJ. Studies of the corpus callosum: IV. Diagonistic dyspraxia in epileptics following partial and complete section of the corpus callosum. *Am J Psychiat*. 1945;101:594–599.
167. Bogen JE. Physiological consequences of complete or partial commissural section. In: MLJ A, ed. *Surgery of the third ventricle*. Baltimore, Williams and Wilkins; 1987:175–194.
168. Goldstein K. Zur Lehre von der motorisschen Apraxie. *J Psychol Neurol*. 1908;XI:169–187.
169. Della Sala S, Marchetti C, Spinnler H. Right-sided anarchic (alien) hand: a longitudinal study. *Neuropsychologia*. 1991;29(11):1113-27. doi:10.1016/0028-3932(91)90081-i
170. Marchetti C, Della Sala S. Disentangling the alien and anarchic hand. . *Cognitive Neuropsychiatry*. 1998;3(3):191–207.
171. Caplan B, Kinsbourne M. Cerebral lateralization, preferred cognitive mode, and reading ability in normal children. *Brain and Language*. 1981/11/01/ 1981;14(2):349-370. doi:https://doi.org/10.1016/0093-934X(81)90085-7
172. Gaffan D, Hornak J. Visual neglect in the monkey. Representation and disconnection. *Brain*. 1997;120(9):1647-1657. doi:10.1093/brain/120.9.1647
173. Luck SJ, Hillyard SA, Mangun GR, Gazzaniga MS. Independent hemispheric attentional systems mediate visual search in split-brain patients. *Nature*. 1989/11/01 1989;342(6249):543-545. doi:10.1038/342543a0
174. Garic D, Broce I, Graziano P, Mattfeld A, Dick AS. Laterality of the frontal aslant tract (FAT) explains externalizing behaviors through its association with executive function. https://doi.org/10.1111/desc.12744. *Developmental Science*. 2019/03/01 2019;22(2):e12744. doi:https://doi.org/10.1111/desc.12744
175. Aron AR, Fletcher PC, Bullmore ET, Sahakian BJ, Robbins TW. Stop-signal inhibition disrupted by damage to right inferior frontal gyrus in humans. *Nature Neuroscience*. 2003/02/01 2003;6(2):115-116. doi:10.1038/nn1003
176. Nelson AJD, Powell AL, Kinnavane L, Aggleton JP. Anterior thalamic nuclei, but not retrosplenial cortex, lesions abolish latent inhibition in rats. *Behav Neurosci*. Oct 2018;132(5):378-387. doi:10.1037/bne0000265
177. Koini M, Rombouts SARB, Veer IM, Van Buchem MA, Huijbregts SCJ. White matter microstructure of patients with neurofibromatosis type 1 and its relation to inhibitory control. *Brain Imaging and Behavior*. 2017/12/01 2017;11(6):1731-1740. doi:10.1007/s11682-016-9641-3
178. Huber E, Donnelly PM, Rokem A, Yeatman JD. Rapid and widespread white matter plasticity during an intensive reading intervention. *Nature Communications*. 2018/06/08 2018;9(1):2260. doi:10.1038/s41467-018-04627-5
179. Karolis VR, Corbetta M, Thiebaut de Schotten M. The architecture of functional lateralisation and its relationship to callosal connectivity in the human brain. *Nature Communications*. 2019/03/29 2019;10(1):1417. doi:10.1038/s41467-019-09344-1
180. Hampshire A, Chamberlain SR, Monti MM, Duncan J, Owen AM. The role of the right inferior frontal gyrus: inhibition and attentional control. *NeuroImage*. 2010/04/15/ 2010;50(3):1313-1319. doi:https://doi.org/10.1016/j.neuroimage.2009.12.109
181. Sebastian A, Pohl MF, Klöppel S*, et al.* Disentangling common and specific neural subprocesses of response inhibition. *NeuroImage*. 2013/01/01/ 2013;64:601-615. doi:https://doi.org/10.1016/j.neuroimage.2012.09.020
182. Scheffer M, Kroeff C, Steigleder BG, Klein LA, Grassi-Oliveira R, de Almeida RMM. Right frontal stroke: extra-frontal lesions, executive functioning and impulsive behaviour. *Psicologia: Reflexão e Crítica*. 2016/04/21 2016;29(1):28. doi:10.1186/s41155-016-0018-8
183. Kim EJ, Choi KD, Han MK*, et al.* Hemispatial neglect in cerebellar stroke. *Journal of the Neurological Sciences*. 2008/12/15/ 2008;275(1):133-138. doi:https://doi.org/10.1016/j.jns.2008.08.012

184. Milano NJ, Heilman KM. Cerebellar Allocentric and Action-Intentional Spatial Neglect. *Cognitive and Behavioral Neurology*. 2014;27(3)
185. Koski LM, Paus T, Petrides M. Directed attention after unilateral frontal excisions in humans. *Neuropsychologia*. 1998/12/01/ 1998;36(12):1363-1371. doi:https://doi.org/10.1016/S0028-3932(98)00018-9
186. Olson IR, Heide RJVD, Alm KH, Vyas G. Development of the uncinate fasciculus: Implications for theory and developmental disorders. *Developmental Cognitive Neuroscience*. 2015/08/01/ 2015;14:50-61. doi:https://doi.org/10.1016/j.dcn.2015.06.003
187. Mashour GA, Roelfsema P, Changeux J-P, Dehaene S. Conscious Processing and the Global Neuronal Workspace Hypothesis. *Neuron*. 2020/03/04/ 2020;105(5):776-798. doi:https://doi.org/10.1016/j.neuron.2020.01.026
