# Supplementary material for: Latent disconnectome prediction of long-term cognitive-behavioural symptoms in stroke
Source: Brain. 2023 Mar 16;146(5):1963–78. doi: 10.1093/brain/awad013 (PMC10151183; doi:10.1093/brain/awad013)
Supplement: awad013_Supplementary_Data [file awad013_Supplementary_Data.zip › brain-2022-00965-File012.pdf]

Supplementary Materials

TABLE OF CONTENTS

C. Neuropsychological evaluations and composite morphospace maps

C.4 Visuospatial memory. Supplementary Figures 32-35 p.2

C.5 Verbal memory. Supplementary Figures 36-38 p.7

C.6. Pain. Supplementary Figures 39 p.13

C.7. Sickness profile. Supplementary Figures 40-49 p.14

C. Neuropsychological evaluations and composite morphospace maps

C.4 Visuospatial memory

Brief visuospatial memory test revised

The brief visuospatial memory test - revised BVMT, [1] has been used as assessment for visuospatial domain of the memory ability. The test scores are divided in recall memory (immediate/delayed) and additional learning and memory scores (learning, percentage retained, discrimination index, response bias, false alarm).

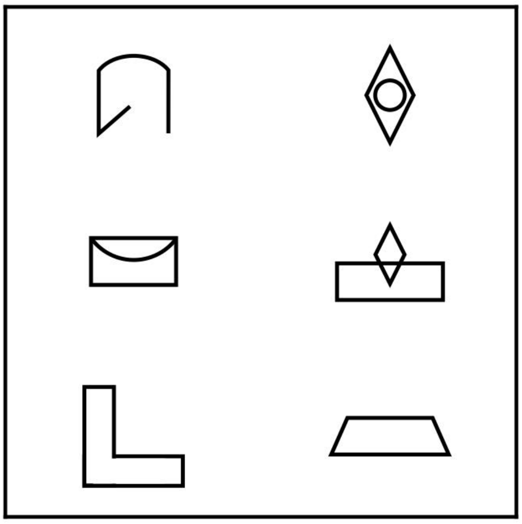

Supplementary Figure 32. Example of the BVMT stimuli.

The test material is an 8x11-inch plate containing six geometrical figures presented in a 2x3 matrix (Supplementary Figure 32). The patients are required to observe the figures in three consecutive trials lasting 10s each. The immediate recall task occurs at the end of the first trial, when the patients must draw the figures as accurately and fast as they can. The figures are presented again in two following trials (learning trials), at the end of which the patients are encouraged to improve their performance. After a delay of 25m, the delayed recall task is administered, during which the patients are instructed to reproduce the figure matrix by memory. Finally, in the delayed recognition task, the patients are presented with 12 drawings representing the six matrix figures (target) and six foil figures (nontarget). Patients are required to recognise the six matrix figures, responding "yes" when the target figures are presented and "no" when the non-target figures are shown.

For each response in the immediate and delayed recall a 0-1 score is assigned for each drawing according to the drawing position. The type of drawing is also evaluated on a 0-1 scoring scale for each drawing, for a total of 0-36 range score for the immediate recall and 0-18 range for the delayed recall. The immediate and delayed recall scores have been age-normed using the tables provided in the test manual, providing two additional scores, the immediate t and delayed t-scores. Furthermore, scores were calculated for the following variables: 1) Learning, how much the patient has learned by the later trials during the learning phase compared to the first trial (trial 1 - the better of trial 2 or 3); Percent Retained, is the percentage of how much an individual remembers from the later immediate recall trials following the 25-minute delay ((delayed recall score/the better score of trial 2 or 3)*100); 4) Recognition Hits, the total number of correctly identified drawings in the recognition phase (score range: 0-6); 5) Recognition False Alarms, total number of incorrectly identified drawings in the

recognition phase (score range: 0-6); 6) Delayed Recognition discrimination index, calculated from proportion of correct recognitions, correct rejections, misses, and false alarms; 7) Recognition Response Bias, how likely the patient is to say "yes" rather than "no" during the recognition phase (score range: 0-1).

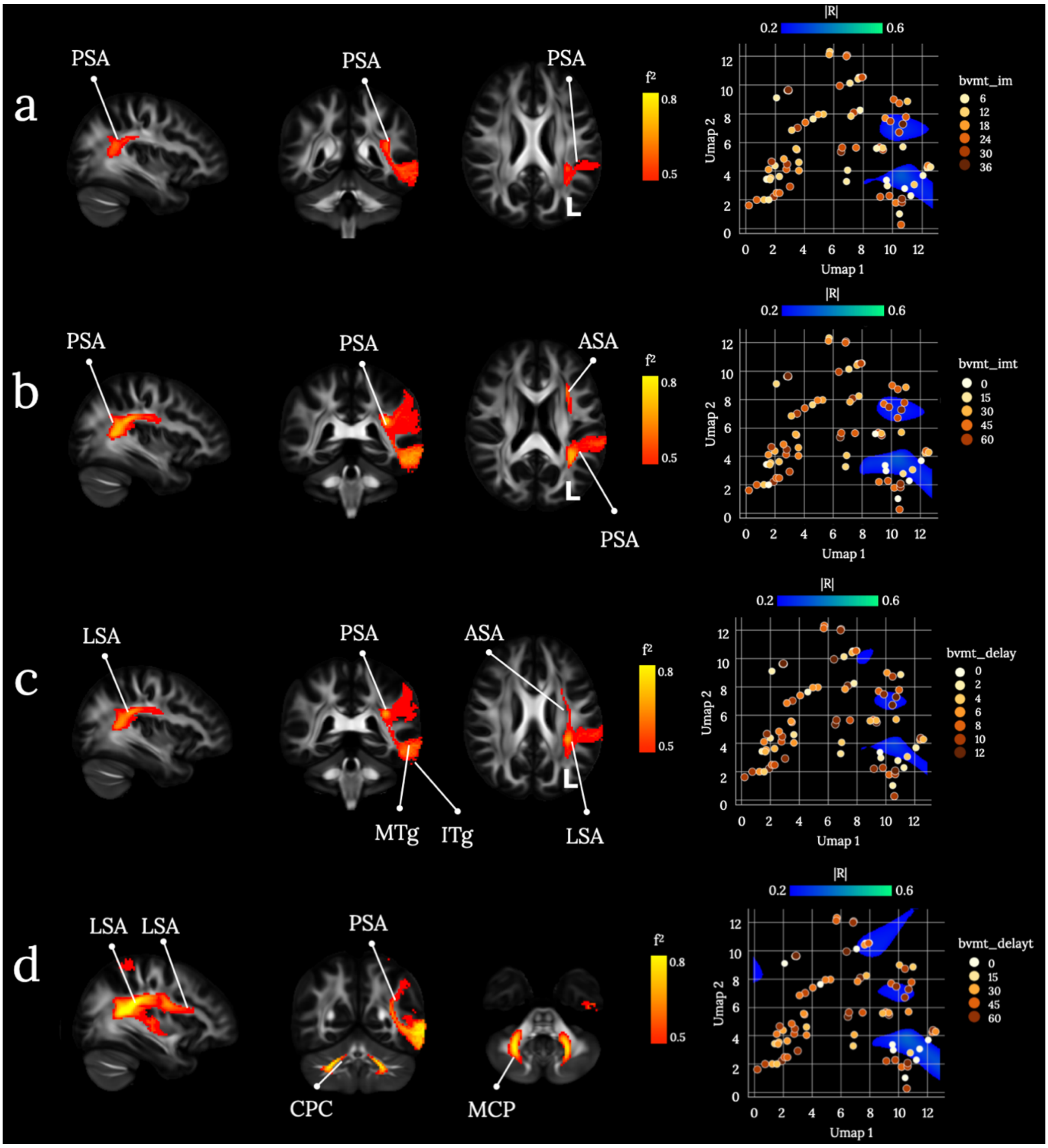

Supplementary Figure 33. Brain disconnections and UMAP related territories contributing significantly to four variables (immediate recall, age-normed immediate recall, immediate delay, age-normed immediate delay) of the Brief visuospatial memory test revised (BVTM-R). (a) Immediate recall (bvmt-im), (b) Age-normed immediate recall (bvmt-imt), (c) Immediate delay (bvmt-delay), (d) Age-normed immediate delay (bvmt-delayt). ASA: Anterior Segment of the arcuate fasciculus; CPC: Cortico-Ponto-Cerebellar tract; ITg: Inferior Temporal gyrus; LSA: Long Segment of the Arcuate fasciculus; MCP: Middle Cerebellar Peduncles; MTg: Middle Temporal gyrus. Maps are freely available at https://neurovault.org/collections/11260/ .

The low scores in immediate recall (raw and t-scores) and delayed recall (raw scores and t-scores) of the BVMT cluster in correspondence of frontoparietal and parietotemporal connections of the left hemisphere. The age-normed scores of the delayed recall are predicted also by the disconnection of the

cortico-pontine-cerebellar tract. Memory recall has been associated with attentional and integration processes subserved by the left frontoparietal structures [2-5]. It has been put forward the hypothesis that different parietal cortices contribute with diverse attentional processes to memory recall. Namely, the dorsal parietal and prefrontal cortices seem to carry out top-down attentional control in the active memory retrieval, while the ventral parietal region subserves the bottom-up attentional processing of spontaneous retrieval [6]. Unfortunately, results of the contribution of the left parietal cortex to memory recall are not consistent [7; for a review].

Nevertheless, visuospatial memory has been prevalently associated with right hemisphere structures [7] and previous studies reported the involvement of different brain structures during the performance of visuospatial memory immediate and delayed recall. For instance, the activation of the right prefrontal cortex has been associated with the performance in the two recall types of the BVTM [8], and the studies on brain damage and neurodegenerative disorders confirmed the role of the medial temporal cortex in memory recall.

However, immediate and delayed recall sub-tasks of the BVMT require object form and object position memory, which rely on visuospatial and mental visual-imagery processes as lesion study revealed [9,10]. A recent meta-analysis [11] put forward the hypothesis that left frontoparietal and temporal structures participate in the visual mental imagery network by internally driving the attentional control. Thus, our results may reflect the patients' deficit in other cognitive domains required by the BVTM.

Adjusting the delayed recall scores for the age allowed the scores to cluster in correspondence of the cortico-pontine cerebellar tract within the cerebellum. The role of the cerebellum in visual-motor, implicit motor and stimuli-response learning is well established [12-14], as well its participation in conscious memory retrieval [15-17].

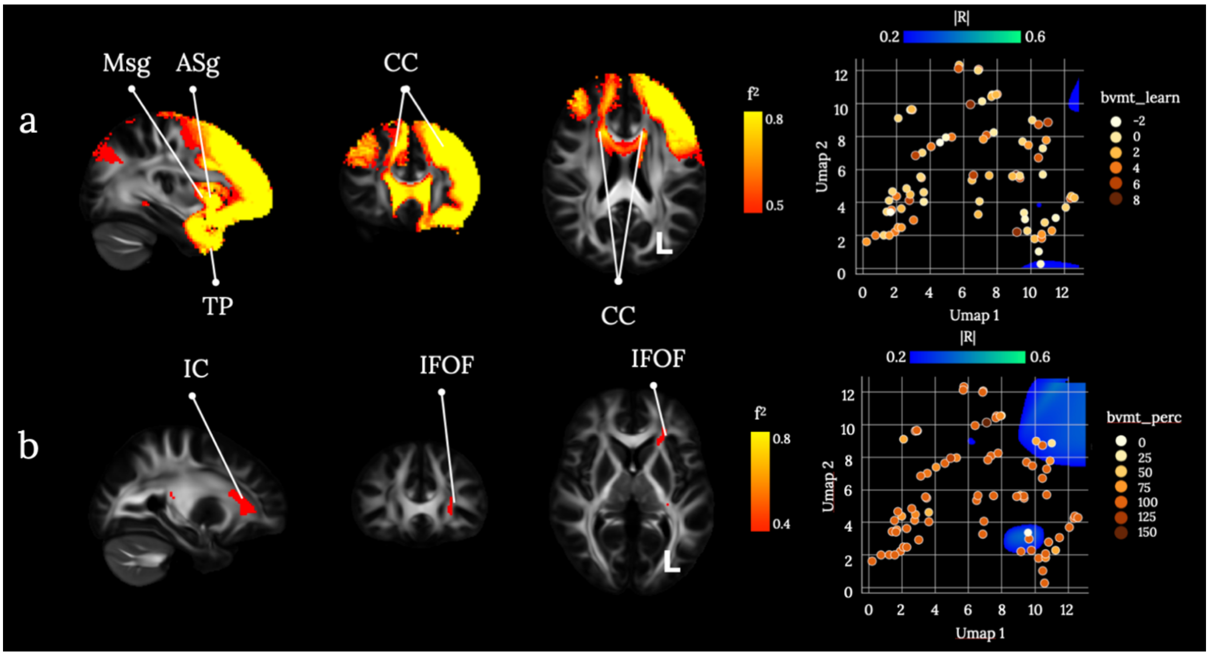

Supplementary Figure 34: Brain disconnections and UMAP related territories contributing significantly to two variables (learning, and percent retained) of the Brief visuospatial memory test revised (BVTM-R). (a) Learning (bvmt-learn), (b) percent retained (bvmt-imt). ASg: Anterior Short insular gyrus; CC: Corpus Callosum; IC: Internal Capsule; IFOF: Inferior Fronto-Occipital Fasciculus; Msg: Middle Short insular gyrus; TP: Temporal Pole. Maps are freely available at https://neurovault.org/collections/11260/ .

The low score in the learning variable is predicted mainly by the disconnection of the corpus callosum. Studies on patients with the stenosis of the corpus callosum have revealed the importance of the interhemispheric connection in visual learning [18,19] Specifically, the corpus callosum is associated with

visuomotor processing [20,21] and our results confirm that the deficit of visual and motor information integration prevents the performance improvement trial by trial.

The disconnection of fronto-occipital structures via the left Inferior Fronto-Occipital Fasciculus predicts low scores of the percent retained scores. The anatomical studies that explored in depth the IFOF structure have identified different components of the tract [22-25]. Interestingly, a study explored the contribution of the different components beyond their participation in language-domain processing [25]. While the anterior and posterior, deep components may be involved in emotion and semantic processing, the middle deep component seems to be dedicated to multi sensor-motor integration. Given that the percent retained score reflects the information retained from previous drawing trials, the disconnection of the IFOF may indicate that sensory-motor integration is the key to the improvement of the drawing performance after a delay.

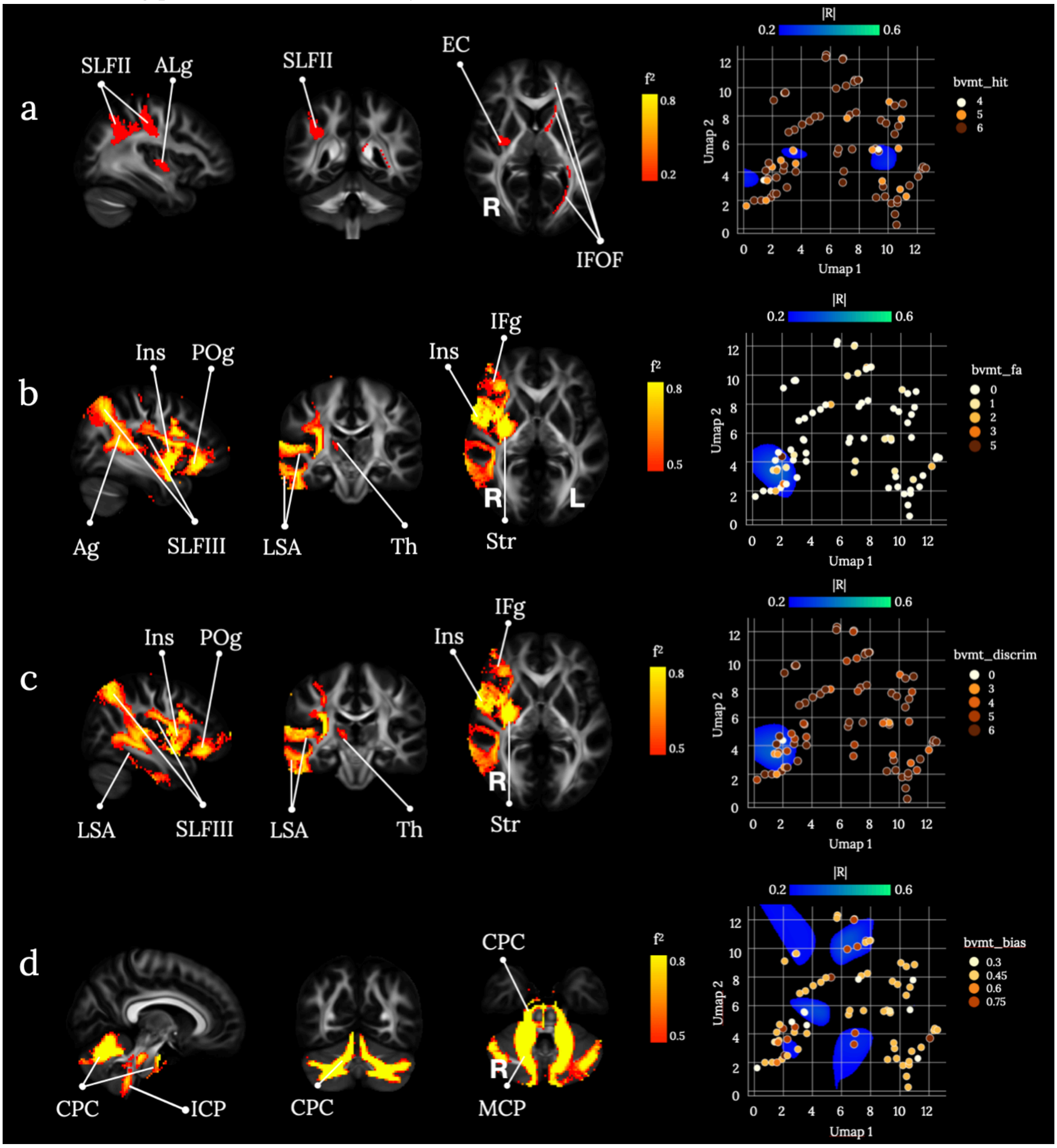

Supplementary Figure 35: Brain disconnections and UMAP related territories contributing significantly to four variables (recognition hits, recognition false alarms, delayed recognition discrimination index, recognition response bias) of the Brief visuospatial memory test revised (BVTM-R). (a) Recognition hits (bvmt-hit), (b) recognition false alarms (bvmt-fa), (c) delayed recognition discrimination index (bvmt-discrim), (d) recognition response bias (bvmt-bias). Ag: Angular gyrus;

ALg: Anterior Long insular gyrus; ALg: Anterior Long insular gyrus; CPC: Cortico-Ponto-Cerebellar tract; EC: External/Extreme Capsule; ICP: Inferior Cerebellar Peduncles; IFg: Inferior Frontal gyrus; IFOF: Inferior Fronto-Occipital Fasciculus; Ins: Insula; LSA: Long Segment of the Arcuate fasciculus; MCP: Middle Cerebellar Peduncles; POg: Posterior Orbito gyrus; SLFII: second branch of the Superior Longitudinal Fasciculus; SLFIII: third branch of the Superior Longitudinal Fasciculus; Str: Striatum; Th: Thalamus. Maps are freely available at https://neurovault.org/collections/11260/ .

A poor performance in the recognition hits score is credited by the disconnection of the angular gyrus and the middle frontal gyrus via the middle, via the second branch of the superior longitudinal fasciculus (SLF II), and the fronto-occipital disconnection via the IFOF. The low scores of the false alarm and the delayed recognition discrimination test cluster in correspondence of the disconnection of the supra marginal gyrus and the inferior frontal gyrus, the insula, and the posterior orbitofrontal cortex. The insula and the fronto-parietal network are part of the salience network, which is engaged during the top-down driving of attention toward relevant stimuli [26] and their activation has been associated with recognition performance [27-29]. The prefrontal cortex participates in the identification of cues during recognition and exploits the cues to reactivate stored information, then it supports the monitoring, disambiguation and verification of the retrieved information [30]. Further, frontal cortex damage prevents to exert executive control of the recognition process leading to false familiarity attributions [31], and the damage of the fronto-striatal pathway with the subsequent deficit in executive control is believed to contribute to memory impairment in Parkinson's disease patients [32; for a review]. Our results confirm that the integrity of attentional and executive control mechanisms supported by the insular, frontoparietal and striatal connections is crucial to accurately recognise the correct drawings of the BVTM.
The contribution of the fronto-occipital disconnection in the recognition hit scores may suggest that the hits are specifically associated to the visuomotor integration that is needed for the drawing performance improvement described in the percent retained scores section.

The low scores of the recognition bias correspond to less likelihood that a patient responds "yes" during the recognition phase and are predicted by the disconnections of the cerebellum. It has been proposed that the tendency to less likely identify an item as already presented rather than "new" indicates the need for more cues to use in the memory recognition process [33]. Previous studies have identified the contribution to recognition bias of top-down decision-making processes driven by the prefrontal cortex [34,35]. The participation of the cerebellum in decision making under uncertainty has been reported as well [36,37]. Blackwood and colleagues (2004) proposed that the cerebellum is involved in the building of internal models of external events, given its activation during uncertain tasks demands. These models may support the ability to infer predictions of the uncertainty. One may speculate that the recognition bias score of the BVTM mostly rely on the ability of making predictions during the categorisation of the stimuli as old and new.

C.5 Verbal memory

The Hopkins Verbal Learning Test HVLT, [38] serves as a measure of verbal learning and memory. The HVLT is composed of 12 items, organised into three semantic categories, and presented over three consecutive learning trials. The examiner reads the list aloud at the rate of 1 word every 2 seconds, and the patient is asked to memorise the list. The measure of interest is the patient's free recall. This procedure is repeated two more times. After the learning period, 24 words are read aloud to the patient who needs to verbally indicate if the word was also in the previous 12-item list (12 targets) or not (12 distractors). Whereby the latter are balanced between related distractors, meaning from the same semantic clusters as the target words, or unrelated. The HVLT has three main advantages: a short administration time (~10min), 6 parallel versions allowing for repeated measures for patients who are assessed at frequent intervals, and no ceiling effect on recall in healthy controls. However, while the HVLT appears to adequately assess basic verbal learning capacity, its utility in assessing some of the more complex and qualitative aspects of verbal learning and memory function may be limited [39].
We extracted the following scores from the HVLT: the absolute total immediate recall (hvlt_im) with a maximum score of 36 (all 12 words are recalled perfectly across the three trials), learning (hvlt_learn), percentage retained (hvlt_perc), hits (hclt_hit), false-positives related (hvlt_fa1), false-positives unrelated (hvlt_fa2), total false positives (hvlt_fa3), the recognition discremination index (hvlt_discrim), total immediate recall t-scores (hvlt_imt), delayed recall as t-scores (hvlt_delayt), and teh t-score converted discrimination index (hvlt_discrimt). The total immediate recall (hvlt_im) did not survive the first thresholding of R>0.2 and is therefore not included in the further analysis. It is of note though, that immediate recall is a commonly used measure of the test in neuropsychological studies.

Form 1: Four-legged animals, precious stones, human dwellings

Form 1, Part A: Free Recall:

	Trial 1	Trial 2	Trial 3
EMERALD			
HORSE			
TENT			
SAPPHIRE			
HOTEL			
CAVE			
OPAL			
TIGER			

PEARL			
COW			
HUT			
LION			
Number correct recall:			

Form 1, Part B: Recognition

Horse	Ruby*	Cave	Balloon	Coffee	Lion
House*	Opal	Tiger	Boat	Scarf	Pearl
Hut	Emerald	Sapphire	Dog*	Apartment*	Penny
Tent	Mountain	Cat*	Hotel	Cow	Diamond*

NOTE: An asterix (*) indicates semantically related distractors, while underlined words are the target items from the training list.

Form 1, Scoring:

Number of true positives (max value is 12): _______

Number of false-positive errors (max value is 12):
Related distractors (max 6):____ Unrelated (max6): ____

Discrimination Index: (Number true-positives) – (Number false-positives) = _______

NOTE from the authors: While Form 1 was exemplified in detail for the parallel forms, only the items of the parallel forms are listed below.

Form 2 word list (semantic categories: kitchen utensils, alcoholic beverages, weapons):
Free Recall: Fork, Rum, Pan, Pistol, Sword, Spatula, Bourbon, Vodka, Pot, Cow, Hut, Wine
Recognition:

spoon*	pistol	doll	whiskey*	fork	pot

harmonica	can opener*	sword	pencil	Gun*	vodka
knife*	rum	trout	bomb	pan	gold
wine	lemon	spatula	Bourbon	beer *	rifle

Form 3 word list (semantic categories: musical instruments, fuels, food flavourings):
Free Recall: sugar, trumpet, violin, coal, garlic, kerosine, vanilla, wood, clarinet, flute, cinnamon, gasoline
Recognition:

Pepper*	Garlic	Wood	Drum*	Oil*	Sugar
Harmonica	Salt*	Priest	Chair	Coal	Clarinet
Trumpet	Basement	Cinnamon	Flute	Electricity*	Moon
Kerosine	Vanilla	Gasoline	Sand	Piano*	Violin

Form 4 word list (semantic categories: birds, clothing, carpenter's tools):
Free Recall: canary, shoes, eagle, blouse, nails, crow, bluebird, screwdriver, pants, chisel, skirt, wrench
Recognition:

Bluebird	Shirt*	Chisel	Eagle	Chocolate	Robin*
Chapel	Screwdriver	Crow	Sparrow*	Wrench	Pants
Nails	Socks*	Child	Shoes	Hair	Hammer*
Canary	Apple	Skirt	Saw*	Silver	Blouse

Form 5 word list (semantic categories: occupations/professions, sports, vegetables):
Free Recall: teacher, basketball, lettuce, dentist, tennis, bean, engineer, potato, professor, golf, corn, soccer
Recognition:

Tennis	Football*	Professor	Spinach*	Lawyer*	Submarine
Golf	Dentist	Lettuce	Spider	Water	Bean
Basketball	Doctor*	Corn	Baseball*	Teacher	Snake

Carrot*	Engineer	Glove	Soccer	Potato	tulip

Form 6 word list (semantic categories: fish, parts of a building, weather):
Free Recall: shark, wall, herring, rain, floor, hail, catfish, roof, salmon, storm, ceiling, snow
Recognition:

Hail	Bass*	Snow	Bank	Floor	Mustard
Window*	Ceiling	Canyon	Rain	Ladder	Storm
Herring	Salmon	Tornado*	Trout*	Melon	Roof
Shark	Hurricane*	Elbow	Catfish	Wall	Door*

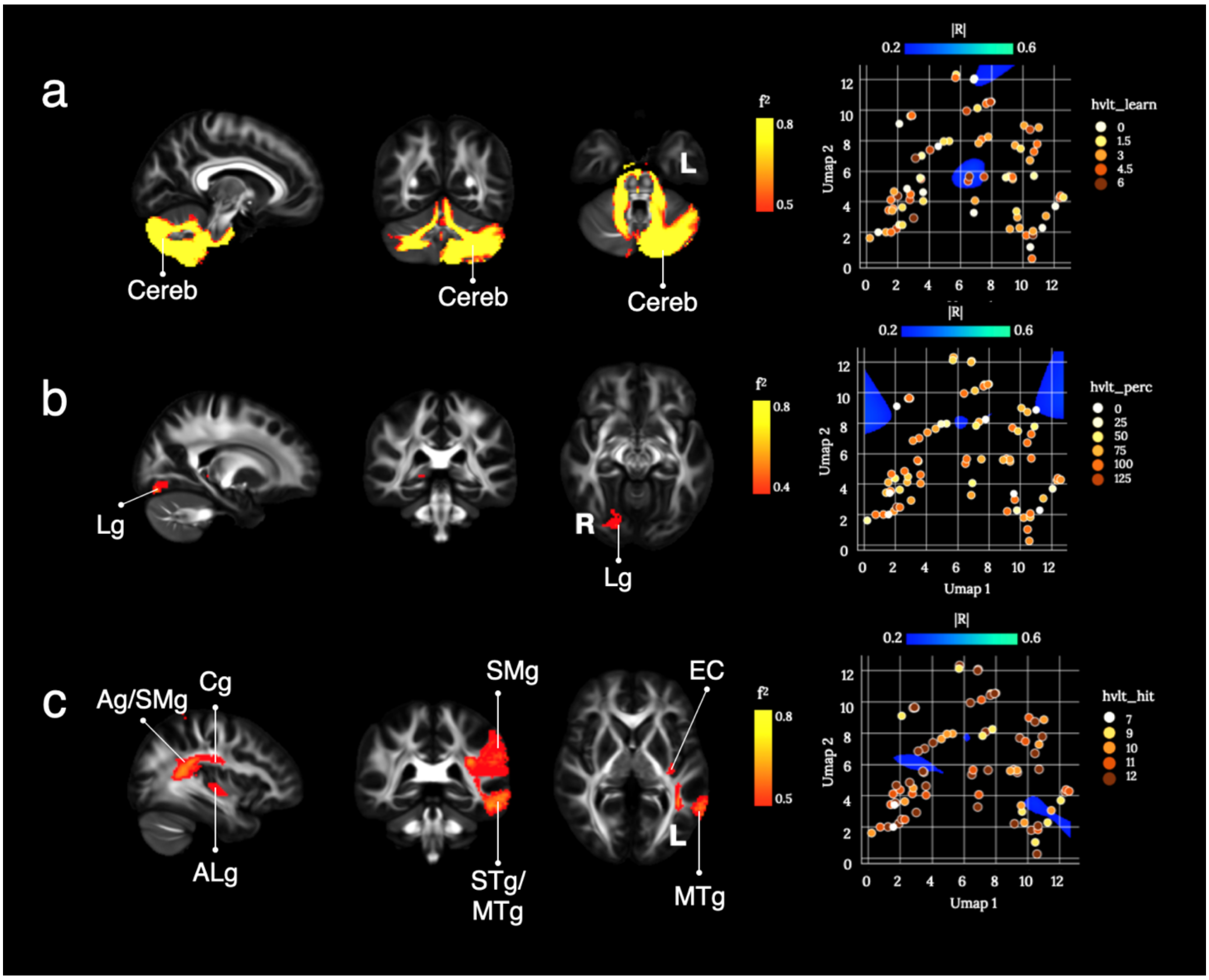

Supplementary Figure 36: Brain disconnections and UMAP related territories contributing significantly to the Hopkins Verbal Learning Test (HVLT) correct responses. (a) learning (hvlt_learn), (b) percent retained (hvlt_perc), and (c) recognition hits (hvlt_hit). Ag: Angular gyrus; ALg: Anterior Long insular gyrus; Cereb: Cerebellum; Cg: Cingulate gyrus; EC: External/Extreme Capsule; Lg: Lingual gyrus; MTg: Middle Temporal gyrus; SMg: Supra Marginal gyrus; STg: Superior Temporal gyrus. Maps are freely available at https://neurovault.org/collections/11260/ .

Functional topography and clinical case studies have previously linked the cerebellum to verbal working memory [40,41], for a review see [42]. In a more recent consensus paper the emerging field of cerebellar neurocognition has been discussed in light of language processing [43]. The lingual gyrus is classically associated with reading e.g. [44] which might indicate a learning strategy whereby the heard words might have been visualised for encoding. Numerous investigations in healthy participants and patient cohorts have demonstrated the importance of the inferior parietal lobe (angular and supramarginal gyri) for verbal working memory by highlighting individual processes such as the phonological loop, sensorimotor integration of speech, representation of phonetic sequences, attentional capture of verbal information, and phonological retrieval [45-49].
A decline in learning and memory has been described for patients with insular tumours [50].

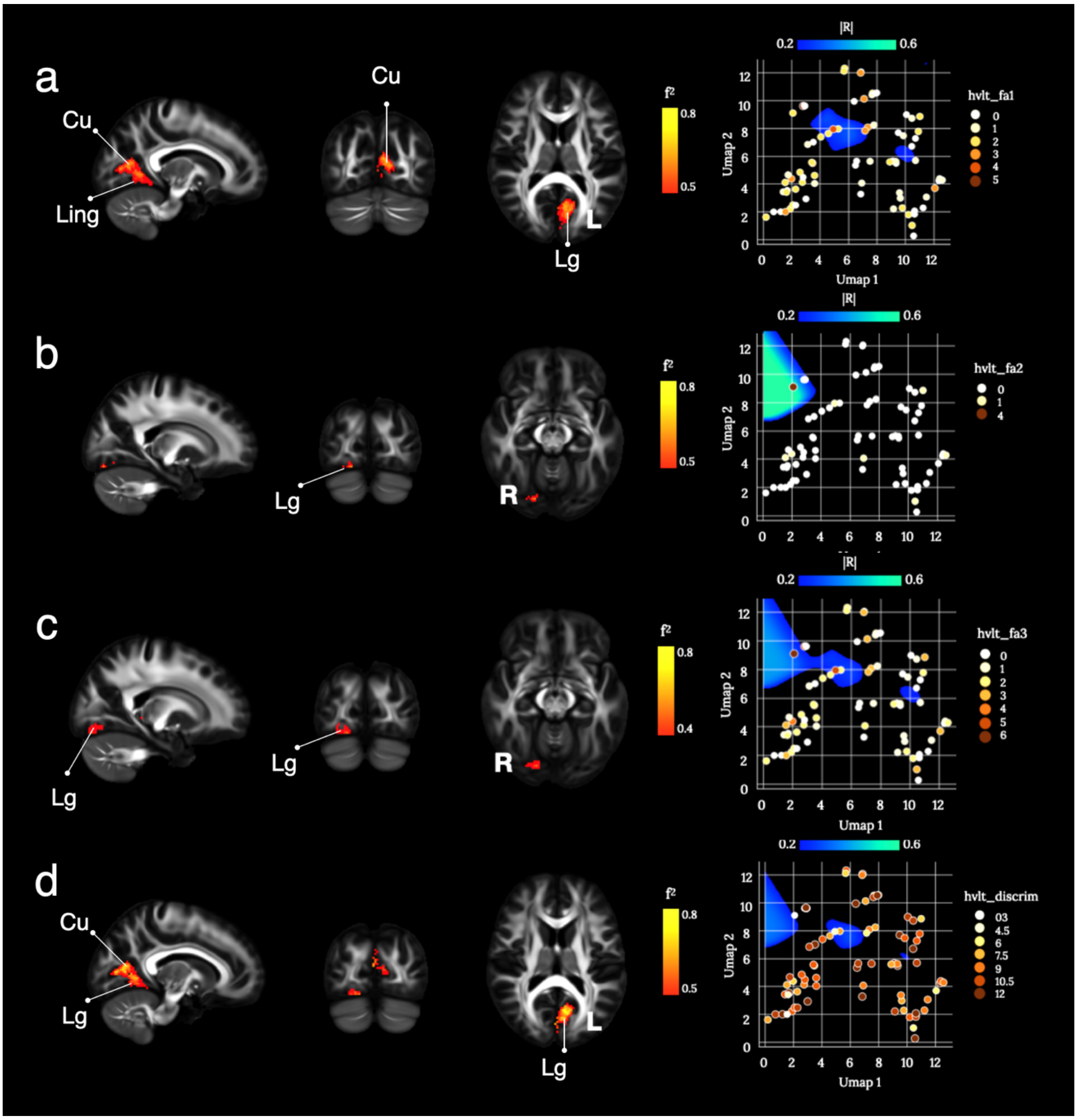

Supplementary Figure 37: Brain disconnections and UMAP related territories contributing significantly to the Hopkins Verbal Learning Test (HVLT) false-positive responses. (a) false-positives related (hvlt_fa1), (b) false-positives unrelated (hvlt_fa2), (c) total false positives (hvlt_fa3), and (d) the recognition discremination index (hvlt_discrim). Cu: Cuneus; Lg: Lingual gyrus. Maps are freely available at https://neurovault.org/collections/11260/ .

According to the composite morphospace results, profiles of disconnections predicting individual false-positive responses and item discrimination were localized in the bilateral lingual gyri and the left cuneus. The lingual gyrus has been implicated in the identification and recognition of words [51]. It has also been linked to visual imagery, which might be an encoding and retrieval strategy during the discrimination tasks [52].

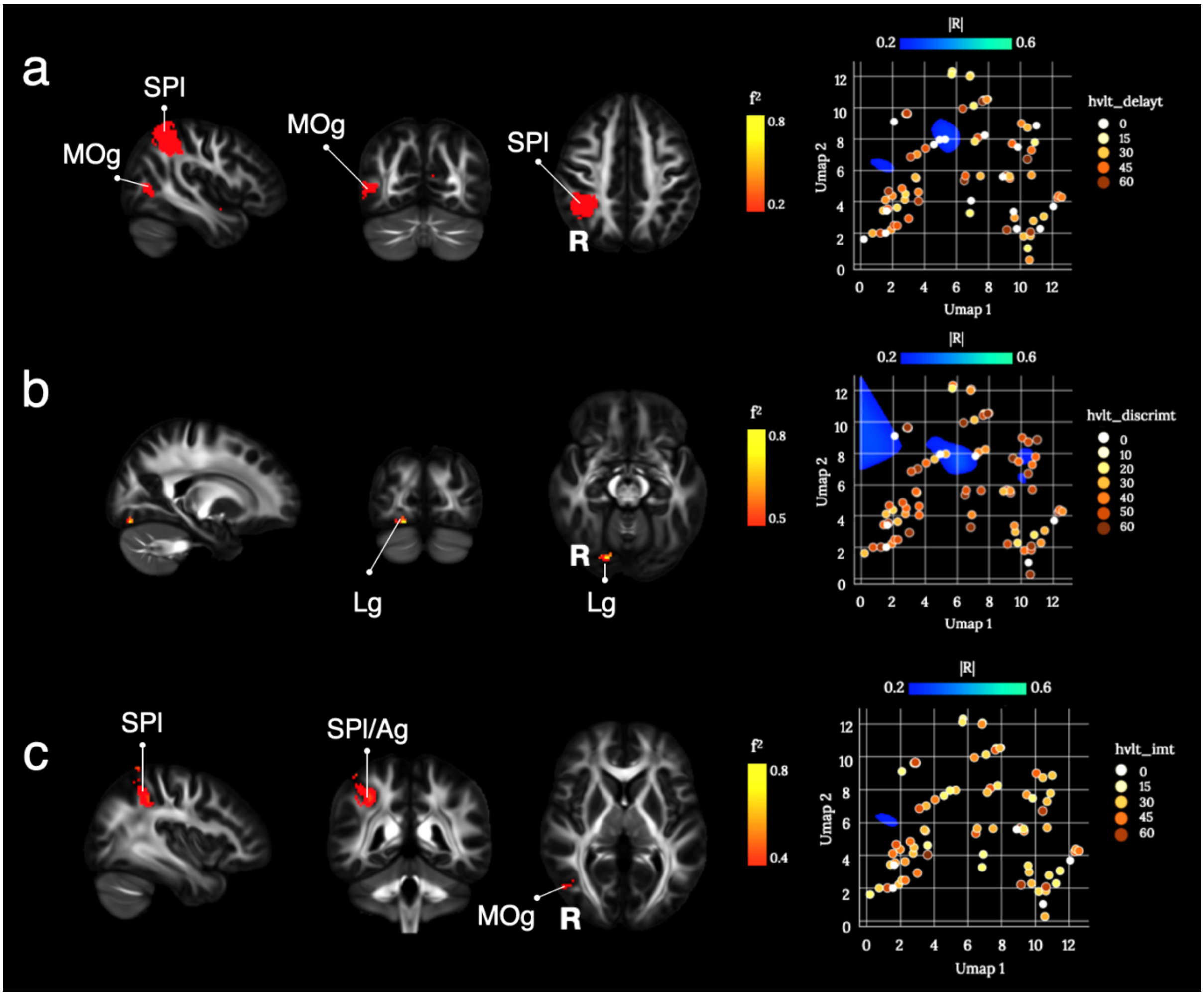

Supplementary Figure 38: Brain disconnections and UMAP related territories contributing significantly to the Hopkins Verbal Learning Test (HVLT) responses after t-score conversion. (a) delayed recall (hvlt_delayt), (b) discrimination index (hvlt_discrimt), and (c) total immediate recall (hvlt_imt). Ag: Angular gyrus; Cu: Cuneus; Lg: Lingual gyrus; MOg: Middle Occipital gyrus; SPl: Superior Parietal lobule. Maps are freely available at https://neurovault.org/collections/11260/ .

Anatomically, the parietal lobe is at the crossroad between the frontal, occipital, and temporal lobes and highly connected to each lobe. This connectivity pattern results in the parietal lobe being a central hub for multimodal sensory integration. Functional studies have indicated its role in higher cognitive functions that are characteristic of the human species, including semantic and pragmatic aspects of language, episodic retrieval, memory integration, and sustained attention [53-58]. Semantic activations were primarily described for the left hemisphere inferior parietal lobe but have been consistently shown for the right angular gyrus as well [59]. Given the high semantic load of these word lists, a disconnection of the superior parietal lobe can lead to deficits in the immediate (hvlt_imt) and delayed (hvlt_delayt) recall.

C.6 Pain

Pain is one of the long-term compliances that can manifest after a stroke event. Pain incidence can vary across the stroke survivor population. Lundström et al. (2009) estimated a stroke-related pain incidence of 21% at 1-year after the stroke event (N=140 stroke patients). Langhorne et al. (2000) rated a pain incidence of 34%, and more specifically a shoulder pain of 9 %, in patients up to 30 months after stroke (N=311 stroke patients).
Moreover, pain perception significantly increases in the MRI scanner environment, probably due to higher awareness and alertness, as shown by Ellerbrock et al (2015) in a healthy population.
In our study, pain sensation after the MRI scanning was recorded. A scale ranging between 0-100 was used, and patients had to answer the following question: "During the scan, how much of the time did you... Feel pain or discomfort?". A multiple choice of answers was presented:

- all of the time (100%);
- most of the time (80%);
- a good bit of the time (60%);
- some of the time (40%);
- a little of the time (20%);
- none of the time (0%).

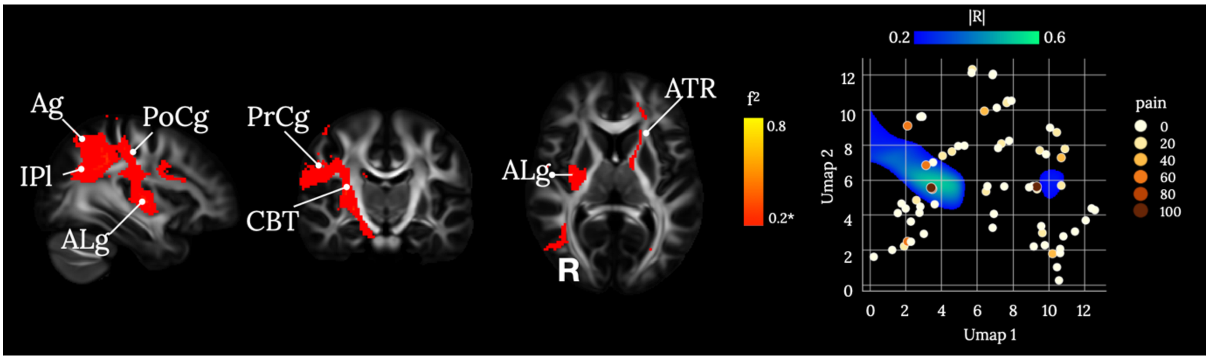

Supplementary Figure 39. Brain disconnections and UMAP related territories contributing significantly to the pain score. Ag: Angular gyrus; ALg: Anterior Long insular gyrus; ATR: Anterior Thalamic Radiation; CBT: Cortico Bulbar Tract; IPl: Inferior Parietal lobe; PoCg: Post-Central gyrus; PrCg: Pre-Central gyrus. * Indicate a medium effect size ($0.15 < f^2$). Maps are freely available at https://neurovault.org/collections/11260/ .

According to the composite morphospace results, profiles of disconnections predicting individual pain perception were localized mainly in the right white matter; including pathways reaching the parietal lobe, posterior insula, post-central gyrus and the lower division of the precentral gyrus. In the left hemisphere, correlations occurred along with the anterior thalamic radiation.
As reviewed by Tracey and Mantyh [60], brain perception is modulated between the peripheral nervous system and cerebral pain processing. In particular, the nociceptive information ascends to the thalamus along the contralateral spinothalamic tract, medulla, and brainstem. The descending pain modulatory system also includes projections to the amygdala, hypothalamus, insula, and anterior cingulate cortex. Spinal projections to the brainstem are also extremely important [60]. Our findings are in agreement with the described nociceptive system, highlighting pyramidal connections with the lower portion of the precentral gyrus (cortico bulbar tract fibers), and the postcentral gyrus connected by the spinothalamic tract mainly involved in the somatosensory responses to pain [61].

In the disconnectome results the right posterior insula and left anterior thalamic radiation were also highlighted.
Our pain related results have a medium effect size ($0.15< f^2$), probably reflecting the brief pain examination conducted. However, the found pain correlates present encouraging results.

C.7 Sickness

The Sickness Impact Profile (SIP) scale is a commonly used scale to assess the quality of life [62]. The SIP scale is composed of 136 items divided into 12 subscales, exploring three main aspects: physical, social, and emotional functioning. Van Staten et al. [63] proposed a shorter and stroke-adapted version of the SIP (SA-SIP) including 30 items subdivided into 8 subscales exploring both physical and psychosocial sickness dimensions. Using the SA-SIP it was possible to explain the 91% of the SIP evaluation variance in stroke patients (N=319, 6-months after stroke onset). This result was replicated in an independent cohort explaining 89% of the sickness profile variance [63]. The SA-SIP scale has been also translated in different languages than English (e.g., French, Spanish) for a wider use.

<u>Sip_body</u>
The sip_body constitutes the total score for the sickness of body care and movement.
It has been evaluated with a yes/no answer to the following five questions:

- I make difficult moves with help, for example getting into or out of cars, bathtubs (sip_body1);
- I move my hands or fingers with some limitation or difficulty (sip_body2);
- I get in and out of bed or chairs by grasping something for support or using a cane or walker (sip_body3);
- I have trouble getting shoes, socks, or stockings on (sip_body4);
- I get dressed only with someone's help (sip_body5).

The total sickness of body care and movement score ranges from 0 to 100, and it is calculated as:

sip_body = (sip_body1*84+ sip_body2*64 + sip_body3*82 + sip_body4*57 + sip_body5*88)/3.75

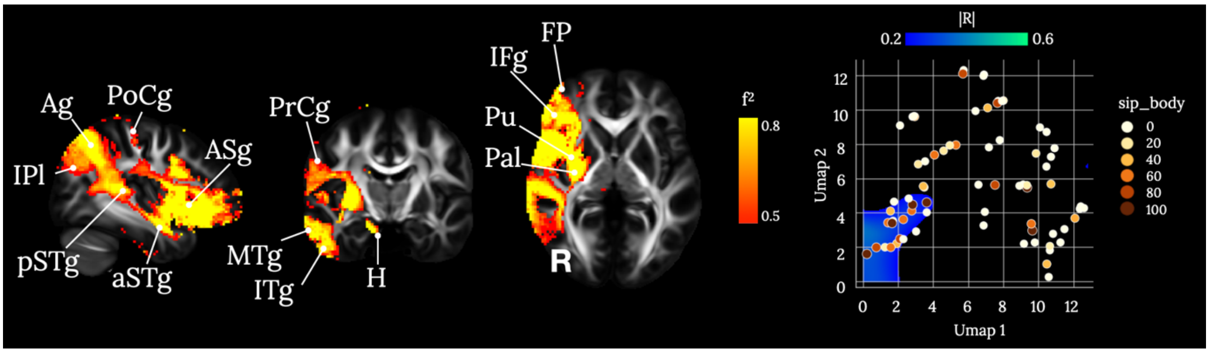

Supplementary Figure 40. Brain disconnections and UMAP related territories contributing significantly to the sickness of body care and movement score (sip_body). Ag: Angular gyrus; Anterior Short insular gyrus: ASg: Anterior Short insular gyrus; aSTg: anterior Superior Temporal gyrus; FP: Frontal Pole; H: Hippocampus; IFg: Inferior Frontal gyrus; IPl: Inferior Parietal lobe; ITg: Inferior Temporal gyrus; MTg: Middle Temporal gyrus; Pal: Pallidum; PoCg: Post-Central gyrus; PrCg: Pre-Central gyrus; pSTg: posterior Superior Temporal gyrus; Pu: Putamen. Maps are freely available at https://neurovault.org/collections/11260/ .

Sip_mob

The sip_mob constitutes the total score for mobility sickness. It has been evaluated with a yes/no answer to the following three questions:

- I stay home most of the time (sip_mob1);
- I am not going into town (sip_mob2);
- I do not get around in the dark or in unlit places without someone's help (sip_mob3).

The total score for the mobility sickness ranges from 0 to 100, and it is calculated as:

sip_mob = (sip_mob1*66 + sip_mob2*48 + mob3*72)/1.86

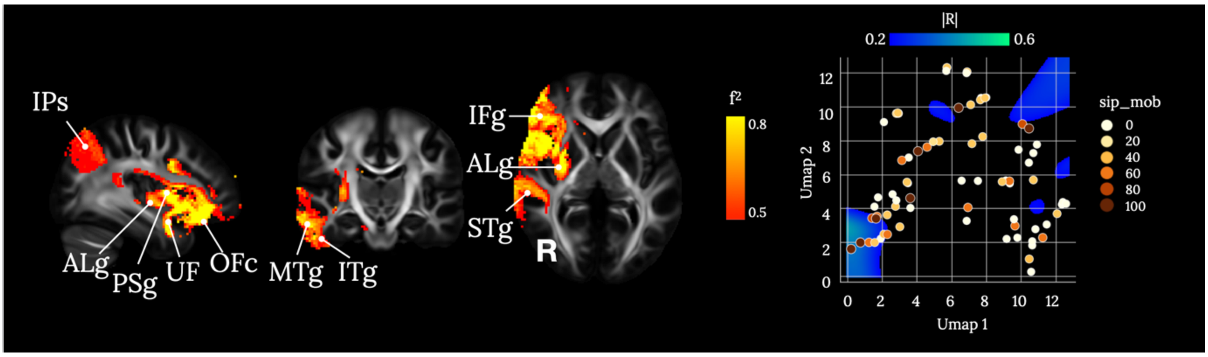

Supplementary Figure 41. Brain disconnections and UMAP related territories contributing significantly to the total score for mobility sickness (sip_mob). ALg: Anterior Long insular gyrus; IFg: Inferior Frontal gyrus; IPs: Intra Parietal sulcus; ITg: Inferior Temporal gyrus; MTg: Middle Temporal gyrus; OFc: Orbito Frontal cortex; PSg: Posterior Short insular gyrus; STg: Superior Temporal gyrus; UF: Uncinate Fasciculus. Maps are freely available at https://neurovault.org/collections/11260/ .

Sip_house

The sip_house constitutes the total score for the household management sickness. It has been evaluated with a yes/no answer to the following four questions:

- I am not doing any of the maintenance or repair work that I would usually do in my home or yard (sip_house1);
- I am not doing any of the shopping that I would usually do (sip_house2);
- I am not doing any of the house cleaning that I would usually do (sip_house3);
- I am not doing any of the clothes washing that I would usually do (sip_house4).

The total score for the household management sickness ranges from 0 to 100, and it is calculated as:

sip_house = (sip_house1*62 + sip_house2*71 + sip_house3*77 + sip_house4*77)/2.87

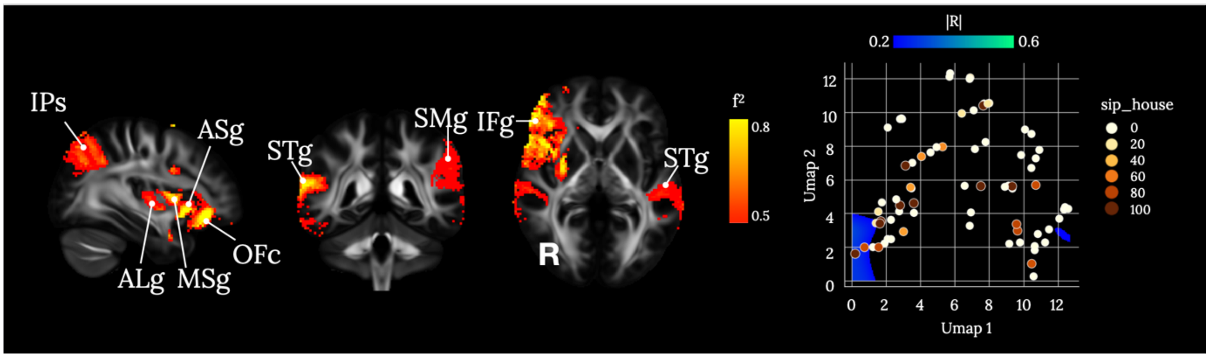

Supplementary Figure 42. Brain disconnections and UMAP related territories contributing significantly to the household management sickness score (sip_house). ALg: Anterior Long insular gyrus; ASg: Anterior Short insular gyrus; IFg: Inferior Frontal gyrus; IPs: Intra Parietal sulcus; MSg: Midddle Short insular gyrus; OFc: Orbito Frontal cortex; SMg: Supra Marginal gyrus; STg: Superior Temporal gyrus; STg: Superior Temporal gyrus. Maps are freely available at https://neurovault.org/collections/11260/ .

Sip_amb

The sip_amb constitutes the total score for ambulation sickness evaluated with a yes/no answer to the following three questions:

- I do not walk up or down hills (sip_amb1);
- I get around only by using a walker, crutches, cane, walls, or furniture (sip_amb2);
- I walk more slowly (sip_amb3).

The total sickness of ambulation score ranges from 0 to 100, and it is calcualted as:

sip_amb = (sip_amb1*56 + sip_amb2*79 + sip_amb3*35)/1.7

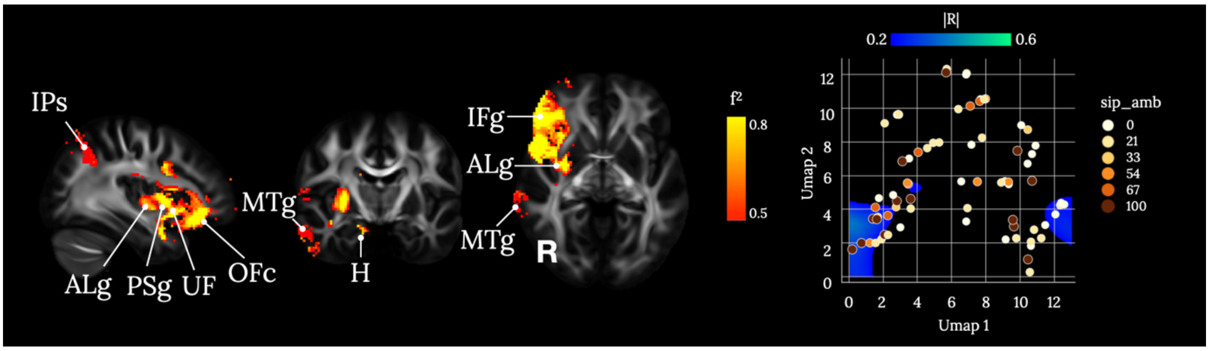

Supplementary Figure 43. Brain disconnections and UMAP related territories contributing significantly to the total score for ambulation sickness (sip_amb). ALg: Anterior Long insular gyrus; H: Hippocampus; IFg: Inferior Frontal gyrus; IPs: Intra Parietal sulcus; MTg: Middle Temporal gyrus; OFc: Orbito Frontal cortex; PSg: Posterior Short insular gyrus ; UF: Uncinate Fasciculus. Maps are freely available at https://neurovault.org/collections/11260/ .

Sip_physical

The sip_physical is a subtotal scale evaluating the physical sickness dimension. The sip_physical score combines together the sickness scores obtained for body care and movement (sip_body), mobility (sip_mob), household management (sip_house), and ambulation (sip_amb).

The physical sickness score ranges from 0 to 100, and it is calculated as:

sip_physical= (sip_body1*84 + sip_body2*64 + sip_body3*82 + sip_body4*57 + sip_body5*88 + sip_mob1*66 + sip_mob2*48 +sip_mob3*72 + sip_house1*62 + sip_house2*71 + sip_house3*77 + sip_house4*77 +sip_amb1*56 + sip_amb2*79 + sip_amb3*35)/10.18

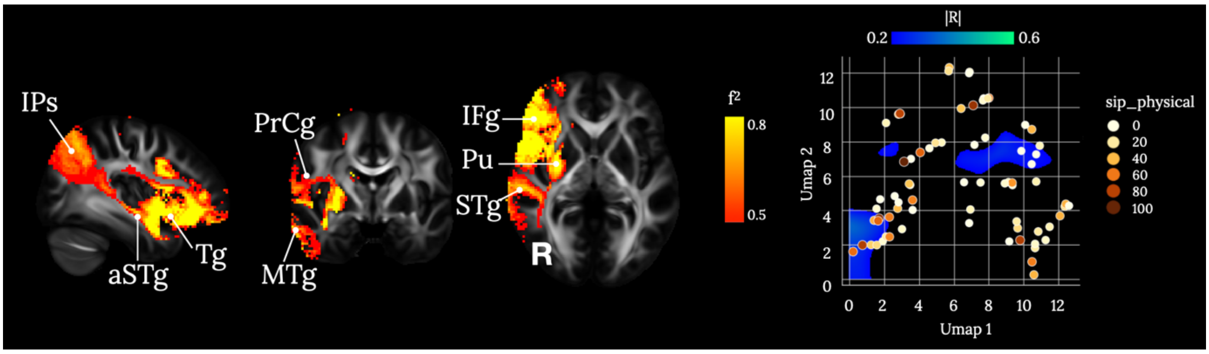

Supplementary Figure 44. Brain disconnections and UMAP related territories contributing significantly to the subtotal scale evaluating the physical sickness dimension (sip_physical). aSTg: anterior Superior Temporal gyrus; IFg: Inferior Frontal gyrus; IPs: Intra Parietal sulcus; MTg: Middle Temporal gyrus; PrCg: Pre-Central gyrus; Pu: Putamen; STg: Superior Temporal gyrus; Tg: Transverse insular gyrus. Maps are freely available at https://neurovault.org/collections/11260/ .

Sip_social

The sip_social constitutes the total score for the social interaction sickness. It has been evaluated with a yes/no answer to the following five questions:

- I show less interest in other people's problems, for example, don't listen when they tell me about their problems, don't offer to help (sip_social1);
- I often act irritable to those around me, for example, snap at people, give sharp answers, criticize easily (sip_social2);
- I show less affection (sip_social3);
- I am doing fewer social activities with groups of people (sip_social4);
- I talk less to those around me (sip_social5).

The total score of social interaction sickness ranges from 0 to 100, and it is calculated as:

sip_social = (sip_social1*67 + sip_social2*84 + sip_social3*52 + sip_social4*36 + sip_social5*56)/2.95

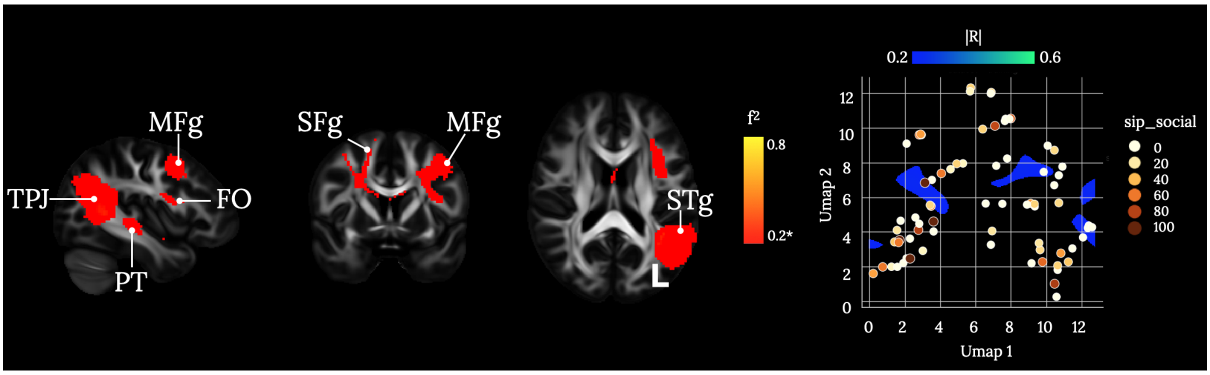

Supplementary Figure 45. Brain disconnections and UMAP related territories contributing

significantly to the total score for the social interaction sickness (sip_social). FO: Frontal Operculum; MFg: Middle Frontal gyrus; PT: Planum Temporale; SFg: Superior Frontal gyrus; STg: Superior Temporal gyrus; TPJ: Temporal Parietal Junction. * Indicates a medium effect size ($0.15<f^2<0.35$). Maps are freely available at https://neurovault.org/collections/11260/ .

Sip_com

The sip_com constitutes the total score for the sickness of communication. It has been evaluated with a yes/no answer to the following three questions:

- I carry on a conversation only when very close to the other person or looking at him (sip_com1);
- I have difficulty speaking, for example, get stuck, stutter, stammer, slur my words (sip_com2);
- I do not speak clearly when I am under stress (sip_com3).

The total communication sickness score ranges from 0 to 100, and it is calculated as:

sip_com = (sip_com1*67 + sip_com2*76 + sip_com3*64)/2.07

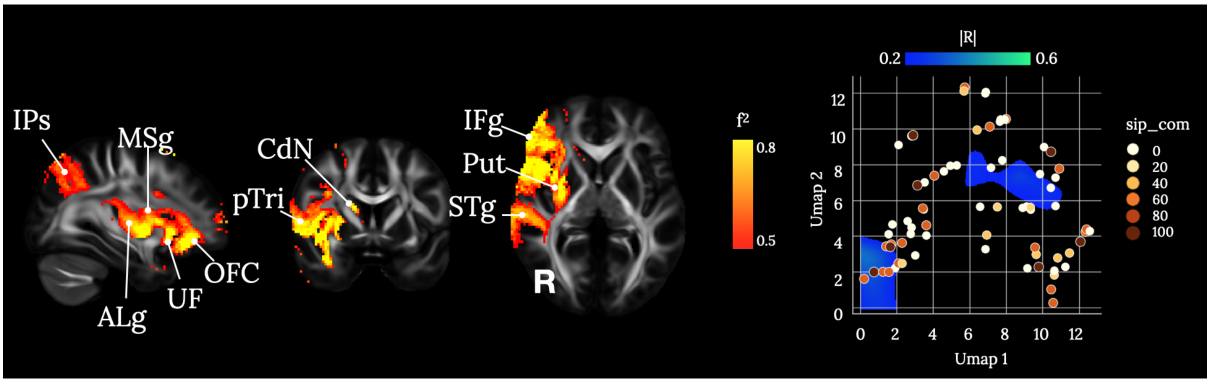

Supplementary Figure 46. Brain disconnections and UMAP related territories contributing significantly to the total score for the sickness of communication (sip_com). ALg: Anterior Long insular gyrus; C: Caudate; IFg: Inferior Frontal gyrus; IPs: Intra Parietal sulcus; MSg: Midddle Short insular gyrus; OFc: Orbito Frontal cortex; Pu: Putamen; STg: Superior Temporal gyrus; Tr: pars Triangularis; UF: Uncinate Fasciculus. Maps are freely available at https://neurovault.org/collections/11260/ .

Sip_emo

The sip_emo constitutes the total score for the sickness of emotional behaviour. It is evaluated with a yes/no answer to the following four questions:

- I say how bad or useless I am, for example, that I am a burden on others (sip_emo1);
- I laugh or cry suddenly (sip_emo2);
- I act irritable and impatient with myself, for example, talk badly about myself, swear at myself, blame myself for things that happen (sip_emo3);
- I get sudden frights (sip_emo4).

The total sickness of emotional behaviour score ranges from 0 to 100, and it is calculated as:

sip_emo = (sip_emo1*87 + sip_emo2*68 + sip_emo3*78 + sip_emo4*74)/3.07

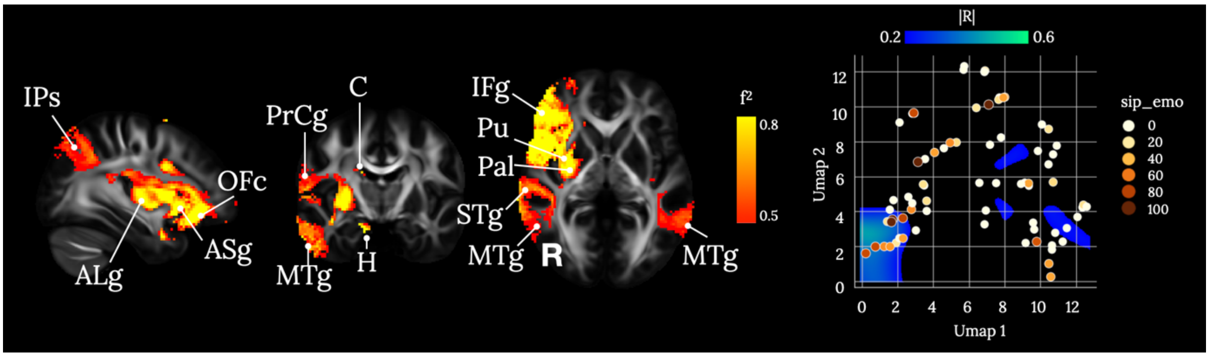

Supplementary Figure 47. Brain disconnections and UMAP related territories contributing significantly to the total score for the sickness of emotional behaviour (sip_emo). ALg: Anterior Long insular gyrus; ASg: Anterior Short insular gyrus; C: Caudate; H: Hippocampus; IFg: Inferior Frontal gyrus; IPs: Intra Parietal sulcus; MTg: Middle Temporal gyrus; OFc: Orbito Frontal cortex; Pal: Pallidum; PrCg: Pre-Central gyrus; Pu: Putamen; STg: Superior Temporal gyrus. Maps are freely available at https://neurovault.org/collections/11260/ .

Sip_alert

The sip_alert constitutes the total score for alertness behaviour evaluated with a yes/no answer to the following three questions:

- I am confused and start several actions at a time (sip_alert1);
- I make more mistakes than usual (sip_alert2);
- I have difficulty doing activities involving concentration and thinking (sip_alert3).

The total sickness of alertness behaviours ranges from 0 to 100, and it is calculated as:

sip_alert = (sip_alert1*90 + sip_alert2*64 + sip_alert3*80)/2.34

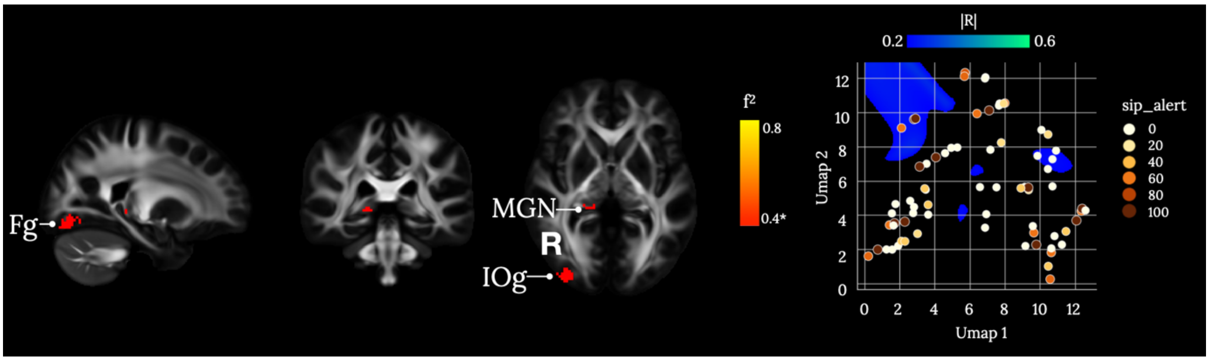

Supplementary Figure 48. Brain disconnections and UMAP related territories contributing significantly to the total score for alertness behaviour (sip_ alert). Fg: Fusiform gyrus; IOg: Inferior Occipital gyrus; MGN: Middle Geniculate Nucleus. * Indicates a large effect size ($0.35<f^2$). Maps are freely available at https://neurovault.org/collections/11260/ .

Sip_psychosoc

The sip_psychosoc is a subtotal scale evaluating the psychosocial sickness dimension. The sip_psychosoc score combines together the different sickness scores obtained for social (sip_social), communication (sip_com), emotional behavior (sip_emo), and alertness behavior (sip_alert).

The psychosocial sickness score ranges from 0 to 100, and it is calculated as:

sip_psychosoc = (sip_soc1*67+ sip_soc2*84+ sip_soc3*52+ sip_soc4*36+ sip_soc5*56+ sip_com1*67+ sip_com2*76+ sip_com3*64+ sip_emo1*87+ sip_emo2*68+ sip_emo3*78+ sip_emo4*74+ sip_alert1*90+ sip_alert2*64+ sip_alert3*80) /10.43.

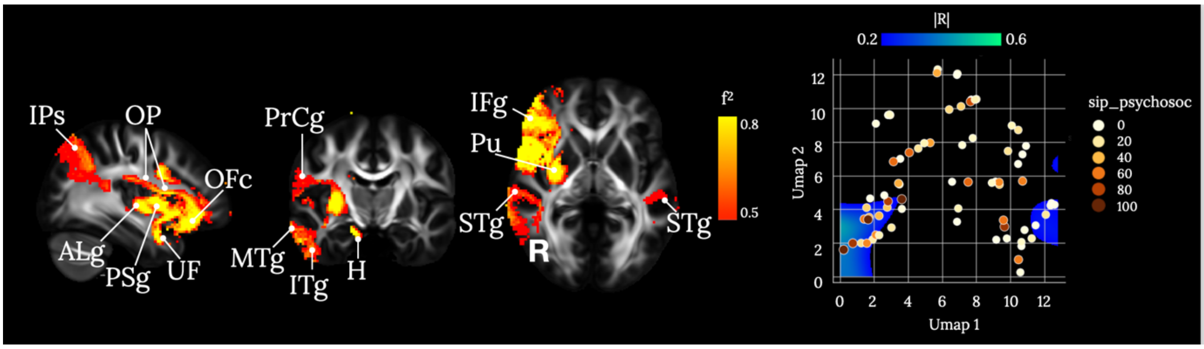

Supplementary Figure 49. Brain disconnections and UMAP related territories contributing significantly to the psychosocial sickness dimension (sip_psychosoc). ALg: Anterior Long insular gyrus; H: Hippocampus; IFg: Inferior Frontal gyrus; IPs: Intra Parietal sulcus; ITg: Inferior Temporal gyrus; MTg: Middle Temporal gyrus; OFc: Orbito Frontal cortex; Op: Operculum; PSg: Posterior Short insular gyrus; PrCg: Pre-Central gyrus; Pu: Putamen; STg: Superior Temporal gyrus; UF: Uncinate Fasciculus. Maps are freely available at https://neurovault.org/collections/11260/ .

The disconnectome analyses highlighted the basal ganglia (e.g., putamen, pallidum and caudate body) on the right hemisphere as predictors for most of the sickness scores. In agreement, Van de Port et al. [64], administering the SA-SIP scale found lower scores in total and psychosocial dimensions in subarachnoid haemorrhage, and subcortical infarction compared to cortical strokes (N=122 stroke patients). In the paper by Van Straten et al. [63], in which the SA-SIP scale was originally defined, no differences were found distinguishing infratentorial and supratentorial strokes, whereas lacunar infarctions presented a better overall sickness response compared to cortical and subcortical strokes. The disconnectome analyses did not highlight any infratentorial, internal capsule or thalamic areas.
In our disconnectome results, the insular gyri presented large effect size correlations in all the evaluated sickness scores with exception for the social and alertness behaviours. The insular correlations were strongly lateralized on the right hemisphere. Interestingly, in the study of Harte et al. [65], investigating patients with a chronic pain condition, fibromyalgia, insular activations in response to visual stimulation were able to discriminate between patients versus healthy participants. In particular, activation in the right anterior insula. Thus, it indicated that a dysregulation of multisensory integration within the right insula can lead to pain conditions, and probably to a higher sickness response.
In the study by Fogel et al. (2011), investigating chronic pain in temporomandibular patients, a decoupling between the prefrontal cortex and the cingulate cortex was measured during cognitive and emotional tasks. These findings suggest that an asynchronization between attention and cognition processing can lead to slow behavioural responses, evaluated in several sickness scores (e.g., sip_psychosoc). In the disconnectome results, the right prefrontal cortex was highly correlated with sickness in all the evaluated scores.
Additionally, the uncinate fasciculus, connecting the orbital prefrontal cortex, is strongly involved in social-emotional processing, presenting abnormalities mainly in the right hemisphere in psychopathy and antisocial personality disorders [66]. The disconnectome results also detect right parietal, temporal and hippocampal regions, and left temporal regions as predictors correlated with sickness. In the study by Shigihara et al. [67], using magnetoencephalography and mental fatigue conditions, decreased alpha power was measured in the right angular gyrus and increased levels in the left middle and superior temporal gyri, after a 30 minute fatigue inducing 0-back test sessions; whereas after a 2-back session, decreased alpha power was measured in right middle and superior frontal gyrus, and widespread increased levels including the right parahippocampal gyrus, right inferior frontal gyrus and left middle

temporal gyrus. These regions were also highlighted in our disconnectome results, and indicate that different types of mental fatigue can produce alterations in distinct brain regions, and with different levels of activity oscillations.

As a limitation, the SA-SIP scale has to be administered with caution to patients presented with a severe stroke, and in case of severe speech and language disorders. In those cases, a proxy respondent, primarily the partner, will answer the questions [68]. Thus, the left hemisphere, mainly damaged in case of language impairment, can be underestimated as neuronal correlates of sickness using the SA-SIP scale.

Supplementary references

1. Benedict RHB, Schretlen D, Groninger L, Dobraski M, Shpritz B. Revision of the Brief Visuospatial Memory Test: Studies of normal performance, reliability, and validity. *Psychological Assessment*. 1996;8(2):145–153.
2. Konishi S, Wheeler ME, Donaldson DI, Buckner RL. Neural Correlates of Episodic Retrieval Success. *NeuroImage*. 2000/09/01/ 2000;12(3):276-286. doi:https://doi.org/10.1006/nimg.2000.0614
3. McDermott KB, Jones TC, Petersen SE, Lageman SK, Roediger HL, III. Retrieval Success is Accompanied by Enhanced Activation in Anterior Prefrontal Cortex During Recognition Memory: An Event-Related fMRI Study. *Journal of Cognitive Neuroscience*. 2000;12(6):965-976. doi:10.1162/08989290051137503
4. Ciaramelli E, Grady CL, Moscovitch M. Top-down and bottom-up attention to memory: A hypothesis (AtoM) on the role of the posterior parietal cortex in memory retrieval. *Neuropsychologia*. 2008/06/01/ 2008;46(7):1828-1851. doi:https://doi.org/10.1016/j.neuropsychologia.2008.03.022
5. Vilberg KL, Rugg MD. Memory retrieval and the parietal cortex: A review of evidence from a dual-process perspective. *Neuropsychologia*. 2008/06/01/ 2008;46(7):1787-1799. doi:https://doi.org/10.1016/j.neuropsychologia.2008.01.004
6. Cabeza R, Ciaramelli E, Olson IR, Moscovitch M. The parietal cortex and episodic memory: an attentional account. *Nature Reviews Neuroscience*. 2008/08/01 2008;9(8):613-625. doi:10.1038/nrn2459
7. Papagno C. Chapter 19 - Memory deficits. In: Vallar G, Coslett HB, eds. *Handbook of Clinical Neurology*. Elsevier; 2018:377-393.
8. Melrose RJ, Zahniser E, Wilkins SS*, et al*. Prefrontal working memory activity predicts episodic memory performance: A neuroimaging study. *Behavioural Brain Research*. 2020/02/03/ 2020;379:112307. doi:https://doi.org/10.1016/j.bbr.2019.112307
9. Goldenberg G. Loss of visual imagery and loss of visual knowledge—A case study. *Neuropsychologia*. 1992/12/01/ 1992;30(12):1081-1099. doi:https://doi.org/10.1016/0028-3932(92)90100-Z
10. Moro V, Berlucchi G, Lerch J, Tomaiuolo F, Aglioti SM. Selective deficit of mental visual imagery with intact primary visual cortex and visual perception. *Cortex*. 2008/02/01/ 2008;44(2):109-118. doi:https://doi.org/10.1016/j.cortex.2006.06.004
11. Spagna A, Hajhajate D, Liu J, Bartolomeo P. Visual mental imagery engages the left fusiform gyrus, but not the early visual cortex: A meta-analysis of neuroimaging evidence. *Neuroscience & Biobehavioral Reviews*. 2021/03/01/ 2021;122:201-217. doi:https://doi.org/10.1016/j.neubiorev.2020.12.029
12. Ferrari C, Cattaneo Z, Oldrati V*, et al*. TMS Over the Cerebellum Interferes with Short-term Memory of Visual Sequences. *Scientific Reports*. 2018/04/30 2018;8(1):6722. doi:10.1038/s41598-018-25151-y

13. Thompson RF, Kim JJ. Memory systems in the brain and localization of a memory. *Proceedings of the National Academy of Sciences*. 1996;93(24):13438. doi:10.1073/pnas.93.24.13438
14. Kitazawa S, Kimura T, Yin P-B. Cerebellar complex spikes encode both destinations and errors in arm movements. *Nature*. 1998/04/01 1998;392(6675):494-497. doi:10.1038/33141
15. Andreasen NC, O'Leary DS, Paradiso S*, et al*. The cerebellum plays a role in conscious episodic memory retrieval. https://doi.org/10.1002/(SICI)1097-0193(1999)8:4<226::AID-HBM6>3.0.CO;2-4. *Human Brain Mapping*. 1999/01/01 1999;8(4):226-234. doi:https://doi.org/10.1002/(SICI)1097-0193(1999)8:4<226::AID-HBM6>3.0.CO;2-4
16. Addis DR, Moloney EEJ, Tippett LJ, P. Roberts R, Hach S. Characterizing cerebellar activity during autobiographical memory retrieval: ALE and functional connectivity investigations. *Neuropsychologia*. 2016/09/01/ 2016;90:80-93. doi:https://doi.org/10.1016/j.neuropsychologia.2016.05.025
17. Dave S, VanHaerents S, Voss JL. Cerebellar Theta and Beta Noninvasive Stimulation Rhythms Differentially Influence Episodic Memory versus Semantic Prediction. *The Journal of Neuroscience*. 2020;40(38):7300. doi:10.1523/JNEUROSCI.0595-20.2020
18. Lassonde MC, Sauerwein HC, Lepore F. Agenesis of the corpus callosum. In: Zaidel E, Iacoboni M, eds. *The parallel brain: The cognitive neuroscience of the corpus callosum*. MIT Press; 2003:357–369.
19. Paul LK, Erickson RL, Hartman JA, Brown WS. Learning and memory in individuals with agenesis of the corpus callosum. *Neuropsychologia*. 2016/06/01/ 2016;86:183-192. doi:https://doi.org/10.1016/j.neuropsychologia.2016.04.013
20. Berlucchi G, Aglioti S, Marzi CA, Tassinari G. Corpus callosum and simple visuomotor integration. *Neuropsychologia*. 1995/08/01/ 1995;33(8):923-936. doi:https://doi.org/10.1016/0028-3932(95)00031-W
21. Eliassen JC, Baynes K, Gazzaniga MS. Anterior and posterior callosal contributions to simultaneous bimanual movements of the hands and fingers. *Brain*. 2000;123(12):2501-2511. doi:10.1093/brain/123.12.2501
22. Martino J, Brogna C, Robles SG, Vergani F, Duffau H. Anatomic dissection of the inferior fronto-occipital fasciculus revisited in the lights of brain stimulation data. *Cortex*. 2010/05/01/ 2010;46(5):691-699. doi:https://doi.org/10.1016/j.cortex.2009.07.015
23. Wu Y, Sun D, Wang Y, Wang Y. Subcomponents and Connectivity of the Inferior Fronto-Occipital Fasciculus Revealed by Diffusion Spectrum Imaging Fiber Tracking. 10.3389/fnana.2016.00088. *Frontiers in Neuroanatomy*. 2016;10:88.
24. Rollans C, Cummine J. One tract, two tract, old tract, new tract: A pilot study of the structural and functional differentiation of the inferior fronto-occipital fasciculus. *Journal of Neurolinguistics*. 2018/05/01/ 2018;46:122-137. doi:https://doi.org/10.1016/j.jneuroling.2017.12.009
25. Sarubbo S, De Benedictis A, Maldonado IL, Basso G, Duffau H. Frontal terminations for the inferior fronto-occipital fascicle: anatomical dissection, DTI study and functional considerations on a multi-component bundle. *Brain Struct Funct*. Jan 2013;218(1):21-37. doi:10.1007/s00429-011-0372-3
26. Corbetta M, Patel G, Shulman GL. The Reorienting System of the Human Brain: From Environment to Theory of Mind. *Neuron*. 2008/05/08/ 2008;58(3):306-324. doi:https://doi.org/10.1016/j.neuron.2008.04.017
27. Clemens B, Regenbogen C, Koch K*, et al*. Incidental Memory Encoding Assessed with Signal Detection Theory and Functional Magnetic Resonance Imaging (fMRI). 10.3389/fnbeh.2015.00305. *Frontiers in Behavioral Neuroscience*. 2015;9:305.
28. Uncapher MR, Rugg MD. Selecting for Memory? The Influence of Selective Attention on the Mnemonic Binding of Contextual Information. *The Journal of Neuroscience*. 2009;29(25):8270. doi:10.1523/JNEUROSCI.1043-09.2009

29. Festini SB, Katz B. A Frontal Account of False Alarms. *Journal of Cognitive Neuroscience*. 2021;33(9):1657-1678. doi:10.1162/jocn_a_01683
30. Simons JS, Spiers HJ. Prefrontal and medial temporal lobe interactions in long-term memory. *Nature Reviews Neuroscience*. 2003/08/01 2003;4(8):637-648. doi:10.1038/nrn1178
31. Rapcsak SZ, Edmonds EC. The Executive Control of Face Memory. *Behavioural Neurology*. 1900/01/01 2011;24:692460. doi:10.3233/BEN-2011-0339
32. Gratwicke J, Jahanshahi M, Foltynie T. Parkinson's disease dementia: a neural networks perspective. *Brain*. 2015;138(6):1454-1476. doi:10.1093/brain/awv104
33. Kantner J, Lindsay DS. Response bias in recognition memory as a cognitive trait. *Memory & Cognition*. 2012/11/01 2012;40(8):1163-1177. doi:10.3758/s13421-012-0226-0
34. Windmann S, Urbach TP, Kutas M. Cognitive and Neural Mechanisms of Decision Biases in Recognition Memory. *Cerebral Cortex*. 2002;12(8):808-817. doi:10.1093/cercor/12.8.808
35. Hill H, Windmann S. Examining Event-Related Potential (ERP) correlates of decision bias in recognition memory judgments. *PLoS One*. 2014;9(9):e106411. doi:10.1371/journal.pone.0106411
36. Blackwood N, ffytche D, Simmons A, Bentall R, Murray R, Howard R. The cerebellum and decision making under uncertainty. *Cognitive Brain Research*. 2004/06/01/ 2004;20(1):46-53. doi:https://doi.org/10.1016/j.cogbrainres.2003.12.009
37. Kim SG, Uğurbil K, Strick PL. Activation of a Cerebellar Output Nucleus During Cognitive Processing. *Science*. 1994/08/12 1994;265(5174):949-951. doi:10.1126/science.8052851
38. Brandt J. The hopkins verbal learning test: Development of a new memory test with six equivalent forms. *Clinical Neuropsychologist*. 1991/04/01 1991;5(2):125-142. doi:10.1080/13854049108403297
39. Lacritz LH, Cullum CM. The Hopkins Verbal Learning Test and CVLT: A preliminary comparison. *Archives of Clinical Neuropsychology*. 1998;13:623–628.
40. Marvel CL, Desmond JE. Functional Topography of the Cerebellum in Verbal Working Memory. *Neuropsychology Review*. 2010/09/01 2010;20(3):271-279. doi:10.1007/s11065-010-9137-7
41. Akshoomoff NA, Courchesne E, Press GA, Iragui V. Contribution of the cerebellum to neuropsychological functioning: Evidence from a case of cerebellar degenerative disorder. *Neuropsychologia*. 1992/04/01/ 1992;30(4):315-328. doi:https://doi.org/10.1016/0028-3932(92)90105-U
42. Murdoch BE. The cerebellum and language: Historical perspective and review. *Cortex*. 2010/07/01/ 2010;46(7):858-868. doi:https://doi.org/10.1016/j.cortex.2009.07.018
43. Mariën P, Ackermann H, Adamaszek M*, et al*. Consensus Paper: Language and the Cerebellum: an Ongoing Enigma. *The Cerebellum*. 2014/06/01 2014;13(3):386-410. doi:10.1007/s12311-013-0540-5
44. Paulesu E, Frith CD, Frackowiak RSJ. The neural correlates of the verbal component of working memory. *Nature*. 1993/03/01 1993;362(6418):342-345. doi:10.1038/362342a0
45. Binder JR. The Wernicke area. *Neurology*. 2015;85(24):2170. doi:10.1212/WNL.0000000000002219
46. Herman AB, Houde JF, Vinogradov S, Nagarajan SS. Parsing the Phonological Loop: Activation Timing in the Dorsal Speech Stream Determines Accuracy in Speech Reproduction. *The Journal of Neuroscience*. 2013;33(13):5439. doi:10.1523/JNEUROSCI.1472-12.2013
47. López-Barroso D, Catani M, Ripollés P*, et al*. Word learning is mediated by the left arcuate fasciculus. *Proceedings of the National Academy of Sciences*. 2013;110(32):13168. doi:10.1073/pnas.1301696110
48. Ravizza SM, Hazeltine E, Ruiz S, Zhu DC. Left TPJ activity in verbal working memory: Implications for storage- and sensory-specific models of short term memory.

NeuroImage. 2011/04/15/ 2011;55(4):1836-1846. doi:https://doi.org/10.1016/j.neuroimage.2010.12.021
49. Wise RJS, Scott SK, Blank SC, Mummery CJ, Murphy K, Warburton EA. Separate neural subsystems within `Wernicke's area'. *Brain*. 2001;124(1):83-95. doi:10.1093/brain/124.1.83
50. Wu AS, Witgert ME, Lang FF*, et al*. Neurocognitive function before and after surgery for insular gliomas. *J Neurosurg*. Dec 2011;115(6):1115-25. doi:10.3171/2011.8.JNS11488
51. Mechelli A, Humphreys GW, Mayall K, Olson A, Price CJ. Differential effects of word length and visual contrast in the fusiform and lingual gyri during reading. *Proc Biol Sci*. Sep 22 2000;267(1455):1909-13. doi:10.1098/rspb.2000.1229
52. Leshikar ED, Duarte A, Hertzog C. Task-selective memory effects for successfully implemented encoding strategies. *PLoS One*. 2012;7(5):e38160. doi:10.1371/journal.pone.0038160
53. Catani M, Robertsson N, Beyh A*, et al*. Short parietal lobe connections of the human and monkey brain. *Cortex*. 2017/12/01/ 2017;97:339-357. doi:https://doi.org/10.1016/j.cortex.2017.10.022
54. Sestieri C, Shulman GL, Corbetta M. The contribution of the human posterior parietal cortex to episodic memory. *Nature Reviews Neuroscience*. 2017/03/01 2017;18(3):183-192. doi:10.1038/nrn.2017.6
55. Coslett HB, Schwartz MF. Chapter 18 - The parietal lobe and language. In: Vallar G, Coslett HB, eds. *Handbook of Clinical Neurology*. Elsevier; 2018:365-375.
56. Husain M, Nachev P. Space and the parietal cortex. *Trends in Cognitive Sciences*. 2007/01/01/ 2007;11(1):30-36. doi:https://doi.org/10.1016/j.tics.2006.10.011
57. Singh-Curry V, Husain M. The functional role of the inferior parietal lobe in the dorsal and ventral stream dichotomy. *Neuropsychologia*. 2009/05/01/ 2009;47(6):1434-1448. doi:https://doi.org/10.1016/j.neuropsychologia.2008.11.033
58. Seghier ML. The Angular Gyrus: Multiple Functions and Multiple Subdivisions. *The Neuroscientist*. 2013/02/01 2012;19(1):43-61. doi:10.1177/1073858412440596
59. Binder JR, Desai RH, Graves WW, Conant LL. Where Is the Semantic System? A Critical Review and Meta-Analysis of 120 Functional Neuroimaging Studies. *Cerebral Cortex*. 2009;19(12):2767-2796. doi:10.1093/cercor/bhp055
60. Tracey I, Mantyh PW. The cerebral signature for pain perception and its modulation. *Neuron*. Aug 2 2007;55(3):377-91. doi:10.1016/j.neuron.2007.07.012
61. Basbaum AI, Bautista DM, Scherrer G, Julius D. Cellular and molecular mechanisms of pain. *Cell*. Oct 16 2009;139(2):267-84. doi:10.1016/j.cell.2009.09.028
62. de Haan R, Aaronson N, Limburg M, Hewer RL, van Crevel H. Measuring quality of life in stroke. *Stroke*. Feb 1993;24(2):320-7. doi:10.1161/01.str.24.2.320
63. van Straten A, de Haan RJ, Limburg M, Schuling J, Bossuyt PM, van den Bos GA. A stroke-adapted 30-item version of the Sickness Impact Profile to assess quality of life (SA-SIP30). *Stroke*. Nov 1997;28(11):2155-61. doi:10.1161/01.str.28.11.2155
64. Van de Port IG, Ketelaar M, Schepers VP, Van den Bos GA, Lindeman E. Monitoring the functional health status of stroke patients: the value of the Stroke-Adapted Sickness Impact Profile-30. *Disabil Rehabil*. Jun 3 2004;26(11):635-40. doi:10.1080/09638280410001672481
65. Harte SE, Ichesco E, Hampson JP*, et al*. Pharmacologic attenuation of cross-modal sensory augmentation within the chronic pain insula. *Pain*. Sep 2016;157(9):1933-1945. doi:10.1097/j.pain.0000000000000593
66. Von Der Heide RJ, Skipper LM, Klobusicky E, Olson IR. Dissecting the uncinate fasciculus: disorders, controversies and a hypothesis. *Brain*. Jun 2013;136(Pt 6):1692-707. doi:10.1093/brain/awt094
67. Shigihara Y, Tanaka M, Ishii A, Kanai E, Funakura M, Watanabe Y. Two types of mental fatigue affect spontaneous oscillatory brain activities in different ways. *Behav Brain Funct*. Jan 10 2013;9:2. doi:10.1186/1744-9081-9-2

68. van Straten A, de Haan RJ, Limburg M, van den Bos GA. Clinical meaning of the Stroke-Adapted Sickness Impact Profile-30 and the Sickness Impact Profile-136. *Stroke*. Nov 2000;31(11):2610-5. doi:10.1161/01.str.31.11.2610
