# Supplementary material for: Latent disconnectome prediction of long-term cognitive-behavioural symptoms in stroke
Source: Brain. 2023 Mar 16;146(5):1963–78. doi: 10.1093/brain/awad013 (PMC10151183; doi:10.1093/brain/awad013)
Supplement: awad013_Supplementary_Data [file awad013_Supplementary_Data.zip › brain-2022-00965-File013.pdf]

Supplementary Materials

TABLE OF CONTENTS

D. The Neuropsychological White Matter Atlas - NWMA

Supplementary Figures 50-57: High-resolution summary maps. p.2

D. The Neuropsychological White Matter Atlas - NWMA

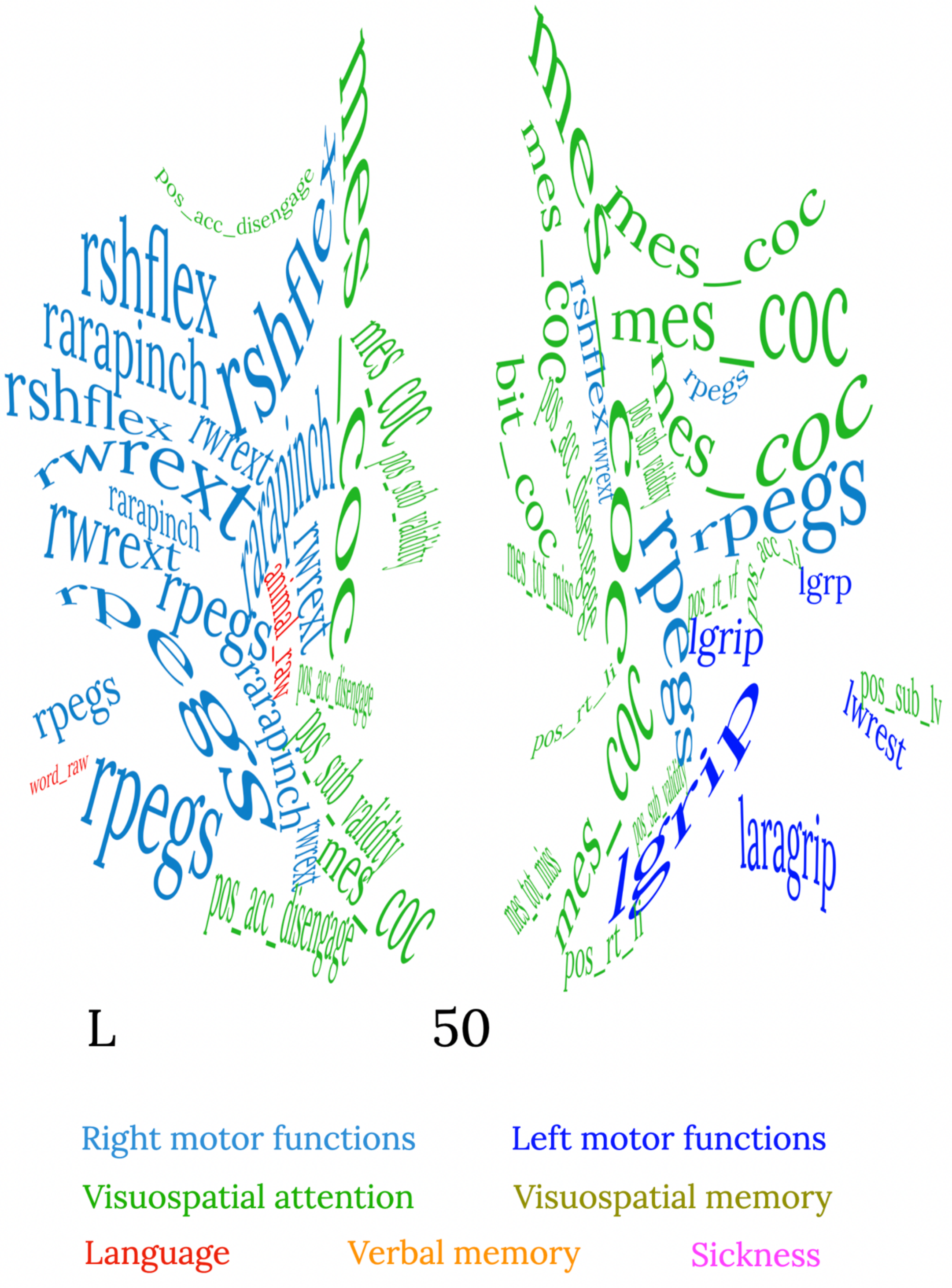

Supplementary Figure 50: z=50 mm (Neurological convention, MNI coordinates). Full names for the abbreviations used for the scores are available in Supplementary Table 1 and at http://disconnectomestudio.bcblab.com. The text font size and curvature reported were manually set to suit the size of the significant clusters and to follow the orientation of the subjacent white matter orientations.

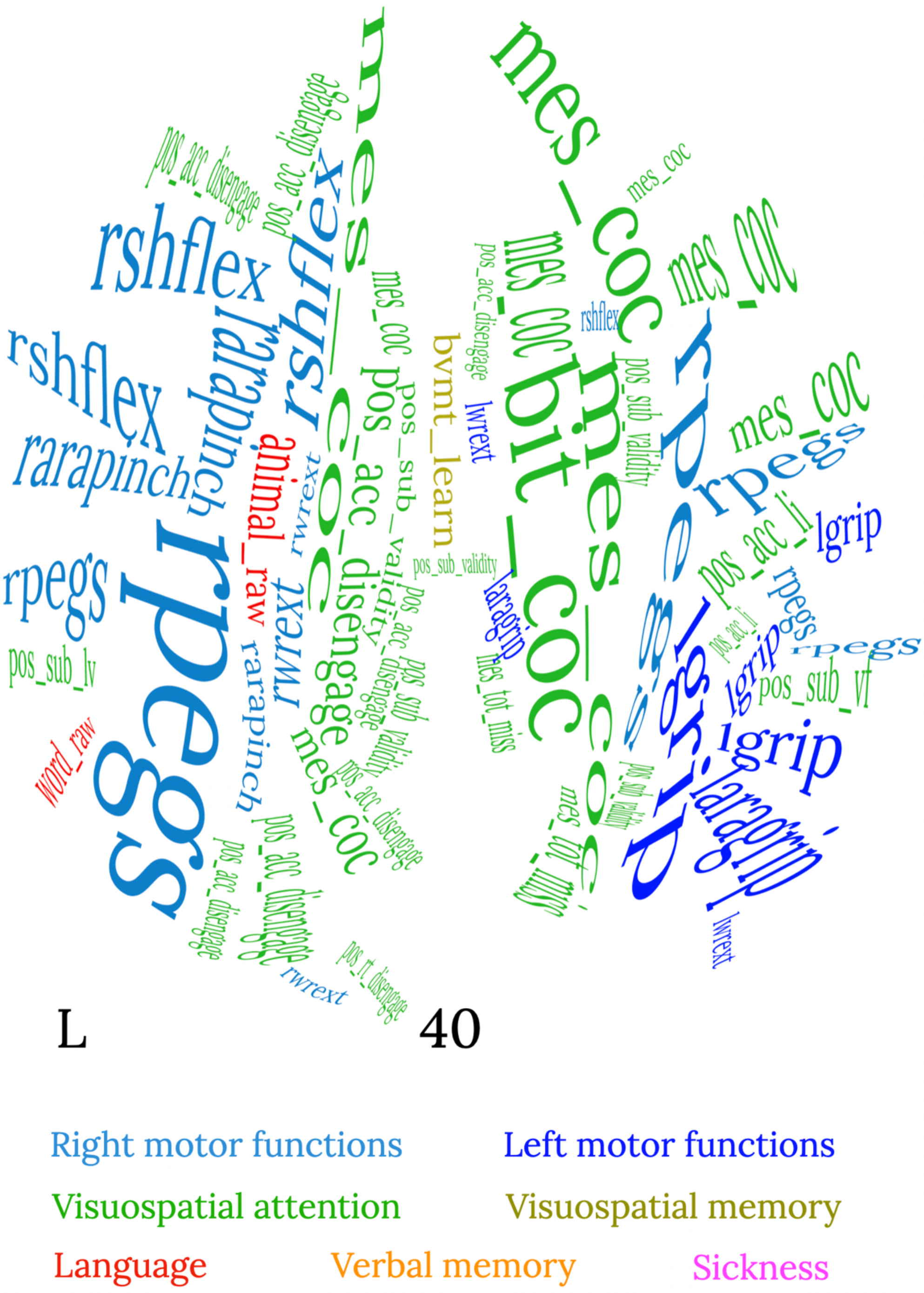

Supplementary Figure 51: z=40 mm (Neurological convention, MNI coordinates). Full names for the abbreviations used for the scores are available in Supplementary Table 1 and at http://disconnectomestudio.bcblab.com. The text font size and curvature reported were manually set to suit the size of the significant clusters and to follow the orientation of the subjacent white matter orientations.

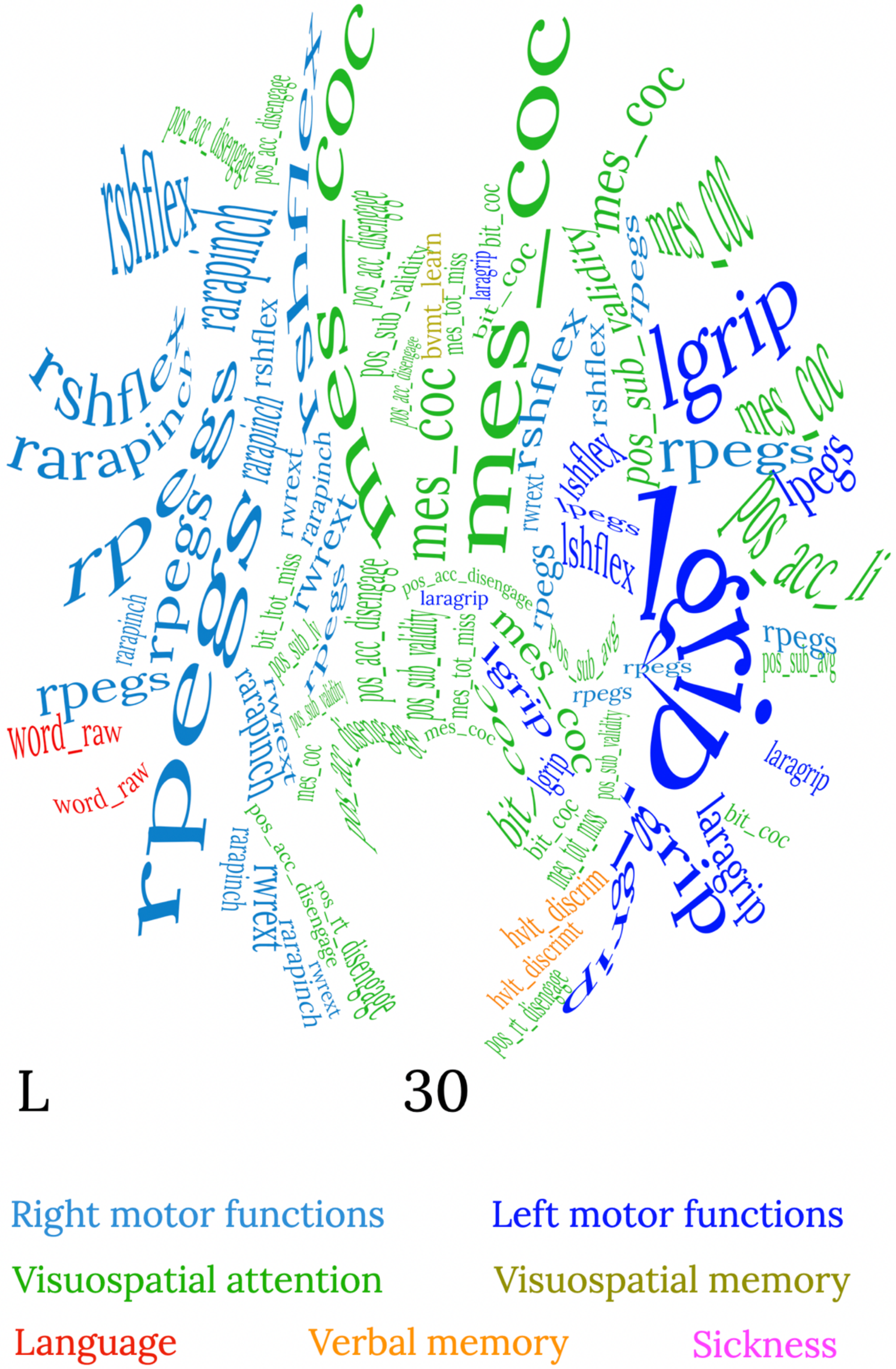

Supplementary Figure 52: z=30 mm (Neurological convention, MNI coordinates). Full names for the abbreviations used for the scores are available in Supplementary Table 1 and at http://disconnectomestudio.bcblab.com. The text font size and curvature reported were manually set to suit the size of the significant clusters and to follow the orientation of the subjacent white matter orientations.

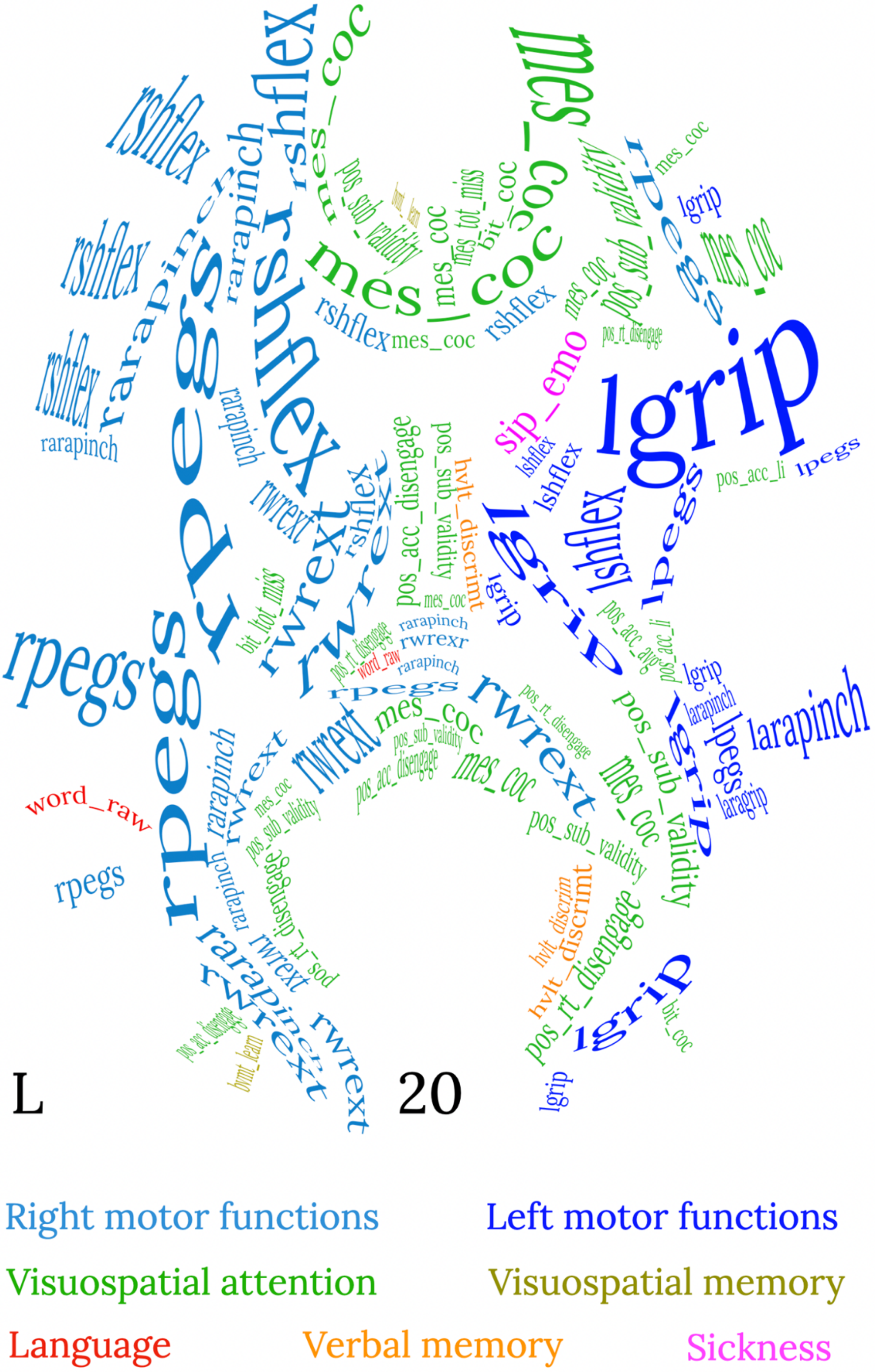

Supplementary Figure 53: z=20 mm (Neurological convention, MNI coordinates). Full names for the abbreviations used for the scores are available in Supplementary Table 1 and at http://disconnectomestudio.bcblab.com. The text font size and curvature reported were manually set to suit the size of the significant clusters and to follow the orientation of the subjacent white matter orientations.

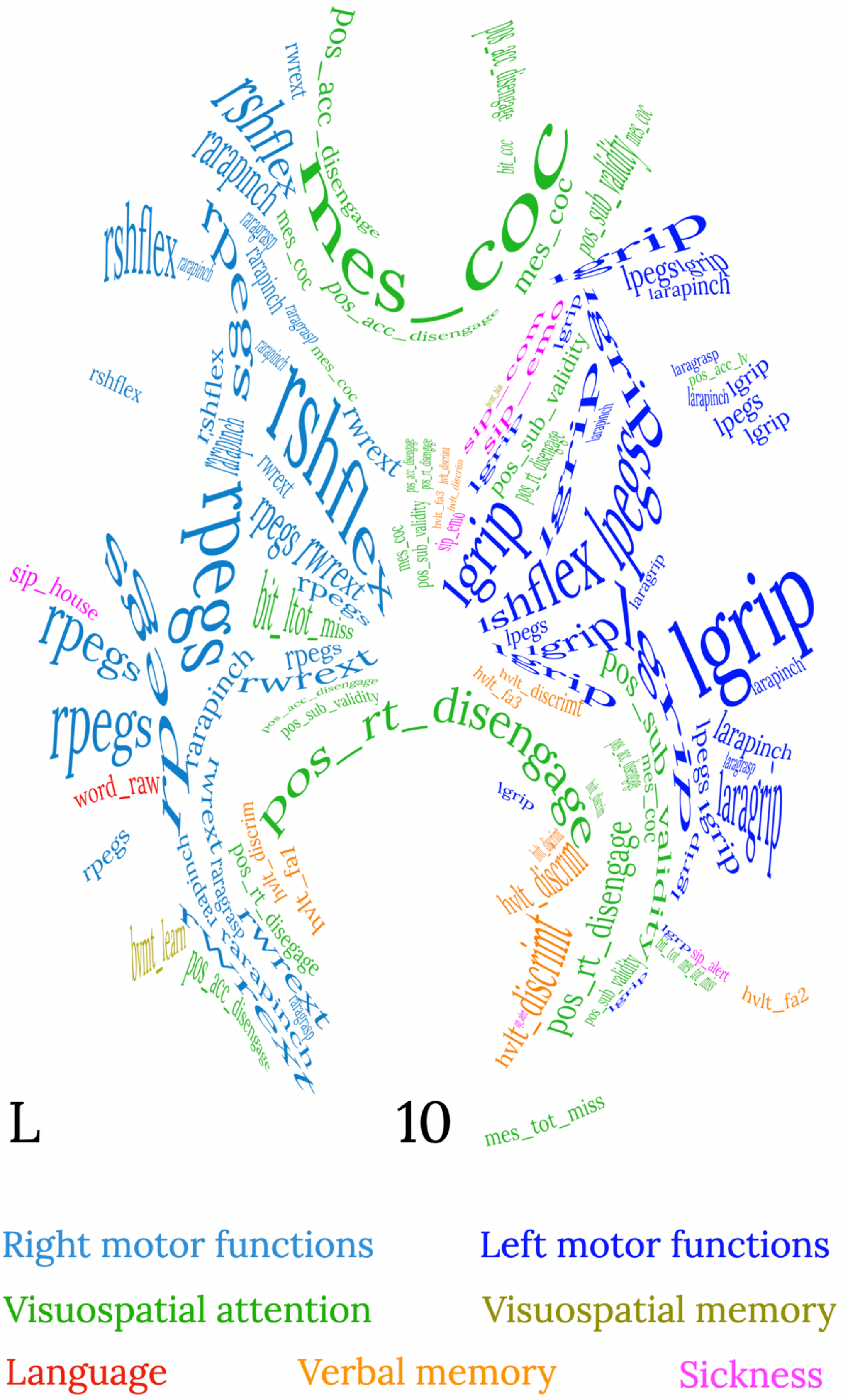

Supplementary Figure 54: z=10 mm (Neurological convention, MNI coordinates). Full names for the abbreviations used for the scores are available in Supplementary Table 1 and at http://disconnectomestudio.bcblab.com. The text font size and curvature reported were manually set to suit the size of the significant clusters and to follow the orientation of the subjacent white matter orientations.

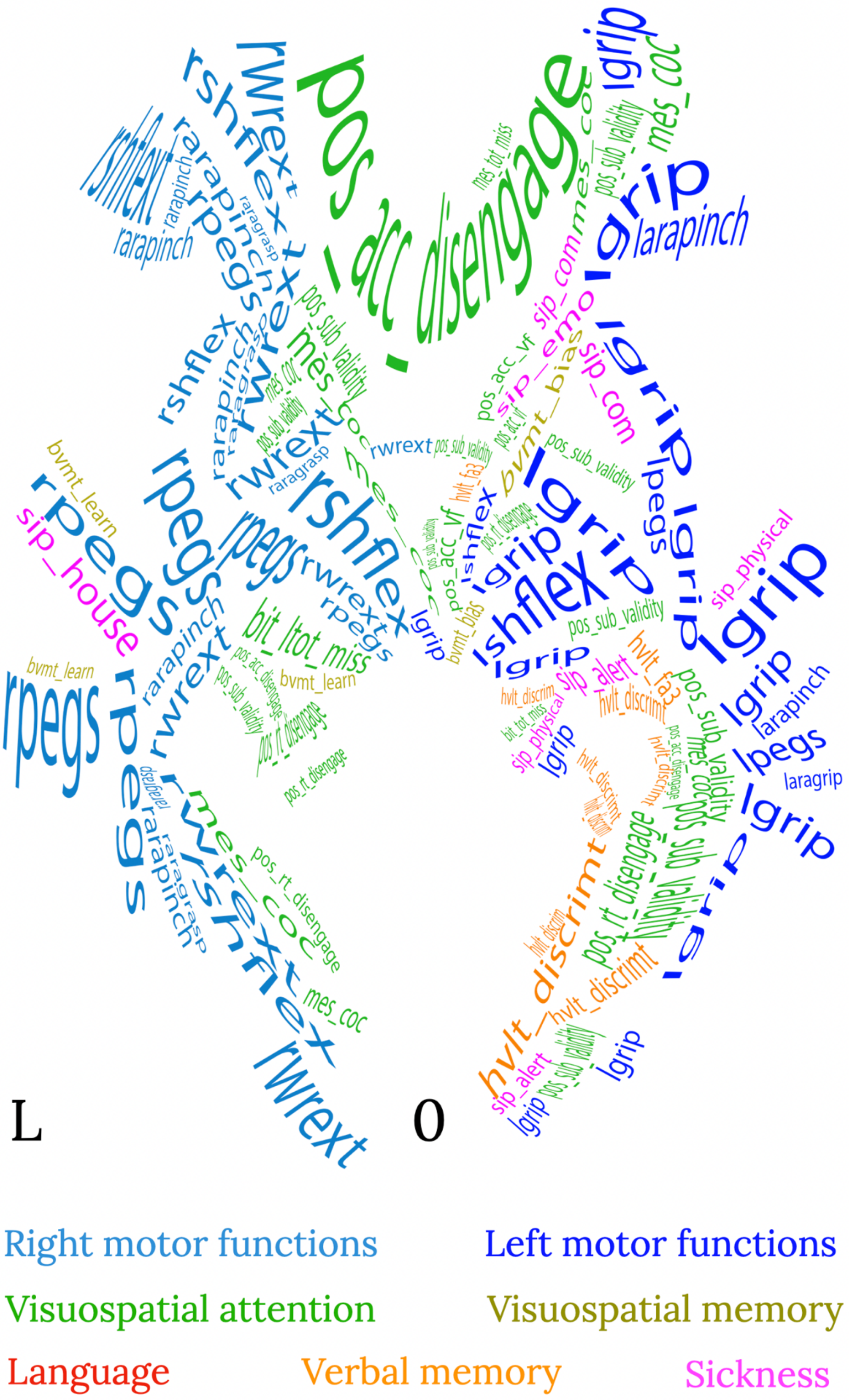

Supplementary Figure 55: z=0 mm (Neurological convention, MNI coordinates). Full names for the abbreviations used for the scores are available in Supplementary Table 1 and at http://disconnectomestudio.bcblab.com. The text font size and curvature reported were manually set to suit the size of the significant clusters and to follow the orientation of the subjacent white matter orientations.

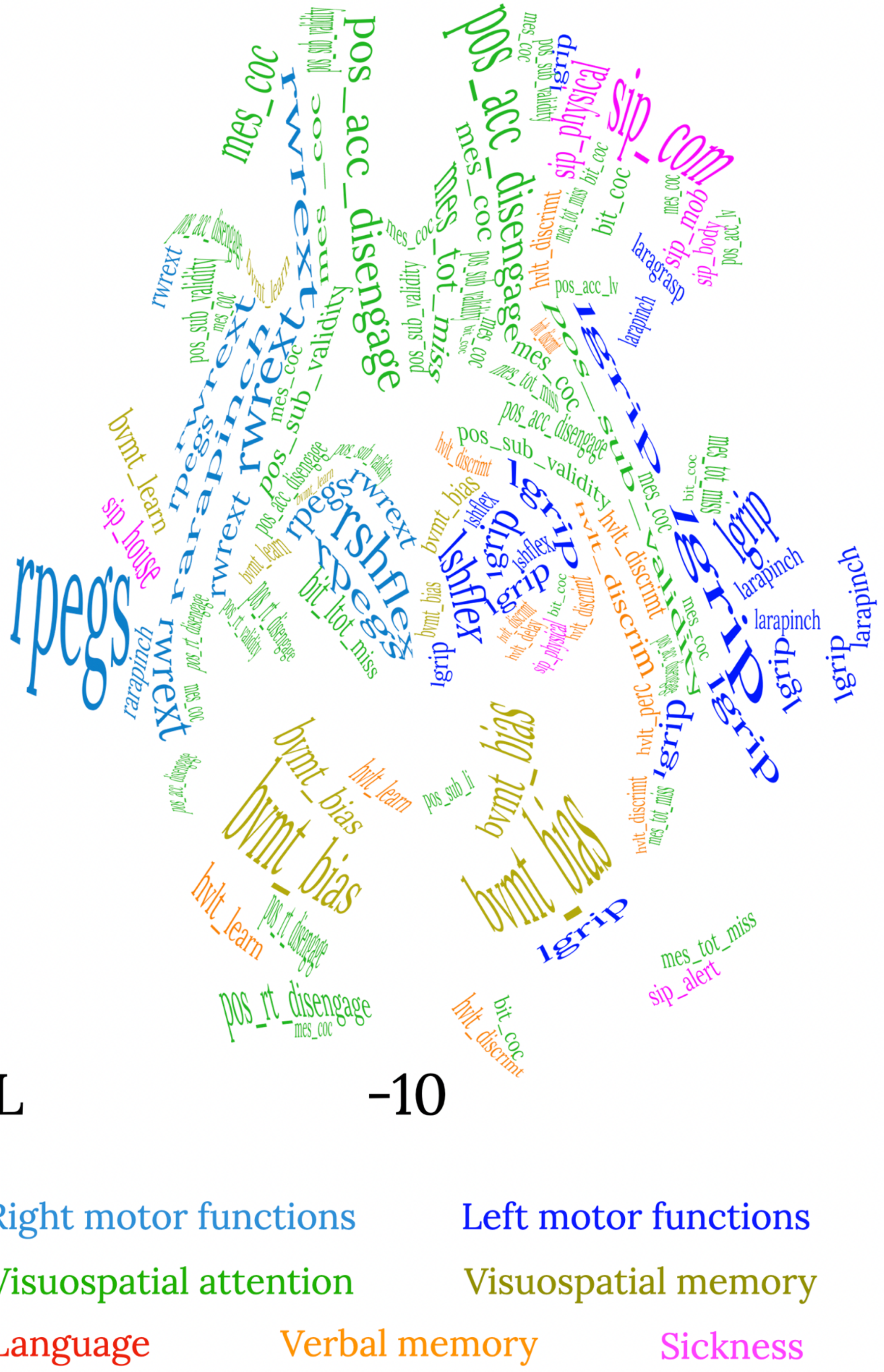

Supplementary Figure 56: z=-10 mm (Neurological convention, MNI coordinates). Full names for the abbreviations used for the scores are available in Supplementary Table 1 and at http://disconnectomestudio.bcblab.com. The text font size and curvature reported were manually set to suit the size of the significant clusters and to follow the orientation of the subjacent white matter orientations.

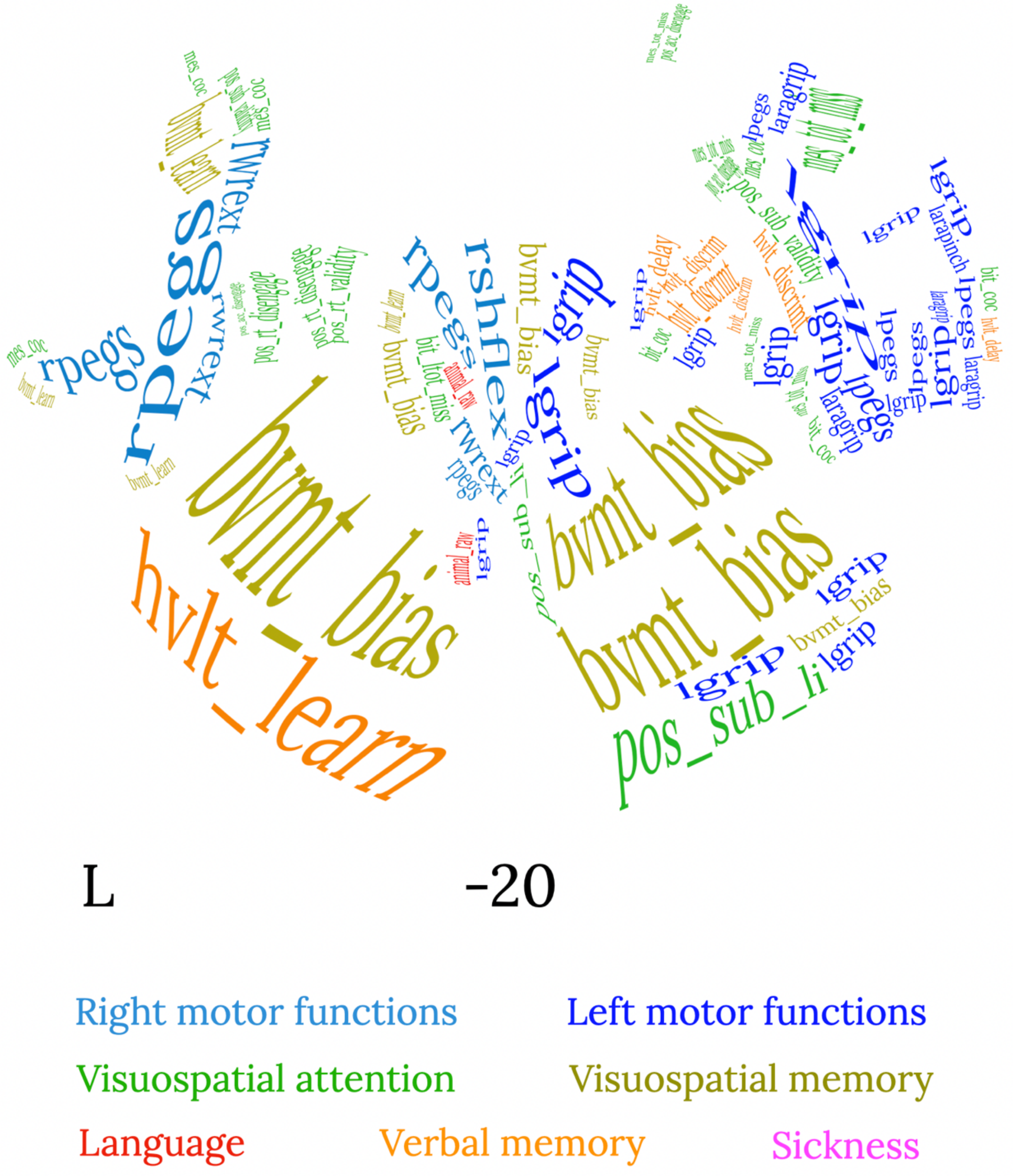

Supplementary Figure 57: z=-20 mm (Neurological convention, MNI coordinates). Full names for the abbreviations used for the scores are available in Supplementary Table 1 and at http://disconnectomestudio.bcblab.com. The text font size and curvature reported were manually set to suit the size of the significant clusters and to follow the orientation of the subjacent white matter orientations.
