# Supplementary material for: Latent disconnectome prediction of long-term cognitive-behavioural symptoms in stroke
Source: Brain. 2023 Mar 16;146(5):1963–78. doi: 10.1093/brain/awad013 (PMC10151183; doi:10.1093/brain/awad013)
Supplement: awad013_Supplementary_Data [file awad013_Supplementary_Data.zip › brain-2022-00965-File014.pdf]

Supplementary Materials

TABLE OF CONTENTS

E. Disconnectome studio web application user guide p.2

F. Individual neuropsychological profiles prediction p.2

Supplementary Figures 58-77: radar plots of the measured scores and DSD predictions.

E. Disconnectome studio web application user guide

Before running the online DSD web application you need to:

1. segment your stroke patient's lesion. For this purpose, several software can be used, e.g. MRIcron (https://www.nitrc.org/projects/mricron), FSLeyes (https://fsl.fmrib.ox.ac.uk/fsl/fslwiki/FSLeyes), mrview (https://mrtrix.readthedocs.io/en/dev/reference/commands/mrview.html), ITKsnap (http://www.itksnap.org/pmwiki/pmwiki.php). Lesions can be segmented using T1-weighted MRI contrast or a lesion multi contrast inspection with T2-weighted and FLAIR MRIs.
2. Download and install the BCBlab toolkit following the instructions at http://bcblab.com/BCB/Softwares.html. Run the "Normalisation" command first, and then the "Disconnectome maps".

Subsequently, go to the web application site http://disconnectomestudio.bcblab.com.
The "info" option provides information about the predicted scores currently available within each neuropsychological domain. The "calculate" option will run the patient predictions. After choosing the "calculate" option, press "Choose File" and select a patient disconnectome file (registered to the MNI152-2mm space and in the nii.gz imaging format) obtained from the BCBlab toolkit computation. Press "Run," and after about one minute, the neuropsychological score table will be filled with the DSD calculated 1-year expected scores and the UMAP patient corresponding coordinates. Press the "Export to CSV," to save a .csv file containing the neuropsychological score on your computer.
No files or tables are saved on the webserver. All the computations run in a temporary directory that is deleted when the output neuropsychological table is displayed online. Accordingly, to re-run the analysis, it will be necessary to upload the patient disconnectome file again.

F. Individual neuropsychological profiles prediction

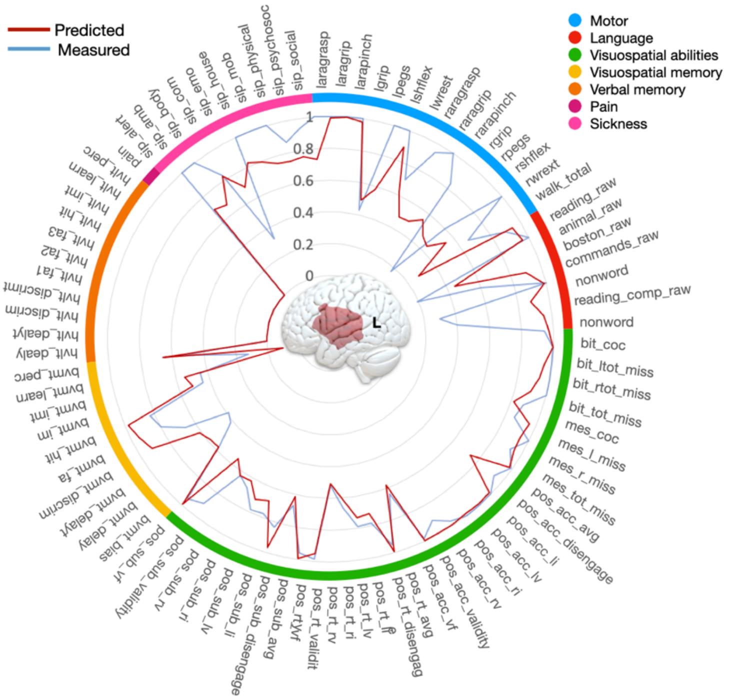

Supplementary Figure 58: Female, 43 years old, left hemisphere stroke. Note that the polarity of some scores was inverted for readability, so that high scores always indicate better performance. The abbreviations of the scores are explained in Supplementary Table 1.

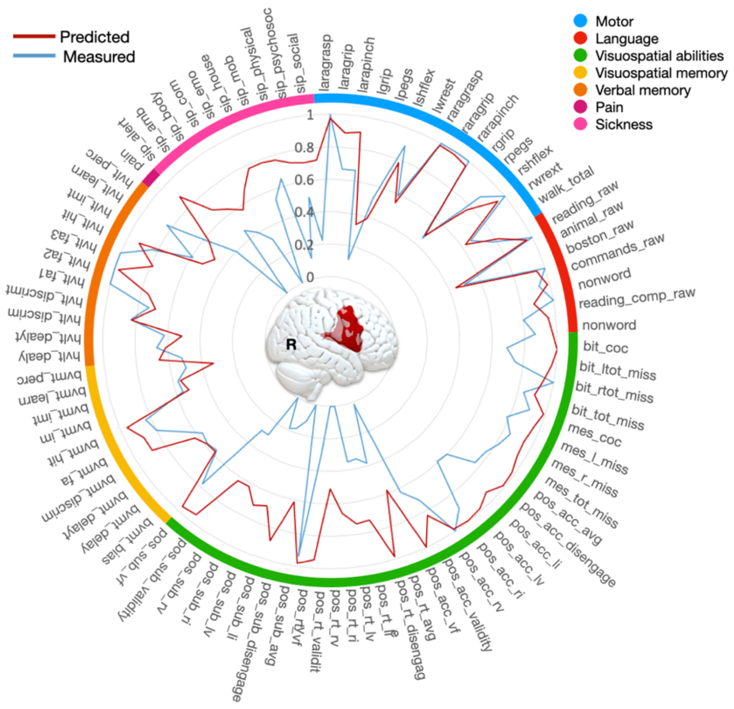

Supplementary Figure 59: Female, 50 years old, right hemisphere stroke. Note that the polarity of some scores was inverted for readability, so that high scores always indicate better performance. The abbreviations of the scores are explained in Supplementary Table 1.

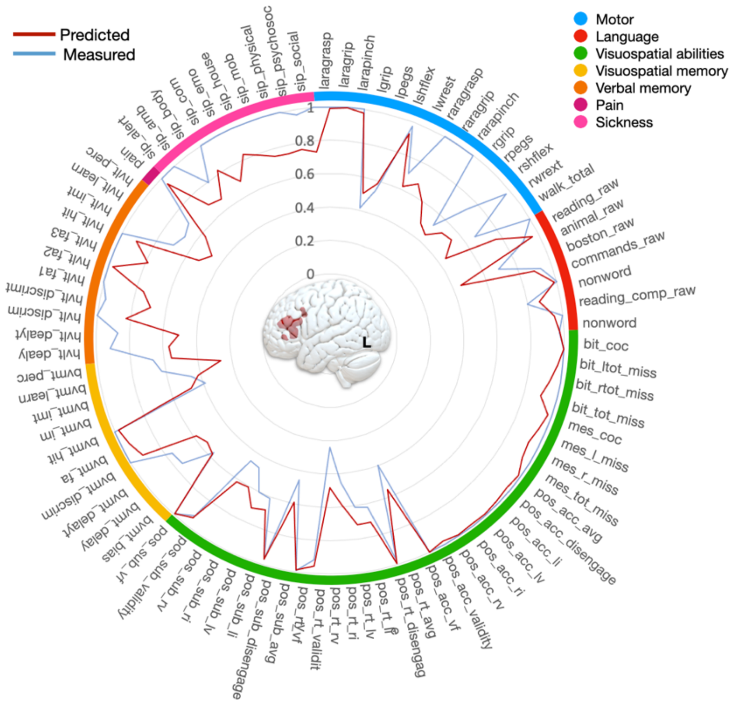

Supplementary Figure 60: Female, 56 years old, left hemisphere stroke. Note that the polarity of some scores was inverted for readability, so that high scores always indicate better performance. The abbreviations of the scores are explained in Supplementary Table 1.

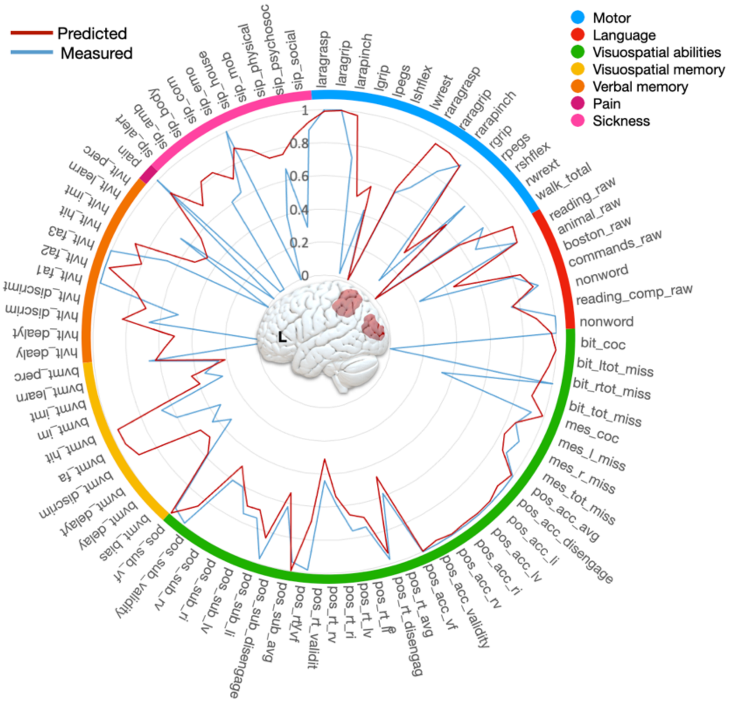

Supplementary Figure 61: Female, 59 years old, left hemisphere stroke. Note that the polarity of some scores was inverted for readability, so that high scores always indicate better performance. The abbreviations of the scores are explained in Supplementary Table 1.

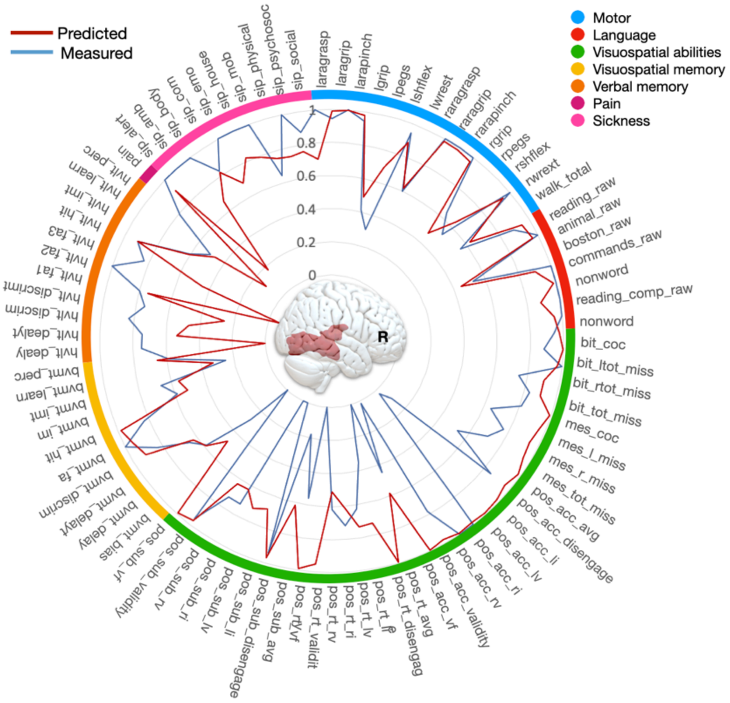

Supplementary Figure 62: Male, 66 years old, right hemisphere stroke. Note that the polarity of some scores was inverted for readability, so that high scores always indicate better performance. The abbreviations of the scores are explained in Supplementary Table 1.

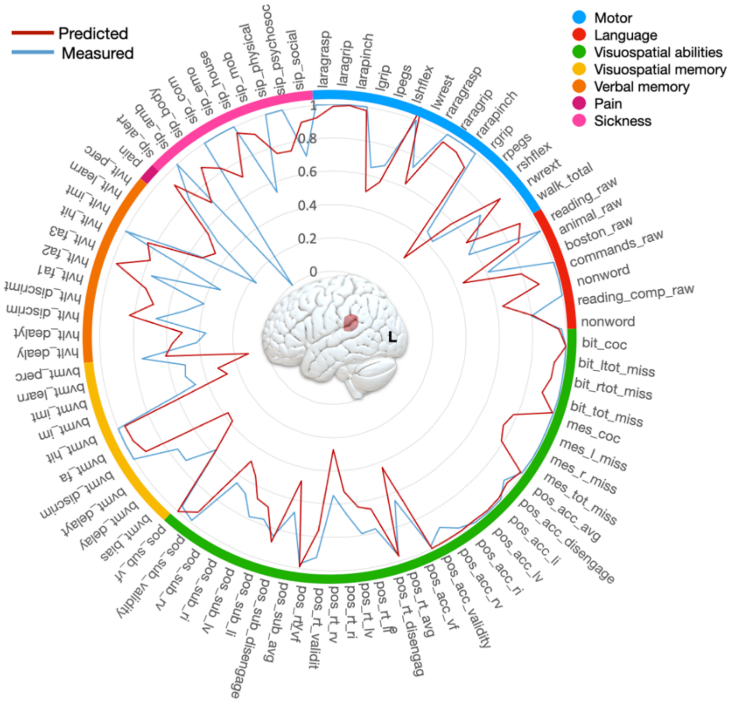

Supplementary Figure 63: Male, 52 years old, left hemisphere stroke. Note that the polarity of some scores was inverted for readability, so that high scores always indicate better performance. The abbreviations of the scores are explained in Supplementary Table 1.

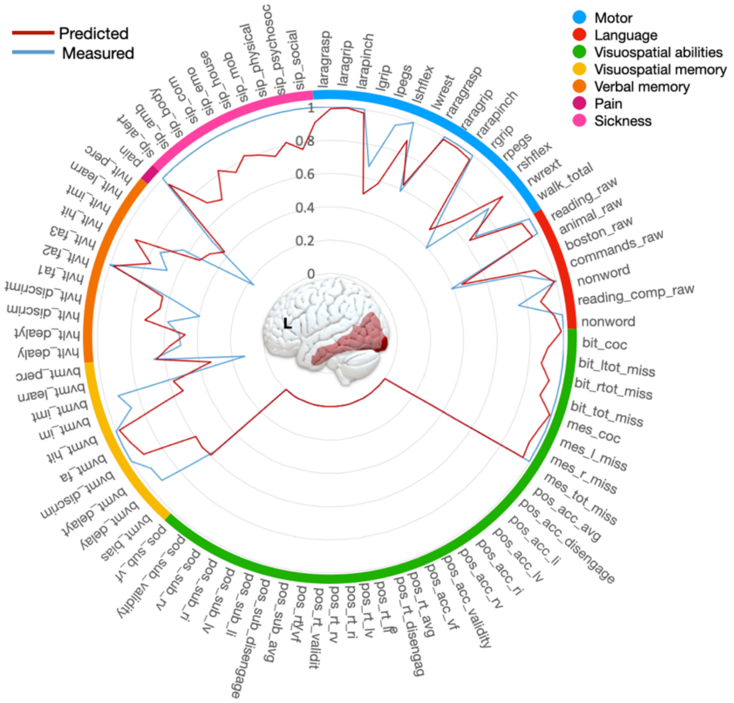

Supplementary Figure 64: Male, 64 years old, left hemisphere stroke. Note that the polarity of some scores was inverted for readability, so that high scores always indicate better performance. The abbreviations of the scores are explained in Supplementary Table 1.

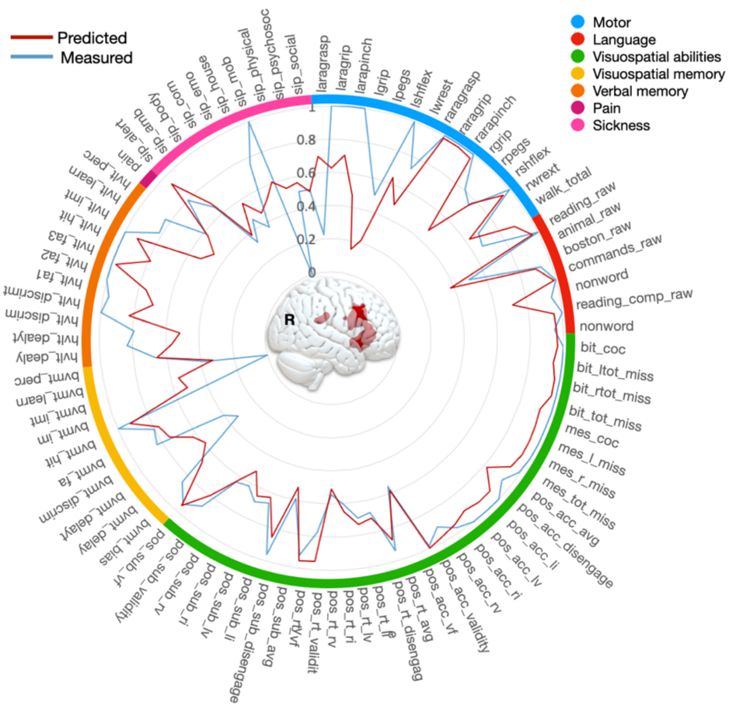

Supplementary Figure 65: Male, 57 years old, right hemisphere stroke. Note that the polarity of some scores was inverted for readability, so that high scores always indicate better performance. The abbreviations of the scores are explained in Supplementary Table 1.

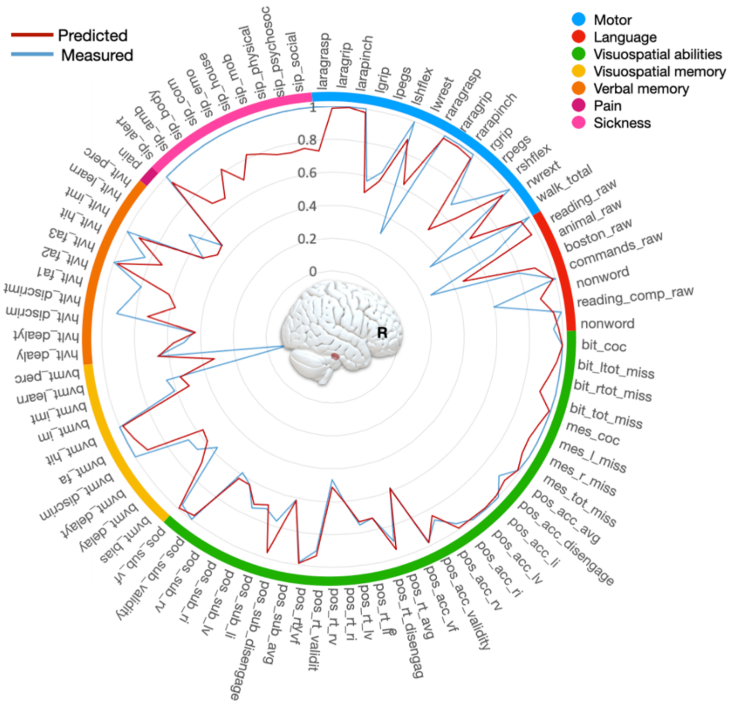

Supplementary Figure 66: Male, 52 years old, right hemisphere stroke. Note that the polarity of some scores was inverted for readability, so that high scores always indicate better performance. The abbreviations of the scores are explained in Supplementary Table 1.

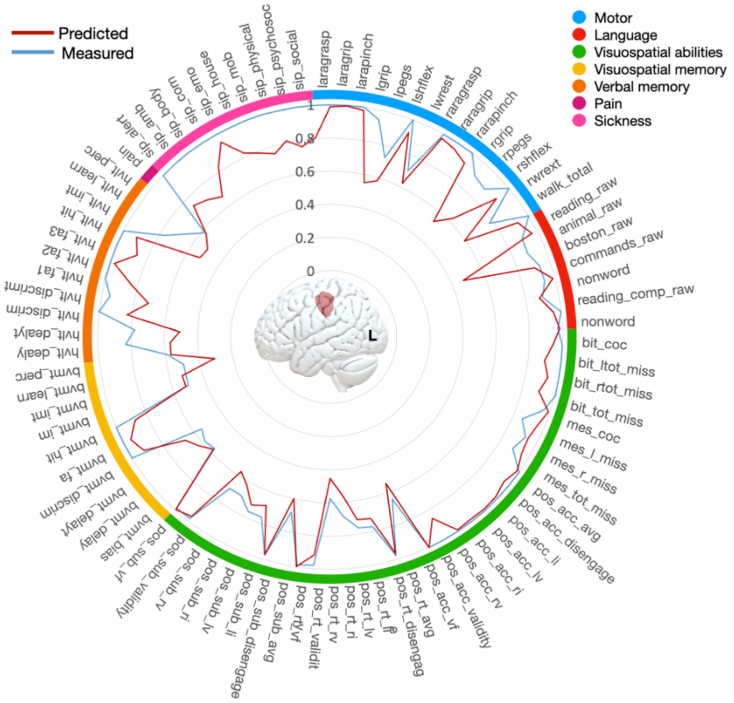

Supplementary Figure 67: Male, 44 years old, left hemisphere stroke. Note that the polarity of some scores was inverted for readability, so that high scores always indicate better performance. The abbreviations of the scores are explained in Supplementary Table 1.

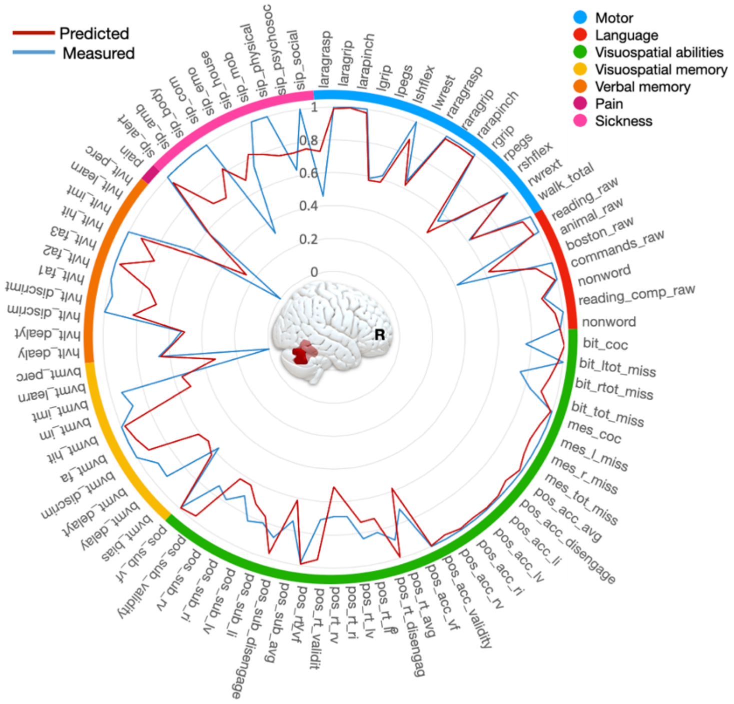

Supplementary Figure 68: Male, 58 years old, right hemisphere stroke. Note that the polarity of some scores was inverted for readability, so that high scores always indicate better performance. The abbreviations of the scores are explained in Supplementary Table 1.

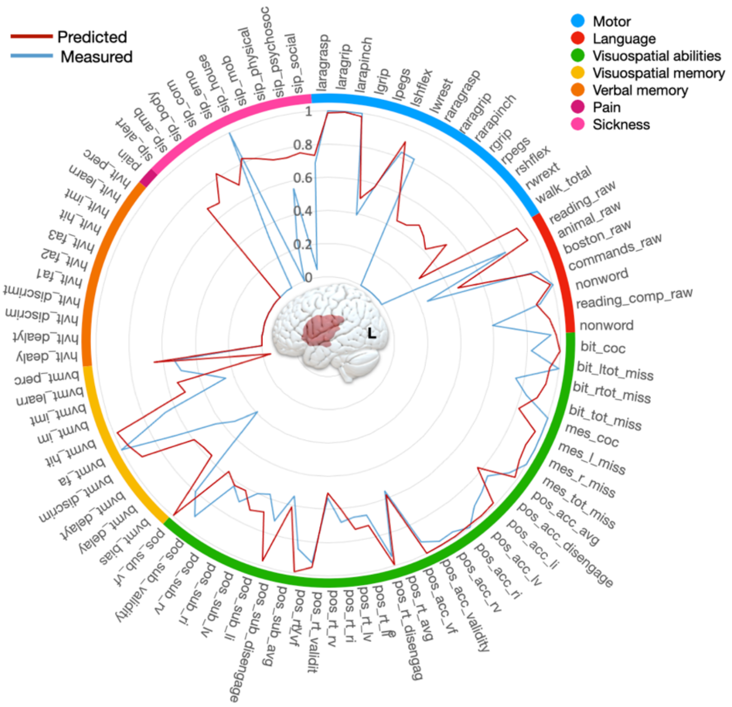

Supplementary Figure 69: Female, 59 years old, left hemisphere stroke. Note that the polarity of some scores was inverted for readability, so that high scores always indicate better performance. The abbreviations of the scores are explained in Supplementary Table 1.

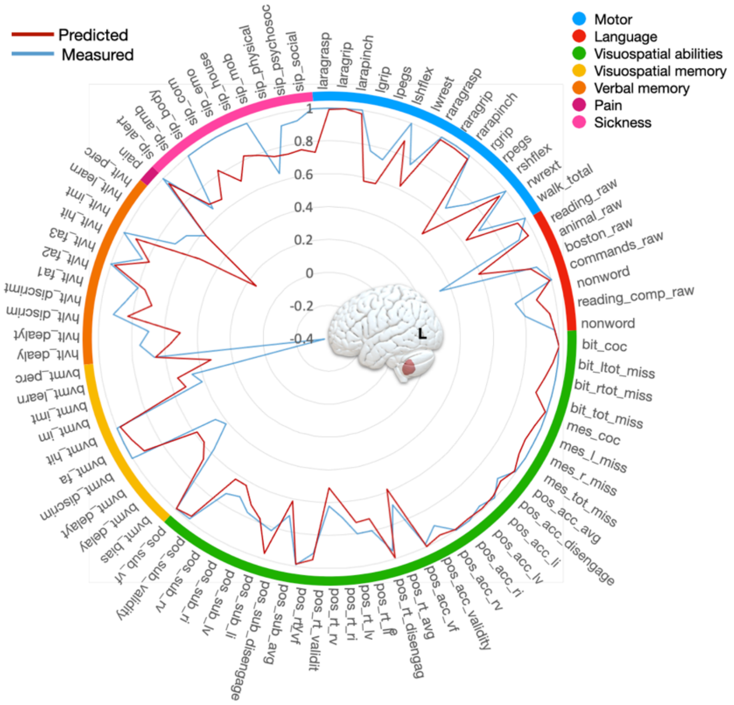

Supplementary Figure 70: Male, 51 years old, left hemisphere stroke. Note that the polarity of some scores was inverted for readability, so that high scores always indicate better performance. The abbreviations of the scores are explained in Supplementary Table 1.

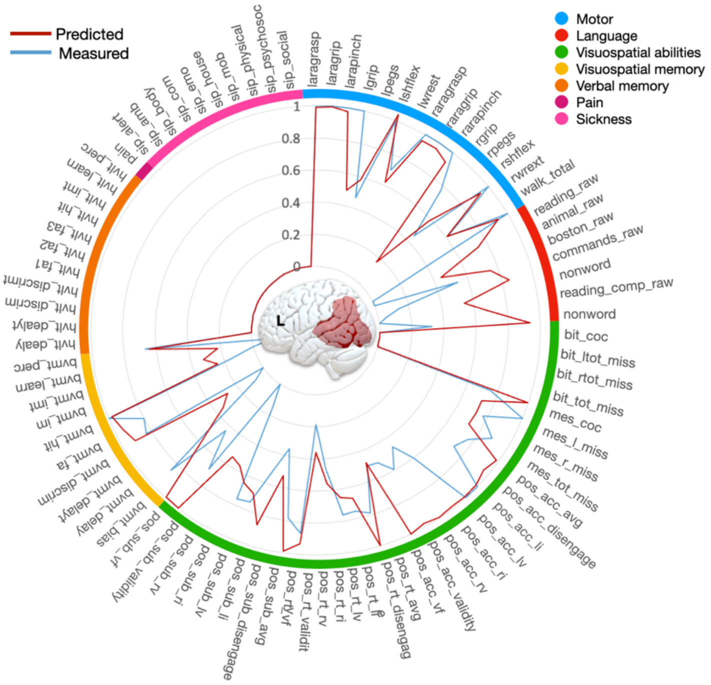

Supplementary Figure 71: Male, 59 years old, left hemisphere stroke. Note that the polarity of some scores was inverted for readability, so that high scores always indicate better performance. The abbreviations of the scores are explained in Supplementary Table 1.

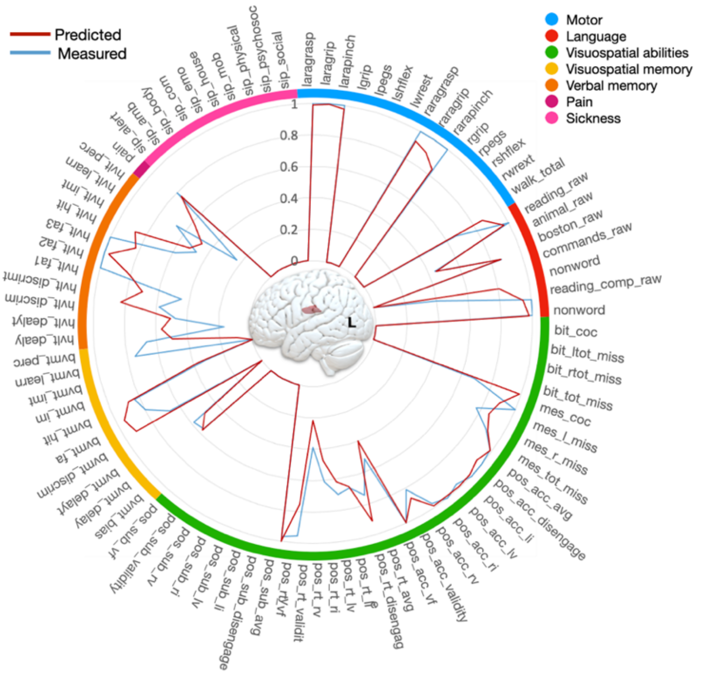

Supplementary Figure 72: Female, 95 years old, left hemisphere stroke. Overall, across the patient's neuropsychological profile, prediction and measured scores matched. Note that the polarity of some scores was inverted for readability, so that high scores always indicate better performance. The abbreviations of the scores are explained in Supplementary Table 1.

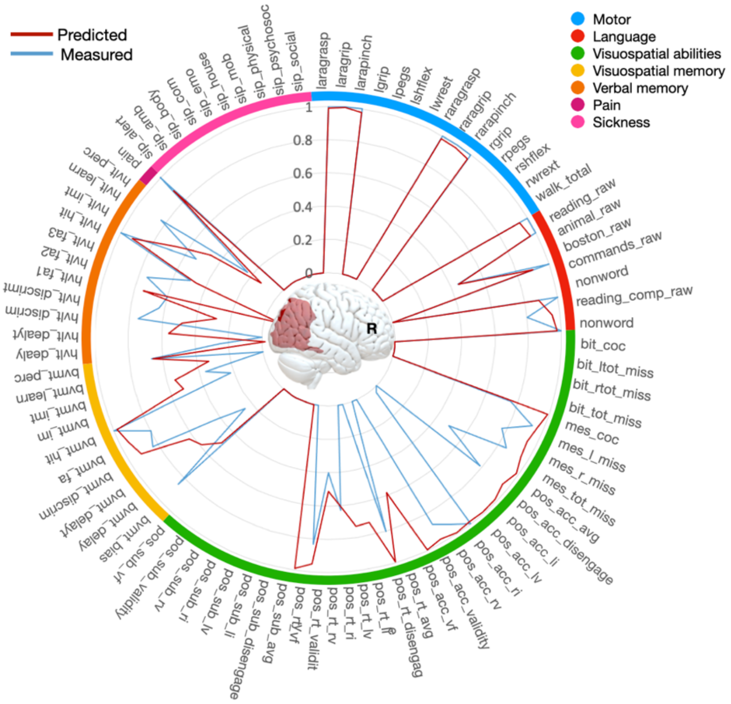

Supplementary Figure 73: Female, 68 years old, right hemisphere stroke. Note that the polarity of some scores was inverted for readability, so that high scores always indicate better performance. The abbreviations of the scores are explained in Supplementary Table 1.

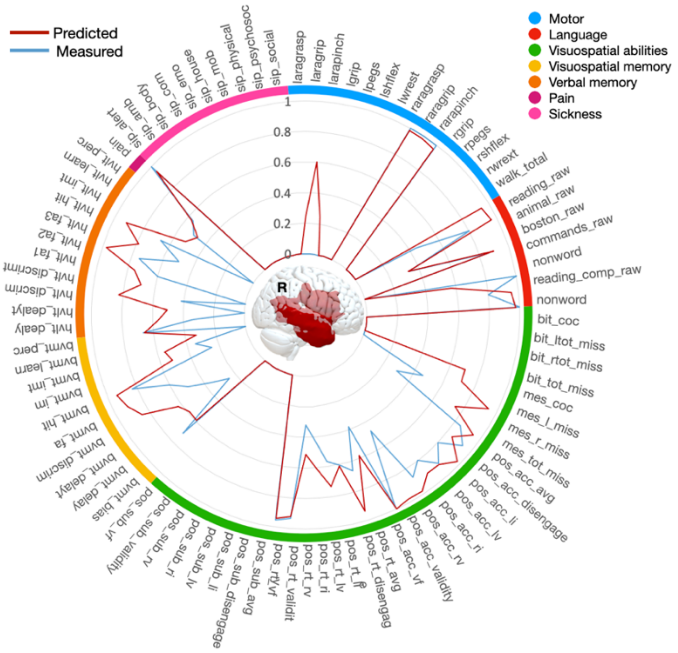

Supplementary Figure 74: Male, 58 years old, right hemisphere stroke. Note that the polarity of some scores was inverted for readability, so that high scores always indicate better performance. The abbreviations of the scores are explained in Supplementary Table 1.

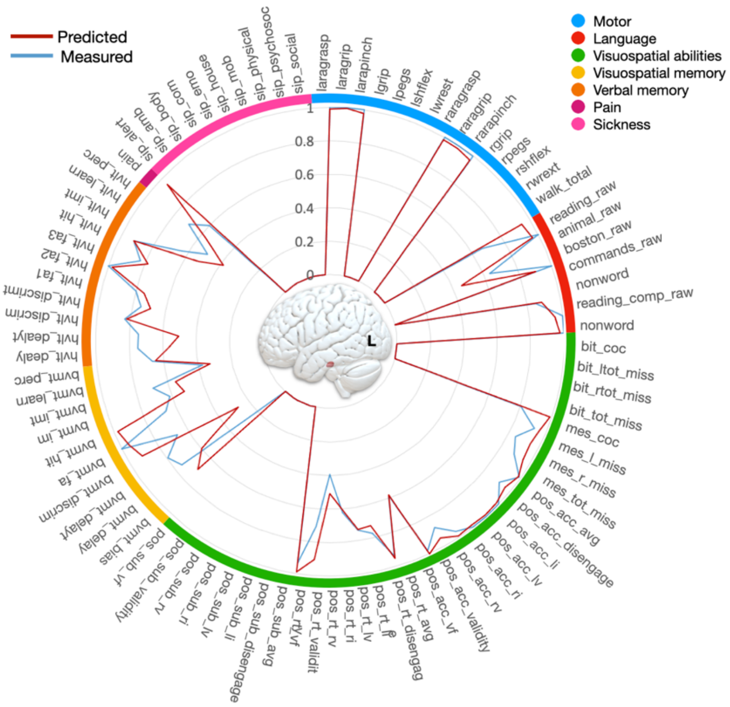

Supplementary Figure 75: Male, 63 years old, left hemisphere stroke. Note that the polarity of some scores was inverted for readability, so that high scores always indicate better performance. The abbreviations of the scores are explained in Supplementary Table 1.

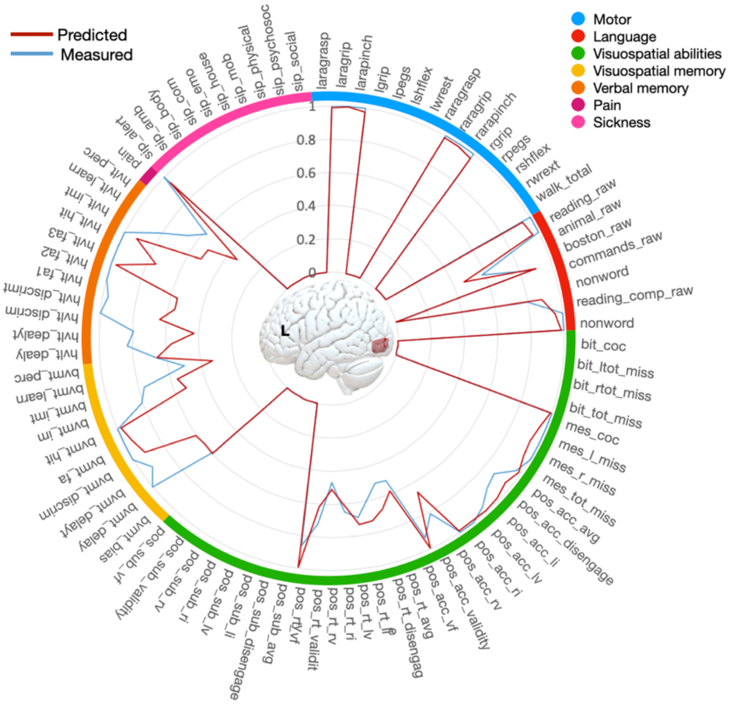

Supplementary Figure 76: Female, 34 years old, left hemisphere stroke. Note that the polarity of some scores was inverted for readability, so that high scores always indicate better performance. The abbreviations of the scores are explained in Supplementary Table 1.

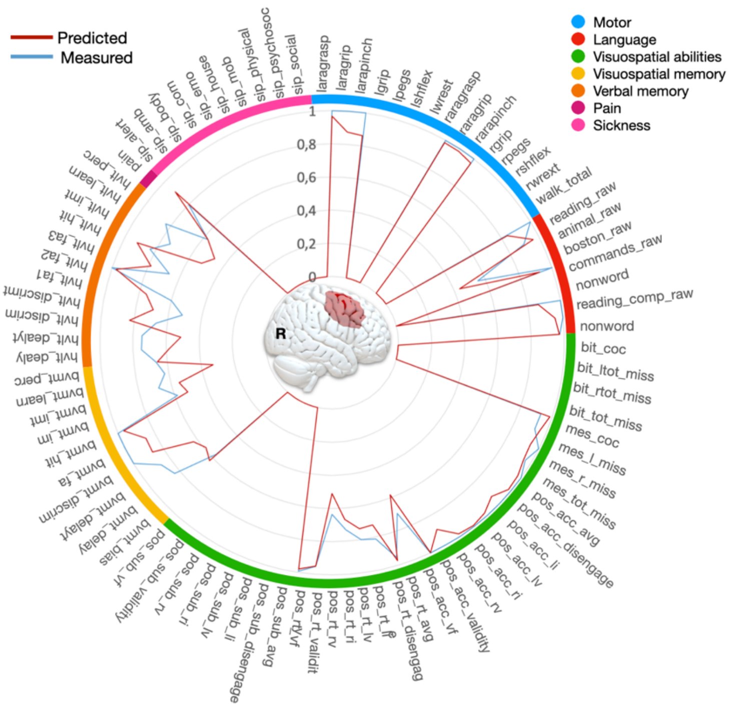

Supplementary Figure 77: Male, 68 years old, right hemisphere stroke. Note that the polarity of some scores was inverted for readability, so that high scores always indicate better performance. The abbreviations of the scores are explained in Supplementary Table 1.
